# The Synergy of Macrocycles and Heterocycles: A Review of Coordination Behavior, Sensing, and Functional Materials Based on Calixarenes

**DOI:** 10.3390/molecules31173079

**Published:** 2026-09-01

**Authors:** Valeriy Kalinin, Pavel Padnya, Ivan Stoikov

**Affiliations:** A.M. Butlerov’ Chemistry Institute, Kazan Federal University, 420008 Kazan, Russia; valikalinin@kpfu.ru

**Keywords:** heterocycles, calixarenes, supramolecular chemistry, coordination chemistry, molecular recognition, catalysis, biomedical applications, structure–property relationships, smart materials

## Abstract

Heterocycle-containing calixarenes have attracted increasing attention over the past three decades owing to the unique combination of the preorganized macrocyclic framework of calixarenes with the structural and functional diversity of heterocyclic building blocks. The incorporation of heterocycles significantly expands the coordination, supramolecular, electronic, catalytic, and biological properties of these macrocycles, enabling the rational design of multifunctional molecular systems for a wide range of applications. This review provides a comprehensive overview of the synthesis, structural diversity, and applications of heterocycle-containing (thia)calixarenes reported over the last 30 years. Synthetic methodologies are critically analyzed and classified into convergent and divergent approaches, with emphasis on their advantages, limitations, and synthetic scope. Particular attention is devoted to the effects of linker type, heterocycle structure, substitution pattern, and macrocyclic conformation on the physicochemical properties and functional behavior of the resulting compounds. By integrating synthetic strategies with a systematic analysis of structure–property relationships, this review highlights how molecular design governs the performance of heterocycle-containing calixarenes across coordination, supramolecular, sensing, catalytic, and biomedical applications. Finally, current challenges and emerging research directions are discussed, providing perspectives for the rational development of advanced calixarene-based functional systems.

## 1. Introduction

Heterocyclic systems form the structural basis of a vast array of compounds with diverse applications. These applications range from analytical purposes, such as creating indicators for titrimetry [1], extraction [2,3], transmembrane transport into a living cell [4], voltammetry [5], organic electronics [6], biomedical goals [7,8,9,10,11,12,13,14,15,16], diagnostic tools [17,18,19]. Heterocycles are a large group of natural compounds that includes plant alkaloids [20,21,22,23,24,25,26,27,28]. Heterocyclic moieties play a key role in the structure of ligands for coordination chemistry [29,30]. The presence of nitrogen, oxygen, or sulfur atoms in the cycle provides effective donor centers that bind to metal ions [31]. Heterocycles are capable of forming strong coordination bonds due to unshared electron pairs from heteroatoms and their participation in donor–acceptor interactions through the π-system [32,33,34]. Including heterocycles in the ligand framework allows one to vary the electronic and steric properties of the complex, thereby regulating its geometry, stability, and reactivity [35]. Additionally, condensed and polyfunctional heterocyclic systems contribute to the formation of chelate and polydentate ligands, increasing the thermodynamic and kinetic stability of complexes [36]. For these reasons, heterocyclic fragments are widely used in creating catalysts [37], sensors [38], and materials with specified properties [39,40,41], as well as biomimetic models of metalloenzymes [42], etc. In recent decades, new data on S*_N_*^H^ reactions has emerged, enabling the creation of polyheterocyclic systems with various properties and structures [43], as well as, for example, the use of *N*-heterocyclic carbenes [44].

In modern supramolecular chemistry, there is a steady trend toward the creation of hybrid molecular systems in which the macrocyclic calixarene platform is used as a structural scaffold for the covalent attachment of heterocyclic fragments. This approach combines the advantages of a preorganized macrocyclic backbone with the functional diversity of heterocycles, opening up new possibilities for directed molecular design. The three-dimensional organization of the calixarene platform ensures conformational preorganization and the possibility of multicenter functionalization, whereas the heterocyclic substituents act as coordination, catalytic, sensing, or biologically active groups. Consequently, the combination of these two structural elements allows for the creation of multifunctional molecular systems whose properties are determined not only by the nature of the individual fragments but also by their spatial arrangement relative to one another on the macrocyclic platform.

Calixarenes are the most widely studied macrocyclic platforms [45,46]. Modifying this platform with heterocyclic fragments opens up broad prospects for practical applications in various fields of science and industry. Combining the potential of calixarenes and heterocycles in a single structure offers several advantages:

(1) Calixarenes are a convenient platform for modification by various methods at both the upper and lower rims. This allows different functional groups to be combined on one or two sides of the macrocycle, giving the macrocyclic derivatives a wide variety of properties. For example, the introduction of both positive and negative charges, as well as long hydrophobic fragments, gives the derivatives surfactant properties; recognition and self-assembly of macrocycle derivatives with biologically significant substrates such as proteins [47], nucleic acids [48,49], etc. Additionally, a macrocyclic platform can be used to create the specific spatial arrangement of heterocyclic structures necessary for various tasks at the intersection of supramolecular and coordination chemistry. One important example is the targeted delivery of biologically active compounds to organs and cells [50,51,52,53,54], obtaining metal complex catalysts [55,56], use of calixarene derivatives as chelating extractants for transition metals [57], synthesis of materials with predetermined properties [58,59,60,61,62,63], synthesis of macrocyclic ionic liquids [64,65,66], etc.
(2) Heterocycles can coordinate with substrates through various mechanisms. For example, they can coordinate with Lewis acids through the lone pair of a nitrogen atom. Aligning heterocyclic binding sites with the rigid, preorganized structure of calixarenes enables the creation of selective ionic and molecular receptors.
(3) Many heterocycles have been described as interacting with biologically important molecules, such as DNA and RNA, due to intercalation between nitrogenous base pairs [67], binding in major [68] and minor [69] DNA grooves, and through Coulomb interactions [70]. Ideally, binding would be possible not only between any pair of nucleotides, but also in cases of multiple pattern matches. This would allow for selective binding to specific sequences [71]. Introducing heterocyclic fragments into the composition of macrocyclic platforms allows one to exploit the advantages of both structures [50,72,73].
(4) Heterocyclic motifs can form the structural core of known drugs and biologically active compounds. In this case, calix[4]arene can serve as the basis for creating a prodrug that is hydrolyzed via an ester bond, releasing the drug [74]. It can also serve as the basis for stable mimetics of biologically active compounds, such as calix[4]arene derivatives containing saccharide fragments of different natures and molecular weights [75,76]. Recent review articles [77,78] on macrocycles with antibacterial properties and other biological applications can provide readers with more detailed information on this topic.
(5) Many different approaches exist for introducing heterocyclic fragments into molecules. The chemistry of calixarenes and related macrocyclic platforms is extensive, opening up virtually unlimited possibilities for combining heterocyclic fragments of various types with calixarenes using different linkers. This is particularly useful for creating large libraries of compounds obtained by modifying a single lead molecule.

**Figure 1:**
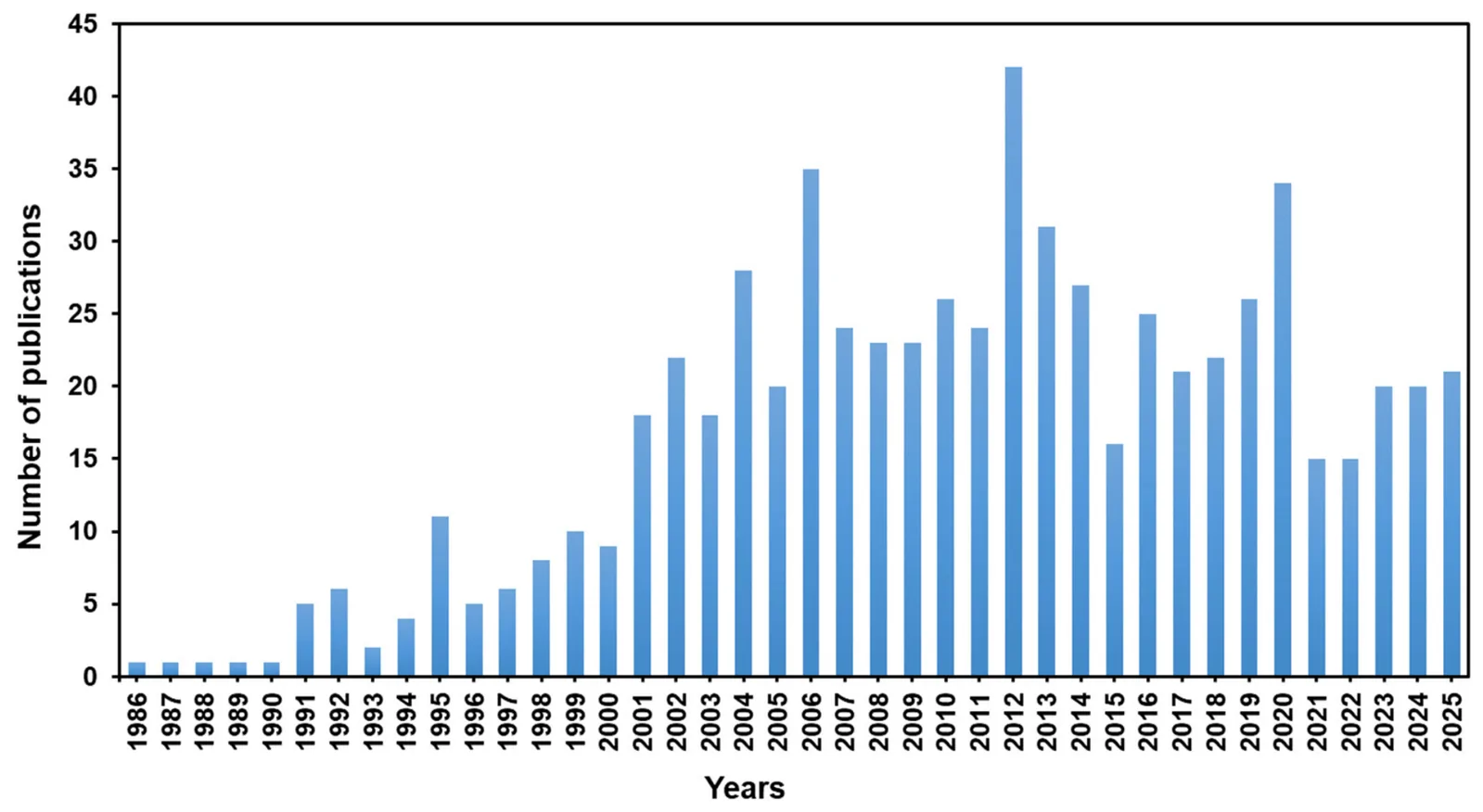
Scopus search results for titles, abstracts, and keywords for the query “(calix[4]arene OR Calixarene OR Thiacalixarene OR Thiacalix[4]arene) AND (Benzimidazole OR Benzofurazan OR BODIPY OR Furan OR Imidazole OR Indole OR Morpholine OR Oxadiazole OR Phenothiazine OR Phthalimide OR Piperidine OR Porphyrin OR Pyrazole OR Pyridine OR Pyrrole OR Pyrrolidine OR Quinoline OR Quinolone OR Tetrathiafulvalene OR Thiazole OR Thiophene OR Triazine OR Triazole)”.

Scopus analysis of titles, abstracts, and keywords showed that interest in calixarenes modified with heterocyclic fragments (**HetCA**s) has been steadily increasing since 1991, peaking in publication numbers in 2012 (more than 40 papers per year) and plateauing at approximately 20 papers per year to date (Figure 1). It is worth noting that this analysis is not exhaustive, since it most likely does not include, for example, macrocyclic conjugates of *L*-tryptophan, which is an indole derivative, but at the same time, the name of the heterocycle itself will rarely be mentioned in the text.

For this article, the literature data were selected through Google Scholar, PubMed, and Scopus systems by mentioning the words “calixarene” or “thiacalixarene” along with the names of heterocycles from the list indicated in the title of Figure 1. The search covered the period from 1986 to 2025. After removal of duplicates, other articles were screened based on titles, abstracts, and, in cases of full-text article availability, contents. Articles were excluded if they did not deal with calixarene-based systems (e.g., resorcinarenes, cyclodextrins, or pillararenes), if the heterocyclic fragment was not covalently attached to the macrocycle, or if the study focused solely on theoretical calculations without experimental synthesis or characterization. Following full-text articles were included in the final analysis. Additional references were identified through cross-referencing the bibliographies of key review articles.

The last review devoted to methods for obtaining heterocyclic derivatives of calixarenes was published as a chapter in a book in 2009 [79], in which the applied aspects of **HetCA**s are practically not covered. Furthermore, **HetCA** chemistry has changed and expanded significantly in the more than 15 years since then. This review article not only serves as a compendium of synthetic and applied articles, but also represents a comprehensive analysis of the topic of **HetCA** derivatives. First, we provide a critical comparative assessment of the available synthetic methodologies, highlighting their respective scope, limitations, and complementarity. Second, we introduce a dedicated structure–activity relationship framework that correlates the nature, position, and number of heterocyclic substituents, as well as the macrocyclic conformation, with the resulting physicochemical and functional properties. Third, we demonstrate how these structural features govern the performance of **HetCA**s in diverse application areas, including coordination chemistry, catalysis, sensing, and biomedicine. This integrated approach, which bridges synthetic chemistry and functional design, represents the principal novelty of the present review and provides readers with a comprehensive toolbox for the rational development of **HetCA**-based systems.

This review consists of four main sections. Section 2 provides a brief history and overview of the chemistry of (thia)calix[4]arenes. Section 3 describes synthetic approaches for obtaining **HetCA**s, with particular emphasis on convergent and divergent strategies for the introduction or construction of heterocyclic fragments on the macrocyclic platform. Section 4 provides a critical analysis of the synthetic strategies employed for the preparation of **HetCA**s, followed by a systematic evaluation of the structure–activity relationships. Particular attention is paid to the effects of the macrocycle type, degree of substitution, and the nature of the heterocyclic fragments and linkers on the physicochemical and biological properties of the resulting **HetCA**s. Building on these structural insights, Section 5 describes the applications of materials based on **HetCA**s. These applications include coordination chemistry, analytical chemistry, supramolecular chemistry, biomedical applications, as well as catalysis. Thus, the review follows a logical progression from synthesis and molecular design, through structure–property relationships, to functional applications, highlighting how structural modifications of **HetCA**s translate into their diverse properties and practical capabilities.

## 2. Calixarenes and Related Macrocycles: Brief History and Terminology

**Figure 2:**
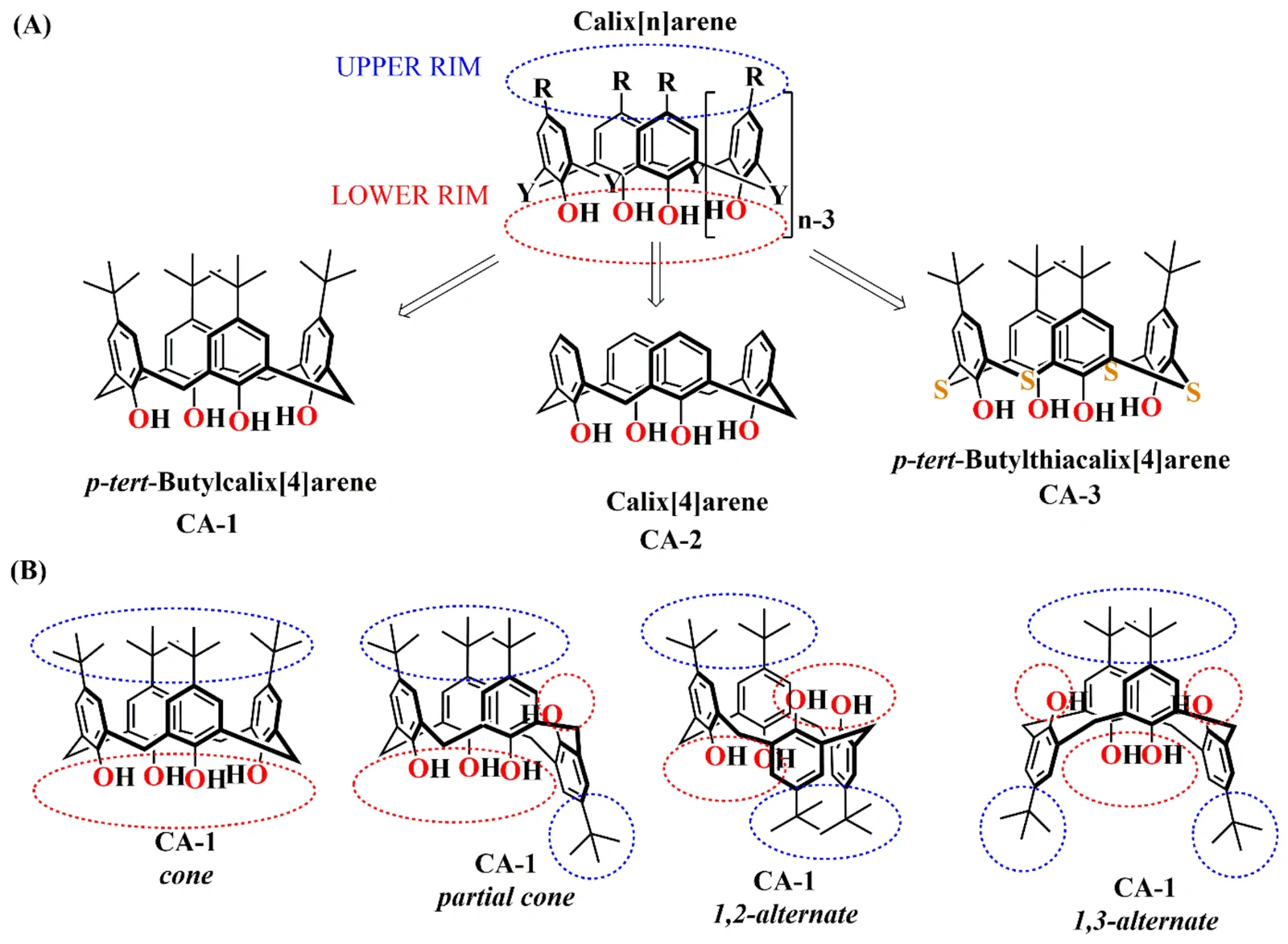
(**A**) Some of calix[4]arenes representatives; (**B**) stereoisomeric forms of *p*-*tert*-butylcalix[4]arene **CA-1**.

Calixarenes represent a class of *meta*-cyclophane derivatives of phenol (Figure 2A), connected by a linker Y which may be, for example, one of the following fragments: a methylene group CH_2_ or sulfur (like **CA-1**–**CA-3**). In the first case, when it comes to classical calixarenes, traces of which are certainly present in the condensation products of phenol and formaldehyde by A. von Baeyer in 1872 [80]. This was followed by the work of A. Zinke and E. Ziegler, who isolated *para*-substituted calixarenes for the first time in 1944 [81]. However, the first synthetically valuable method was proposed by C. D. Gutsche and R. Muthukrishnan in 1978 [82], who used 4-*tert*-butylphenol and formaldehyde for condensation in the presence of potassium hydroxide to synthesize **CA-1**. The sterically hindered *tert*-butyl group enhanced the regioselectivity of the reaction to produce *meta*-cyclophane while also rendering the molecule more resistant to conformational changes. The calixarene structure is defined by the number of monomeric fragments. Currently, the following main types of calix[n]arenes are distinguished depending on their number: calix[4]arenes, calix[5]arenes [83,84], calix[6]arenes [85,86], calix[7]arenes [87], and calix[8]arenes [88]. As demonstrated in the research conducted by Stewart and Gutsche, calixarenes can contain up to 20 phenol moieties [88].

This review focuses primarily on calix[4]arenes with four phenolic fragments. calix[4]arenes are the most widely used due to their synthetic availability and ease of conformation control using the template effect. This feature makes it relatively easy to obtain a series of isomeric derivatives, differing only in their stereochemistry. The following conformations are distinguished for calix[4]arenes: *cone*, *partial cone*, *1,2-alternate*, and *1,3-alternate* (Figure 2B). It is important to note that the *1,2-alternate* conformation is not very significant, as it is difficult to synthesize. However, recent research has identified potential applications for this particular conformation [89,90]. Alkali metal cations such as sodium, potassium, and cesium are used as templates, which coordinate with the nucleophilic centers of *meta*-cyclophane, stabilizing one or another form depending on the size of the cation [91]. Therefore, the sodium cation, the smallest of these three cations, is the most effective stabilizer of the most compact stereoisomeric form—*cone* conformation—while the bulky cesium cation, on the contrary, stabilizes *1,3-alternate*. The potassium cation stabilizes the intermediate stereoisomeric form, *partial cone*, in the case of *p*-*tert*-butylcalix[4]arene.

**Scheme 1:**
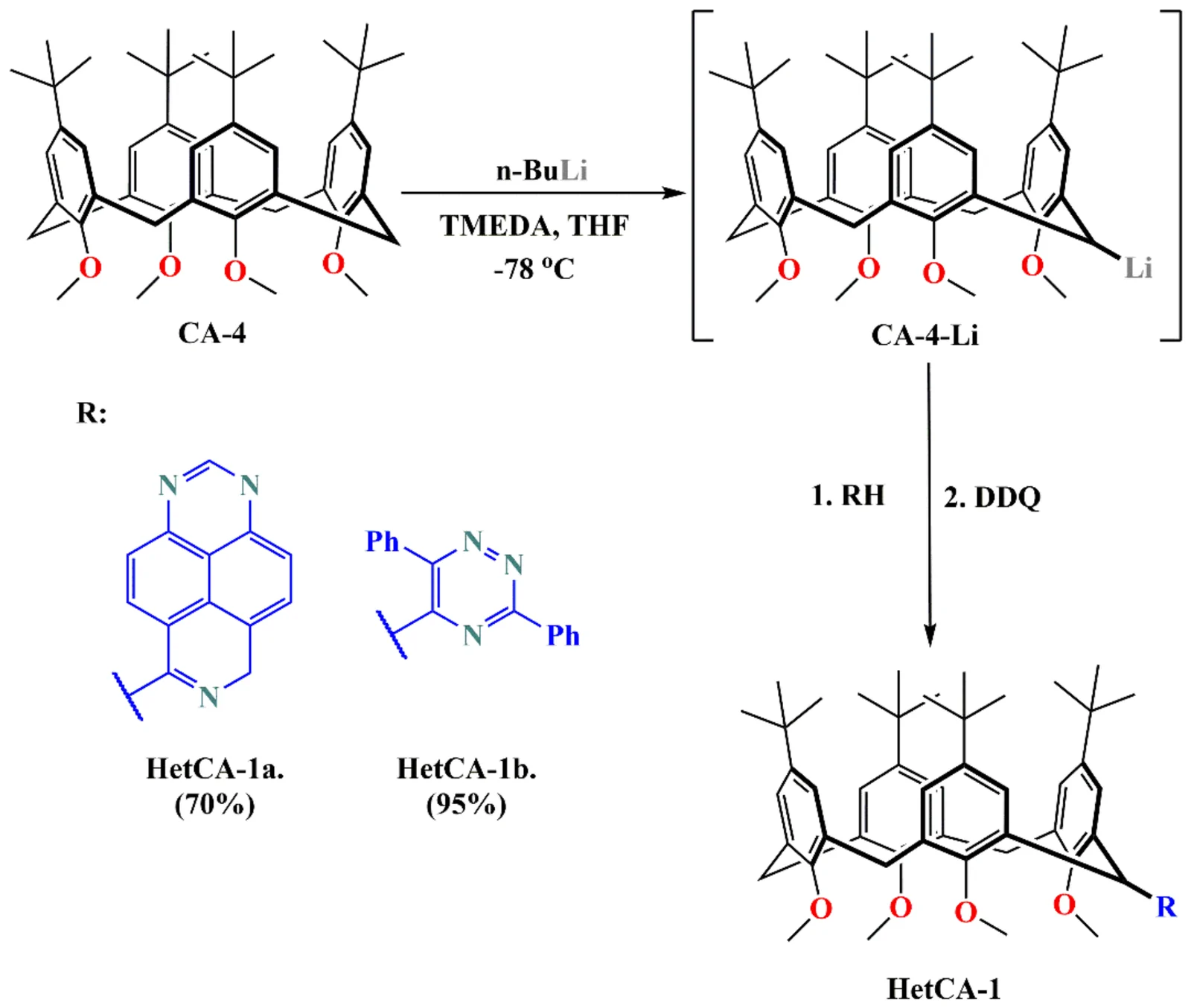
Synthesis of *meso*-functionalized calix[4]arenes [98,100].

As a rule, the upper rim of calix[4]arenes is modified with a *tert*-butyl group. It acts as a steric hindrance, which is necessary for the synthesis of calixarenes with satisfactory yields and directs the electrophilic substituent to the *ortho* position relative to the hydroxyl group. There are number of methods for carrying out dealkylation, for example, the Friedel–Crafts retro reaction, which allows an additional aromatic reaction center to be obtained in the macrocycle at the site of the alkyl moiety like in calixarene **CA-2** [92], which can be nitrated with nitric acid [93], sulfonated with sulfuric acid [94], or introduced into the diazotization reaction [95,96]. By altering the functional groups on the rims (in both the lower and upper ones [97]), calixarene derivatives can be given different solubilities in various solvents. This allows for precise adjustment of the hydrophilic–lipophilic balance. Numerous methods have been developed for comprehensive modification in all four positions, as well as for partial substitution along with substitution by various groups at the lower rim. In addition, valuable synthetic approaches have been developed that allow the use of the CH acidity of methylene fragments of calix[4]arenes and the production of so-called *meso*-functionalized derivatives as in works [98,99,100,101]. Calixarene **CA-4** reacted with *n*-BuLi in the presence of tetramethylethylenediamine (TMEDA) at low temperature (Scheme 1).

**Scheme 2:**
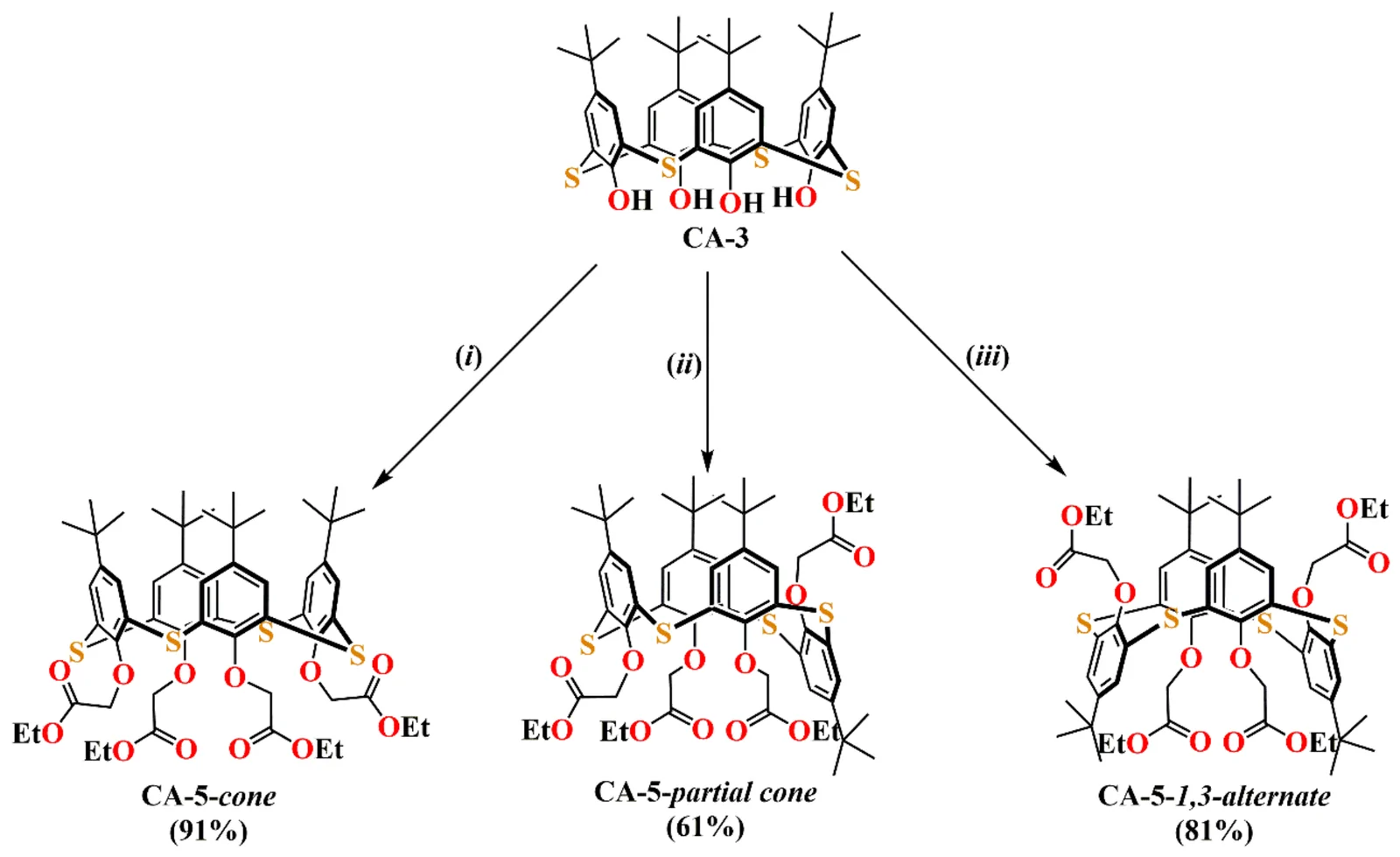
Optimized synthesis of tetraesters based on *p*-*tert*-butylthiacalix[4]arenes using the template effect for the preferential formation of a specific stereoisomeric form. Reagents and conditions: ethyl bromoacetate, acetone, reflux (i) Na_2_CO_3_, 36 h; (ii) K_2_CO_3_, 28 h; (iii) Cs_2_CO_3_, 4 h [108].

In addition to classical calixarenes, sulfur-containing thiacalixarene **CA-3** has also become widespread in modern chemistry. The synthesis of *p*-*tert*-butylthiacalix[4]arene was first reported in 1997 by Sone et al. [102] and Kumagai et al. [103]. The synthesis processes of thiacalix[4]arenes were thoroughly studied and then optimized [104]. Thiacalixarenes are non-toxic platforms characterized by a large intramolecular cavity and slightly different conditions for realizing the template effect when setting a particular conformation. They also have additional “soft” binding centers (sulfur atoms), a hydrophobic cavity, and several modification sites. Typically, the lower (phenolic) and upper rims are modified, while reactions affecting the linker fragments are rare [91]. Also, thiacalix[4]arenes, due to the presence of sulfur atoms, can undergo oxidation to form both sulfinic [105] and sulfonic acid derivatives [106]. This opens up new varieties of the thiacalix[4]arene platform. For *p*-*tert*-butylthiacalix[4]arenes, the most common approach for modification is the synthesis of tetraesters **CA-5** by reacting the macrocycle **CA-3** with ethyl bromoacetate in acetone in the presence of alkali metal carbonates, whose cations cause a template effect in the formation of the stereoisomeric *cone*, *partial cone*, or *1,3-alternate* forms, respectively [107,108] (Scheme 2). For classical *p-tert*-butylthiacalixarenes, the same main modification route is usually not distinguished.

**Figure 3:**
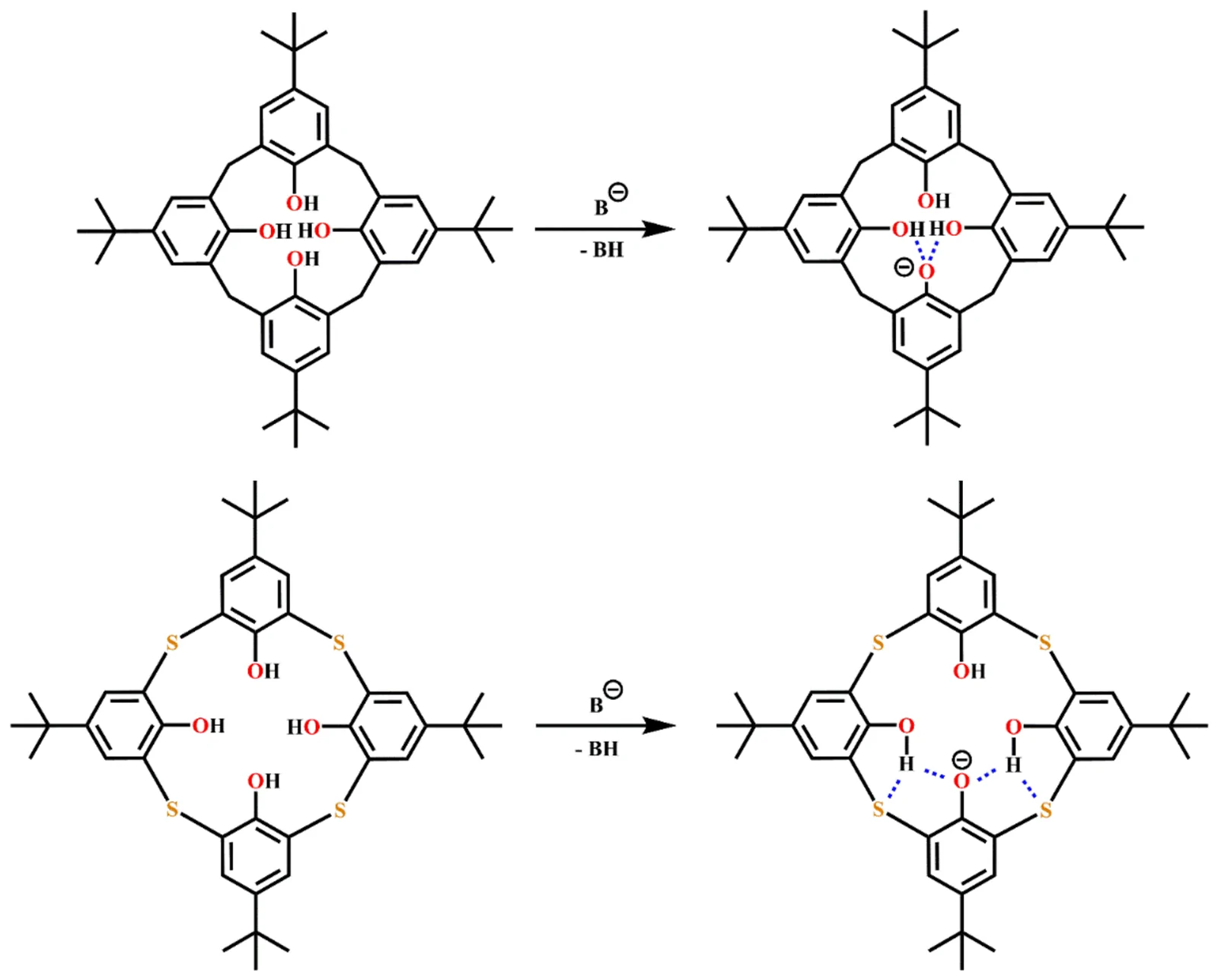
The relationship between the structure of *p*-*tert*-butyl(thia)calix[4]arene and intramolecular hydrogen bonding forces [102,113].

The structure of the starting macrocycle (calixarene or thiacalixarene) is also important to consider, in addition to the structure of the heterocyclic electrophilic agent. This allows prediction of the reaction products and likely yields. A notable distinction between *p*-*tert*-butylcalix[4]arene and its sulfur-containing analogue is the size of the cavity [91]. The smaller cavity in *p-tert*-butylcalix[4]arene leads to the absence of an additional hydrogen bond acceptor (a sulfur atom) that would “dilute” the hydrogen bond (the so-called bifurcated hydrogen bond). This results in stronger hydrogen bonds being formed between the hydroxyl groups on the lower rim of the methylene variant of the macrocycle, compared to thiacalixarene [109,110]. The difference in pK values is more significant as a result (for the classical calix[4]arene—pK_1_ = 3.08, pK_2_ = 12.02, pK_3_ и pK_4_ > 13; for thiacalix[4]arene—pK_1_ = 2.18, pK_2_ = 8.45, pK_3_ = 11.19, pK_4_ = 11.62) [111], which leads to a more expressed stepwise dissociation process and allows for sequential substitution of one, two, three, or four groups in the classical calix[4]arene. Distal, i.e., 1,3-substitution in classical calixarene occurs under relatively mild conditions, requiring carbonate and acetonitrile. At the same time, the substitution of the last unbound hydroxyl, whose negative charge is not stabilized by hydrogen bonds from neighboring groups, often requires the presence of sodium hydride in DMF [112]. However, it is more difficult to achieve partial substitution for thiacalix[4]arene, since the pK values of hydroxyl groups in this case do not differ greatly, and therefore tetrasubstituted derivatives generally predominate, although they are not exclusive (Figure 3).

In addition to the two types of macrocycles described above (classical calixarene and thiacalixarene), there are several others that are much less common, such as azacalixarene [114,115,116], oxa(hetero)calixarenes [117], selena(hetero)calixarenes [118,119], and others [120,121]. Benzene rings of the calixarene scaffold can also be modified in such ways that macroheterocyclic structures are obtained. Further study of these structures is currently pending [122]. It is also worth mentioning such a rapidly developing field as calixheteroarenes (particularly upper rim-bridged calixarenes) [123,124,125], which is discussed further in the review in Section 3.2.2.

Thus, (thia)calix[4]arenes are convenient platforms for modification, capable of existing in the form of three widespread stereoisomers with certain geometrically preorganized parameters. This is particularly valuable for creating new ligands for both inorganic and organic compounds. They are successfully used for analytical purposes, creating synthesis catalysts, and obtaining various groups of drugs, etc. For a detailed overview of the properties of (thia)calixarenes and methods for their synthesis, we can recommend a number of important review papers [91,126,127,128,129,130]. We decided to focus this review only on heterocycle derivatives of calix[4]arenes and thiacalix[4]arenes rather than other macrocyclic systems, e.g., resorcinarenes and other (thia)calix[n]arenes (where *n* > 4). This choice is motivated by the desire for a more in-depth and systematic analysis. calix[4]arenes are the most studied and conformationally predictable representatives of their family and serve as an ideal reference platform. Their sulfur-containing analogs, thiacalix[4]arenes, are purposefully synthesized and provide a unique opportunity to investigate how the nature of the heteroatom in the bridge group influences coordination and supramolecular properties while maintaining an identical aromatic framework. Including resorcinarenes, whose chemistry is oriented toward constructing cavitands or more flexible and less predictable (thia)calix[n]arenes (n > 4), would obscure the study’s focus and prevent a detailed comparative analysis of fundamental structure–property relationships, the main objective of this work.

## 3. Chemistry. Synthetic Approaches

The key difference between the alkylation of (thia)calix[4]arenes and that of simple phenolic compounds is the need to control the conformational composition of the final products. Unlike acyclic systems, where conformational dynamics generally have little influence on reactivity, in calixarenes the choice of base serves not only as a deprotonating agent but also as a factor governing the preferential formation of one of the four possible conformations (*cone*, *partial cone*, or *1,3-alternate*).

In addition, (thia)calix[4]arenes possess several structural features that distinguish them from their low-molecular-weight analogs. These include the presence of four functional groups within a single macrocyclic framework, pronounced steric hindrance, and the poor solubility of both the parent macrocycles and many of their uncharged derivatives in polar solvents unless hydrophilic substituents are introduced. Although these are intrinsic properties of the macrocyclic scaffold rather than disadvantages, they must be taken into account when designing synthetic strategies.

For example, the hydroxyl groups of calix[4]arenes are not equivalent. In classical calix[4]arenes, intramolecular hydrogen bonding results in stepwise deprotonation of the phenolic groups. This enables selective mono- and disubstitution, whereas complete substitution is more difficult to achieve. In contrast, the weaker hydrogen bonding in thiacalix[4]arenes leads to smaller differences in the pKa values of the hydroxyl groups, making selective partial functionalization considerably more challenging. Thus, the reactivity of calix[4]arenes and thiacalix[4]arenes is governed not only by the nature of their functional groups but also by the structural organization of the macrocyclic framework, which distinguishes them both from low-molecular-weight compounds and from each other.

**Figure 4:**
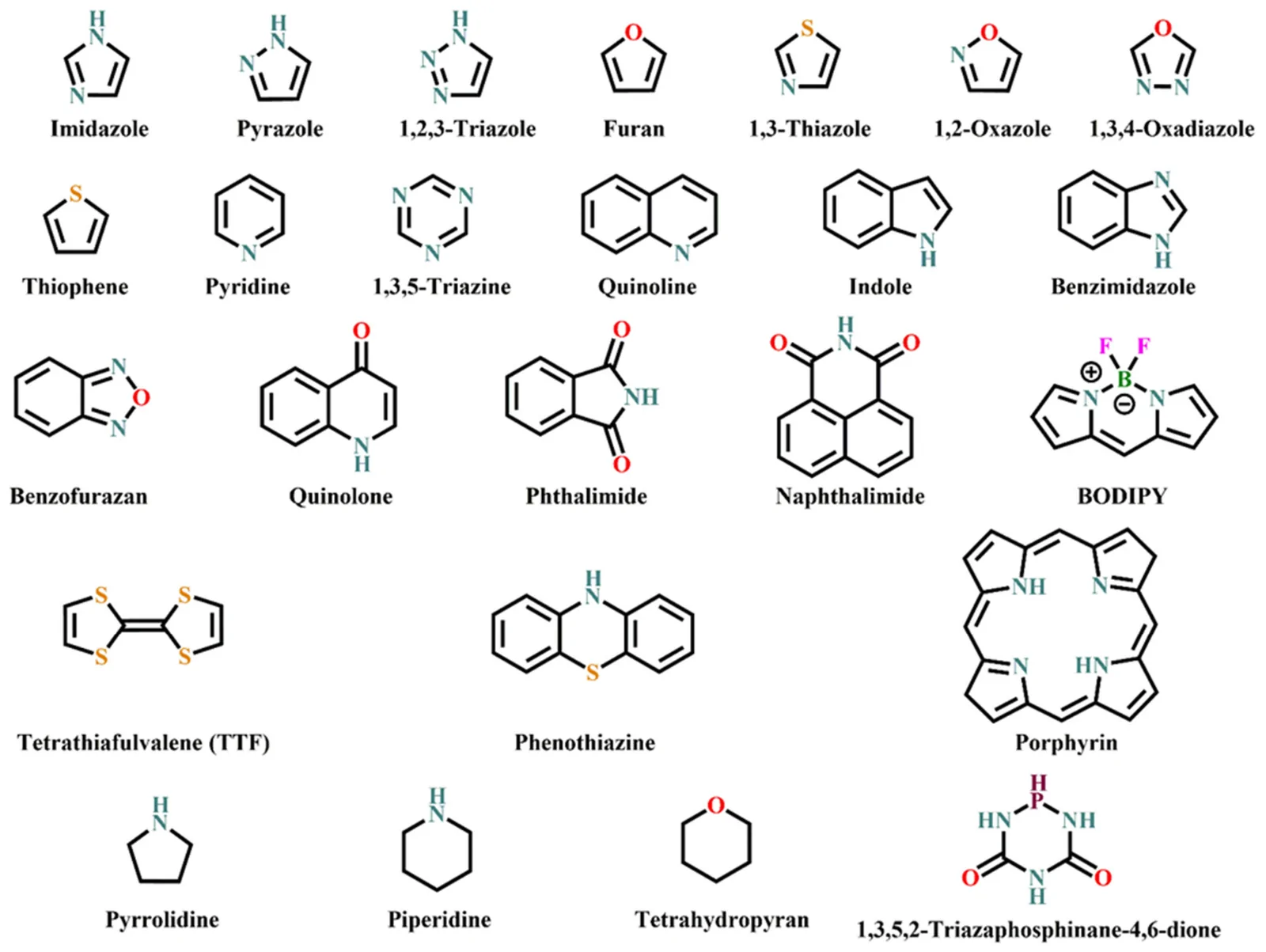
Examples of heterocyclic fragments forming **HetCA** derivatives.

The variety of heterocyclic fragments that comprise **HetCA**s is wide. Figure 4 illustrates the main heterocyclic fragments based on calixarene derivatives discussed in subsequent pages of this article. The heterocycles used can be nitrogen- or oxygen-containing, sulfur-containing, or contain various types of heteroatoms. Heterocycles can be single or fused.

Among the presented structures (Figure 4), several groups of heterocyclic substituents can be conditionally distinguished, divided by the area of application and partly overlapping: (1) donor heterocycles, which are introduced to impart coordination properties to the macrocycle. Structures of this kind include imidazole, pyrazole, pyridine, 1,2,3-triazole, 1,3,5-triazine, quinoline, indole, benzimidazole, and thiophene; (2) heterocycles acting as chromo- and fluorophores, namely BODIPY, phthalimide, naphthalimide, benzofurazan, phenothiazine and porphyrin; (3) heterocycles with pronounced pharmacophoric properties: indole, 1,3,4-oxadiazole, 1,3-thiazole, pyrrolidine, piperidine, tetrahydropyran; (4) π-deficient heterocycles for conducting materials and organic electronics, such as tetrathiafulvalene (TTF), thiophene, furan; (5) other heterocycles that play an important role in the creation of catalysts: triazaphosphinane, imidazole and benzimidazole; (6) heterocycles used for subsequent reactions, such as phthalimide for the Gabriel reaction. For a detailed discussion of the application areas of **HetCA**s, see Section 5.

**Figure 5:**
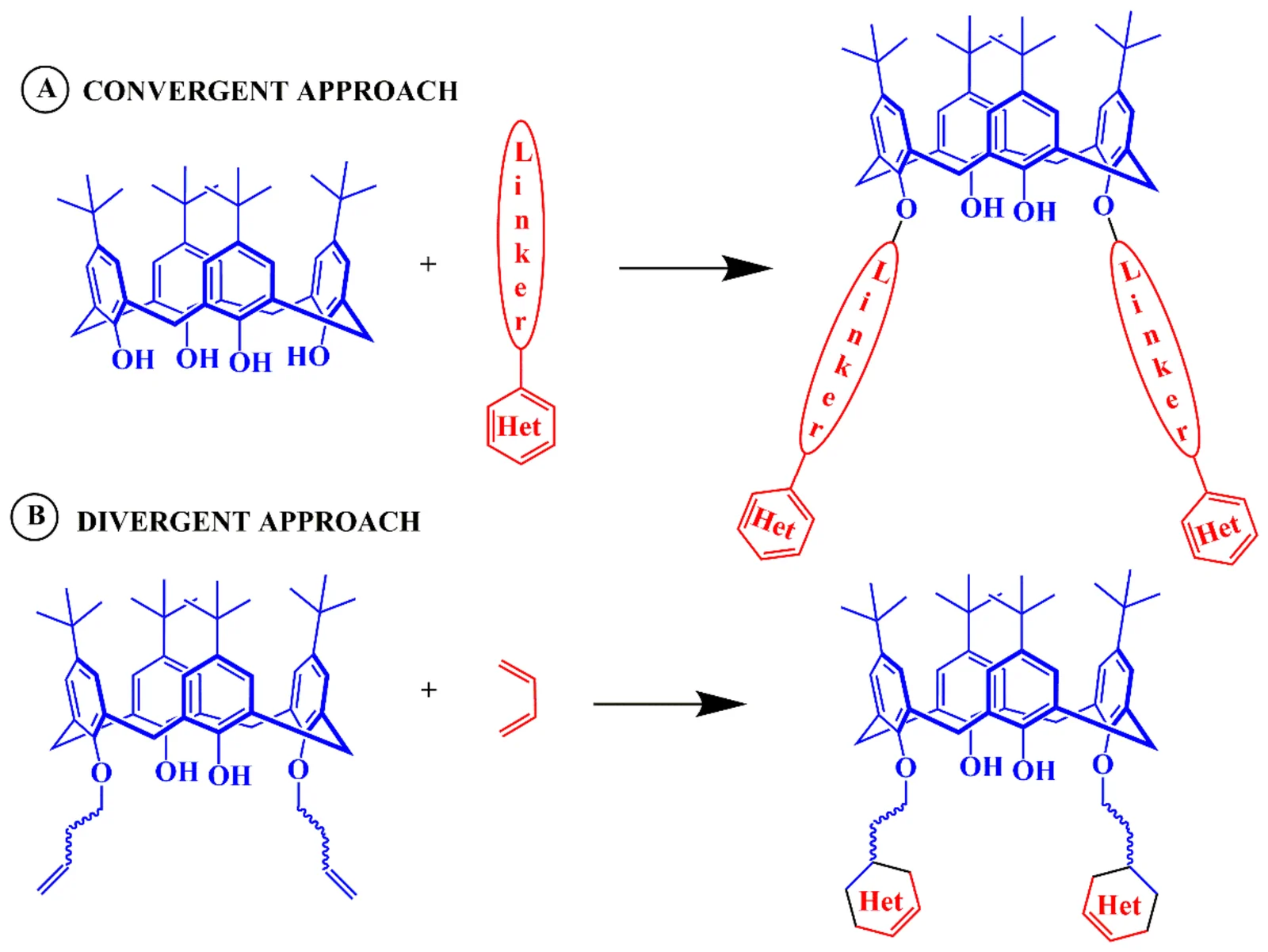
Representation of convergent (**A**) and divergent (**B**) approaches of **HetCA**s synthesis.

There are two main approaches to obtaining hybrid calixarene–heterocyclic compounds (**HetCA**s), i.e., convergent (Figure 5A) and divergent (Figure 5B). The convergent approach involves synthesizing the heterocyclic system outside the macrocycle and then introducing it into the macrocycle. The second approach, divergent, differs from the first in that the heterocyclic fragment is synthesized in stages directly on the calixarene.

Convergent methods typically introduce a wide range of aromatic heterocyclic and fused systems into macrocycle substituents, e.g., pyridine, bipyridine, thiazole, quinoline, imidazole, BODIPY, 1,3,5-triazine, and crown ethers. This group also includes heterocyclic fragments found in amino acids, carbohydrates, and other biologically active molecules. In contrast, divergent approaches form a heterocyclic ring directly on an already functionalized macrocyclic platform. The most common derivatives obtained in this manner include those containing 1,2,3-triazole, tetrazole, 1,2,4-oxadiazole, 1,3,5-triazine, phthalimide, naphthalimide, and porphyrin fragments. It should be noted that for most of the heterocyclic systems listed, there are also convergent methods for introducing calixarenes into the structure; however, it is the divergent methods that often prove to be more effective in terms of the availability of starting compounds, modularity of synthesis, and the diversity of the derivatives obtained.

An analysis of the extensive synthetic literature on this topic revealed that the convergent approach (Section 3.1) is implemented much more frequently than the divergent one (Section 3.2), for which relatively few works were found. So, we present an analysis of the main trends in **HetCA** synthesis that have emerged over the last thirty years. Below we provide an overview of the main convergent approaches, namely nucleophilic substitution reactions such as Williamson synthesis, cross-coupling reactions such as the Steglich reaction, azide–alkyne coupling, and others.

The following principal synthetic approaches can be distinguished among the divergent methods of **HetCA** synthesis (Section 3.1):

1. The Williamson-type nucleophilic substitution reactions (Section 3.1.1 and Section 3.1.2);
2. Cross-linking reactions (Section 3.1.3);
3. Azide–alkyne cycloaddition (AAC, Section 3.1.4);
4. Synthesis of Schiff bases, hydrazones, and related compounds (Section 3.1.5);
5. Electrophilic addition to a tertiary nitrogen atom (Section 3.1.6).

Divergent synthesis methods fall into two categories (Section 3.2):

1. Synthesis of heterocyclic fragments in side chains (Section 3.2.1);
2. Synthesis of upper rim-bridged calixarenes (Section 3.2.2).

### 3.1. Convergent Approaches

#### 3.1.1. The Williamson-Type Nucleophilic Substitution Reactions. Part I: Synthesis of HetCAs via Interaction Between Electrophilic Heterocyclic Agents and Macrocycle

**Scheme 3:**
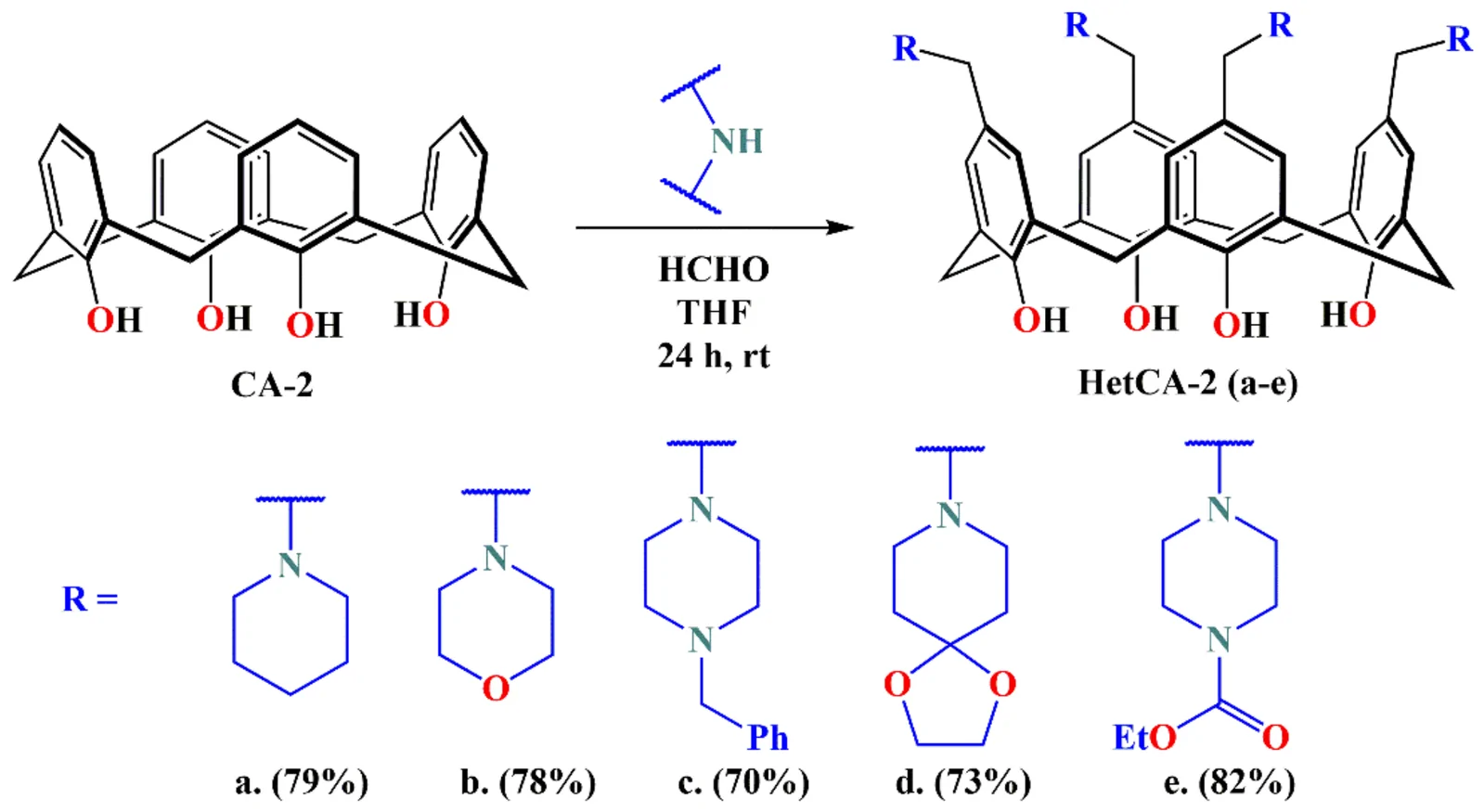
One of the first syntheses of **HetCA**s by Gutsche and Nam [131].

Historically, one of the first examples of the synthesis of **HetCA**s was published by Gutsche and Nam in 1988 [131]. The synthesis was carried out by the aminomethine condensation reaction of heterocyclic amines and formaldehyde with calix[4]arene. The reaction proceeded for 24 h under mild conditions with yields of **HetCA-2** in the range of 70–82% (Scheme 3).

Further development of calixarenes modified with heterocyclic fragments has mainly followed the path of modifying the lower rim. The most common methods for synthesis of **HetCA** compounds are Williamson-type reactions. Furthermore, the electrophilic center can be located both on the calixarene and on the introduced heterocyclic fragment.

**Figure 6:**
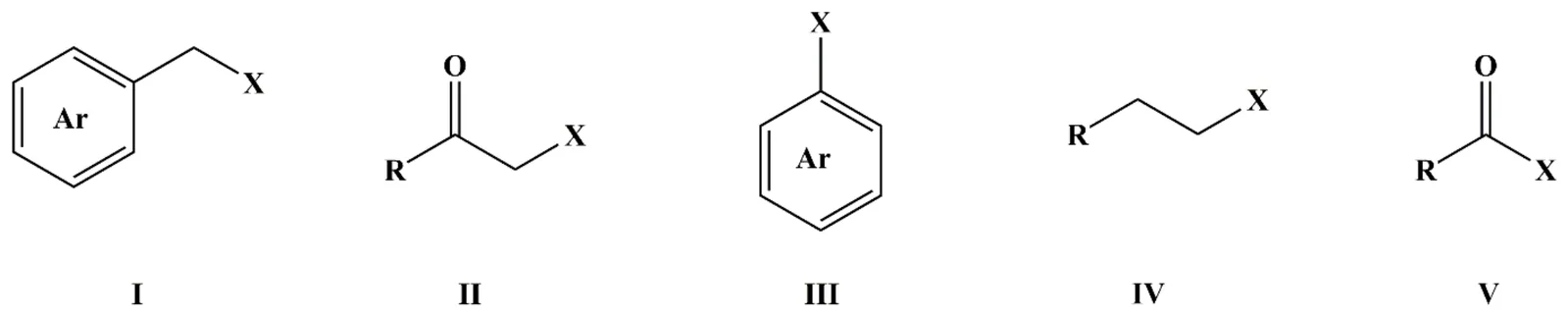
Main types of heterocyclic electrophilic agents. Ar—aromatic (hetero)cyclic fragment, R—fragment containing a heterocycle in any position, X—nucleophile (halogen, tosyl, etc.).

This section examines an approach in which a heterocyclic electrophilic agent is introduced into the calix[4]arene at the lower rim. In terms of the structure of heterocyclic electrophilic agents (Figure 6), they can be classified according to the following main types:

**I**. Benzene-type electrophilic agents containing halogen in the α-position to the aromatic fragment (Ar-CH_2_-X);

**II**. Electrophilic agents containing halogen in the α-position to the carbonyl group (R-CO-CH_2_-X);

**III**. Electrophilic agents containing a halogen in p-π-conjugation with a heterocyclic aromatic fragment (Ar-X);

**IV**. Electrophilic agents whose positive charge is not stabilized by the influence of a neighboring group (R-CH_2_-CH_2_-X);

**V**. Acylating agents.

The first three groups of electrophilic reagents (**I**–**III**) can be classified as strong, since they form stable carbocations of the benzylic or α-carbonyl type, whose positive charge is due to the acceptor effect of heteroatoms in the heterocyclic structure. Electrophilic agents from group **IV** react mainly via the S_N_2 mechanism, which often requires more severe conditions and often leads to lower yields compared to the previous three cases. The group of heterocyclic acylating agents **V** stands apart, which can be represented not only by acyl halides, but also by halo sulfones.

Within the scope of these two approaches, calixarenes interact with heterocyclic electrophilic derivatives via phenolic hydroxyls. In this review article, we chose not to focus on the nature of the bridge group in most cases, unless it resulted in a change in properties, given that thiacalix[4]arenes are less common than their methylene analogs. In most cases, heterocyclic fragments are introduced distally along two phenolic hydroxyl groups (1,3-disubstitution) [59,132,133,134,135,136,137,138,139,140,141,142,143,144,145,146,147,148,149,150,151,152,153,154,155,156,157,158,159,160,161,162,163,164,165,166,167,168,169,170,171,172,173]. Complete tetrasubstitution is also carried out, often involving thiacalix[4]arenes [138,174,175,176,177,178,179,180,181,182,183,184,185,186].

##### Substitution via Type I Electrophilic Reagents

**Scheme 4:**
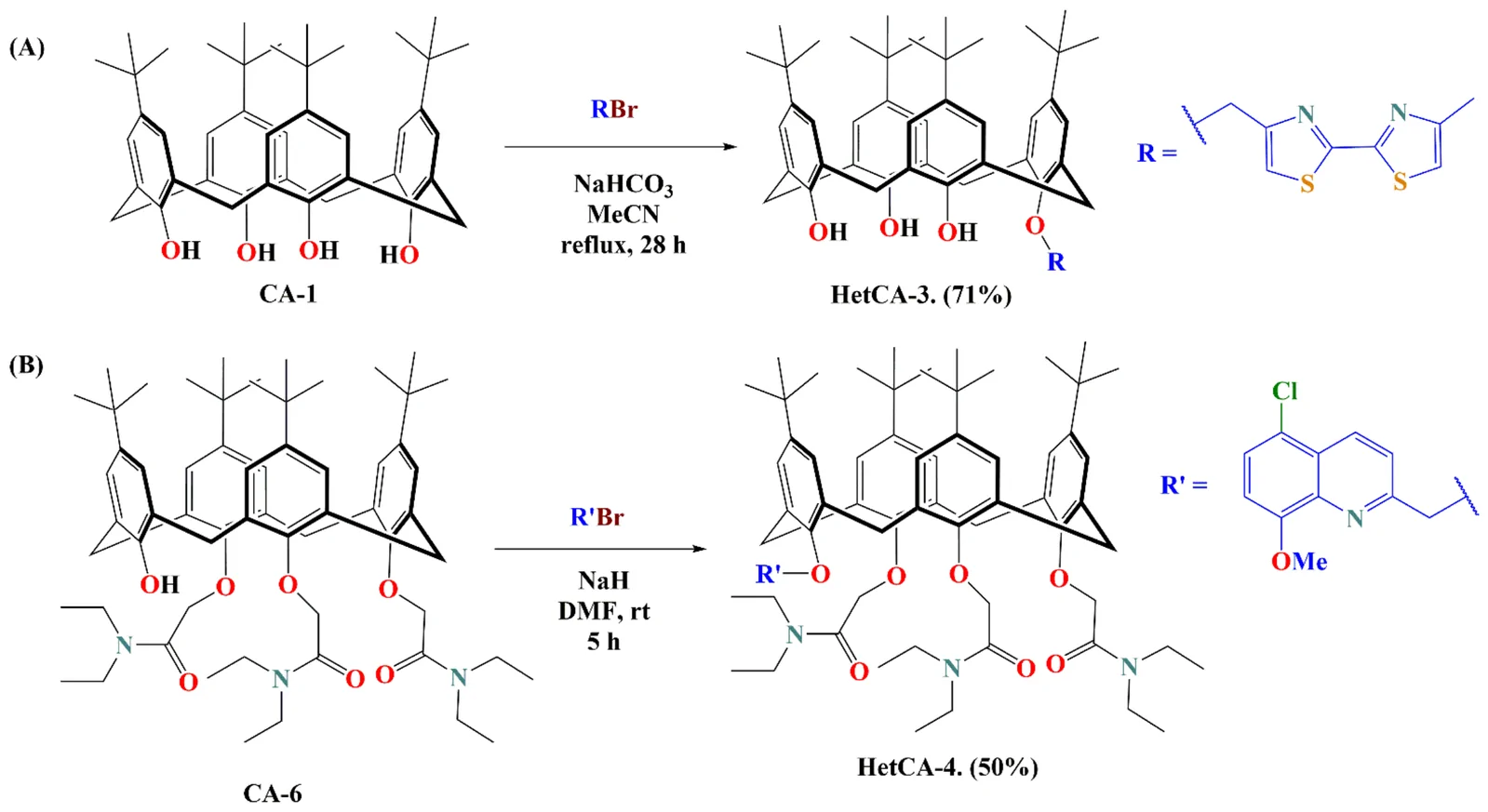
Introduction of a single heterocyclic fragment via type **I** electrophilic agents in the case of (**A**) free *p*-*tert*-butylcalix[4]arene **HetCA-3** [185] and (**B**) trisubstituted CA derivative **HetCA-4** [188].

In general, electrophilic heterocyclic derivatives from group **I** react with calixarenes in the presence of a base with high yields [73,138,140,145,187,188,189]. One heterocyclic fragment can be introduced into the structure of calixarenes. This can be achieved in two ways. The first involves the use of a weak base in acetone or acetonitrile (MeCN) and equimolar amounts of reagents, as in the synthesis of mono-substituted **HetCA-3** (Scheme 4A) [185]. The bithiazole fragment introduced into the macrocyclic framework is known to exhibit intense intrinsic blue fluorescence. The second method involved the use of trisubstituted calixarenes, such as **CA-6**, which have one free hydroxyl group in their structure. In the case of **HetCA-4** synthesis, a strong base is required (as a rule, NaH in *N,N*-dimethylformamide (DMF)), since the acidity constant of a single hydroxyl group in calixarene is quite low (Scheme 4B) [188].

**Scheme 5:**
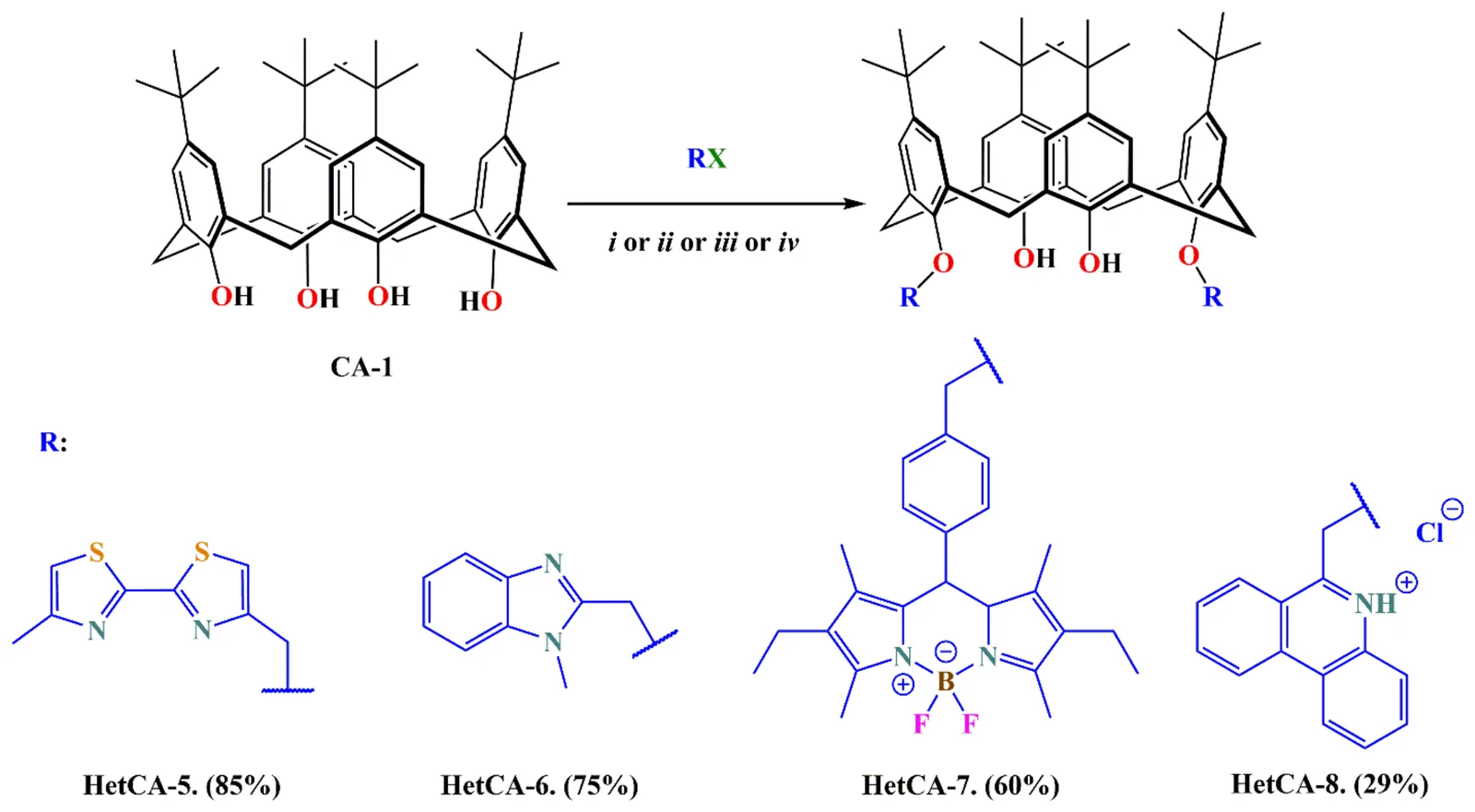
Examples of the synthesis of distal heterocyclic disubstituted derivatives of **CA** with type **I** electrophilic agents. (*i*)—K_2_CO_3_, MeCN, reflux, 5 h; (*ii*)—K_2_CO_3_, MeCN, reflux, 22 h; (*iii*)—DIPEA/TCM/DMF, reflux, 72 h; (*iv*)—K_2_CO_3_, NaI, MeCN, reflux, 48 h [73,138,145,185].

The Williamson reaction is most often used to obtain 1,3-substituted products such as macrocycles **HetCA-5**–**HetCA-8**, which are known as products of distal substitution (Scheme 5) with bithiazole [185] or benzimidazole, and allows the ligand to mimic the histidine coordination environment in copper-containing enzymes [138]. In this case, potassium carbonate is most often used as the base and acetonitrile as the solvent. However, other systems, e.g., DIPEA/TCM/DMF (diisopropylethylamine/chloroform/*N,N*-dimethylformamide), have been reported for producing a BODIPY derivative of **HetCA-8** which exhibits a high binding affinity toward DNA owing to its extended π-conjugated system [73]. To enhance reactivity, electrophilic agents can be involved in the reaction in the presence of sodium or potassium iodides in acetone or acetonitrile (the Finkelstein reaction). In this case, alkylation is preceded by the substitution of chlorine or bromine atoms with iodine, which is a better leaving group than the other two types of halogens [145] (Scheme 5). 1,3-Disubstituted calixarenes possess an optimal number of donor sites for the coordination of structurally diverse substrates. At the same time, the two remaining hydroxyl groups stabilize the *cone* conformation via intramolecular hydrogen bonding, thereby generating a well-defined chelating cavity.

**Scheme 6:**
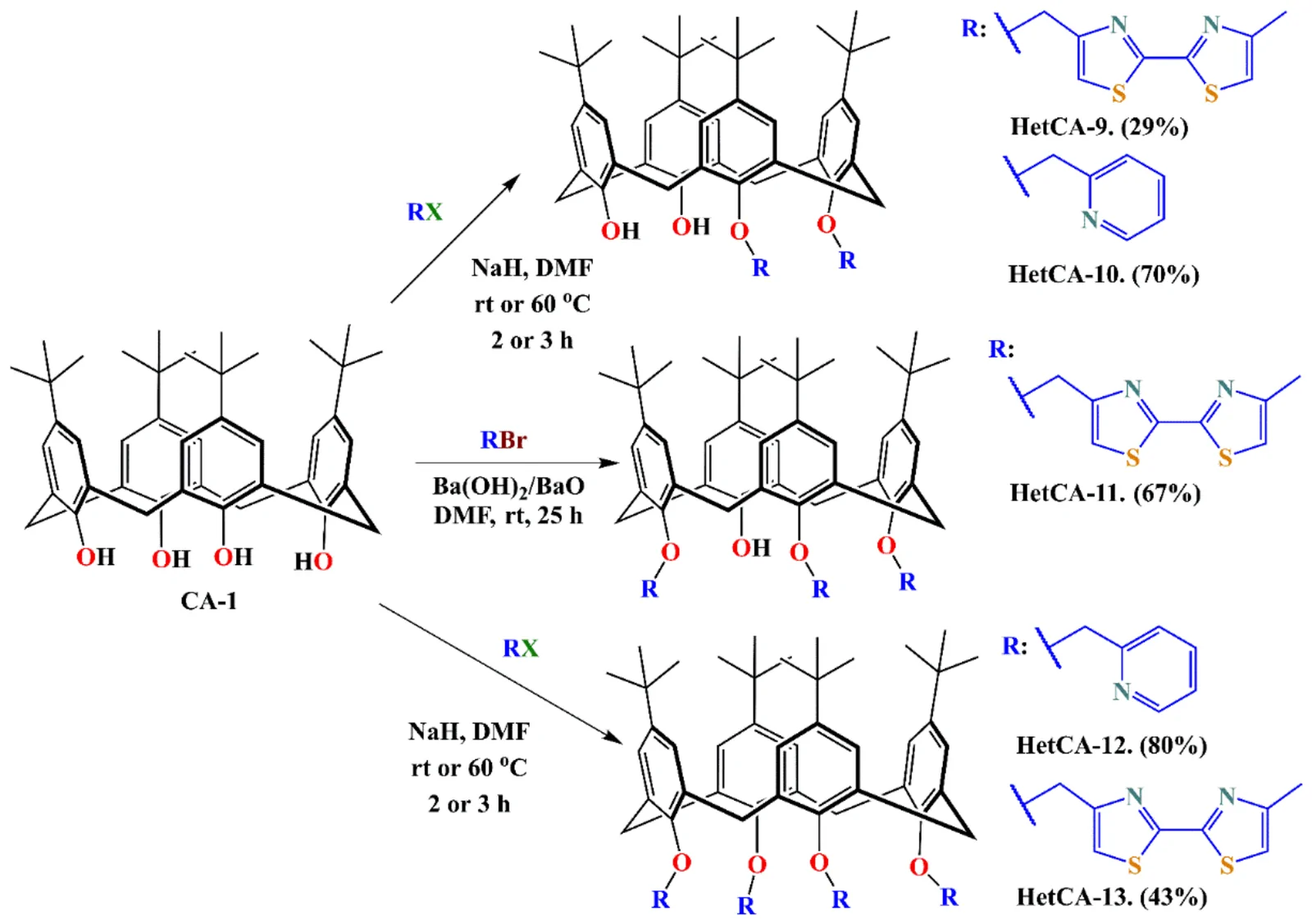
Examples of the synthesis of proximal derivatives **HetCA-9** and **HetCA-10** [153,185], trisubstituted **HetCA-11** [185], tetrasubstituted **HetCA-12** and **HetCA-13** [153,185] via type **I** electrophilic agents.

1,2-Substitution, also known as proximal substitution, occurs much less frequently. It is carried out in the presence of two equivalents of an electrophilic agent and in more basic systems (usually NaH/DMF) compared to distal substitution. For example, replacing the K_2_CO_3_/MeCN system with NaH/DMF resulted in the preferential formation of the proximal **HetCA-9** with 29% yield (Scheme 6) [185]. The low yield in this case can probably be explained by the low reaction temperature. For example, the proximal disubstituted **HetCA-10** can be obtained at 60 °C with a 70% yield (Scheme 6) [153]. The first targeted synthesis of the trisubstituted **HetCA-11** via the Williamson reaction was historically carried out in 1996 by Pellet-Rostaing et al. (Scheme 6). The Ba(OH)_2_/BaO/DMF system at room temperature is used as the base [185]. It should be noted that the synthesis of trisubstituted **HetCA**s derivatives is rare. Tetrasubstituted derivatives of classical calixarenes are synthesized via the Williamson reaction using strong bases, such as NaH. As in previous cases, derivatives of classical calixarenes most often exhibit *cone* conformation. Pappalardo et al. synthesized the pyridine derivative **HetCA-12** at an 80% yield using sodium hydride under mild heating conditions [152] (Scheme 6). Pellet-Rostaing et al. carried out a similar synthesis without heating to introduce bithiazole into the macrocyclic structure and obtain **HetCA-13** (Scheme 6). In this case, the yield was lower at 43% [185].

**Scheme 7:**
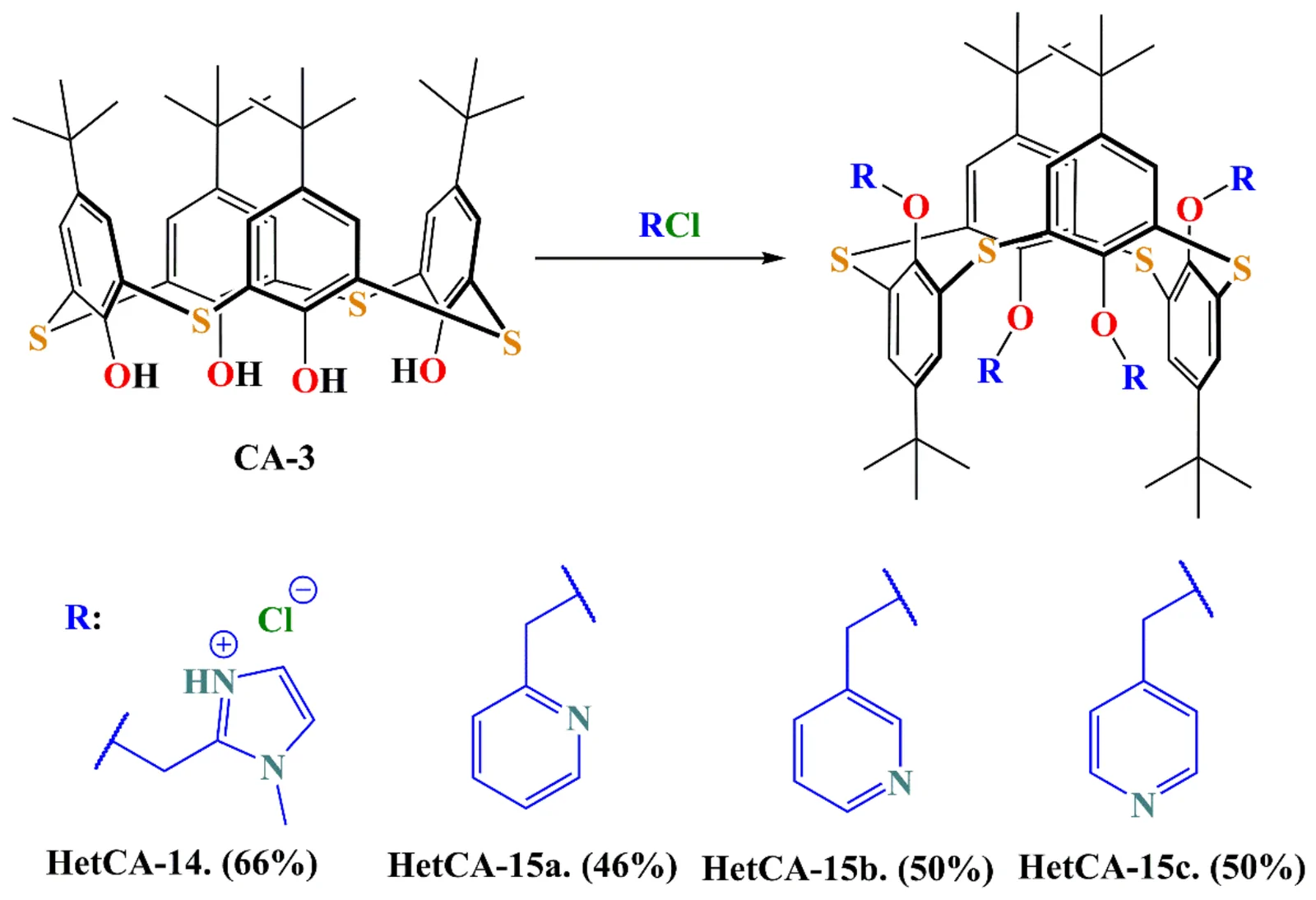
Synthesis of tetrasubstituted **HetCA-14** and **HetCA-15** in *1,3-alternate* conformation via type **I** heterocyclic electrophilic agents (Cs_2_CO_3_, acetone, 16–40 h, reflux) [136,190].

Thiacalix[4]arenes tend to form tetrasubstituted derivatives more easily, and partial substitution products are rare. However, type **I** heterocyclic electrophilic agents are not often used for synthesizing thiacalix[4]arene derivatives. In all examples described in the literature, acetone is used as a solvent and cesium carbonate is used as a base that stabilizes the *1,3-alternate* conformation via the template effect. Thus, Zhao et al. obtained tetrasubstituted thiacalixarene **HetCA-14** with a 66% yield [136], while Ovsyannikov et al. obtained a series of tetrasubstituted pyridine derivatives **HetCA-15** with 46–56% yields [190] (Scheme 7).

**Scheme 8:**
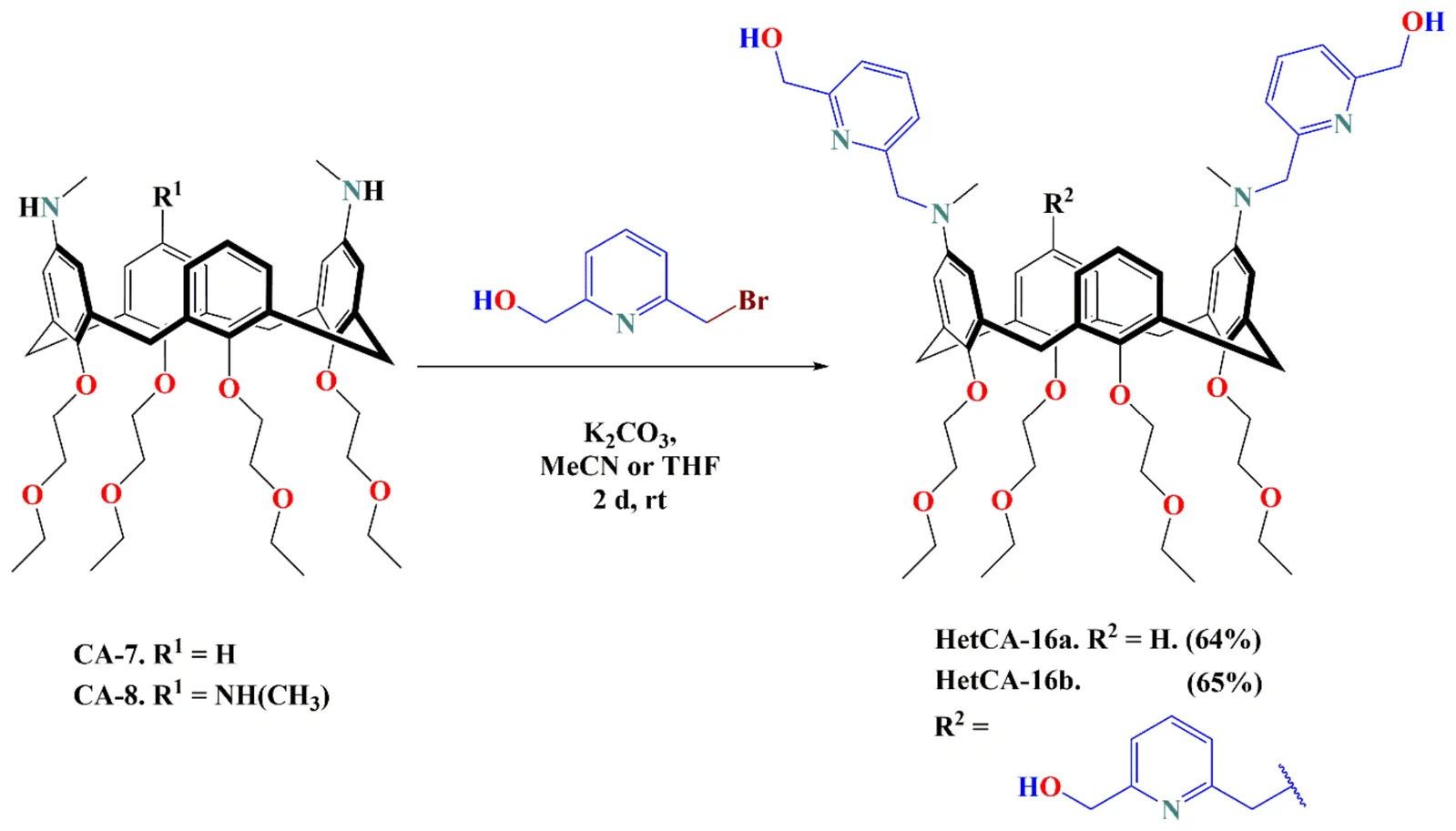
Synthesis of hydromethylpyridine **HetCA-16** [191,192].

**Scheme 9:**
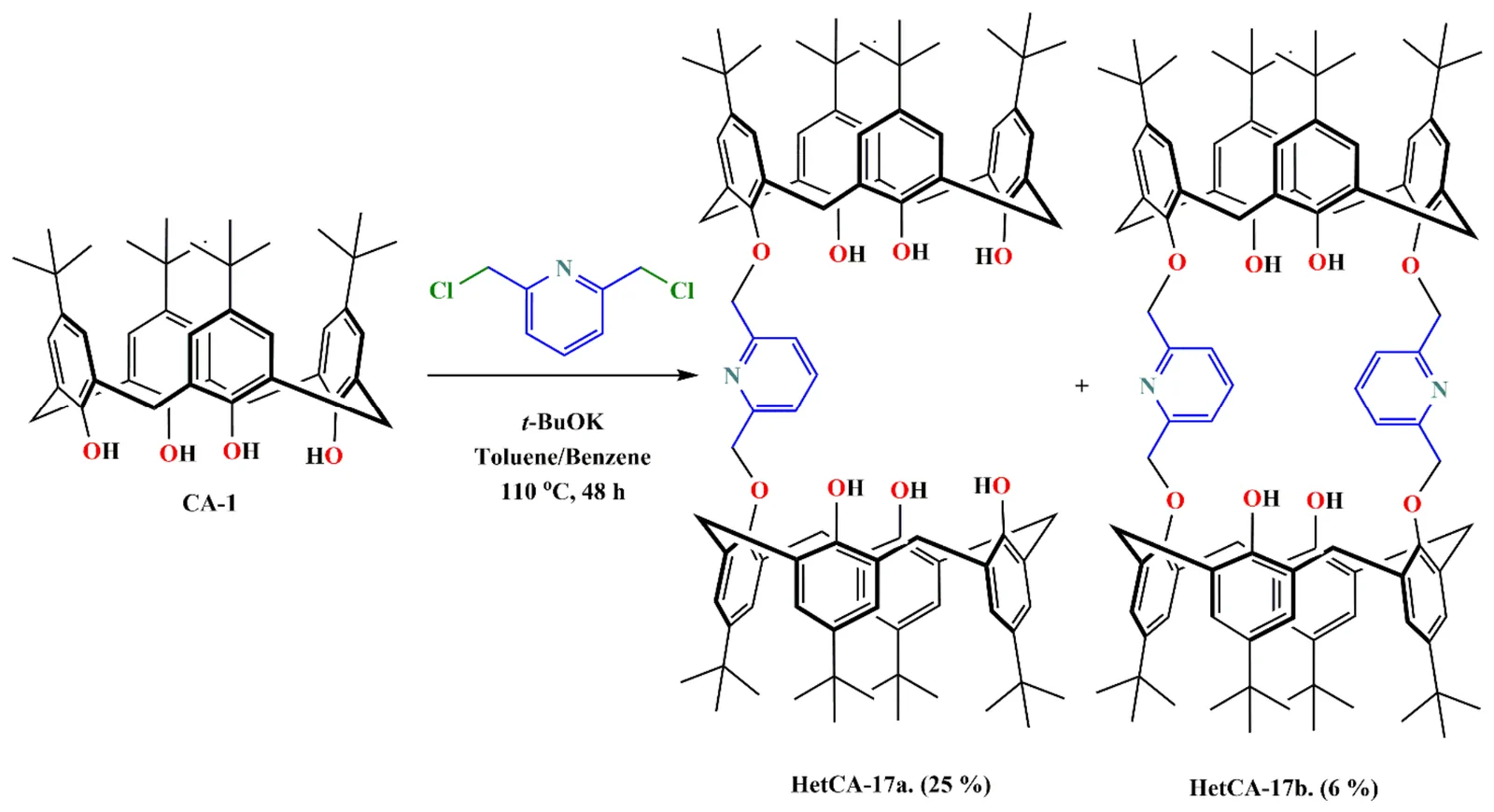
Synthesis of bis-calixarenes **HetCA-17** [193].

As a rule, upper-rim functionalization is more common for classical calix[4]arenes, although analogous modification strategies have also been developed for their sulfur-containing counterparts. In many cases, the lower rim is either left unmodified or functionalized with alkyl or polyether substituents, which not only stabilize the desired macrocyclic conformation but also provide a lipophilic “anchor” for the molecule. In contrast, the introduction of heterocyclic fragments at the upper rim has been reported only rarely. The use of electrophilic agents of type **I** also allowed for the synthesis of amino derivatives of **HetCA-16** at the upper rim with moderate yields (Scheme 8), as demonstrated in studies conducted by Molenveld et al. [191,192]. The use of type **I** electrophilic agents with two reaction centers allows the synthesis of bis-calixarenes, which are particularly appreciated in supramolecular chemistry and sensor chemistry due to the formation of an enlarged cavity, as in the work of Marchand et al. in the case of **HetCA-17** [193] (Scheme 9).

Type **I** heterocyclic electrophilic reagents are the most common method for synthesizing **HetCA** derivatives. Most reactions of this type occur during reflux or moderate heating. This method is well-suited for introducing aromatic heterocyclic structures and can introduce one, two, three, or four substituents into (thia)calix[4]arene. Monosubstitution reactions proceed quickly, within a few hours, while complete substitution of thiacalix[4]arenes requires several days. Polar aprotic solvents, such as acetone, acetonitrile, and DMF, were used. Table S1 summarizes examples of **HetCA** synthesis using type **I** electrophilic reagents [73,138,140,145,152,153,156,159,161,162,163,164,166,168,170,171,177,178,179,181,182,183,184,185,188,189,190,193,194,195,196,197,198,199,200,201,202,203].

##### Substitution via Type II Electrophilic Reagents

**Scheme 10:**
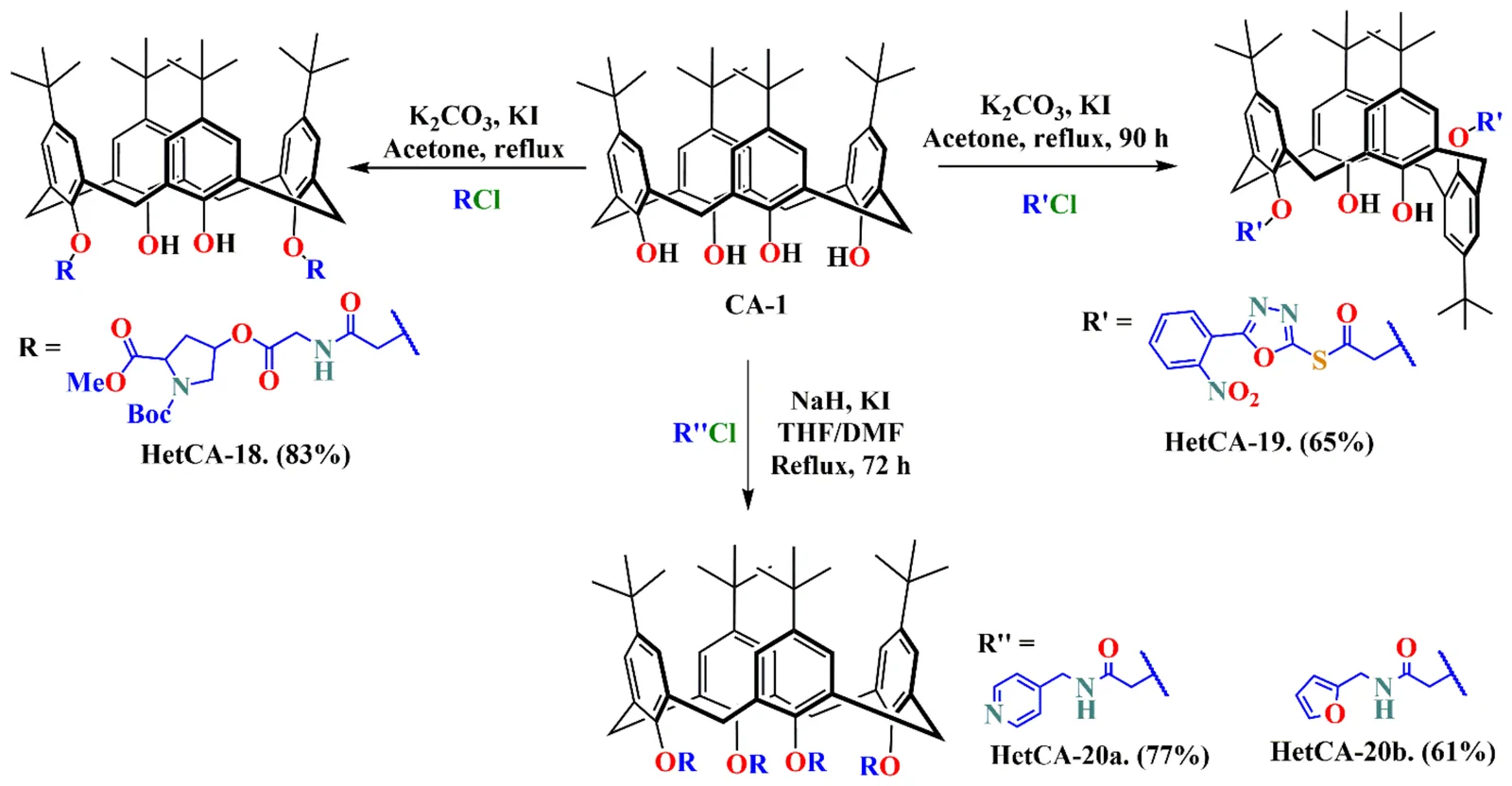
Examples of synthesis of disubstitited **HetCA-18** and **HetCA-19** [134,142] and tetrasubstituted **HetCA-20** using type **II** heterocyclic electrophilic agents [175].

It is frequently convenient to synthesize a type **II** electrophilic agent using chloroacetyl chloride or a similar reagent. For the 1,3-substitution of calix[4]arenes, the Na_2_CO_3_/acetone system is most often used, as it is the simplest and most well-established for S_N_2 reactions. This approach allowed for the standardization of synthetic methods, since the activity of the resulting electrophilic agent does not depend as much on the nature of the heterocycle as in the case of type **I** agents. A wide variety of structures can be used, e.g., Aktas et al. used derivatives of heterocyclic amino acids (such as proline) for the synthesis of **HetCA-18** [142] (Scheme 10). The stereochemistry of the resulting **HetCA**s likely depended primarily on the steric hindrance of the introduced substituent rather than on the nature of the cation and the templating effect it exhibits. For instance, Geselbash and Dilmaghani described the synthesis of compound **HetCA-19** in *partial cone* conformation, which is quite rare in the case of classical calix[4]arenes [134] (Scheme 10). Apparently, the difference in the obtained conformations of **HetCA-18** and **HetCA-19** can be explained by the steric effect of the substituents, which, in the case of **HetCA-19,** prevents the formation of the product in the *cone* conformation. A complete substitution of classical calixarenes requires the use of a strong base, such as sodium hydride. The synthesis of tetrapyridine (**HetCA-20a**) and tetrafuran derivatives (**HetCA-20b**) yielded good results (77 and 61%, correspondingly) in the work of Bayrakcı and Yiğiter [175] (Scheme 10).

Thus, type **II** electrophilic heterocyclic agents are suitable for synthesizing both di- and tetrasubstituted **HetCA**s [134,142,143,150,175,204,205] (Table S2). For disubstituted **HetCA**s, the K_2_CO_3_/KI system in boiling acetone is most often used as a base. Fully tetrasubstituted calix[4]arenes are less common and require a strong base for their synthesis.

##### Substitution via Type III Electrophilic Reagents

**Scheme 11:**
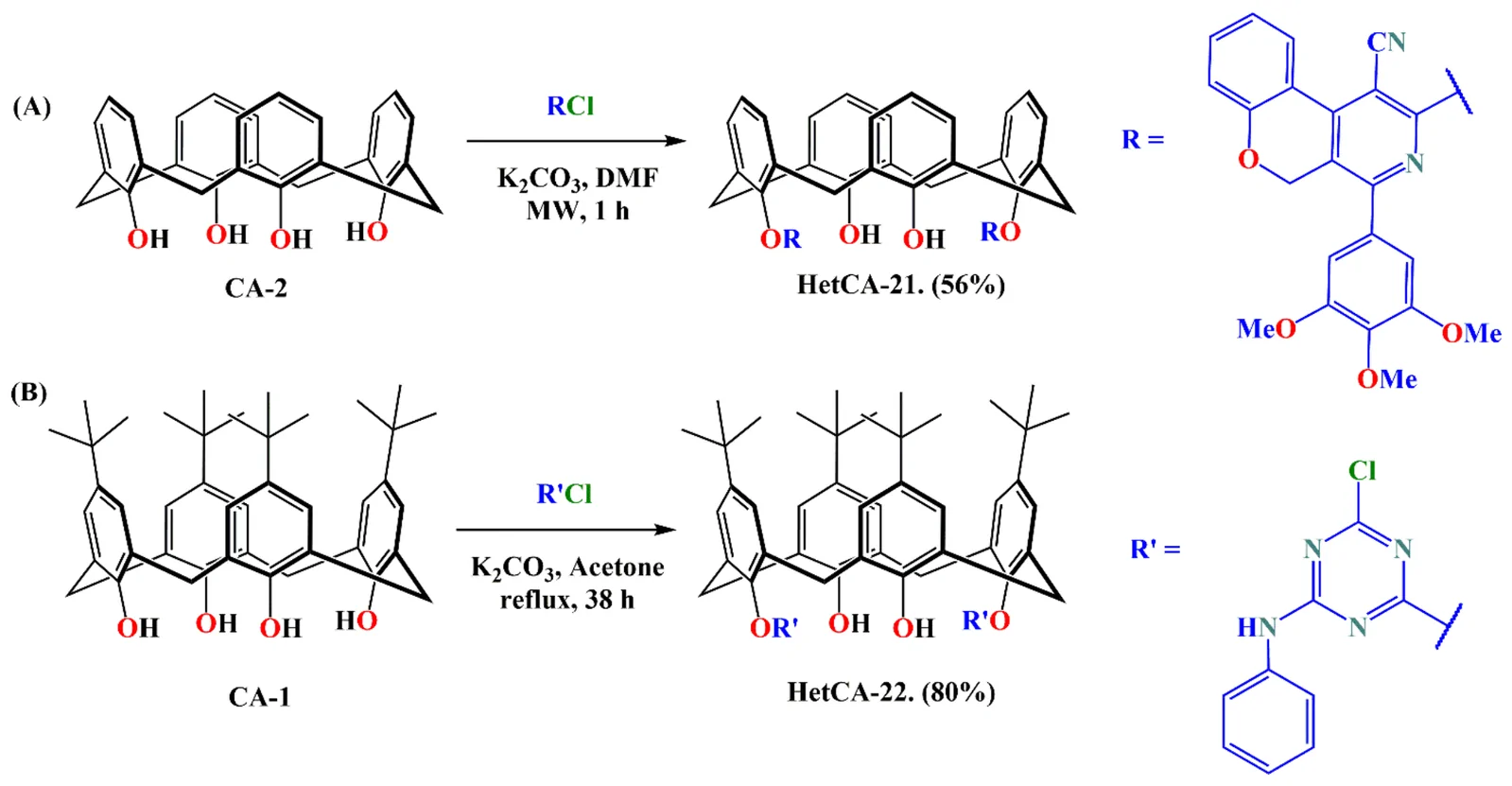
Substitution of *p*-*tert*-butylcalix[4]arenes (**A**) **HetCA-21** and (**B**) **HetCA-22** by type **III** heterocyclic electrophilic agents with electron-withdrawing groups [132,135].

Typically, type **III** electrophilic agents contain a nitrogen-containing aromatic heterocycle, such as pyridine, bipyridine, pyrimidine, 1,3,5-triazine, etc. The heteroatoms in type **III** electrophilic agents promote partial stabilization of the carbocation center directly on the ring. These agents can act as electrophilic agents due to acceptor fragments that stabilize the positive charge in the reaction center. The introduction of two heterocyclic fragments into *p*-*tert*-butylcalix[4]arene usually requires standard heating. It also requires the presence of potassium carbonate as a base. This has been demonstrated by Halay et al. using the example of a 1,3,5-triazine derivative, **HetCA-22** [135] (Scheme 11A). As demonstrated by Ali et al. in the case of introducing a chromenopyridine fragment, bulky heterocycles can be introduced into a macrocyclic structure using microwave radiation. The **HetCA-21** yield was found to be 56% [132] (Scheme 11B).

**Scheme 12:**
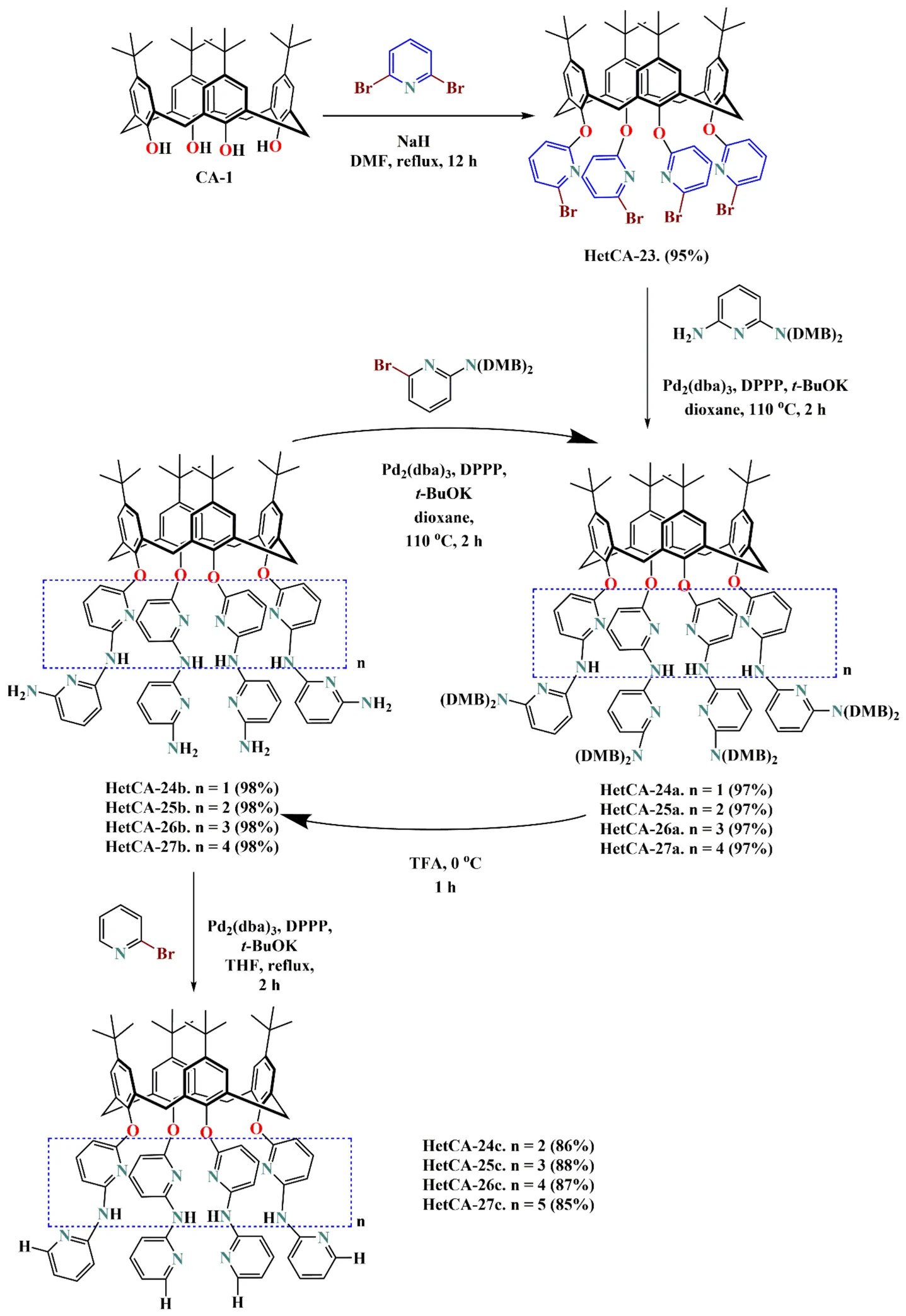
Synthesis of polyaminopyridine derivatives **HetCA-23**–**HetCA-27**. DPPP—1,3-bis(diphenylphosphino)propane, Pd_2_(dba)_3_—tris(dibenzylideneacetone) dipalladium [177].

In particular, the presence of several halogens conjugated with the heteroaromatic system allows cross-coupling reactions to be used for further modification of the primary **HetCA**s obtained. Particularly noteworthy is the recent work by Zeng et al. [177]. A series of **HetCA-23**–**HetCA-27** with a varying number of pyridine fragments in the chain was synthesized. Starting compound **CA-1** was synthesized by heating *p*-*tert*-butylcalix[4]arene with 2,6-dibromopyridine in DMF in the presence of NaH. Compound **HetCA-23** then reacted with 2,6-diaminopyridine in the presence of palladium complexes in a Buchwald–Hartwig-type reaction to produce **HetCA-24a** (Scheme 12). Next, the 2,4-dimethoxybenzyl (DMB) protective group is removed to form macrocycle **HetCA-24b**. There were two ways to modify compound **HetCA-24b**. One method involved modifying it with 2-bromopyridine, which acted as a “plug”, yielding the final compound **HetCA-24c**. The second method involved extending the aminopyridine chain using a process similar to converting **HetCA-24b** to **HetCA-25a**. The protective group is removed from the resulting **HetCA-25b**. The same was true for the next steps. Thus, oligomers of composition **HetCA-24c**–**HetCA-27c** were obtained (Scheme 12).

**Scheme 13:**
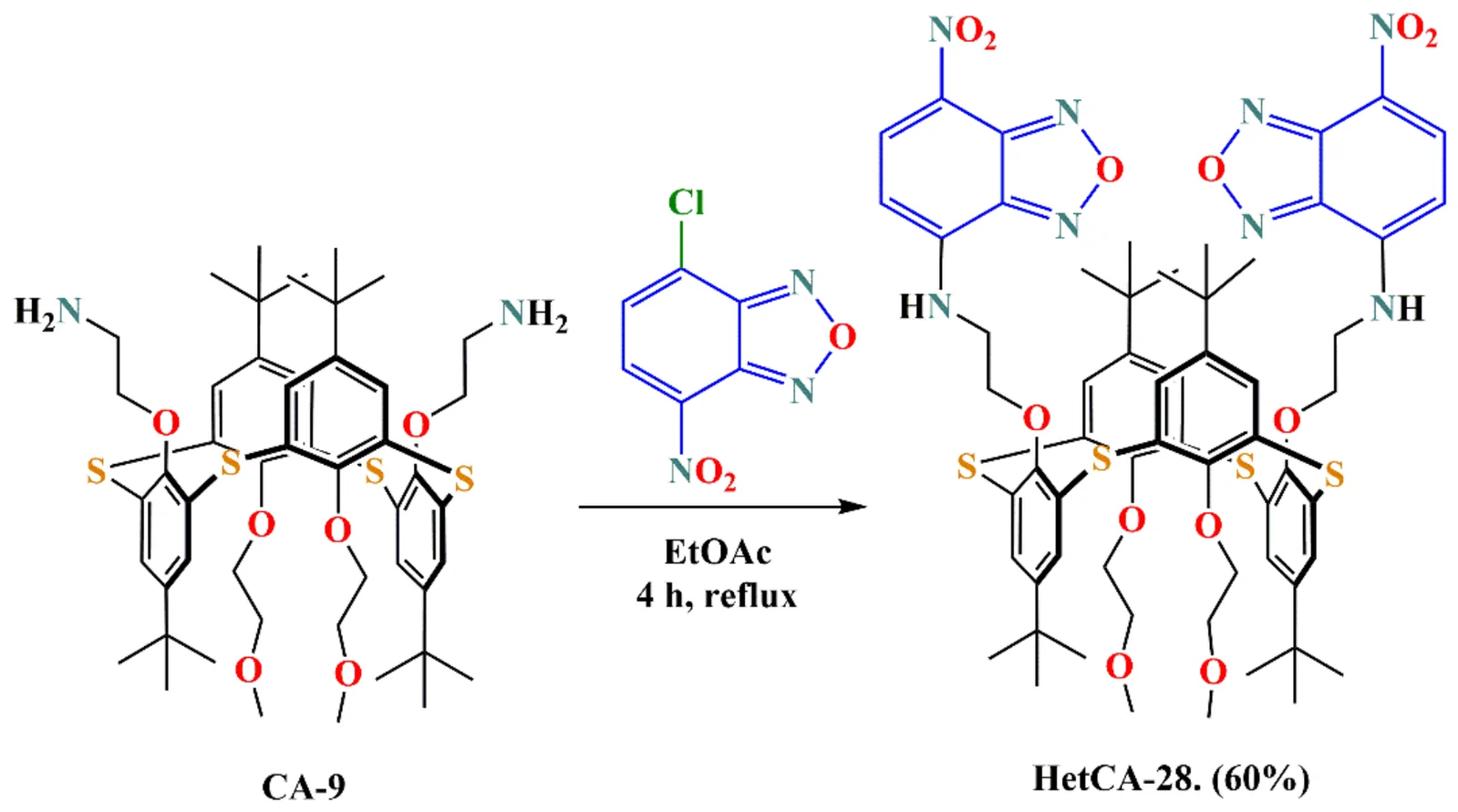
Synthesis of **HetCA-28** modified with two fragments of 7-nitrobenzofurazan [206].

Furthermore, electrophilic agents containing halogens in conjunction with an aromatic heterocycle can be reacted not only directly with phenolic hydroxyls, but also with other functional groups previously introduced into the macrocyclic platform, as demonstrated by Fu et al. [206]. Bis-aminothiacalixarene **CA-9** reacted with 4-chloro-7-nitrobenzofurazan (a chromophoric and fluorophoric building block that becomes highly fluorescent upon reaction with amino groups) without the use of a base (Scheme 13). The **HetCA-28** yield under the conditions of this process was 60%. The *1,3-alternate* conformation enables the construction of a multifunctional receptor in which the nitrobenzofurazan fragments are spatially separated, preventing π–π overlap and minimizing fluorescence self-quenching.

**Scheme 14:**
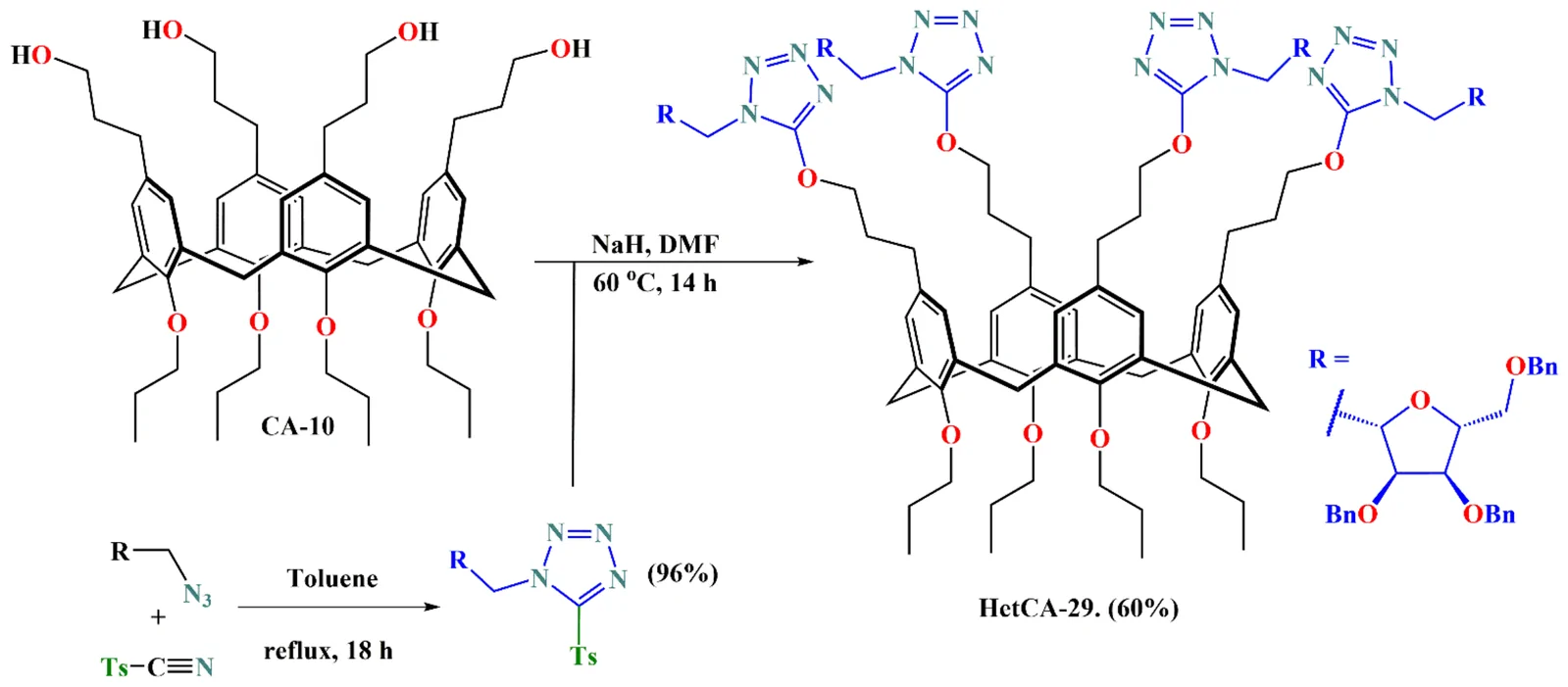
Synthesis of **HetCA-29** (Ts—tosyl) [207].

An interesting approach, representing one of the earliest examples of introducing carbohydrate fragments into calixarenes, involves the use of azide–nitrile cycloaddition instead of the more common azide–alkyne cycloaddition, affording tetrazole derivatives. This approach was implemented by A. Dondoni and A. Marra from the University of Ferrara (Italy) [207]. To carry out this synthesis, the azide derivative of the carbohydrate (*D*-ribose) was reacted with nitrilotosylate, resulting in a conjugate containing a leaving tosylate group. The resulting alkylating agent was used to react with a macrocyclic four-membered alcohol containing hydroxyl groups on the upper rim, yielding **HetCA-29** modified with a *D*-ribose moiety with 60% (Scheme 14). The choice of this structure was dictated by several factors, including the stable *cone* conformation of macrocycle **HetCA-29**, which is ensured by the presence of four propyl substituents at the lower rim, as well as the close spatial arrangement of the carbohydrate fragments. The choice of the upper rim of the macrocycle for modification is determined by the creation of a polytopic ligand in which all functional fragments of the carbohydrate are directed outward and do not overlap each other.

**Scheme 15:**
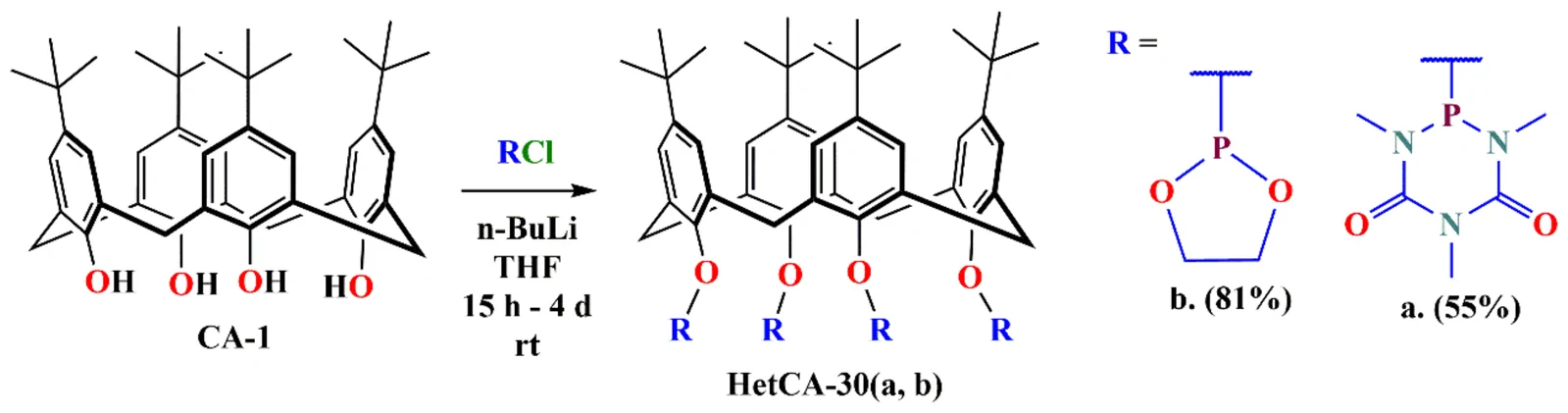
Synthesis of **HetCA-30** [208].

Regarding the structure of heterocyclic fragments, aromatic nitrogen-containing cycles, e.g., pyridine, pyrazine, quinoline, imidazole, benzimidazole, and phthalimide, are most commonly represented. Quite rare, but nevertheless interesting, are cases of the introduction of cyclic structures containing rarer heteroatoms, such as sulfur atoms (benzothiazole, thiophene, and tetrathiafulvalene) and phosphorus atoms. Research teams from Braunschweig and Rostock (Germany) synthesized phosphorus-containing calixarenes **HetCA-30** using butyl lithium as a base in THF at room temperature, with yields of 55 to 81%, correspondingly [208] (Scheme 15).

Type **III** heterocyclic electrophilic agents are ideal for introducing heterocycles with a variety of structures, from simple to fused cyclic systems, into calixarene structures. These approaches can also be used to create heterocyclic chains of different lengths for the synthesis of coordination polymers. The conditions of **HetCA**s synthesis via type **III** electrophilic reagents are shown in Table S3 [132,135,206,207,208,209].

##### Substitution via Type **IV** Electrophilic Reagents

**Scheme 16:**
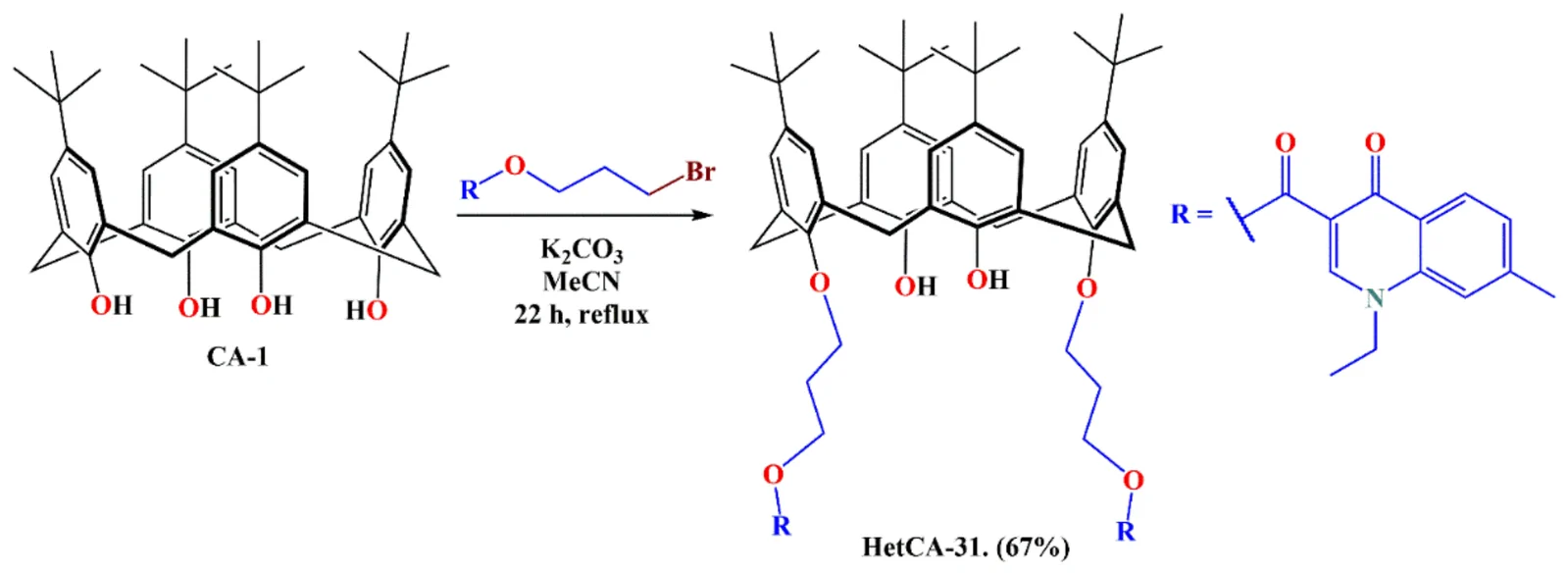
Synthesis of quinozolinone derivative **HetCA-31** [133].

**Scheme 17:**
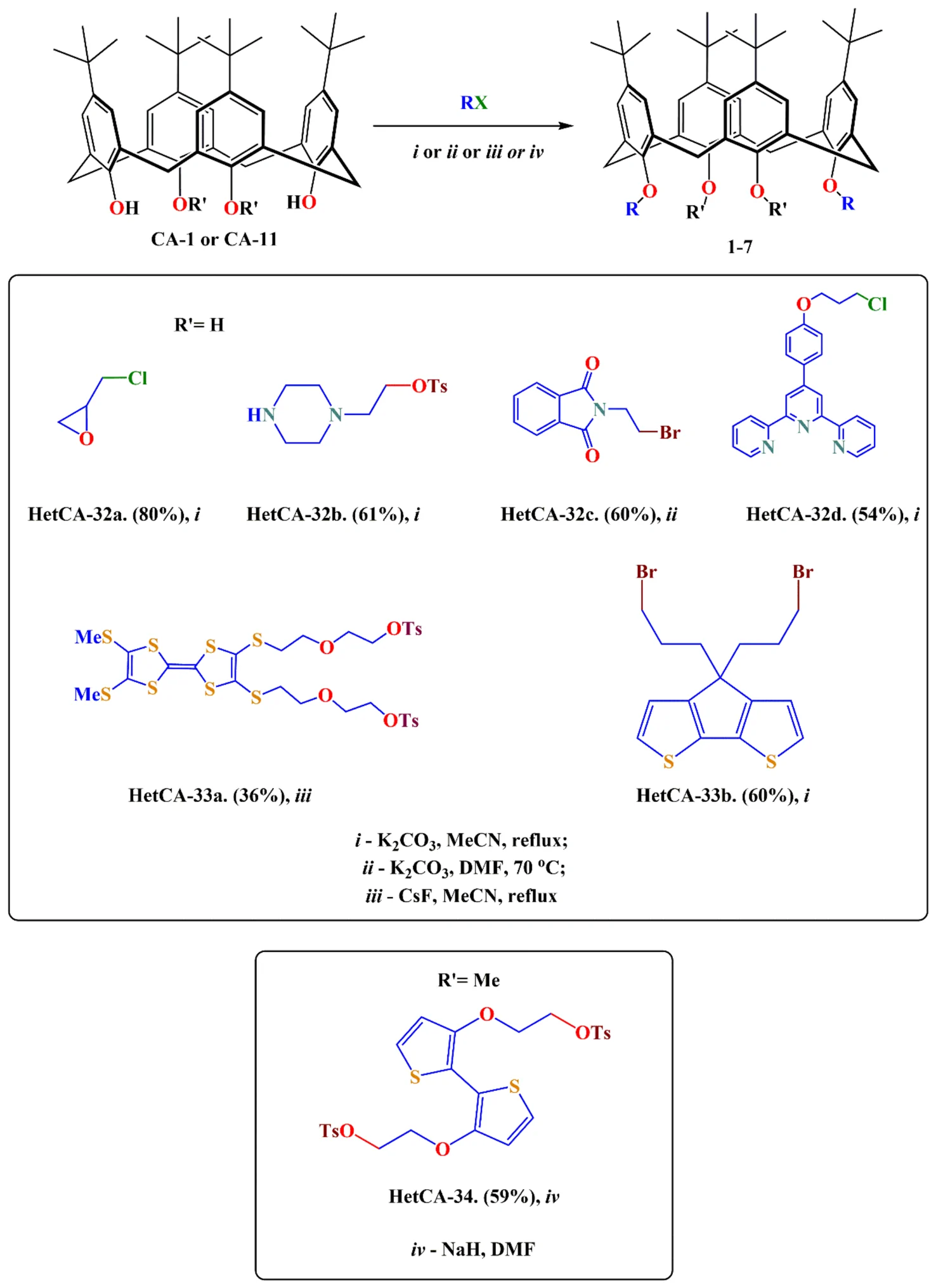
Synthesis of bis-calixarenes **HetCA-32**–**HetCA-34** using heterocyclic type **IV** electrophilic agents [139,141,144,146,147,210,211].

In addition to works on heterocyclic electrophilic agents whose reaction center is stabilized by conjugation, there is considerable information on the use of less active type **IV** electrophilic reagents. These reagents do not have a stabilized electrophilic center [133]. Nevertheless, this approach has proven to be one of the most productive Williamson-type reactions. Using strong bases, polar aprotic solvents, and good leaving groups, it is possible to obtain **HetCA**s without any particular problems, even in the case of electrophilic agents of type **IV**. The conditions for such reactions are quite similar to those required for more active agents, indicating a wide range of possibilities in the selection of electrophilic reagent structures, as in the work of Sautrey et al. for the synthesis of quinazolinone derivative **HetCA-31** (Scheme 16) [133]. The use of type **IV** monomeric heterocyclic derivatives containing two electrophilic centers allowed the production of bis-substitution products, including bis-calixarenes **HetCA-32**–**HetCA-34** as in the works (Scheme 17) [141,193,210,211].

**Scheme 18:**
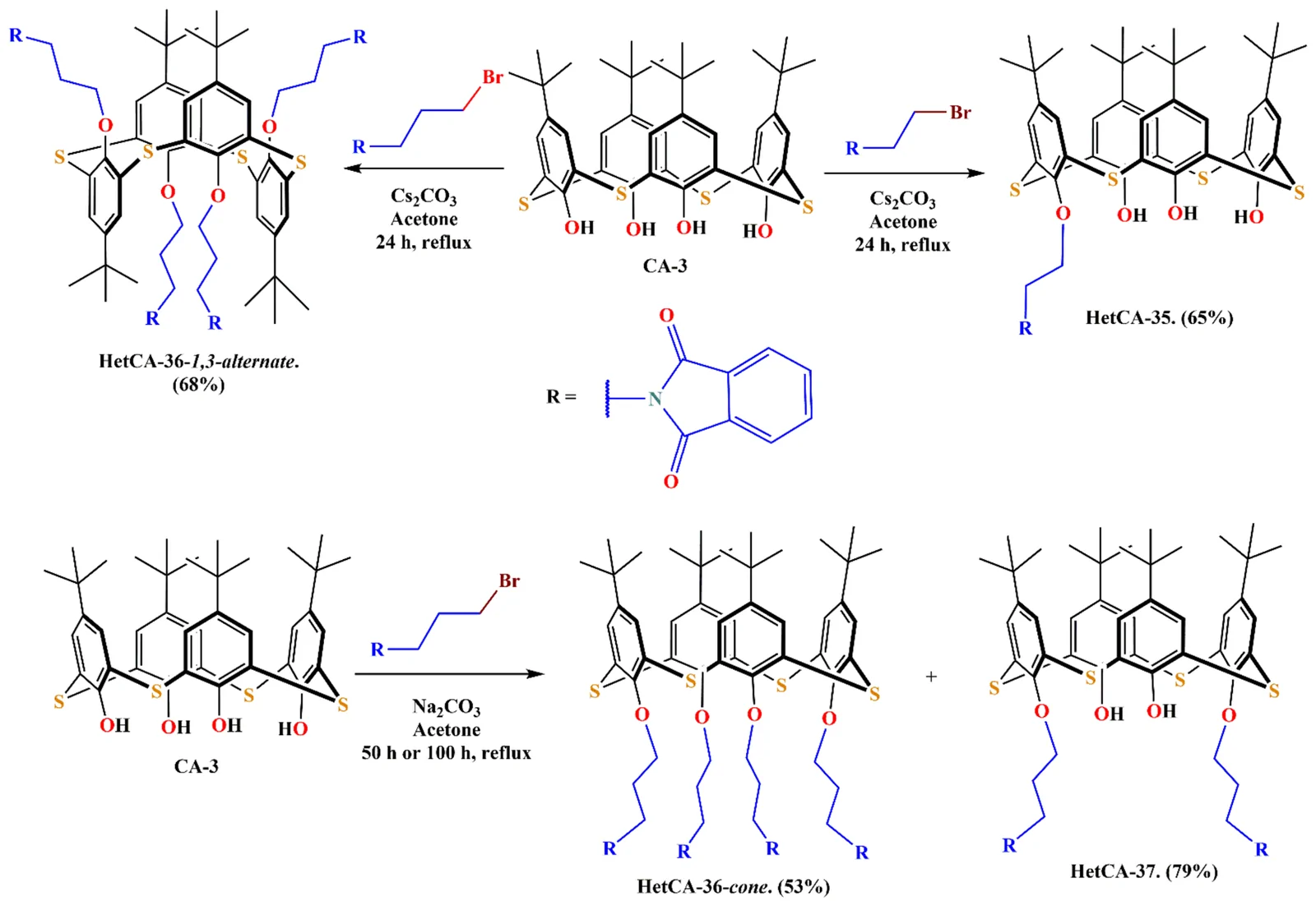
Synthesis of mono-, 1,3- and fully substituted phthalimide derivatives of *p*-*tert*-butylthiacalix[4]arene **HetCA-36**–**HetCA-37** [212,213].

As mentioned above, thiacalix[4]arene is more likely to undergo complete substitution of all protons of phenolic hydroxyl groups at the lower rim in comparison to classical calix[4]arene. However, there are exceptions to this pattern. For example, if a sterically hindered fragment linked to the macrocyclic platform by a short linker is introduced by an electrophilic substitution reaction, it is possible to introduce fewer than four substituents. As demonstrated in the works of Prof. Dr. I. Stoikov’s scientific group from Kazan Federal University (Russia) [212,213], the phthalimide derivative **HetCA-35** (type **III**) containing an ethylene linker was capable of substituting only one hydrogen atom of the macrocyclic platform regardless of the synthesis time, base strength, and reagent ratio (Scheme 18). Increasing the linker by just one methylene fragment allowed the introduction of two (**HetCA-37**) or four phthalimide fragments (depending on the base and the ratio of reagents), while varying the conformation of the resulting **HetCA-36** (*cone* or *1,3-alternate*) [213].

Thus, synthesis of **HetCA**s using electrophilic agents of type **IV** requires more stringent conditions. All reactions proceed upon heating and often require the presence of potassium iodide (Finkelstein reaction). The detailed conditions for these reactions are presented in Table S4 [74,133,139,141,144,146,147,210,211,212,213,214,215,216].

##### Substitution via Type V Electrophilic Reagents

**Scheme 19:**
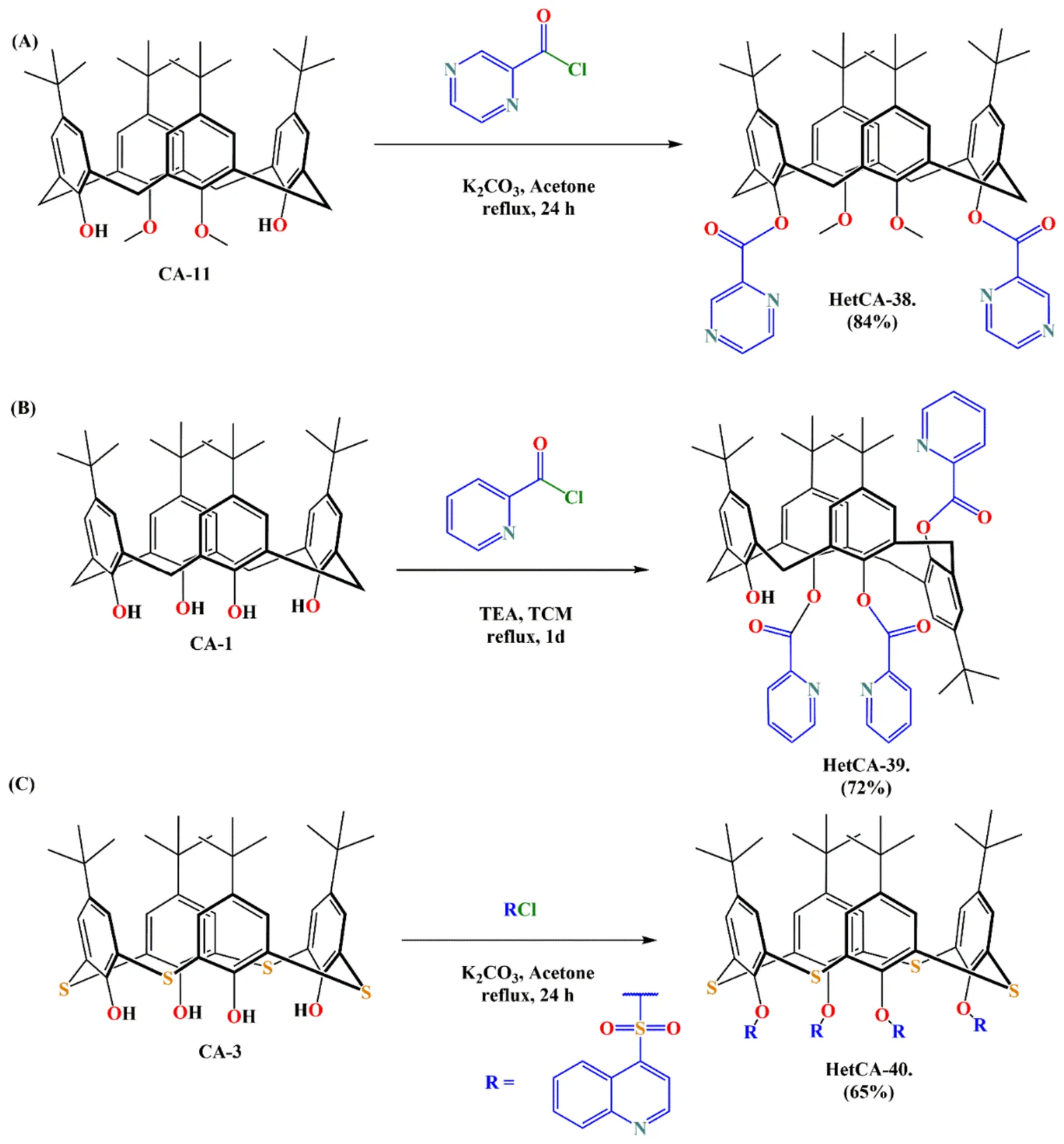
Synthesis of **HetCA-38**–**HetCA-40** with (**A**) 1,4-diazine [217], (**B**) picolyl [218], (**C**) quinoline-8-sulfonate [174] fragments using heterocyclic type **V** electrophilic agents.

Type **V** heterocyclic acylating agents are relatively rare, since this method requires the prior preparation of the corresponding carboxylic acids, which can often limit the electrophilic agent as a whole. As a rule, such agents include relatively simple heterocyclic structures. Alkali metal carbonates or triethylamine are used as the bases. As described for electrophilic agents of types **I**–**V**, acylation is easier than alkylation. Therefore, potassium carbonate is sufficient for complete substitution, as demonstrated in Güngör’s work, in which two 1,4-diazine fragments are introduced to obtain **HetCA-38** (Scheme 19A) [217]. Interestingly, Lee et al. report that trisubstituted product **HetCA-39** (*partial cone*) was obtained upon attempting the exhaustive acylation of *p-tert-*butylcalix[4]arene with picolyl chloride [218] (Scheme 19B). The increase in cavity size during the change from classical *p*-*tert*-butylcalix[4]arene to its sulfur analog likely allowed potassium carbonate to produce a tetrasubstituted **HetCA-40** in the form of a mixture of conformations (Scheme 19C) [174].

**Scheme 20:**
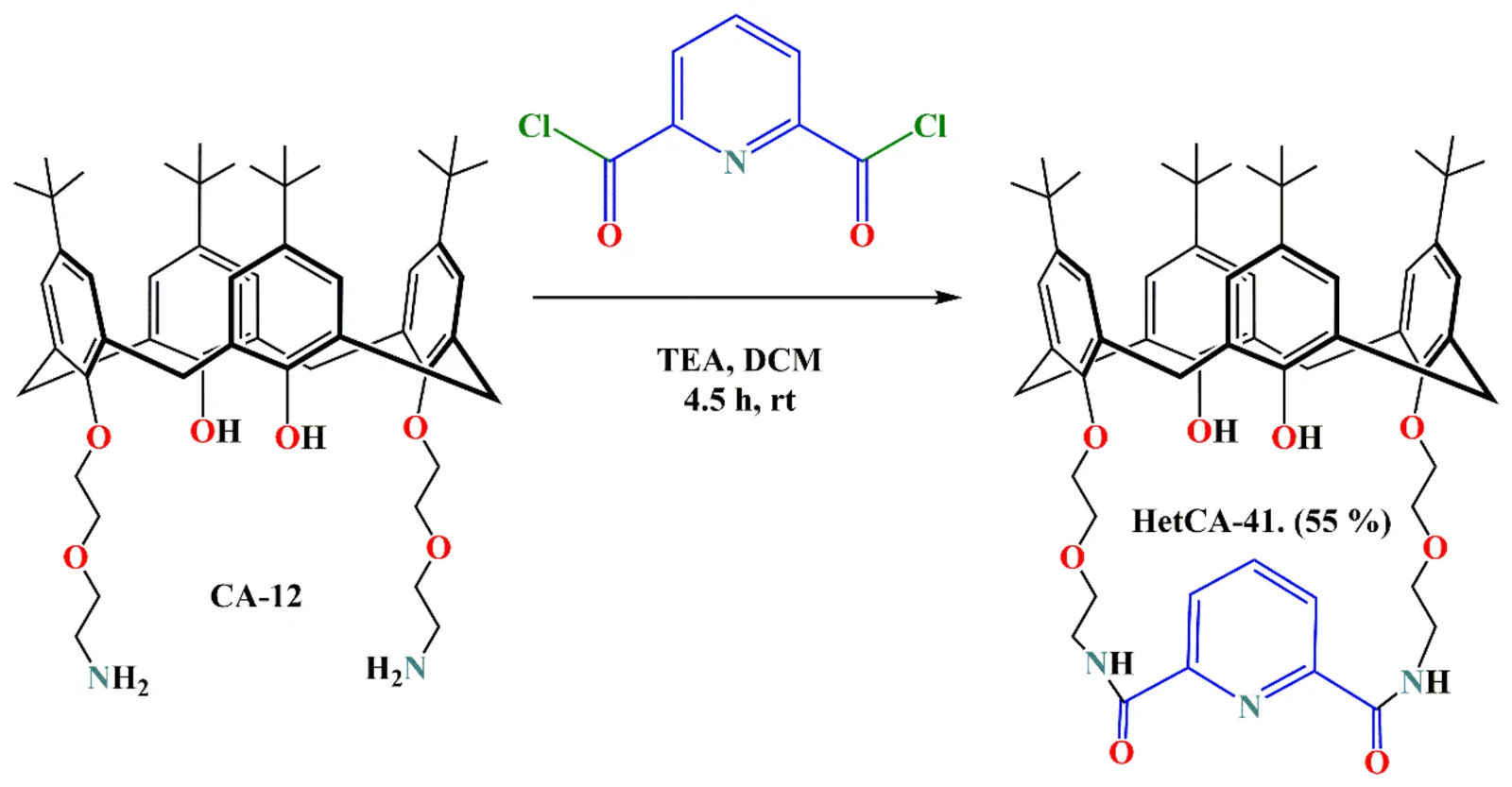
Synthesis of **HetCA-41** with aza-crown fragment using heterocyclic type **V** electrophilic agents [219].

Not only the initial *p*-*tert*-butylcalix[4]arene **CA-1**, but also its derivatives modified with various functional groups can be used to obtain **HetCA**s. McConnell et al. report the synthesis of a macrocyclic aza-crown sensor **HetCA-41** from pyridine-1,3-dicarboxylic acid chlorohydride and calix[4]arene **CA-12** containing primary amino groups (Scheme 20) [219]. The creation of crown structures on calixarene is one of the main methods for creating ligands for hard Lewis acids, in particular for alkali metal cations and ammonium ions.

**Scheme 21:**
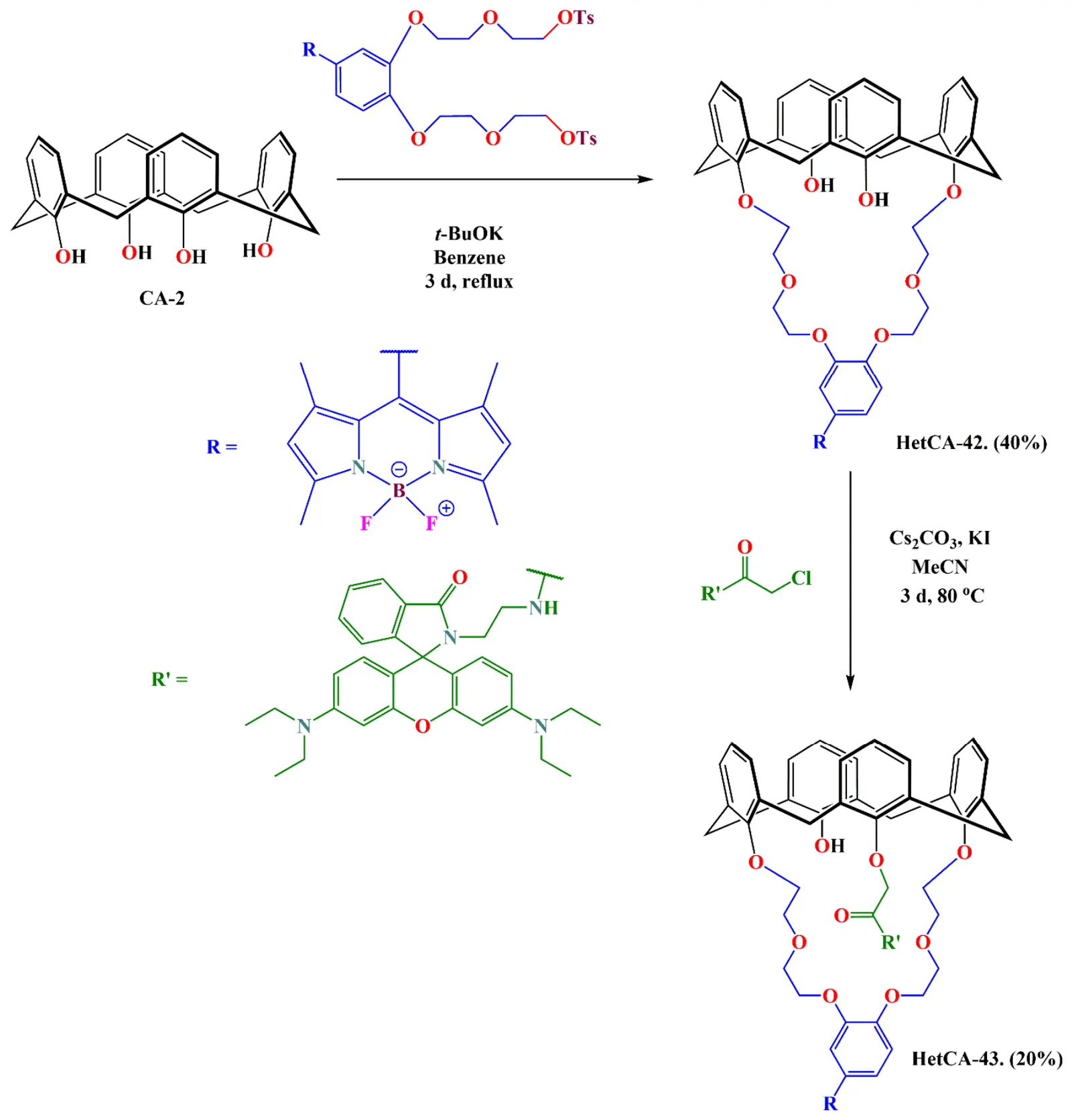
Two-step synthesis of fluorescent ligand **HetCA-43** using heterocyclic type **IV** and **II** electrophilic agents [220].

Of particular interest are calix[4]arenes containing several different heterocyclic fragments introduced by various synthetic schemes. Yuan et al. synthesized the macrocyclic sensor **HetCA-43** by intermediately synthesizing **HetCA-42**, which is selective for mercury(II) and barium(II) cations. by combining heterocyclic electrophilic agents of types **IV** (rhodamine fragment) and **II** (introduction of a BODIPY fragment) (Scheme 21) [220].

In addition to aromatic systems, saturated cyclic amines, such as pyrrolidine and piperidine derivatives, can also be used for **HetCA**s synthesis. Calixarene derivatives with oxygen- (1,3,4- oxadiazole [134], oxirane [144,221], benzo-15-crown-5 [150]) and sulfur-containing heterocycles (thiazole [140], TTF [141]) are also described.

Thus, this type of reaction is one of the rarest methods of **HetCA** synthesis. Heterocyclic acylating agents typically have relatively simple structures due to the difficulty of obtaining halogen anhydrides. Table S5 shows the reaction conditions in detail [137,174,217,218,219,222,223].

The most common approach for synthesizing **HetCA**s at present is the modification of calix[4]arenes at the lower rim with heterocyclic electrophilic agents. This method has several advantages, including the ability to produce a wide range of heterocyclic structures, such as pyridine, piperidine, thiazole and tetrathiafulvalene. However, the method is not without certain limitations. The main limitations are associated with the high reactivity of alkylating agents. This reactivity significantly complicates the use of this approach in cases where the reaction involves compounds containing groups sensitive to alkylation. Examples of these groups include hydroxy, amino, and mercapto fragments.

#### 3.1.2. The Williamson-Type Nucleophilic Substitution Reactions. Part II: Interaction of a Macrocyclic Electrophilic Agent with Heterocyclic Substrates

**Scheme 22:**
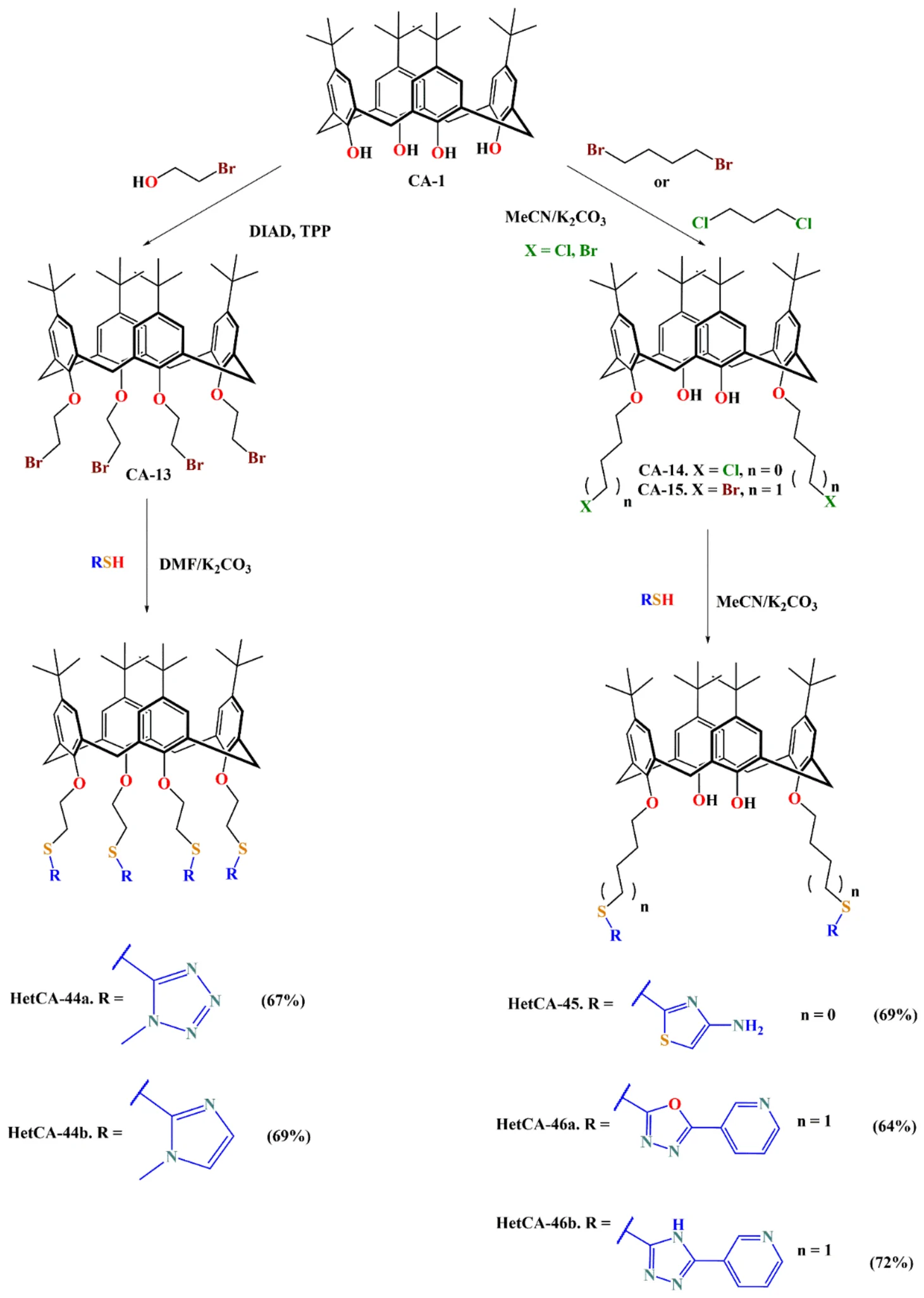
Formation of heterocyclic derivatives of *p*-*tert*-butylcalix[4]arenes, **HetCA-44**–**HetCA-46,** from macrocyclic electrophilic agents and heterocyclic thiols [224,225,226,227].

Another synthetic approach to **HetCA**s involves transferring the electrophilic center to calixarene. This approach is less common than the previous one (Section 3.1.1) and is used to obtain esters and alkylation of thiols and primary amines. An intermediate electrophilic agent is often obtained by the interaction of either halogenated alcohols, which are introduced into the macrocyclic platform with the Mitsunobu reaction (diethyl azodicarboxylate (DEAD), di-*iso*-propyl azodicarboxylate (DIAD) or their analogs, triphenylphosphine (TPP)) to obtain **HetCA-44** [224,225] or a terminal dihaloalkane of the desired length to synthesize **HetCA-45**–**HetCA-46** [226,227]. Alkali metal carbonates are often used as a base. Acetonitrile or DMF are most commonly used as solvents (Scheme 22).

**Scheme 23:**
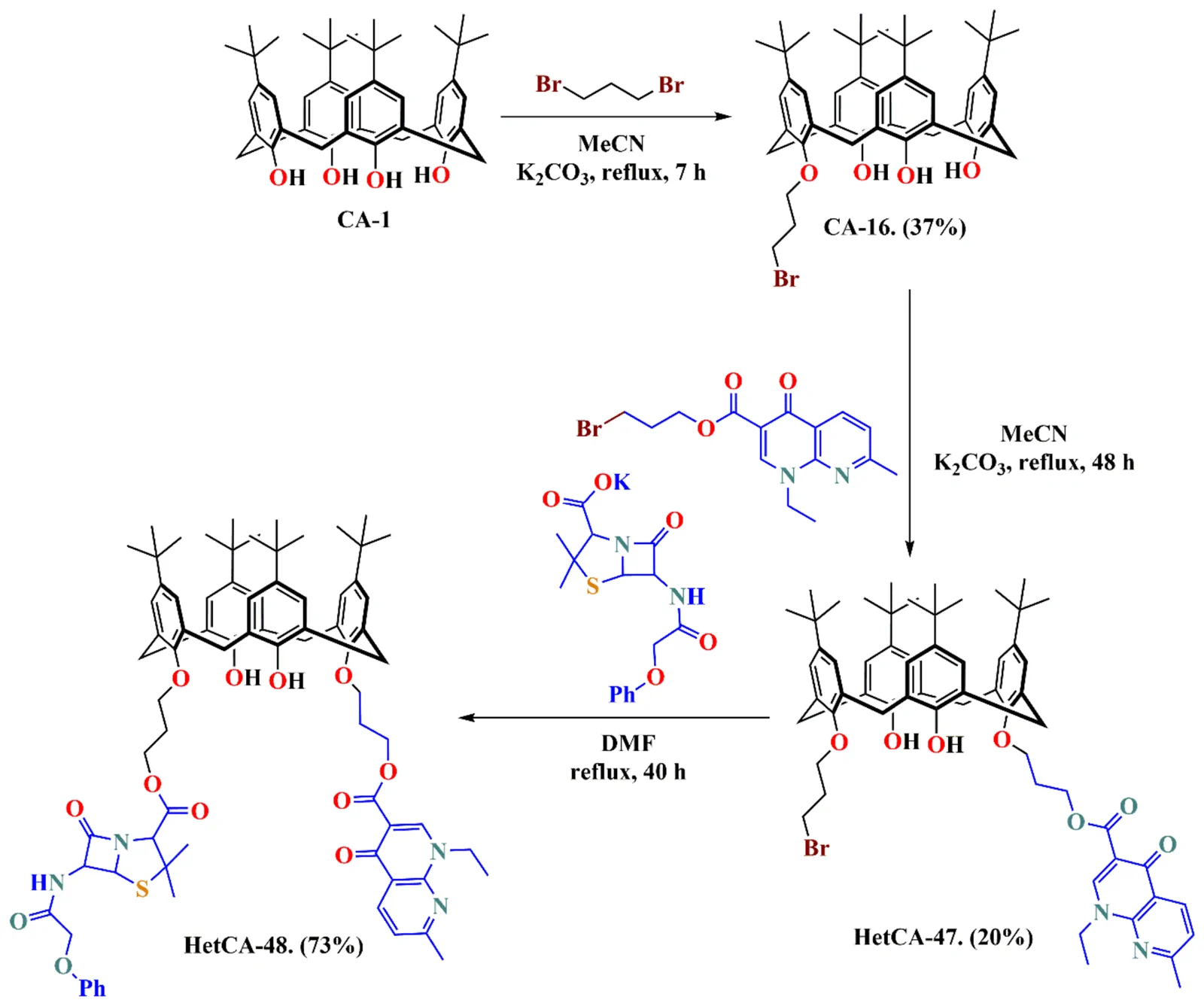
Synthesis of **HetCA-48** modified with fragments of nalidixic acid and penicillin V [74].

This approach usually produces macrocyclic 1,3-disubstituted products [228,229,230,231], although there are cases of complete substitution and occasionally monosubstitution [188,232]. By combining two approaches, the group of Prof. Regnouf-de-Vains synthesized **HetCA-48** modified by two pharmacophore fragments at once—nalidixic acid (a derivative of naphthyridine) and penicillin V (Scheme 23) [74].

**Scheme 24:**
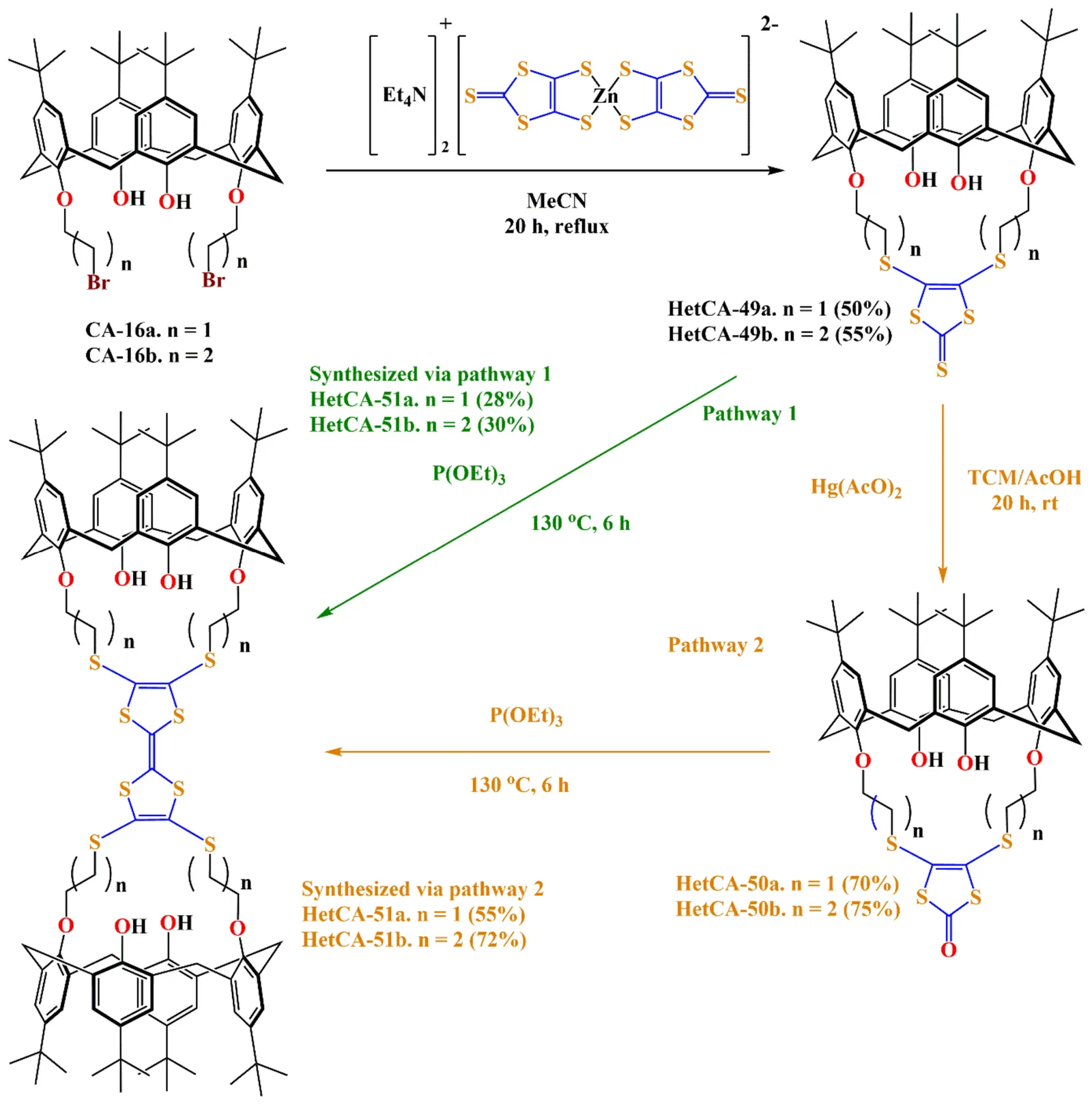
Synthesis of TTF-derivatives of bis-calixarene **HetCA-51** [233].

The method of obtaining bis-calixarenes **HetCA-51**, which is formed during the reductive coupling of a 1,3-dithiol-2-one moiety with the formation of a tetrathiafulvalene derivative, is of considerable interest [233,234]. The first stage of the reaction of macrocyclic amines with a zinc(II) complex of dithiol was carried out in boiling acetonitrile for 20 h with a yield of 50–55%. There were two different ways to obtain the target bis-calixarenes **HetCA-51**. In the first method (Scheme 24, pathway 1), the researchers started with thiol derivatives **HetCA-49** and converted them directly into the target products **HetCA-51**. In the second case, the researchers first converted the compound **HetCA-49** into keto derivatives of the dithione **HetCA-50**. Then, they converted keto derivatives **HetCA-50** into target macrocycles **HetCA-51** (Scheme 24, pathway 2). Pathway 2 had higher yields than Pathway 1 (55–72% vs. 28–30%, correspondingly) [233].

**Scheme 25:**
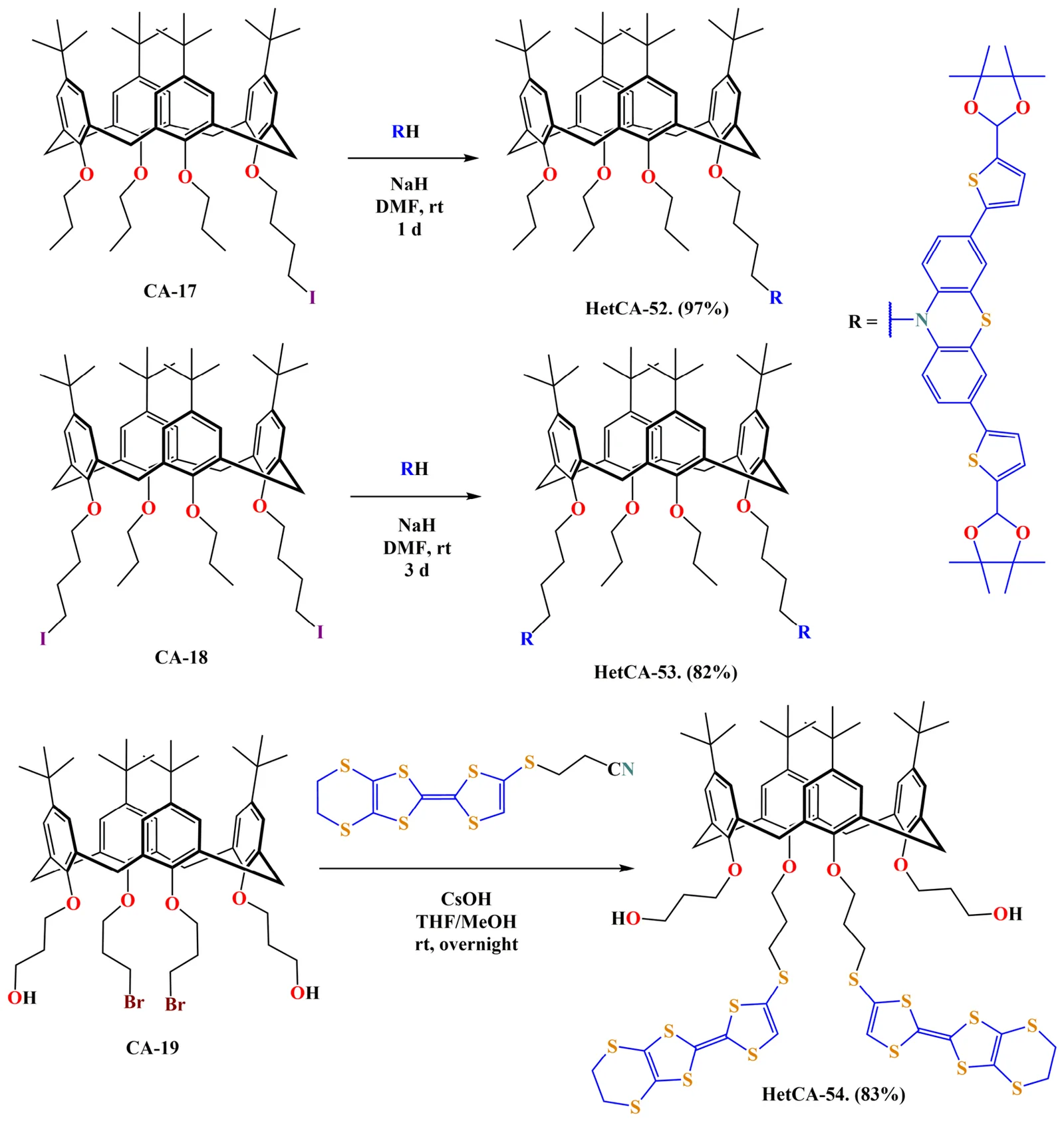
Synthesis of **HetCA-52**–**HetCA-54** [235,236].

One of the best examples of the quantitative yield of **HetCA**s can be seen in the work of Decavoli et al. [235]. Using sodium hydride in DMF at room temperature for one day allowed for the synthesis of macrocyclic phenothiazine and thiophene derivatives **HetCA-52**–**HetCA-53** with yields of up to 97% (Scheme 25). Selecting good leaving groups can increase the yield of target **HetCA**s. For example, TTF is often introduced by cleaving the cyanoethyl group with fairly good results, as in the work of Sun et al. [236], in which **HetCA-54** is synthesized in 83% yield.

**Scheme 26:**
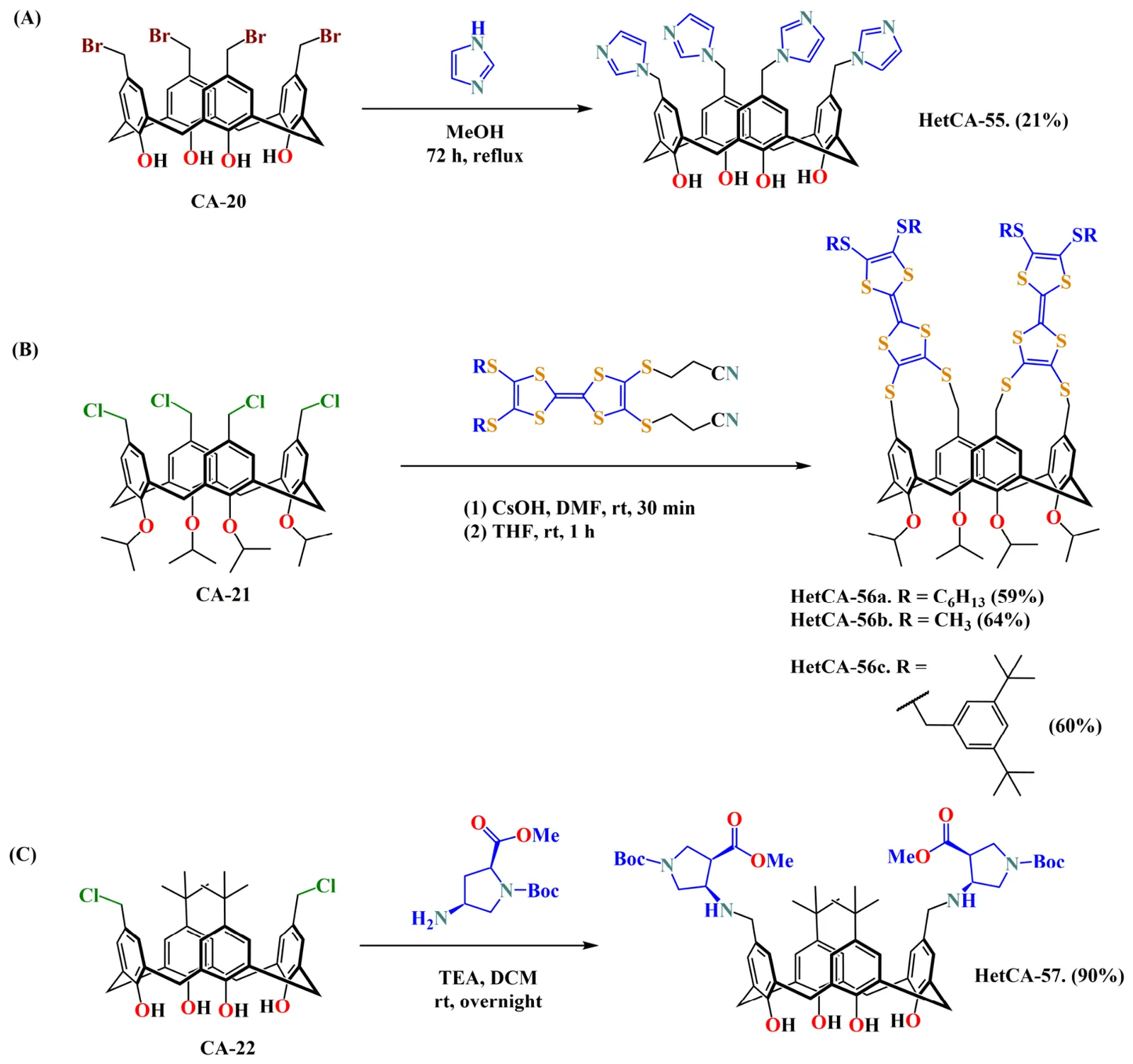
Modification of calix[4]arenes **CA-20**–**CA-22** with heterocyclic fragments on the upper rim. (**A**) Synthesis of tetrakis-imidazole derivative **HetCA-55**; (**B**) functionalization of **CA-21** with TTF moieties to synthesize macrocycles **HetCA-56**; (**C**) synthesis of macrocyclic *L*-proline derivative **HetCA-57** [238,239,240].

In addition to standard modification practices on the lower rim, relatively few examples of introducing heterocycles on the upper rim are also described [237]. For example, a tetraderivative of imidazole **HetCA-55** was obtained (Scheme 26A) [238]. The yield in this reaction was quite low (21%), which can be explained by the authors’ failure to use any bases other than imidazole itself. The combination of strong bases and good leaving groups allowed for the introduction of significantly more complex substrates (such as two TTF fragments) with moderate yields (**HetCA-56**, 59–64%) (Scheme 26B) [239]. This approach can also be successfully applied for the introduction of chiral heterocyclic nucleophilic agents, provided that the asymmetric center is not affected, yielding **HetCA-57** in high yield (90%) [240] (Scheme 26C).

**Scheme 27:**
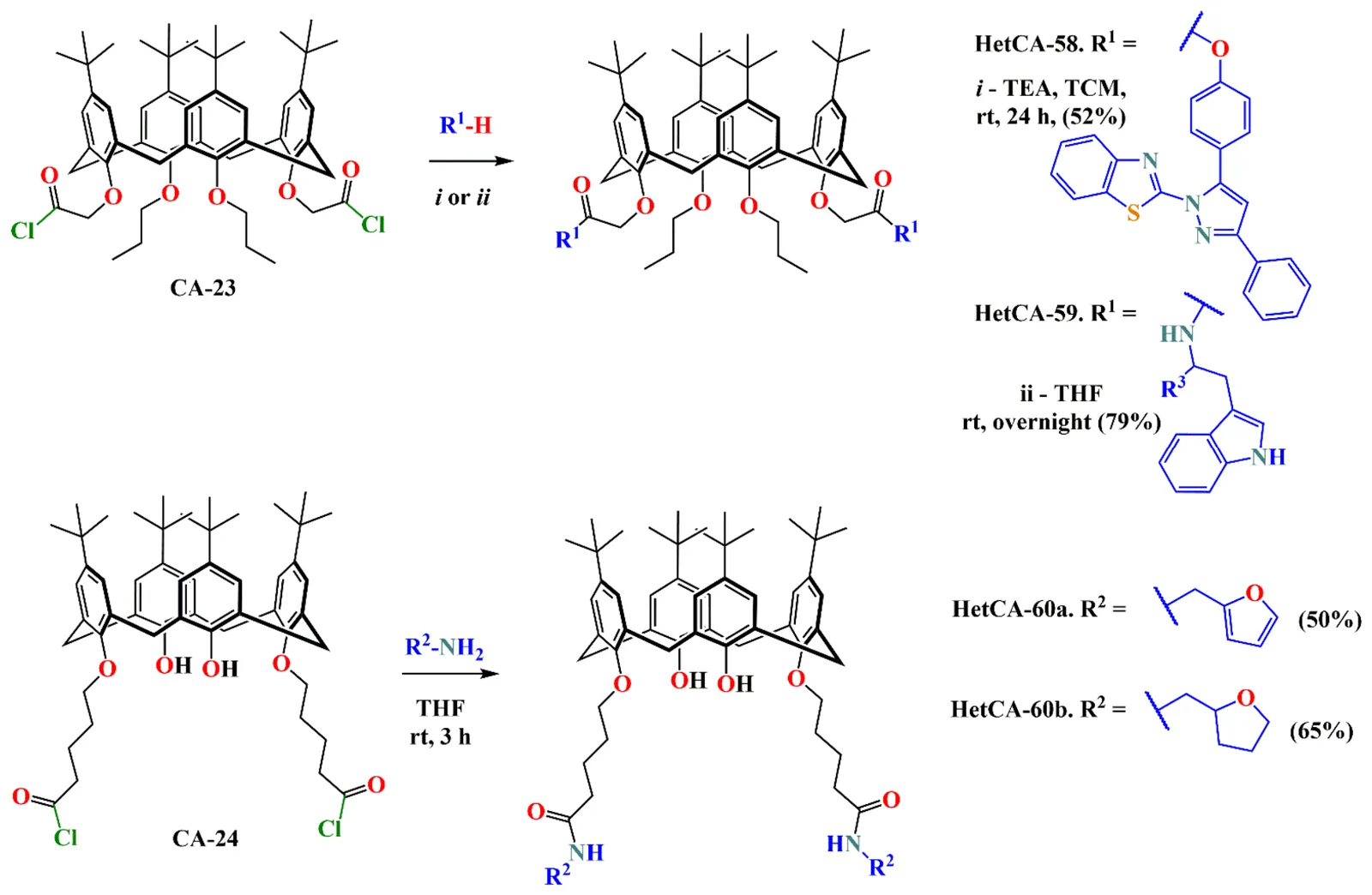
Synthesis of **HetCA-58**–**HetCA-60** from chloranhydride macrocyclic derivatives **CA-23** and **CA-24** [241,242,243].

Chloranhydrides are often used to synthesize **HetCA**s with ester and amide moieties. The most commonly used solvents are chloroform (trichloromethane, TCM), dichloromethane (DCM) and tetrahydrofuran (THF). As a rule, reagents are mixed while being cooled to zero degrees, followed by gradual heating to room temperature. This method can be used to introduce sterically busy heterocyclic substituents, as in the work of Peng et al., in which macrocycle **HetCA-58** was synthesized with 52% yield [241] or **HetCA-59** (79%) by Bolshchikov et al. [242] (Scheme 27). The acylation reaction also proceeds well in the presence of free OH fragments of calixarenes. For example, Özkan et al. reported the synthesis of (tetrahydro)furan derivatives **HetCA-60** from a macrocycle **CA-24** and the corresponding amines [243] (Scheme 27).

**Scheme 28:**
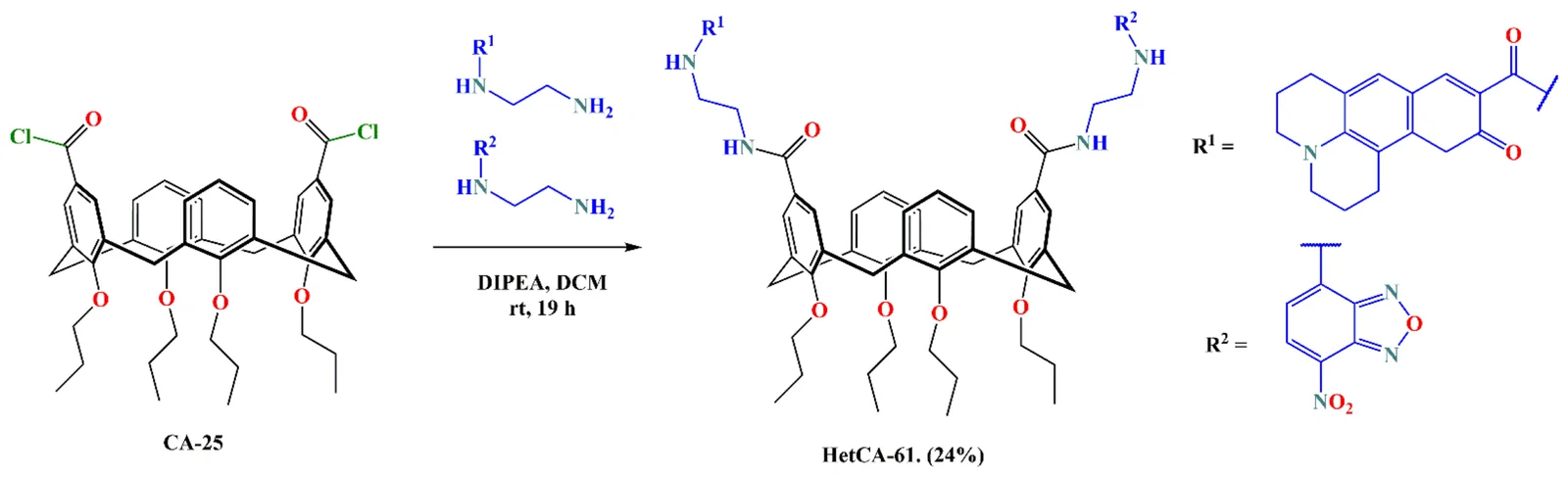
One-pot synthesis of **HetCA-61** modified with two different heterocycles [244].

Modifying calixarenes with halocarbonyl groups at the upper rim opens up prospects for introducing various heterocyclic fragments. These moieties give the macrocyclic platform special properties, particularly the ability to fluoresce at different temperatures. A one-pot introduction of various heterocyclic fragments imparts special properties to the macrocyclic platform. In particular, these fragments impart the property of temperature-dependent fluorescence. This allows the resulting calixarene to be used as thermometers [244]. The reaction proceeded for 19 h at room temperature with a yield of 24% of the target product **HetCA-61** (Scheme 28).

**Scheme 29:**
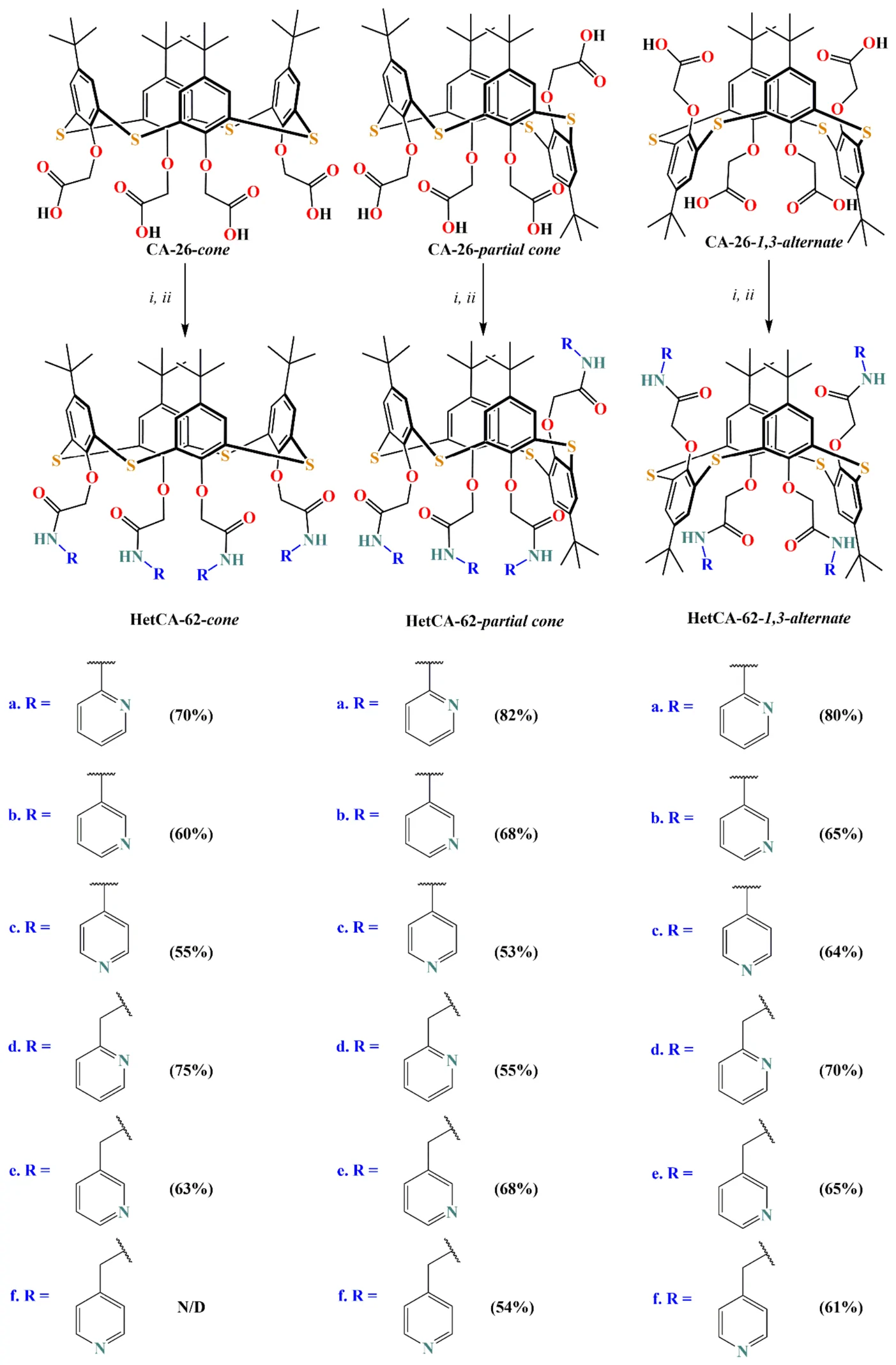
Synthesis of pyridine derivatives **HetCA-62** [245]. Conditions: (i) SOCl_2_, DCM, (ii) DCM, rt, overnight.

The acylating agents do not necessarily need to be isolated from the reaction mixture. Thus, Stoikov et al. converted the tetraacids **CA-26** into an acid chloride without isolation, and then the resulting acid chloride reacted with aminopyridines and picolylamines (Scheme 29). Resulting macrocycles **HetCA-62** were obtained in 54–82% yields [245].

**Scheme 30:**
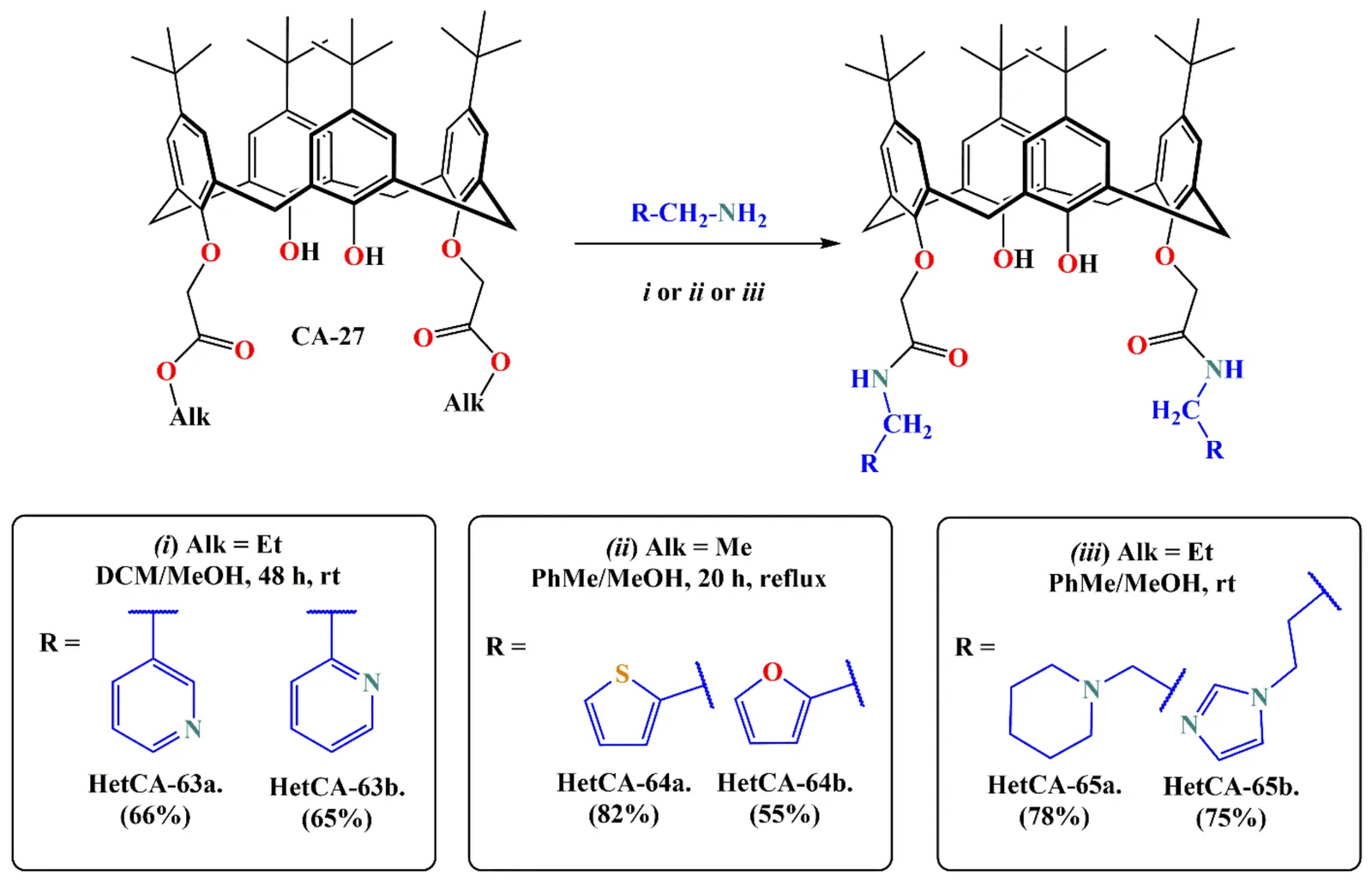
Synthesis of 1,3-disubstituted **HetCA-63**–**HetCA-65** via aminolysis [246,247,248].

Methods for introducing heterocyclic fragments related to the aminolysis reaction have also been developed. Aminolysis of esters, i.e., the interaction of esters with an excess of corresponding amines, is a classic method for introducing substituents to (thia)calix[4]arenes via interaction between macrocyclic esters and amines. However, heterocyclic fragments are rarely introduced into the macrocycle structure. This is probably due to the limited number of commercially and synthetically available heterocyclic primary amines. Aminolysis of esters usually requires multiple excesses of amine, which makes regeneration difficult. There were examples of this synthetic way [246,247,248,249]. The reactions were carried out by refluxing in toluene/MeOH or DCM/MeOH systems in large excesses of amine (10–20 times per ester group) with yields of 55–82% for 1,3-disubstituted macrocyclic esters (Scheme 30). As a result, the **HetCA-63**–**HetCA-65** series were obtained.

Thus, we can conclude that nucleophilic substitution Williamson-type reactions are one of the most common approaches for obtaining **HetCA**s. This family of methods is distinguished by the simplicity of synthetic schemes and the low cost of the reagents used. As a rule, no special catalysts are required. The yields in the examples considered were 21–97%. These approaches mainly yielded 1,3-disubstituted calix[4]arene derivatives at the lower rim (Table S6) [226,227,241,242,243,250,251,252,253,254,255,256,257,258,259,260,261], but there were also numerous examples of tetrasubstituted derivatives being obtained (Table S7) [224,225,238,242,245,262,263,264,265].

#### 3.1.3. Cross-Linking Reactions

Over the past fifteen years, there has been a significant shift in the application of calixarene chemistry, moving from pure supramolecular chemistry to biomedical applications. As a result, researchers are changing their perspective and beginning to view calixarenes not only as a convenient platform for obtaining supramolecular structures, but also as the basis for potential biologically active substances. Reports on the synthesis of calixarene derivatives modified with amino acid residues, nitrogenous base fragments, carbohydrates, and dendritic structures have increased in frequency. Various biomimetic peptides and polysaccharides are obtained based on calixarenes, for which the Williamson and Schotten–Baumann reactions are often unsuitable [266]. This is due to the presence of multiple amino groups or fragments that are sensitive to strong bases, such as potassium carbonate. The methods based on Steglich synthesis or related processes, collectively referred to as “cross-linking reactions”, have proven to be very effective for obtaining such calixarene derivatives [85,267,268,269,270,271,272].

**Scheme 31:**
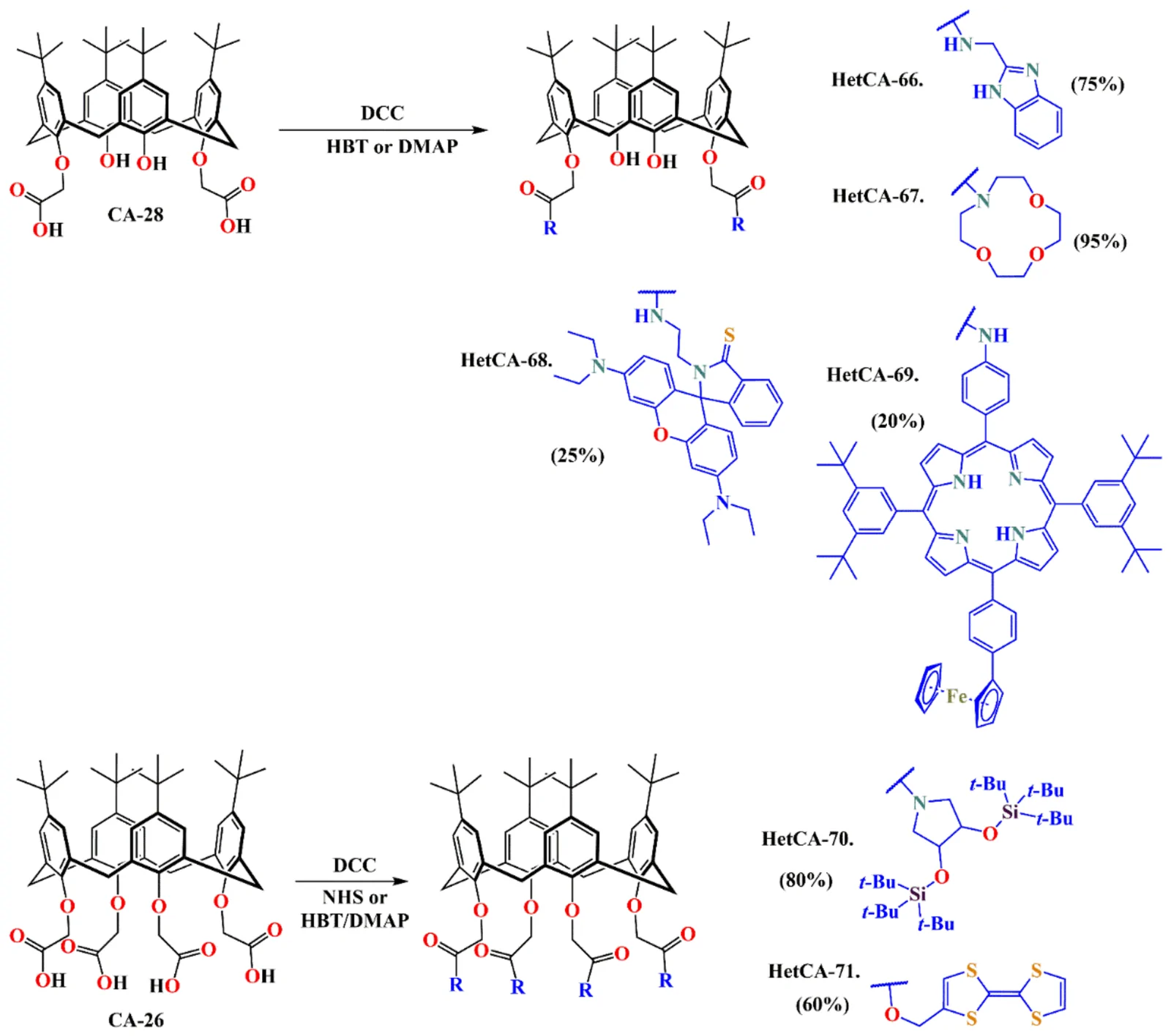
Syntheses of 1,3-disubstituted macrocyclic amides **HetCA-66**–**HetCA-69** and tetrasubstituted esters **HetCA-70**–**HetCA-71** by cross-linking reaction [273,274,275,276,287,288] NHS—*N*-hydroxysuccinimide, HBT—hydroxybenzotriazole.

To obtain **HetCA**s via cross-linking (or Steglich-like) reactions, carboxylate derivatives of macrocycles and primary or secondary amines of both aliphatic and aromatic nature are typically used. The products of such reactions were stable amides like macrocycles **HetCA-66**–**HetCA-68** [273,274,275]. As can be seen from Scheme 31, Steglich-like reactions allowed the use of a wide variety of substrates, including compound **HetCA-69** containing porphyrin fragments modified with ferrocene [276]. Along with this method, other schemes for introducing the porphyrin cycle into *meta*-cyclophane have been implemented previously [277,278,279,280,281,282,283]. The catalyst most commonly used is *p*-(*N*,*N*-dimethylamino)pyridine (DMAP), either alone or in combination with hydroxybenzotriazole (HBT) or 1-ethyl-3-(3-dimethylaminopropyl)carbodiimide (EDCI) [284]. Sometimes the benzotriazol-1-yl-oxytripyrrolidinephosphonium hexafluorophosphate (PyBOP)/DIPEA system can be used [285]. Cross-linking reactions could also be used to produce esters, which are more sensitive to temperature and acidity than amides [286,287].

**Scheme 32:**
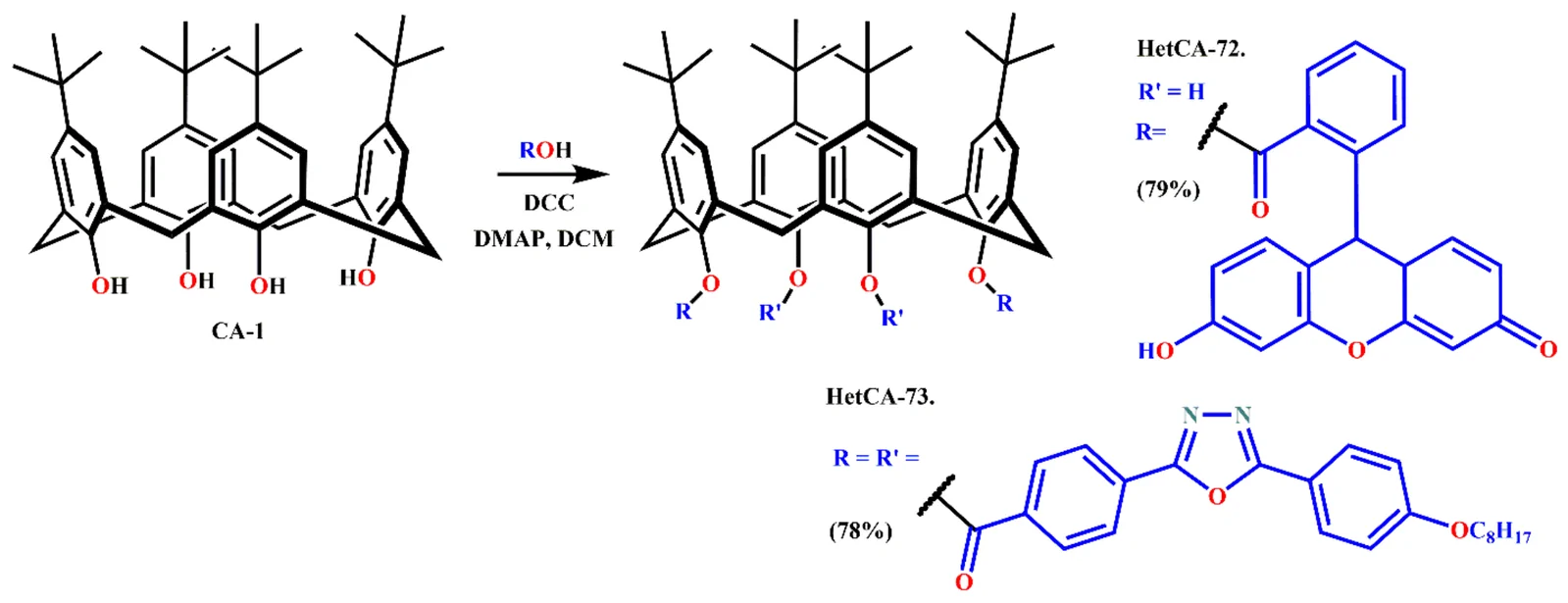
Use of cross-linking reagents for **HetCA-72**–**HetCA-73** synthesis [289,290].

In addition to the approaches described above, there were others in which the phenolic hydroxyl group of calixarene **CA-1** is involved in the cross-linking reaction, and the heterocycle was introduced to synthesize macrocycles **HetCA-72**–**HetCA-73** (Scheme 32) [289,290].

**Scheme 33:**
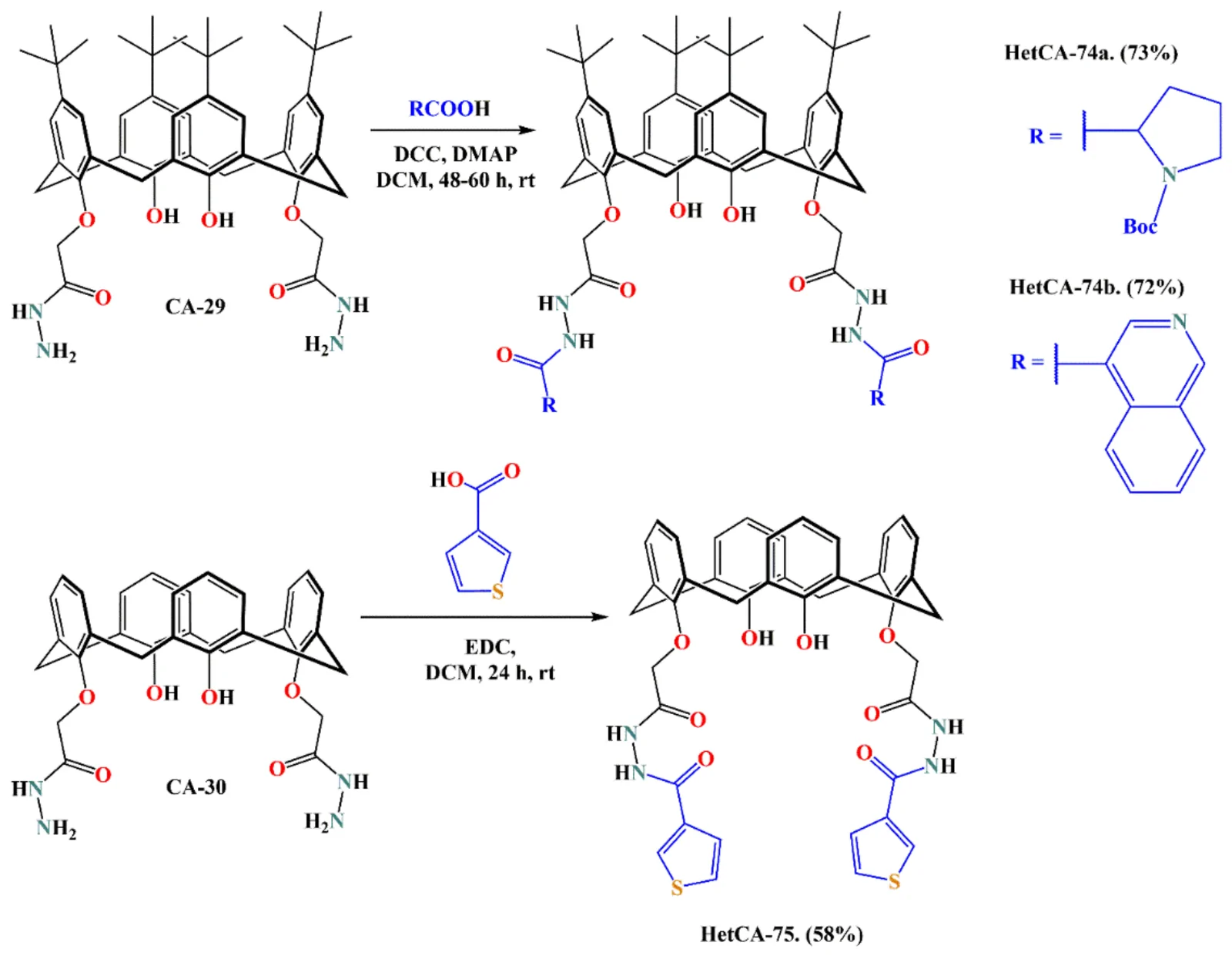
Synthesis of 1,2-diacylhydrazine derivatives **HetCA-74**–**HetCA-75** [291,292,293].

There are also non-mainstream synthesis routes using cross-linking reagents. These reactions are of interest in terms of mechanism and further research. We mention here cases where calixarene was used in the form of a hydrazide derivative, **CA-29**–**CA-30,** to obtain bis-(1,2-diacylhydrazine) derivatives **HetCA-74**–**HetCA-75** (Scheme 33) [291,292,293].

**Scheme 34:**
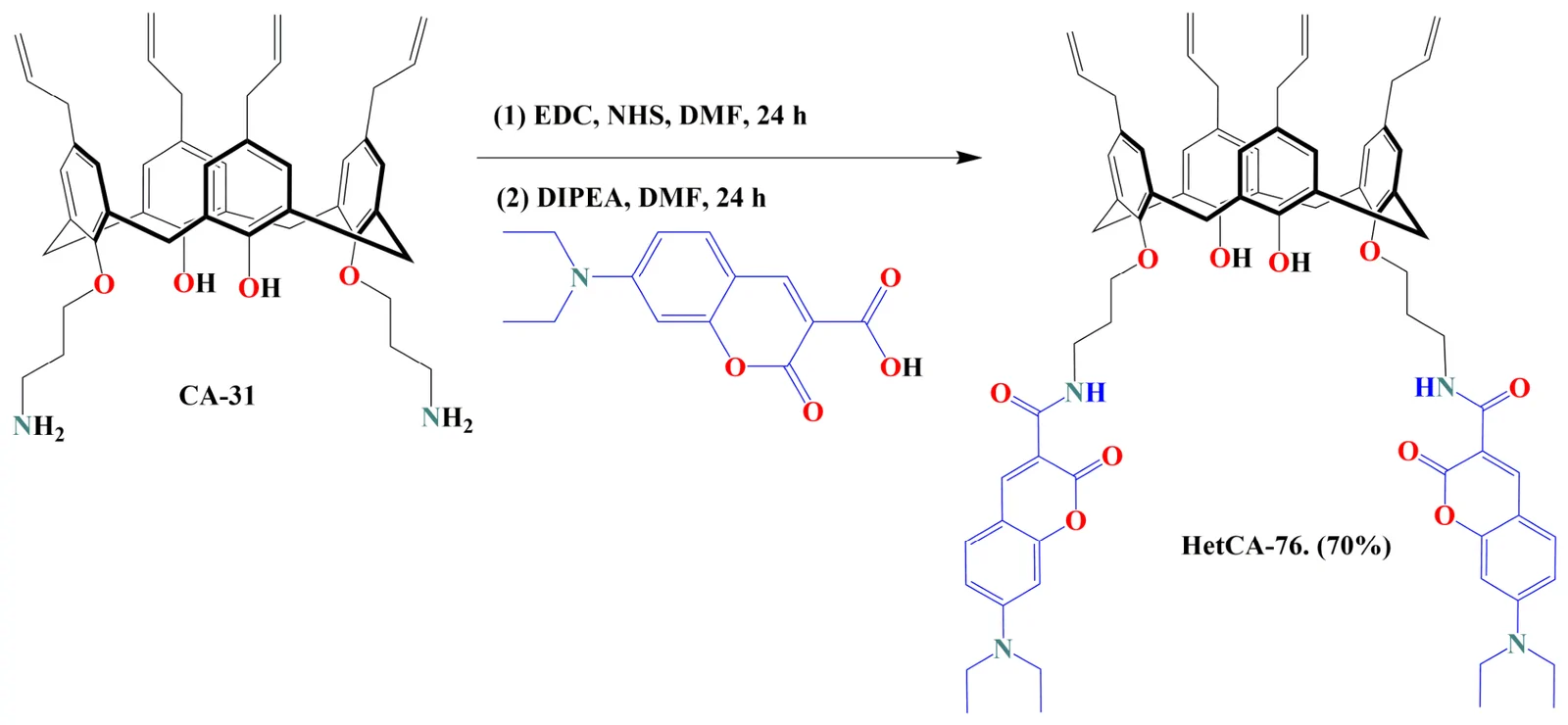
Synthesis of bis-coumarin derivative **HetCA-76** [294].

In addition, there are synthetic approaches that do not fit into Scheme 31, Scheme 32 and Scheme 33. For example, Uttam et al. described the synthesis of a bis-coumarin derivative **HetCA-76** using an activated ester of a heterocyclic carboxylic acid with a yield of 70% in the final stage (Scheme 34) [294]. In fact, the authors divided the classical version of the Steglich reaction into the synthesis of the activated ester and the formation of the target amide.

As a conclusion to this section, we would like to note that cross-linking reactions proceed under very mild conditions, in the temperature range from 0 to 50–60 °C (rarely higher), usually in the presence of the cross-linking agent dicyclohexylcarbodiimide (DCC), 1-ethyl-3-(3-dimethylaminopropyl)carbodiimide (EDC), or (benzotriazol-1-yloxy)tripyrrolidinophosphonium hexafluorophosphatehexafluorophophate (PyBOP). Cross-linking reagents are often used in combination with bases, which may include *N*,*N*-dimethylaminopyridine (DMAP) and hydroxybenzotriazole (HBT), in the presence of catalytic or equimolar amounts of an organic base. Methylene chloride is usually used as a solvent, which is convenient because it produces a by-product precipitate that can be easily removed by filtration. However, in some cases, the solvent can be replaced with ethyl acetate or even DMF if the starting materials are not soluble in organochlorine solvents. This approach is similar to peptide synthesis and can therefore be successfully applied for covalent modification of macrocyclic calixarene platforms with biological compounds. Tables S8 and S9 summarize the experimental data on the synthesis of **HetCA**s using for cross-linking reactions [258,273,274,276,285,286,287,288,289,290,291,292,293,295,296,297,298,299,300,301,302,303,304,305,306,307,308,309,310].

#### 3.1.4. Azide–Alkyne Cycloaddition (AAC)

In addition to cross-linking reactions, azide–alkyne coupling (AAC), also known as the Huisgen reaction or the click reaction, has become a widely used method for preparing **HetCA**s over the past 15 years. A. Michael first obtained triazole in 1893 by reacting phenylazide with dimethyl ether of acetylenedicarboxylic acid [311]. Subsequently, in the 1960s, German chemist R. Huisgen studied this reaction in a non-catalytic variant [312,313]. The modern copper-catalyzed version (CuAAC) was independently proposed by teams led by M. Meldal (Denmark) [314], V. Fokin (Russia), and B. Sharpless (USA) [315] in 2002. Twenty years later, B. Sharpless was awarded the Nobel Prize in Chemistry for this discovery, which he shared with C. Bertozzi [316]. CuAAC reactions comply with the basic principles of green chemistry, such as atom economy, synthesis selectivity, use of room temperature, and avoidance of protective groups [317]. The approach using click reactions is expected to find wide application in biomedical research since these reactions are rapid and produce high yields with minimal by-product formation and high chemo- and regioselectivity [318].

In formal terms, azide–alkyne coupling can be considered an example of introducing a heterocycle (namely, 1,2,3-triazine) into a macrocycle via a divergent route. A vast amount of data exists on the introduction of a 1,2,3-triazole fragment into the structure of calixarene [318,319,320,321,322,323,324,325,326,327,328,329,330,331,332,333,334,335,336,337,338,339,340,341,342,343,344,345,346,347,348,349,350,351,352,353,354,355,356,357,358], including the synthesis of bis-calixarene with a large intramolecular cavity [359,360], or a macrocyclic dendrimer containing ferrocene fragments [361]. It should be noted separately that approaches have already been described that allow the production of triazole-modified calixarene derivatives without using copper(I) as a catalyst [362]. However, we decided to focus only on studies that introduce other heterocyclic fragments using CuAAC reactions. This choice is motivated by the growing interest in biomimetic calixarene systems, as the introduction of saccharide fragments imparts enhanced biocompatibility [363], molecular recognition capability [364], and specificity toward biological targets [365]. The use of 1,2,3-triazole as a linker is particularly advantageous due to its high chemical and metabolic stability [366], ability to participate in hydrogen bonding [367], and facile introduction via the well-established “click chemistry” approach. Moreover, such triazole-linked glycosyl calix[4]arenes combine the structural preorganization and conformational rigidity of the calixarene scaffold with the biological relevance of carbohydrate residues, providing promising architectures for applications in molecular recognition [368], drug delivery [369], and biosensing [370].

There are several methods of introducing carbohydrate residues into the calix[4]arene structure, one of which is the triflate activation method [371,372], nucleophilic substituition [373], addition via a thiourea bridge [374,375], the Wittig reaction followed by hydrogenation of the alkene double bond on a palladium catalyst [376], the cross-linking reactions [377], the Mitsunobu reaction [378], and the Suzuki cross-coupling [379]. However, over the past fifteen years, the most popular method for introducing sugar residues into the structure of meta-cyclophane has been techniques based on azide–alkyne cycloaddition.

**Scheme 35:**
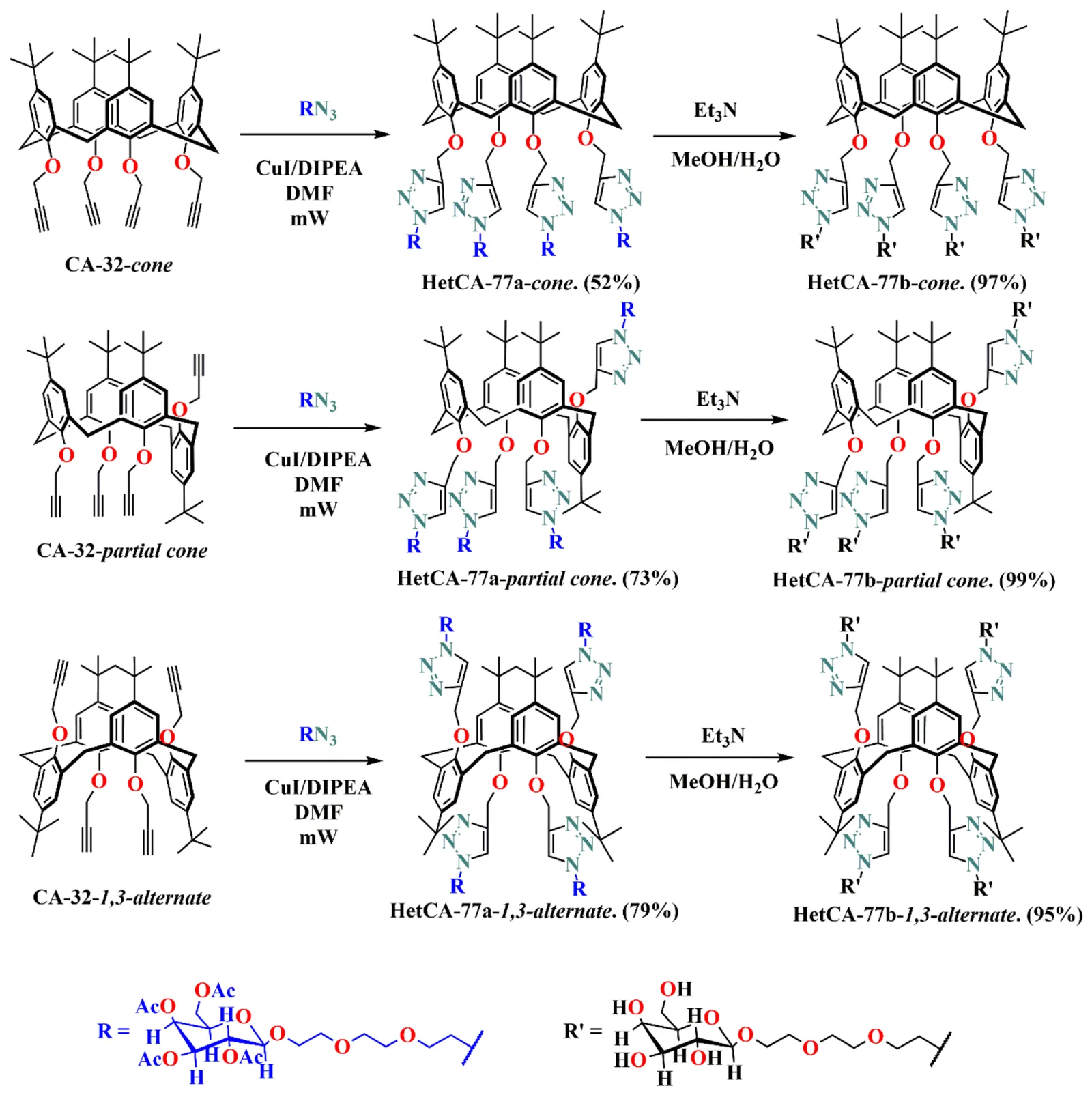
*D*-Galactopyranose derivatives **HetCA-77** obtained by azide–alkyne coupling [383].

The starting compounds are often *p*-*tert*-butylcalix[4]arenes modified with one, two [380] or four propargyl fragments [75,76,346,368,381,382]. Saccharide derivatives in *cone*, *partial cone*, and *1,3-alternate* conformations are also described for fully substituted propargyl derivatives of the macrocycle [383]. Examples of syntheses are shown in Scheme 35. Click coupling of an azide containing a cyclic saccharide fragment under the influence of microwave radiation proceeded with 52–79% yields of **HetCA-77a**, and removal of acetyl protection from the hydroxyl groups of the saccharide to **HetCA-77b** proceeded almost quantitatively. The choice of triethylene glycol linkers is related to a number of factors, including the increased flexibility of the chains, which determines their spatial freedom, as well as the hydrophilicity of this fragment. In the same work [383], the authors experiment with more hydrophobic and rigid linkers, analyzing the affinity of ligands containing them for lectins.

**Scheme 36:**
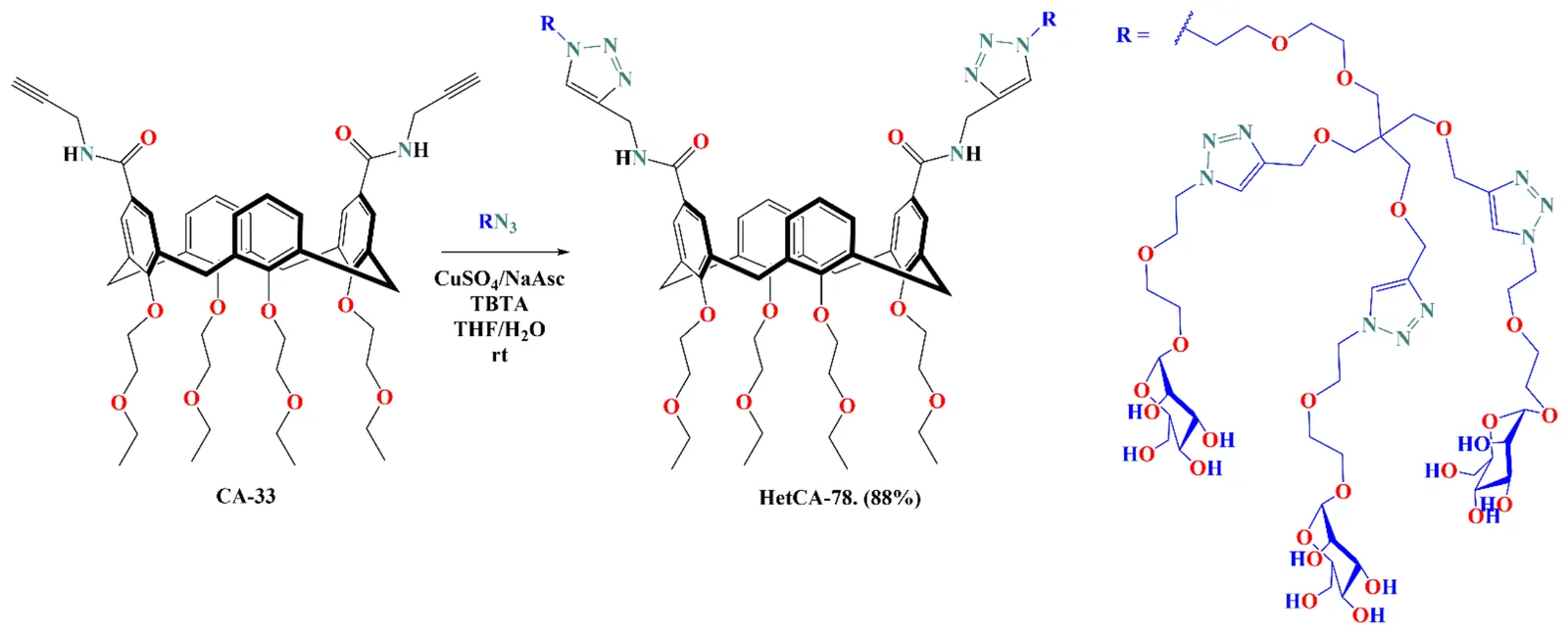
Modification of calix[4]arene **CA-33** with saccharides on the upper rim using CuAAC [384].

In addition to modifying the lower rim of the calixarene, Palmioli et al. recently reported that the upper rim can also be modified to introduce heterocyclic structures via azide–alkyne coupling [384]. The upper rim of the calixarene **CA-33** was modified with a *D*-α-mannopyranose residue that already contained a 1,2,3-triazole moiety, achieving **HetCA-78** with 88% yield (Scheme 36). The modification of the upper rim was chosen due to its greater “openness” compared to the lower rim, which is due to the “sort of bowl” form of calix[4]arene and is manifested in a decrease in the degree of shielding of the carbohydrate fragments by the macrocyclic platform. Copper(I) was obtained in situ from CuSO_4_ in the presence of sodium ascorbate as a reducing agent and catalytic amounts of *tris*-(benzyltriazolylmethyl)amine (TBTA) in a mixture of THF:water (1:1) at room temperature for 7 days [384]. The nature of the linker is determined by the goal of obtaining a water-soluble compound with multiple binding sites for the substrate, namely lectin.

**Scheme 37:**
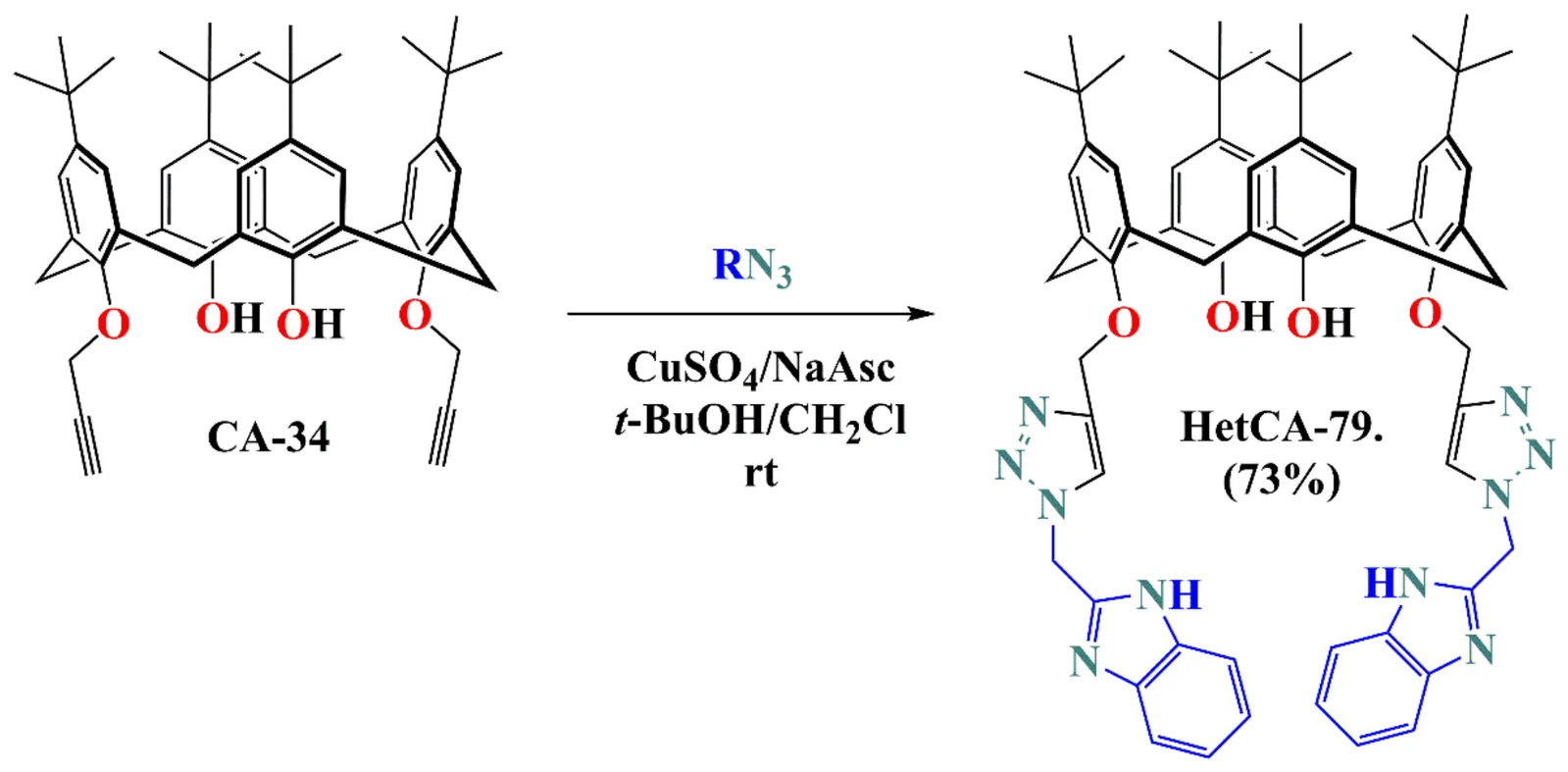
Synthesis of **HetCA-79** with benzimidazole fragments [385].

However, the Huisgen reaction can be used not only for introducing carbohydrate residues into macrocycle structures. Thus, we can provide an example of how to obtain the benzimidazole derivative **HetCA-79**, which exhibits the property of acting as a fluorescent sensor for Cu(II) cations (Scheme 37). The copper catalyst is formed in the same way as in the previous case, but no additional co-catalyst is required [385].

**Scheme 38:**
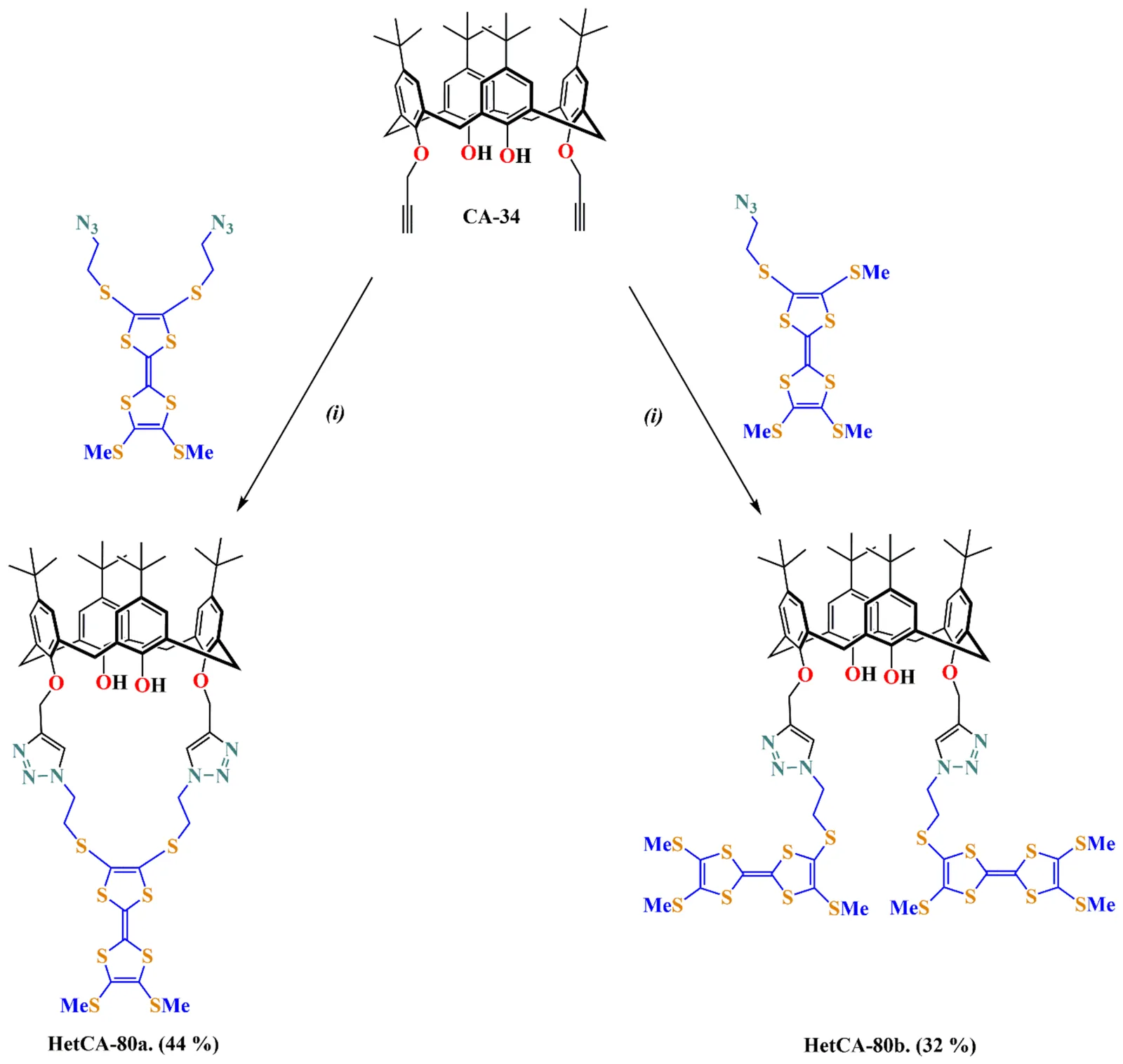
Synthetic route to **HetCA**s; (*i*) CuSO_4_ × 5H_2_O, sodium ascorbate, DMF, 90 °C, 10 h [386].

Zhao et al. reported [386,387] the use of azide–alkyne coupling to obtain electrochemically active tetrathiafulvalene derivatives of *p*-*tert*-butylcalix[4]arene **HetCA-80** (Scheme 38). It is worth noting that the choice of this fragment is due, among other things, to the fact that the 1,2,3-triazole fragment does not participate in redox processes under the same conditions as the TTF fragment. The synthetic approach used is interesting in part because diazide was used to introduce a heterocyclic fragment containing two propargyl moieties into the macrocycle. The yields of the mono- and di-derivatives were 44 and 32%, respectively.

**Scheme 39:**
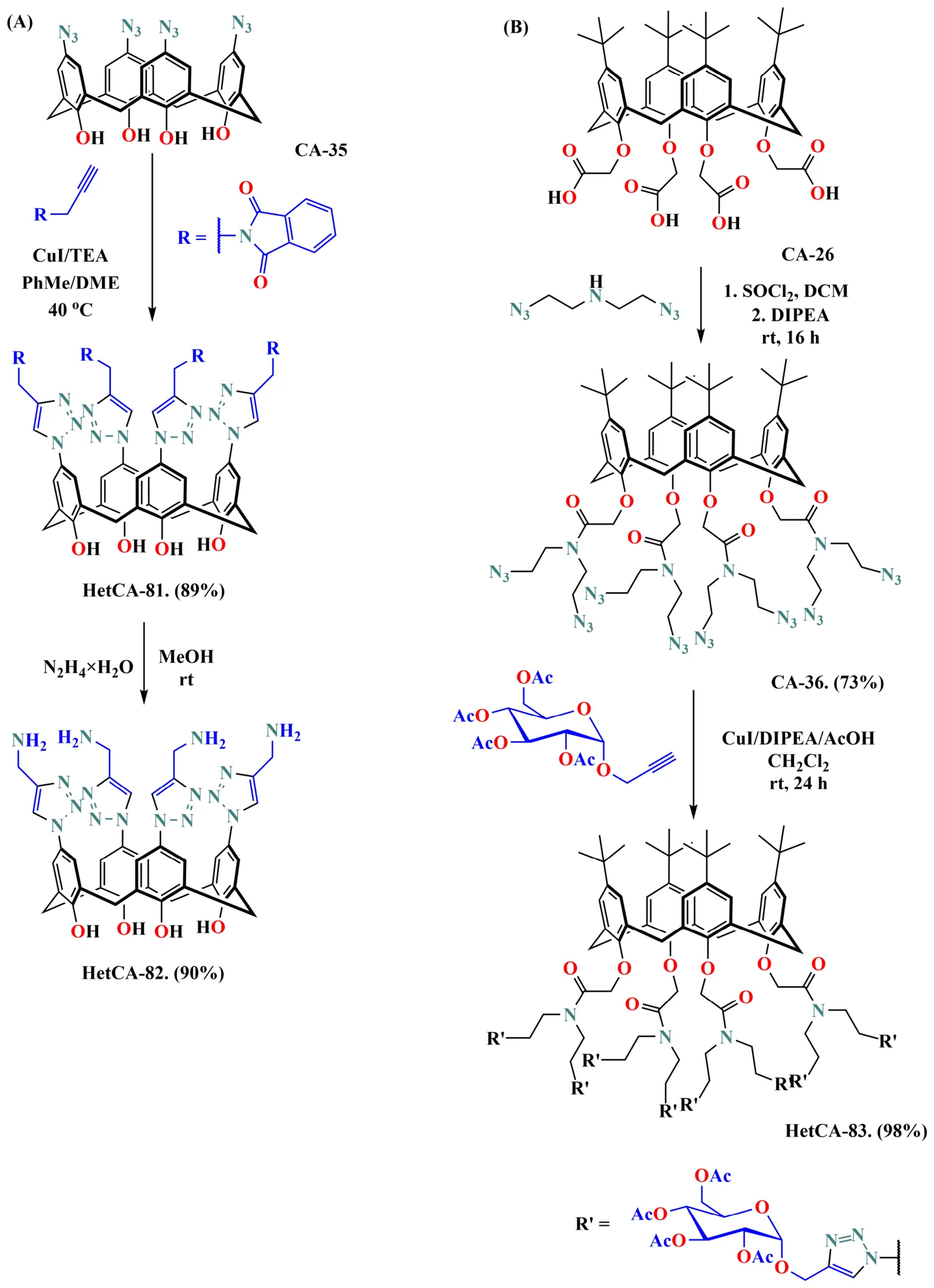
Two-step synthesis of (**A**) **HetCA-82** through the introduction of phthalimide fragments by azide–alkyne coupling [391]; (**B**) *D*-β-glucopyranose octaderivative **HetCA-83** [392].

Furthermore, various calixarene derivatives modified with an –N_3_ group at both the lower and upper rims can be considered azide components. Such examples can be found in the literature [388,389,390]. Recent work [391] on a modification of the upper rim of calixarene **CA-35** described a two-stage method for introducing an aminomethyl fragment via the Gabriel reaction, preceded by the introduction of a phthalimide fragment into the macrocycle structure. The presence of free phenolic hydroxyls did not prevent the formation of the heterocyclic product **HetCA-81** and its subsequent cleavage to tetraamine **HetCA-82** with high yields (Scheme 39A). In 2018, Cindro et al. demonstrated that a derivative containing carbohydrates, **HetCA-83,** with sensory properties for sodium cations could be obtained from calixarene tetracarboxylic acid **CA-26** in two stages (Scheme 39B). Noteworthy is the formation of the octaazide derivative **CA-36**, which yields the octaglycoside derivative of *D*-β-glucopyranose **HetCA-83** in almost quantitative yield [392]. The role of the *D*-glucopyranose fragments in this case is to impart hydrophilicity to macrocycle **HetCA-83**, while the amide carbonyls are needed to coordinate the “hard” Na(I) cations. In addition, the authors emphasize that the choice of CuAAC was due to the desire to carry out reactions without excess reagents to simplify the purification process, and the introduction of 1,2,3-triazole fragments rigidly fixes the glucose residues, preventing them from binding to each other, maintaining the solubility of the macrocycle.

The key advantage of the CuAAC approach is the mild conditions (room temperature, aqueous–organic mixtures, and absence of strong bases), which allow the introduction of sensitive moieties (sugars, peptides, and nucleic acid bases) without disrupting their stereochemistry and reactivity. Furthermore, CuAAC proceeds in high yield even with four substituents (Table S10), preserving the original macrocycle conformation, which is critical for the preparation of glycoclusters and bioconjugates. In this sense, CuAAC has become a bridge between classical synthetic chemistry and the chemistry of biologically active compounds.

Thus, azide–alkyne coupling is a promising method for obtaining heterocyclic derivatives of calixarenes [75,76,368,384,385,386,387,388,389,390,391,392,393,394,395,396,397]. Table S10 shows the experimental data for this reaction type using propargyl derivatives of calix[4]arenes as precursors, while examples using macrocyclic azides as initial compounds are shown in Table S11. As a rule, systems that are standard for click reactions are used: copper(I) iodide or CuSO_4_/NaAsc. TEA, DIPEA, or tris-(benzyltriazolylmethyl)amine (TBTA) can be used as a base. Various solvents are used in click reactions, e.g., DMF, acetonitrile, dioxane/water, tert-butanol/methylene chloride, THF/water, and toluene/dimethoxyethane. Both di- and tetra-derivatives of calixarenes can be obtained using click reactions. Yields ranged from 11 to 99%. For further reading, we recommend the review article of Barthélémy and co-workers [393].

#### 3.1.5. Synthesis of Schiff Bases, Hydrazones, and Related Compounds

**Scheme 40:**
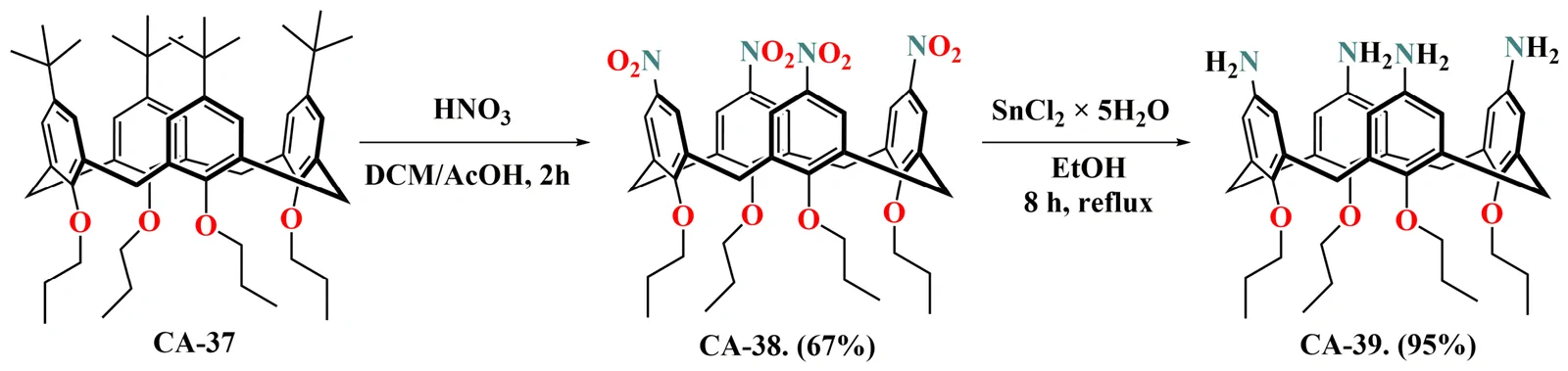
Synthesis of aminocalix[4]arene **CA-39** [398,399].

One of the most common methods of obtaining **HetCA**s involves the interaction of heterocyclic aldehydes with macrocycles that have strong nucleophilic centers, e.g., amino groups or hydrazide fragments, in side chains. The synthesis of amine or hydrazide calixarene derivatives has been streamlined for a long time. Such compounds are extremely useful as starting materials due to the significant synthetic potential of their nitrogen-containing fragments. In this section, we would like to describe some approaches for obtaining amino derivatives of (thia)calixarenes as precursors for synthesizing macrocyclic Schiff bases and related compounds. One of the earliest derivatives is aminocalix[4]arene **CA-39**, first synthesized in 1997 by D. N. Reinhoudt’s group at the University of Twente (The Netherlands) from the corresponding nitrocalix[4]arene **CA-38** (Scheme 40) [398], which had previously been obtained by the same research group [399]. The reaction was carried out in two stages: first, *ipso*-nitration of *p*-*tert*-butylcalix[4]arene, followed by reduction in the nitro groups to amino fragments with almost quantitative yield.

**Scheme 41:**
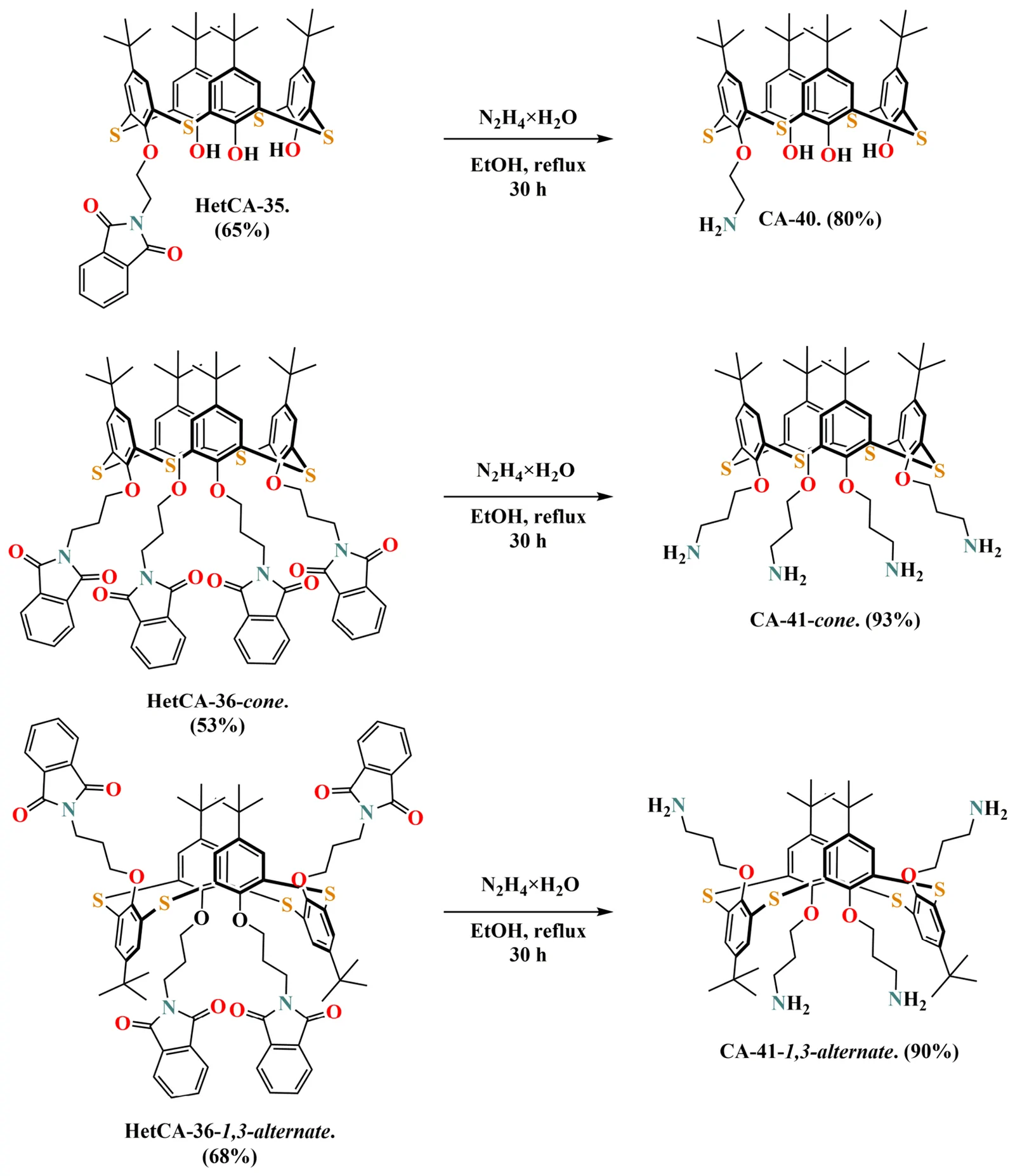
Synthesis of macrocycles **CA-40** and **CA-41** with primary amino groups [212,213,400].

Logically, the next stage of development should involve modification of the lower rim. Significant progress has particularly been made in this area for thiacalix[4]arenes. Historically, the first method for obtaining amino derivatives of thiacalixarenes was the use of alkylating agents based on *N*-substituted phthalimides [212,213,400]. Furthermore, the structure of the products is greatly affected by the length of the linker used. Thus, the use of *N*-(2-bromoethyl)phthalimide led to a monoalkylation product, **HetCA-35,** regardless of the ratio of reagents, the nature of the base, and the solvent. Moreover, lengthening the linker by just one carbon atom allowed for the production of tetrasubstituted thiacalix[4]arenes, **HetCA-36,** in *cone* and *1,3-alternate* conformations. The ratio of these conformers is regulated by the templating effect of Na(I) and Cs(I) cations, respectively [212,213,400]. Hydrazine hydrate initiated the Gabriel reaction, resulting in the cleavage of the phthalimide fragment and the deprotection of free primary amino derivatives **CA-40** and **CA-41** (Scheme 41).

**Scheme 42:**
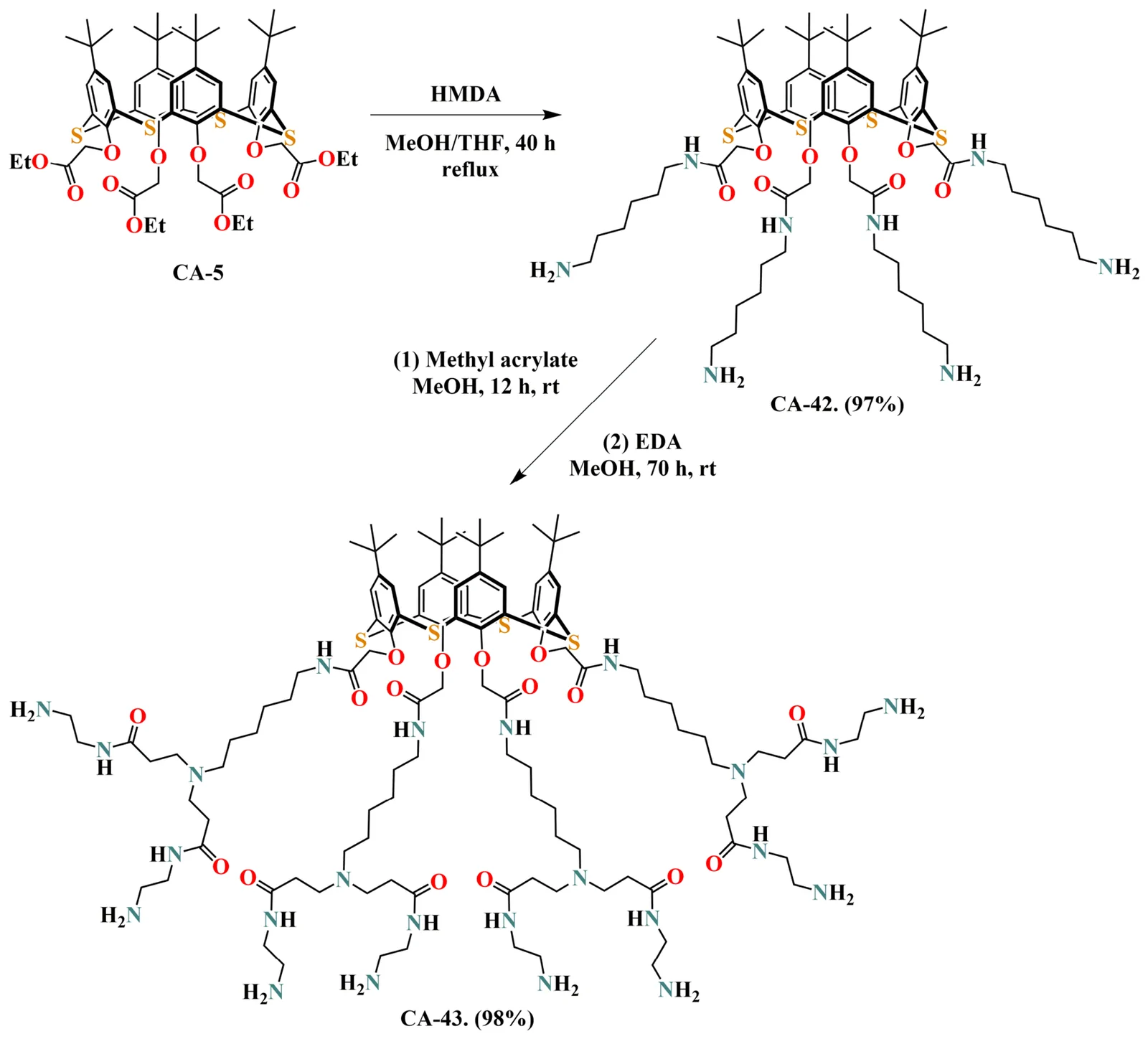
Synthesis of tetra- and octaamine thiacalixarene derivatives **CA-42** and **CA-43** [49,401].

Years later, a different scheme for obtaining macrocyclic tetraamines with a longer linker was realized. Thus, it was demonstrated that the aminolysis of calixarene tetraesters **CA-5** with hexamethylenediamine resulted in the formation of the respective tetraamines **CA-42** [401] (Scheme 42). Then, tetraamines **CA-42** were used as starting compounds for poly(amidoamine) (PAMAM) derivatives of thiacalix[4]arene [49]. Similar structures were implemented in three stereoisomeric forms: *cone*, *partial cone*, and *1,3-alternate*. Compound **CA-43** is a so-called first-generation PAMAM-calix-dendrimer. The authors were also able to obtain PAMAM and PPI dendrimers based on *p-tert-*butylthiacalix[4]arene of higher generations (second and third) [402,403,404,405,406,407].

**Scheme 43:**
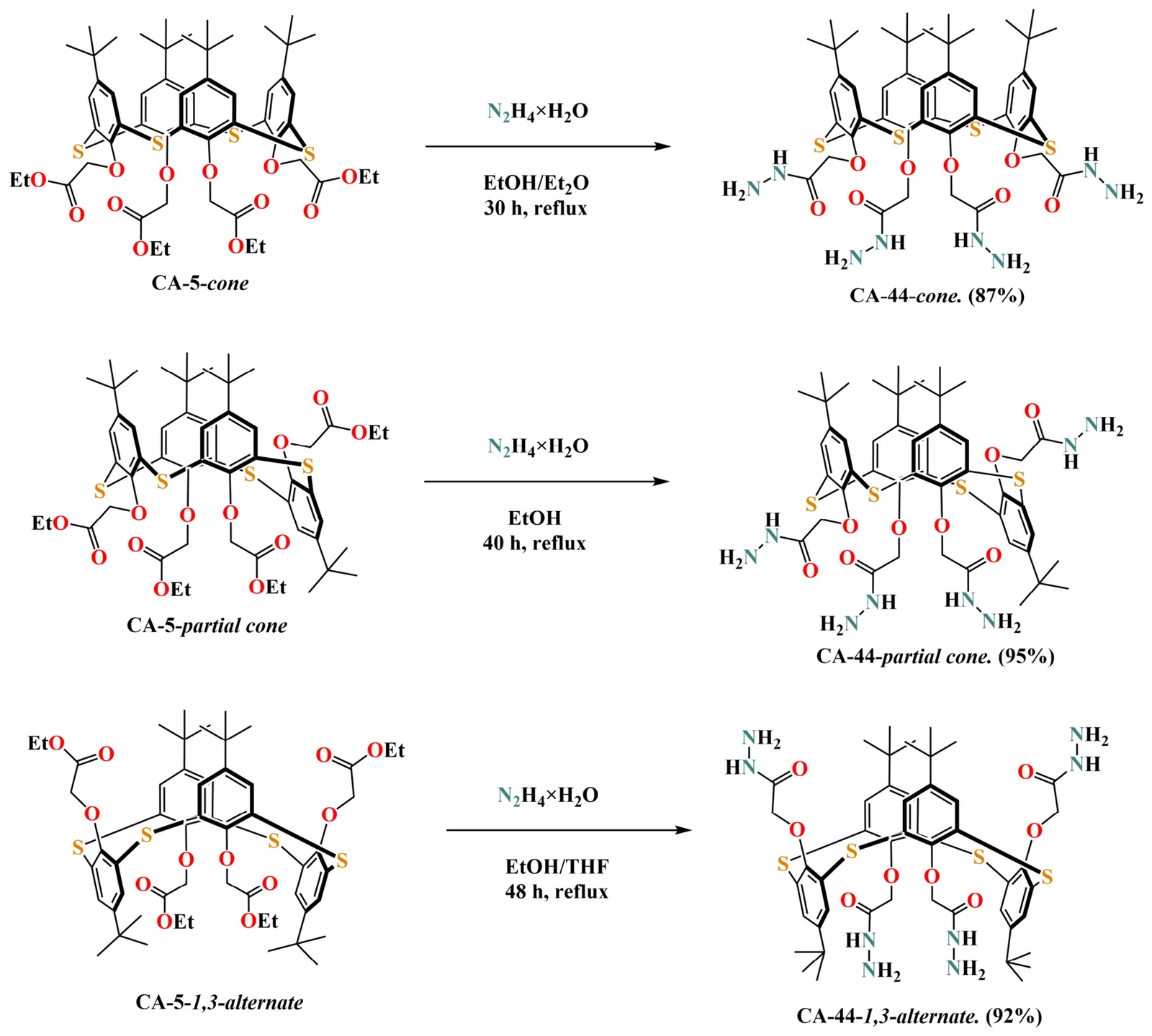
Synthesis of macrocyclic hydrazides **CA-44** [408,409].

In addition to macrocyclic amines, approaches for obtaining hydrazides based on calixarene tetraethers have also been developed. The synthesis is carried out according to a fairly simple scheme of nucleophilic hydrazinolysis of the tetraesters **CA-5**. Thus, *p*-*tert*-butylthiacalix[4]arene-based hydrazides **CA-44** were obtained with good yields for all three most common stereoisomeric forms [408,409] (Scheme 43).

**Scheme 44:**
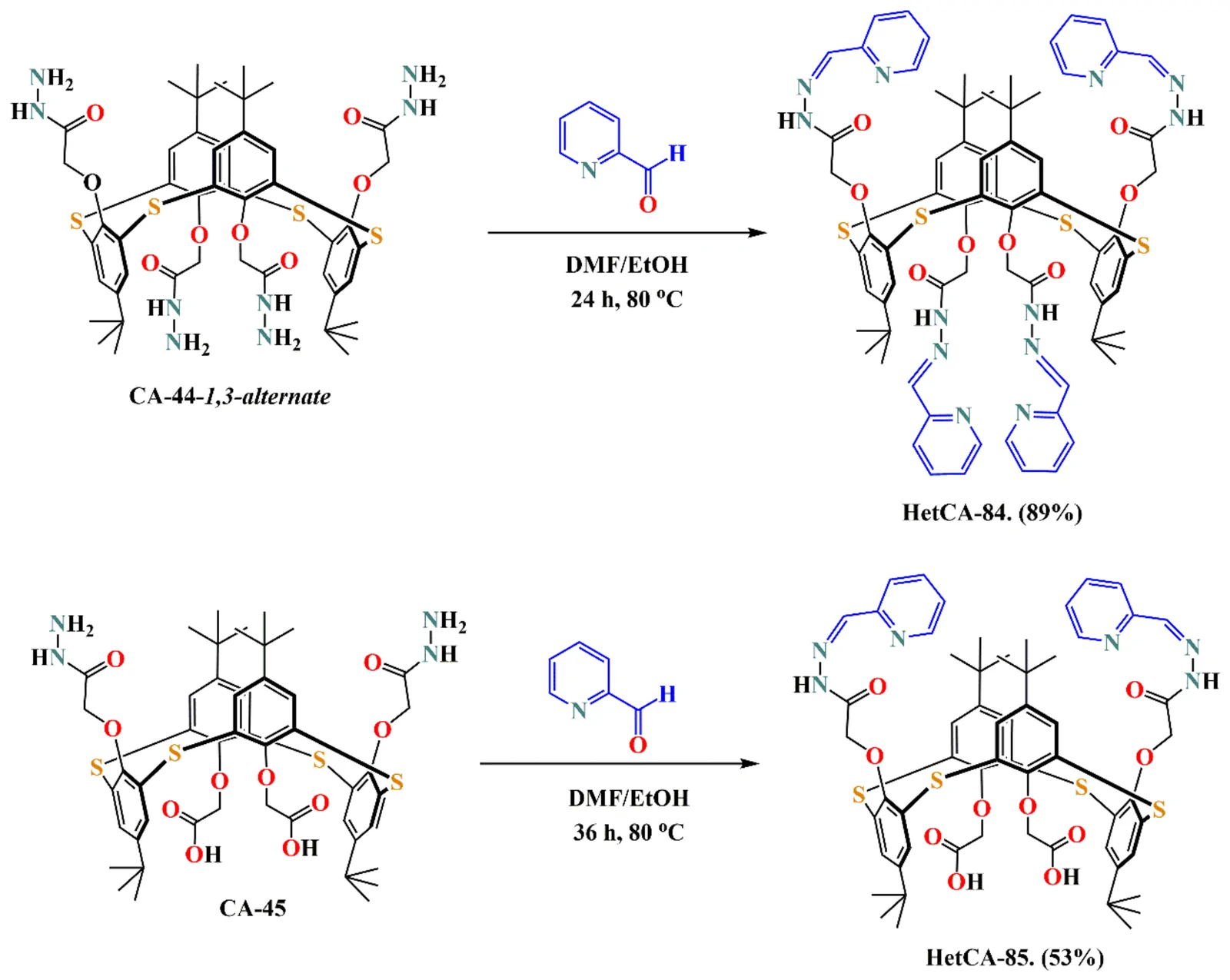
Synthesis of hydrazones **HetCA-84** [413] and **HetCA-85** [414].

Based on the hydrazides **CA-44**, a number of hydrazones were subsequently obtained, in particular, derivatives of the *o*-isomer of nicotinic aldehyde **HetCA-84** (Scheme 44) [410,411,412,413]. In addition, approaches were developed for obtaining dihydrazides **CA-45** based on *p*-*tert*-butylthiacalix[4]arenes [414]. Thus, hydrazone **HetCA-85** was obtained based on the *o*-isomer of nicotinic aldehyde with a yield of 53%.

**Scheme 45:**
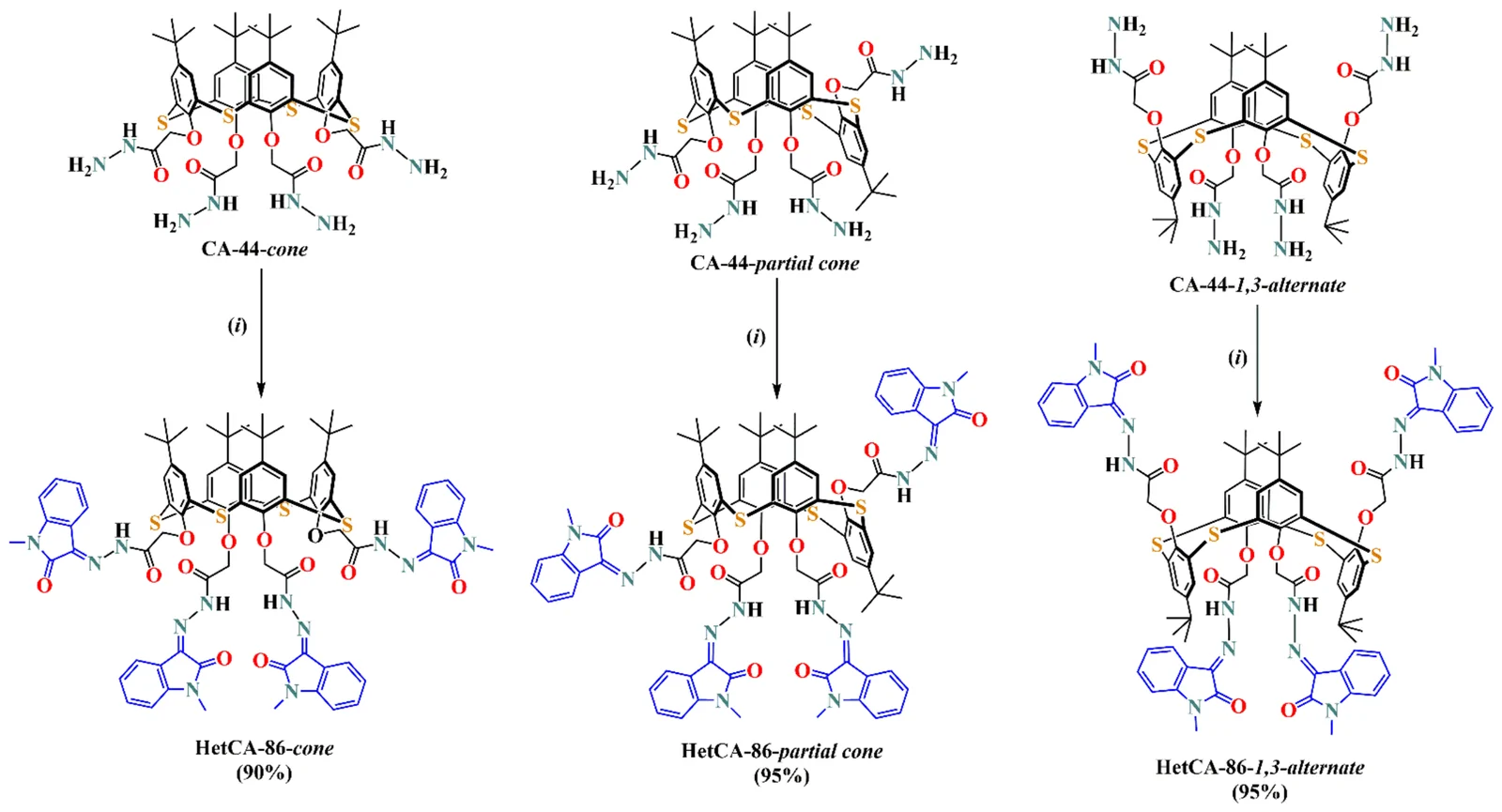
Synthesis of **HetCA-86**: (*i*) *N*-methylisatin, EtOH, reflux, 24 h [415].

Besides pyridine derivatives, there are also **HetCA-86** obtained from the indole derivative *N*-methylisatin [415]. The reaction was carried out by refluxing in anhydrous ethanol for 24 h to obtain **HetCA-86** with 90–95% yields from hydrazides **CA-44** (Scheme 45).

**Scheme 46:**
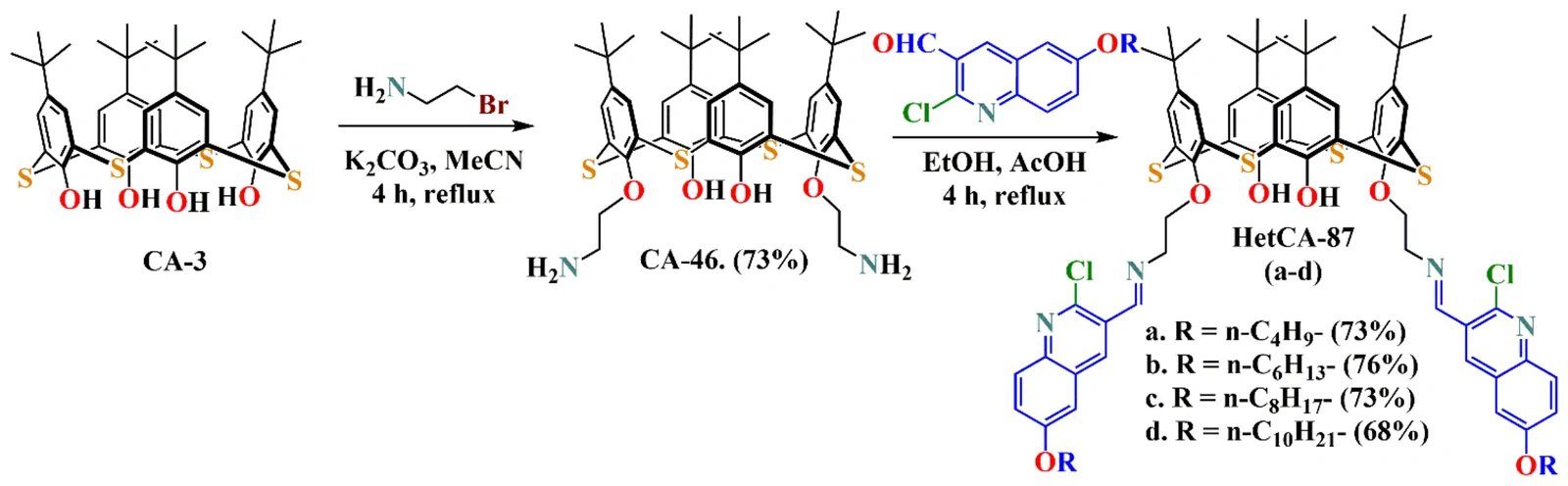
Synthesis of quinoline thiacalixarene conjugates **HetCA-87** [416].

Hydrazides are not only used to synthesize calixarene derivatives with imine groups. For example, Rathod et al. reported the synthesis of Schiff base **HetCA-87** via diamine **CA-46** with moderate yields (68–76%) (Scheme 46) [416]. The resulting compound is capable of fluorescence in the blue range, thus acting as a component of potential OLEDs.

**Scheme 47:**
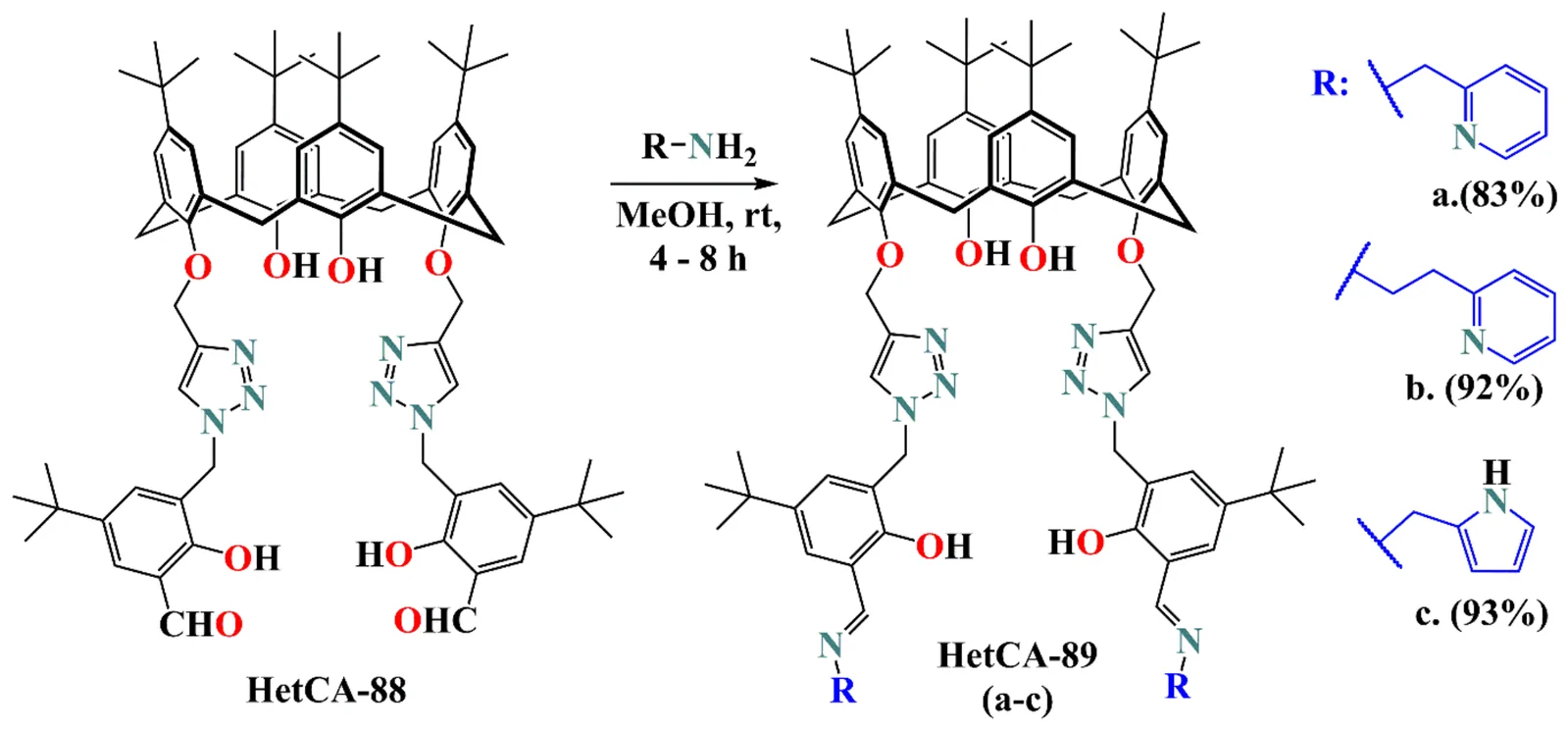
Synthesis of Schiff bases **HetCA-89** [421,422,423].

A series of macrocyclic imines were made by Prof. C.P. Rao’s group at the Indian Institute of Technology Bombay (India) [417,418,419,420,421,422,423]. In all of the mentioned works [417,418,419,420,421,422,423], macrocyclic Schiff bases **HetCA-89** were synthesized from aldehyde derivatives **HetCA-88** without the use of catalysts under mild conditions. The summarized results of **HetCA** syntheses from the works [421,422,423] are presented in Scheme 47.

**Scheme 48:**
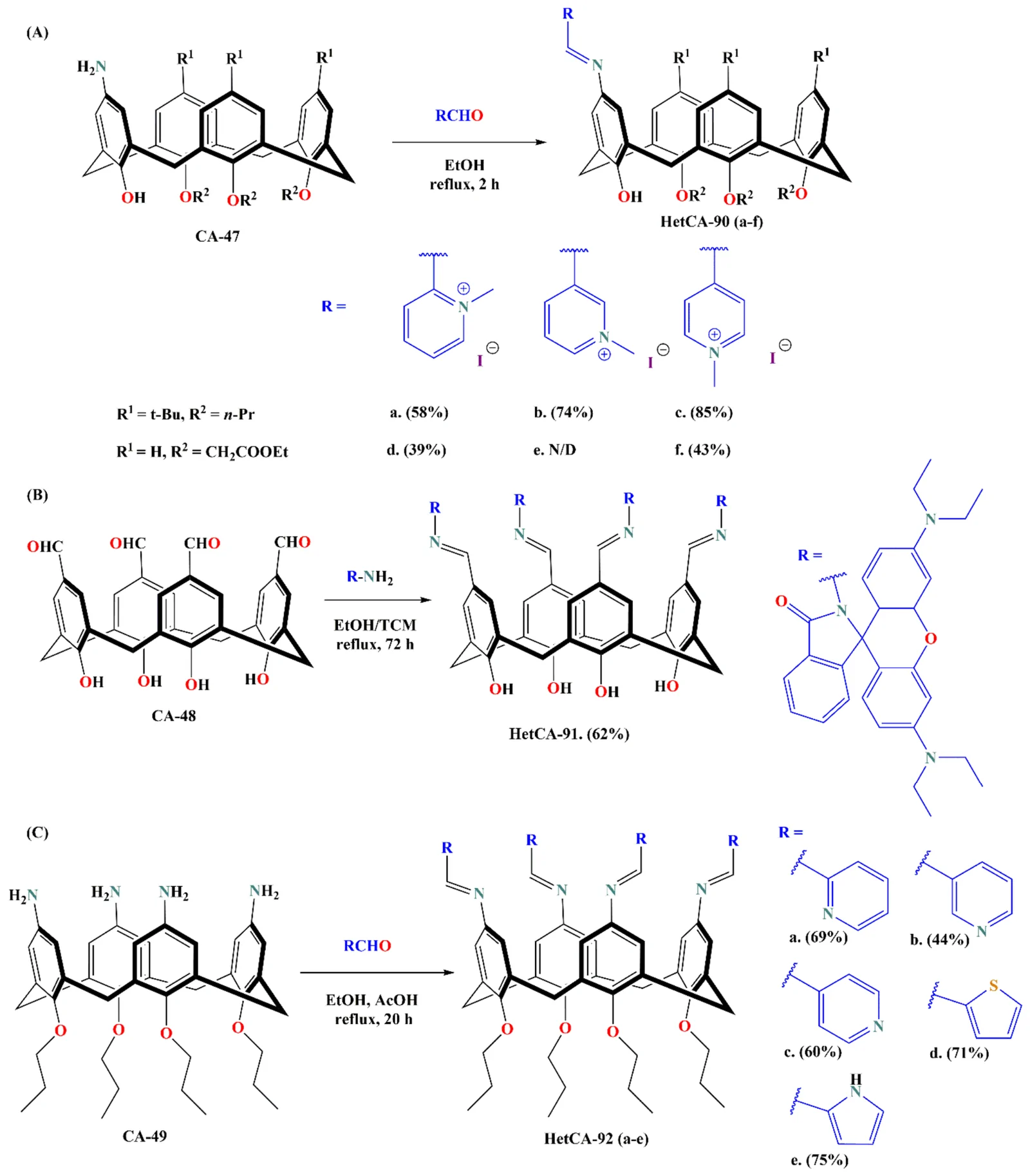
Synthesis of (**A**) pyridinium **HetCA-90** [424], (**B**) rhodamine conjugate **HetCA-91** [425] and (**C**) **HetCA-92** [426] via imine bond formation.

Sometimes, quaternary ammonium structures can be incorporated into macrocyclic derivatives in a ready-made form. For example, calixarenes **CA-47** can be modified with methylpyridinium by replacing the aldehyde groups with nicotinic aldehyde derivatives that contain a quaternary nitrogen atom in the pyridine ring to produce **HetCA-90** (Scheme 48A) [424]. Since the Schiff base formation reaction proceeds easily, this method can even be used in the case of **HetCA-91** to introduce the sterically hindered dye Rhodamine A derivative at the upper rim of the macrocycle **CA-48** [425]. Specifically, an EtOH/TCM system was used for this purpose, and the reaction proceeded for 72 h with a yield of 62% (Scheme 48B). There were also Schiff macrocyclic bases **HetCA-92** containing a heterocyclic fragment on the upper rim (Scheme 48C) [426]. The resulting macrocycles **HetCA-90**–**HetCA-92** were synthesized with moderate yields.

Thus, the synthesis of calixarene-based imines and hydrazones is one of the most convenient methods for introducing heterocyclic fragments into the macrocyclic structure, which is ensured by the diversity of structures of macrocyclic nitrogen-containing nucleophiles and the different nature of heterocyclic carbonyl electrophilic reagents. The reactions often proceed relatively quickly, with good yields, and do not require sophisticated catalysts. Table S12 summarizes the methods for synthesizing imines and hydrazones based on **HetCA** [413,415,416,421,422,423,424,425,426]. Alcohols are typically used as solvents. Reactions are carried out both under heating and at room temperature. Catalytic (with the addition of acetic acid) and non-catalytic variants are described.

#### 3.1.6. Electrophilic Addition to a Tertiary Nitrogen Atom

One of the notable features of calix[4]arenes is the high hydrophobicity of their macrocyclic platforms. There are four main approaches to improving solubility in water and other polar protic solvents: (1) introduction of alcohol and amino groups, (2) introduction of acid groups (carboxyl or sulfo groups), (3) quaternization of tertiary amines by an electrophilic agent, known as the Menshutkin reaction, (4) introduction of ready-made quaternary ammonium fragments. These methods allow for obtaining a permanent positive charge. In combination with heterocyclic fragments, this allows us to expect the formation of complexes with negatively charged substrates of various types, including anions and biomolecules such as proteins and nucleic acids.

In our opinion, this direction is very promising. It combines all the advantages of a non-toxic, stable macrocyclic platform. It also has a positive charge. This increases the solubility of calixarene in water. It also has a heterocyclic fragment. This acts as a binding site. The two most common approaches for obtaining a quaternized nitrogen atom in the substituent of the macrocyclic platform are an attack by an alkylating agent: (a) an aliphatic nitrogen atom in a tertiary amino group and (b) a nitrogen atom in an aromatic heterocycle (usually imidazole or pyridine).

**Scheme 49:**
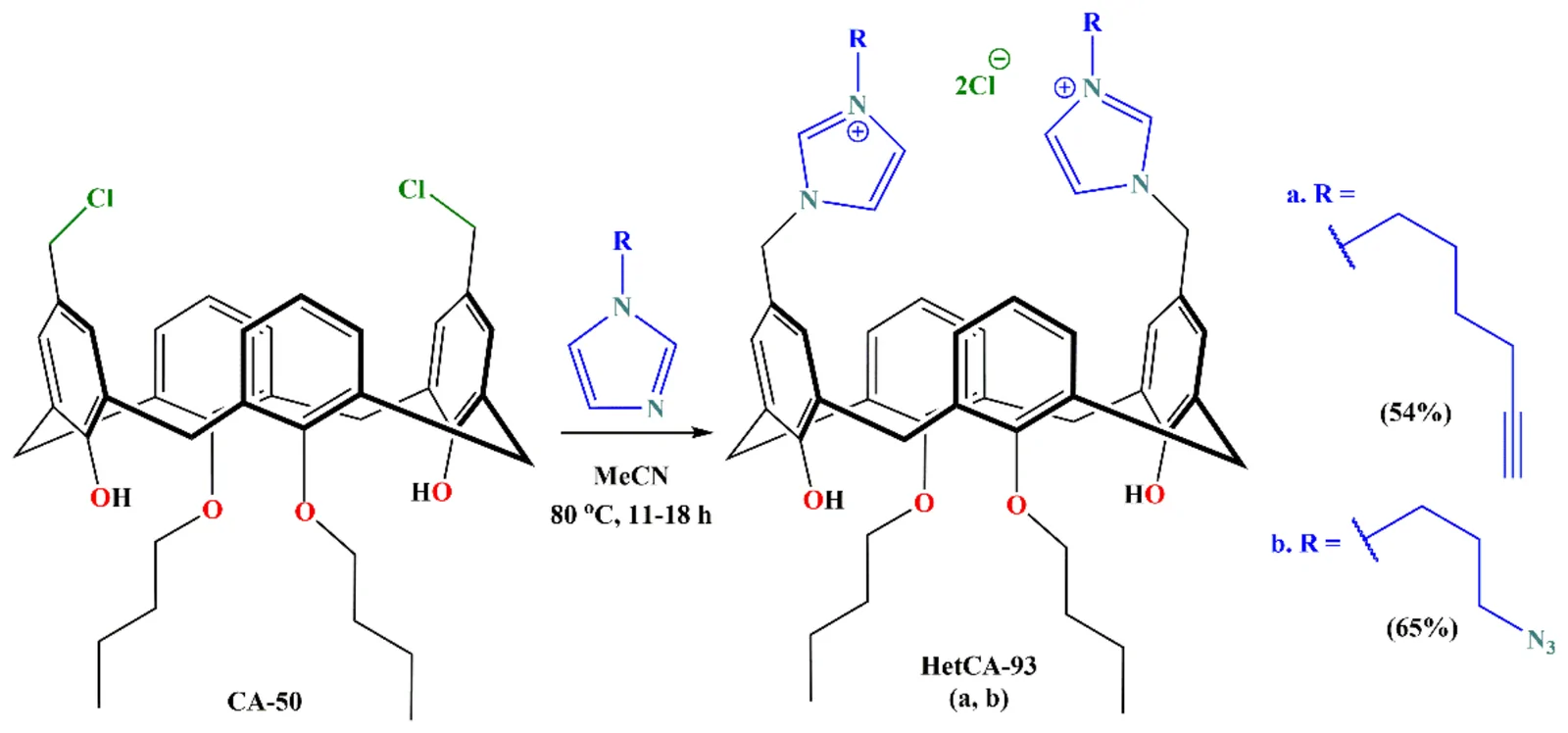
Introduction of alkylimidazole moieties into calix[4]arene **CA-50** [450].

Quaternized heterocyclic derivatives are most commonly used in the production of palladium catalysts or as ligands for constructing supramolecular systems. These are often imidazole [427,428,429,430,431,432,433,434,435,436,437,438,439,440,441,442,443,444,445] or pyridine derivatives [426,427,428,429,430,431,432,433,434,435,436,437,438,439,440,441,442,443,444,445,446,447,448]. Additionally, imidazoline derivatives have been shown to exhibit antibacterial properties [449]. Thus, Burilov et al. obtained diimidazolium derivatives, **HetCA-93,** of calix[4]arene modified with hexynyl and azidopropyl substituents for introduction into a polyazide–alkyl addition reaction to form a polymer that forms complexes with palladium. This polymer can be used as a support for Mizoroki–Heck cross-coupling [450] (Scheme 49).

**Scheme 50:**
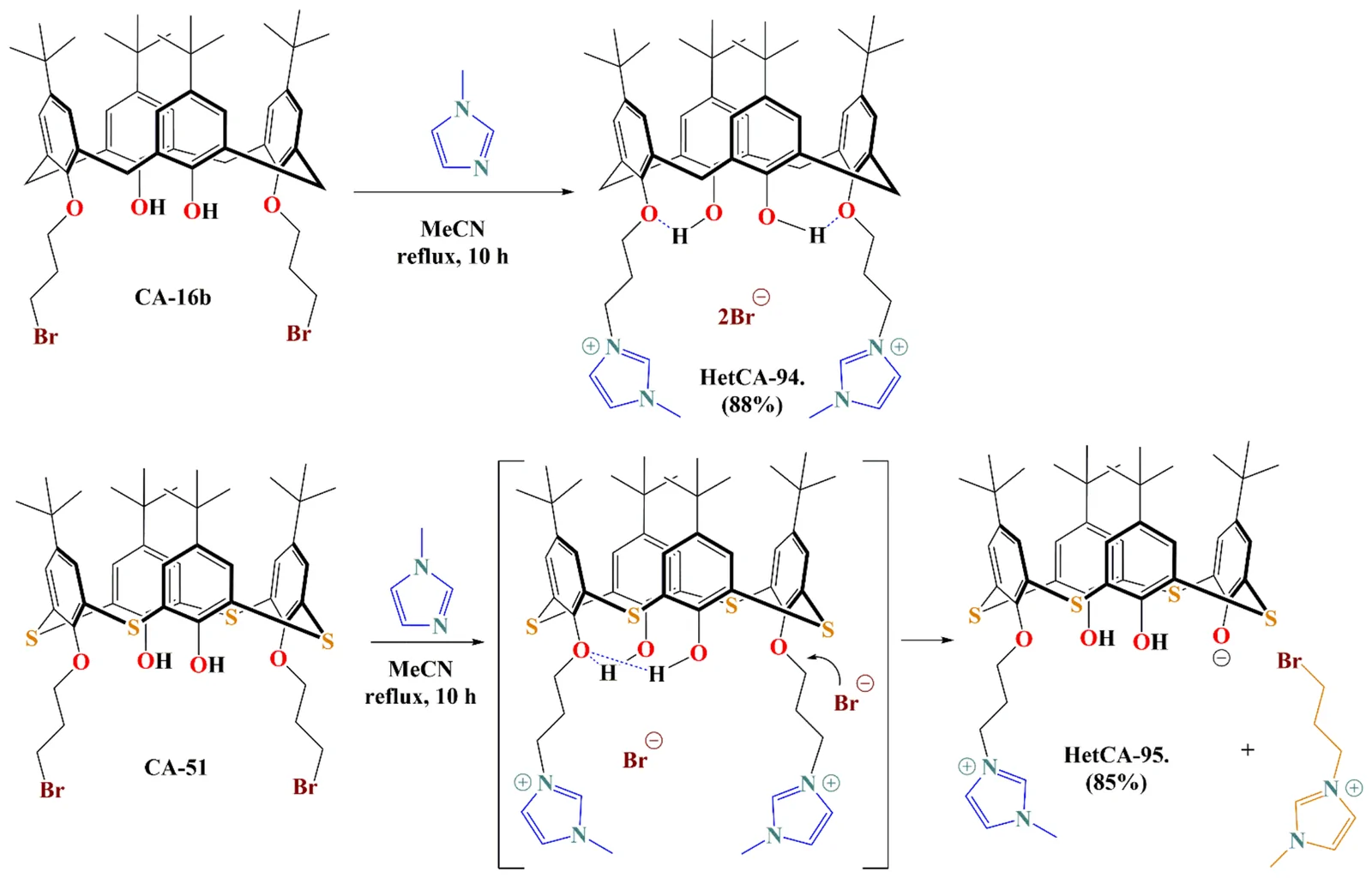
Synthesis of bis-imidazolium calix[4]arene **HetCA-94** and cleavage of the *N*-propylimidazolium fragment to obtain mono-imidazolium thiacalix[4]arene **HetCA-95** [439].

An interesting phenomenon illustrated the difference between classical calixarenes and their sulfur-containing analogs, thiacalixarenes [439]. An attempt to quaternize imidazole fragments for the introduction of two heterocyclic fragments was successful only with classical *p-tert-*butylcalix[4]arene (**HetCA-94**). For its sulfur-containing analog, *p*-*tert*-butylthiacalix[4]arene **CA-51**, the formation of an imidazolium salt was accompanied by elimination of one alkyl group and formation of a monosubstituted derivative **HetCA-95** (Scheme 50). The authors explained this by the peculiarity of the geometry and stabilization of the internal hydrogen bond of two macrocycles. Thus, thiacalixarene is characterized by a “*distorted cone*” conformation. In this conformation, both free hydroxyl groups stabilize only one oxygen of the neighboring substituent via a hydrogen bond. In classical calixarene, however, such stabilization occurs uniformly on both substituents.

**Scheme 51:**
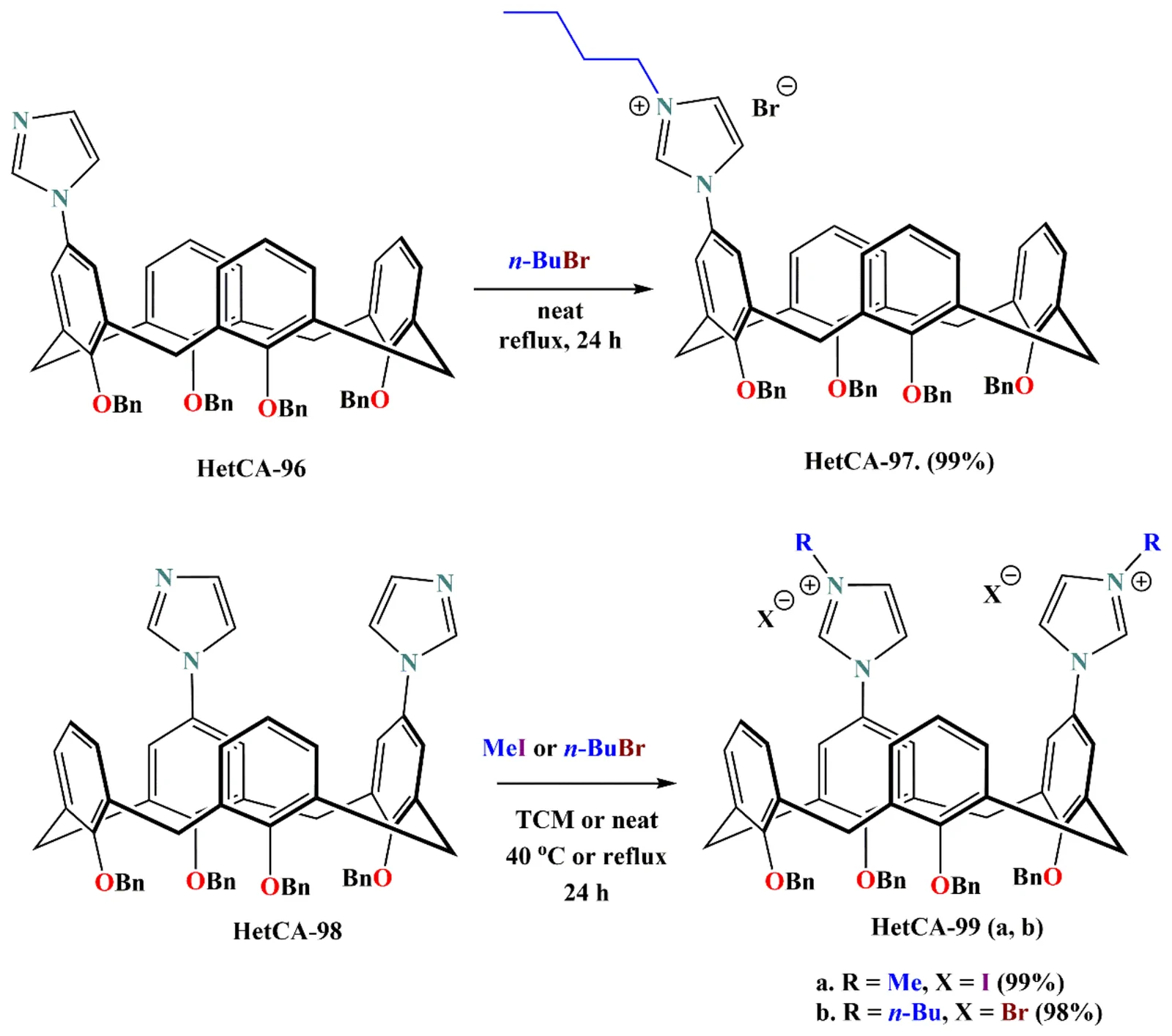
Synthesis of positively charged **HetCA-97** and **HetCA-99** from imidazole macrocyclic derivatives **HetCA-96** and **HetCA-98**, correspondingly [440], via quaternization.

Additionally, there are approaches in which the heterocycle in the macrocyclic structure undergoes quaternization (Scheme 51). Thus, alkylimidazolium derivatives **HetCA-97** and **HetCA-99** were obtained from imidazole derivatives **HetCA-96** and **HetCA-98** in quantitative yields [440].

**Scheme 52:**
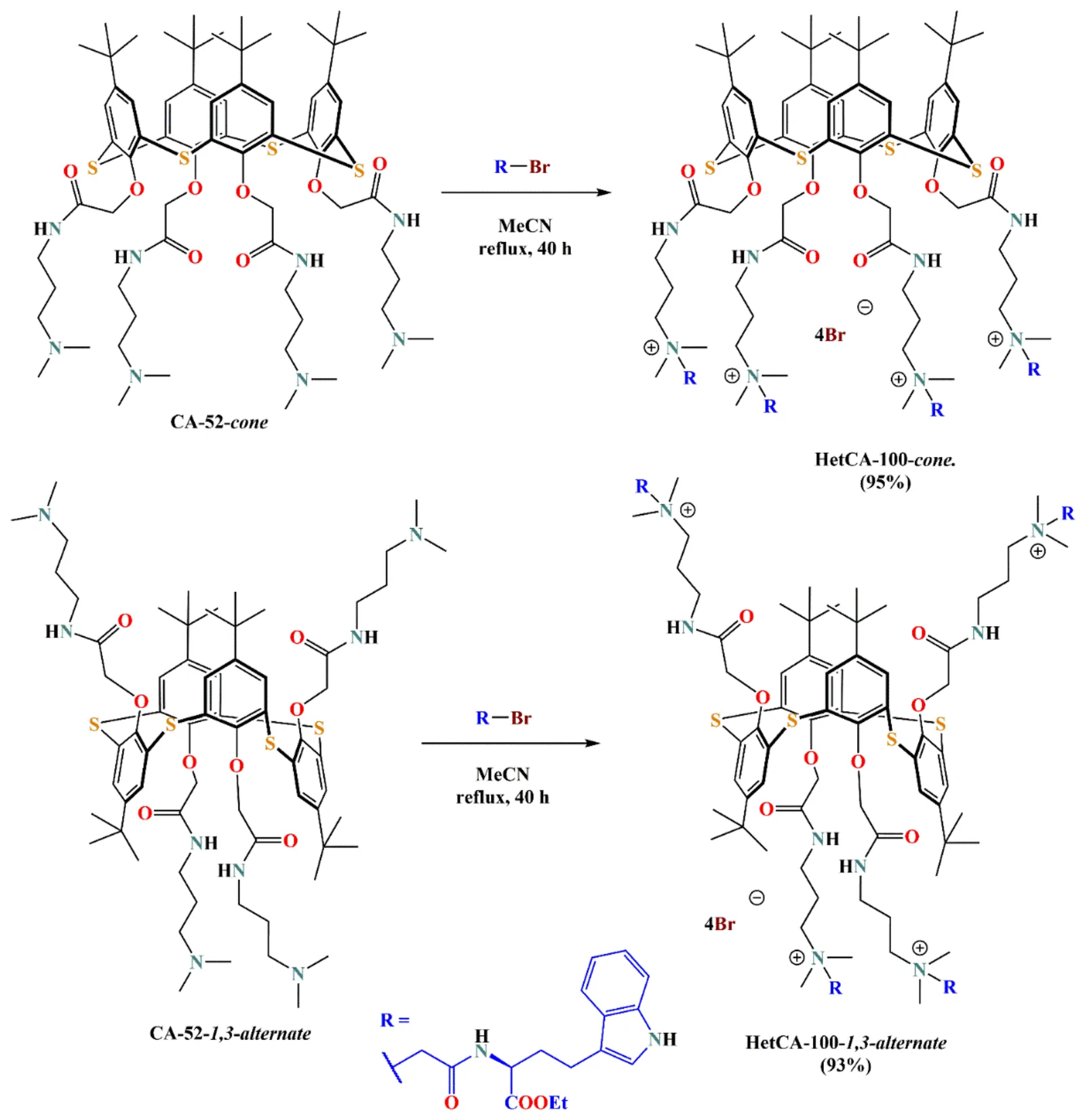
Introduction of *L*-tryptophan fragments into thiacalix[4]arene platform **CA-52** [444] via quaternization.

Additionally, introducing a positive charge into the linker containing an aliphatic tertiary amino group and connecting it to the heterocycle seems very practical [441,442]. Thus, Andreyko et al. modified *L*-tryptophan ethyl ester with bromoacetyl bromide. The resulting alkylating agent was reacted with *p*-*tert*-butylthiacalix[4]arene **CA-52** in *cone* and *1,3-alternate* conformations containing four tertiary amino groups in acetonitrile with almost quantitative yields of **HetCA-100** (Scheme 52) [443,444,451,452]. This method can be applied to a wide variety of heterocyclic substrates.

Thus, quaternization is a fairly effective method for introducing heterocyclic fragments into the composition of thiacalix[4]arenes with good yields. This method has great potential for further development and for obtaining new, diverse structures of heterocyclically modified macrocyclic platforms.

**Scheme 53:**
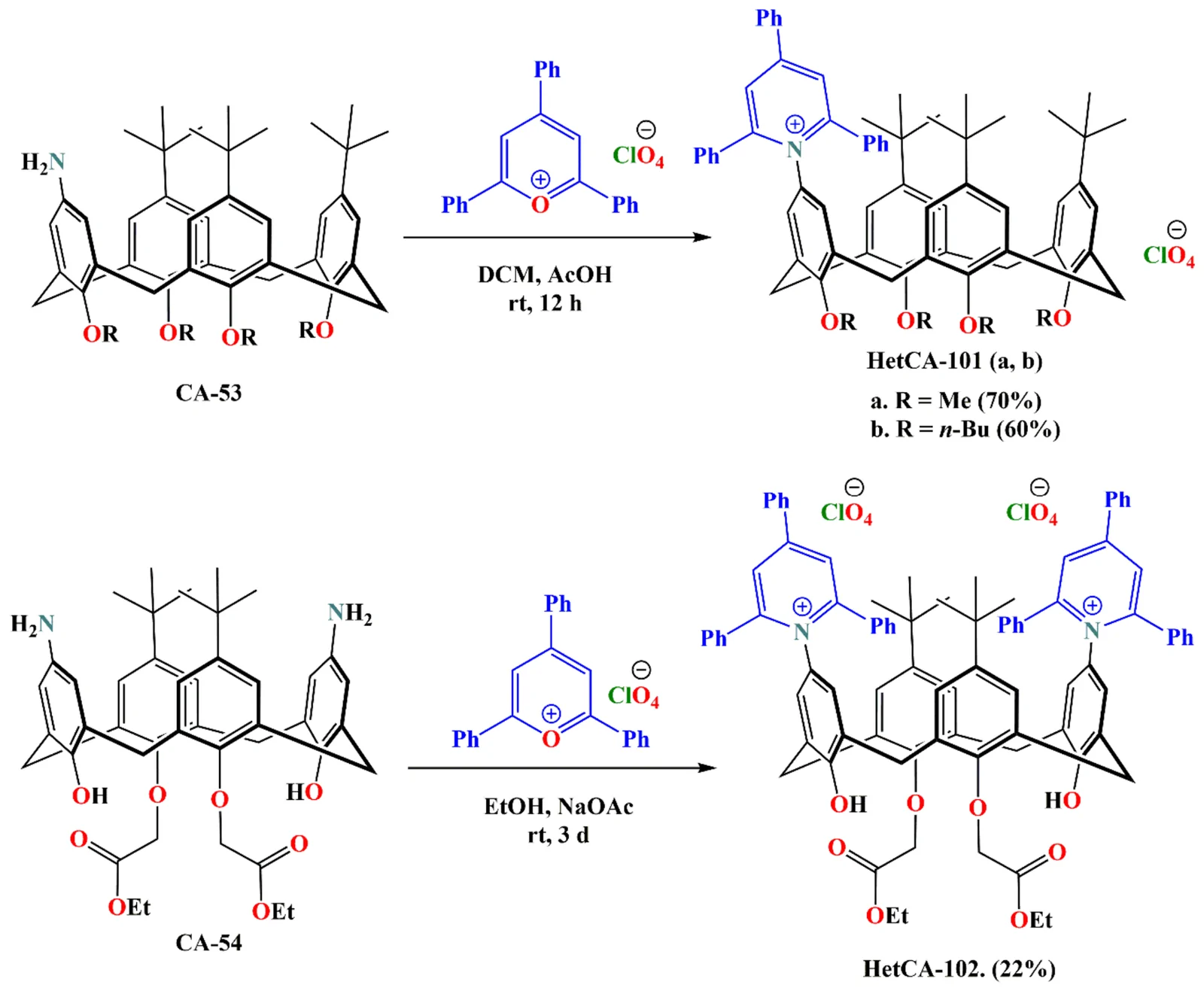
Syntheses of pyridinium **HetCA-101**–**HetCA-102** via pyrylium salts [453].

In addition to the classic Menshutkin reaction, there are other approaches for obtaining quaternized structures. For example, a pyridinium fragment can be introduced along the upper rim of the aminocalixarene using triphenylpyrylium perchlorate [453]. This method has been known for a long time [454], although it is not often found on macrocyclic compounds. Monopyridine derivatives **HetCA-101** were obtained in good yields of 60–70%, while the bis-derivative **HetCA-102** was obtained with a yield of only 22% (Scheme 53).

**Scheme 54:**
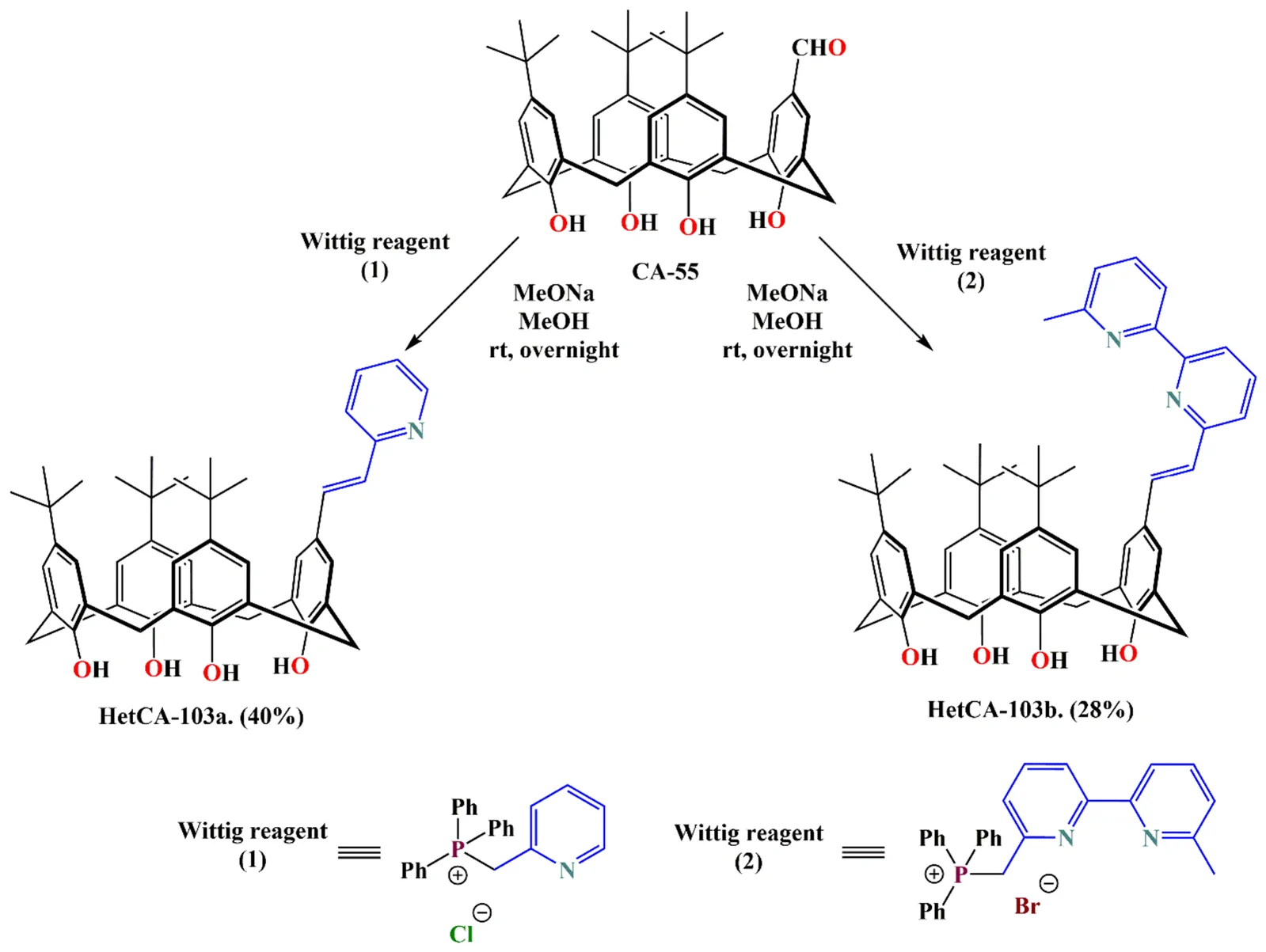
Synthesis of **HetCA-103** from macrocyclic aldehyde **CA-55** [455].

In addition to the above-described methods of introducing heterocyclic fragments into calixarene structures, there are other methods that are not widely used. However, this does not diminish the importance of these protocols, as many of them may soon be recognized. One of the rare but noteworthy approaches to introducing heterocyclic fragments into *meta*-cyclophane is the Wittig reaction [376,455,456]. In the work by Regnouf-de-Vains and Lamartine [455], the yields of calixarenes **HetCA-103** were quite low (28–40%), which the authors explained by the low stability of the “stilbene-like product” itself (Scheme 54).

**Scheme 55:**
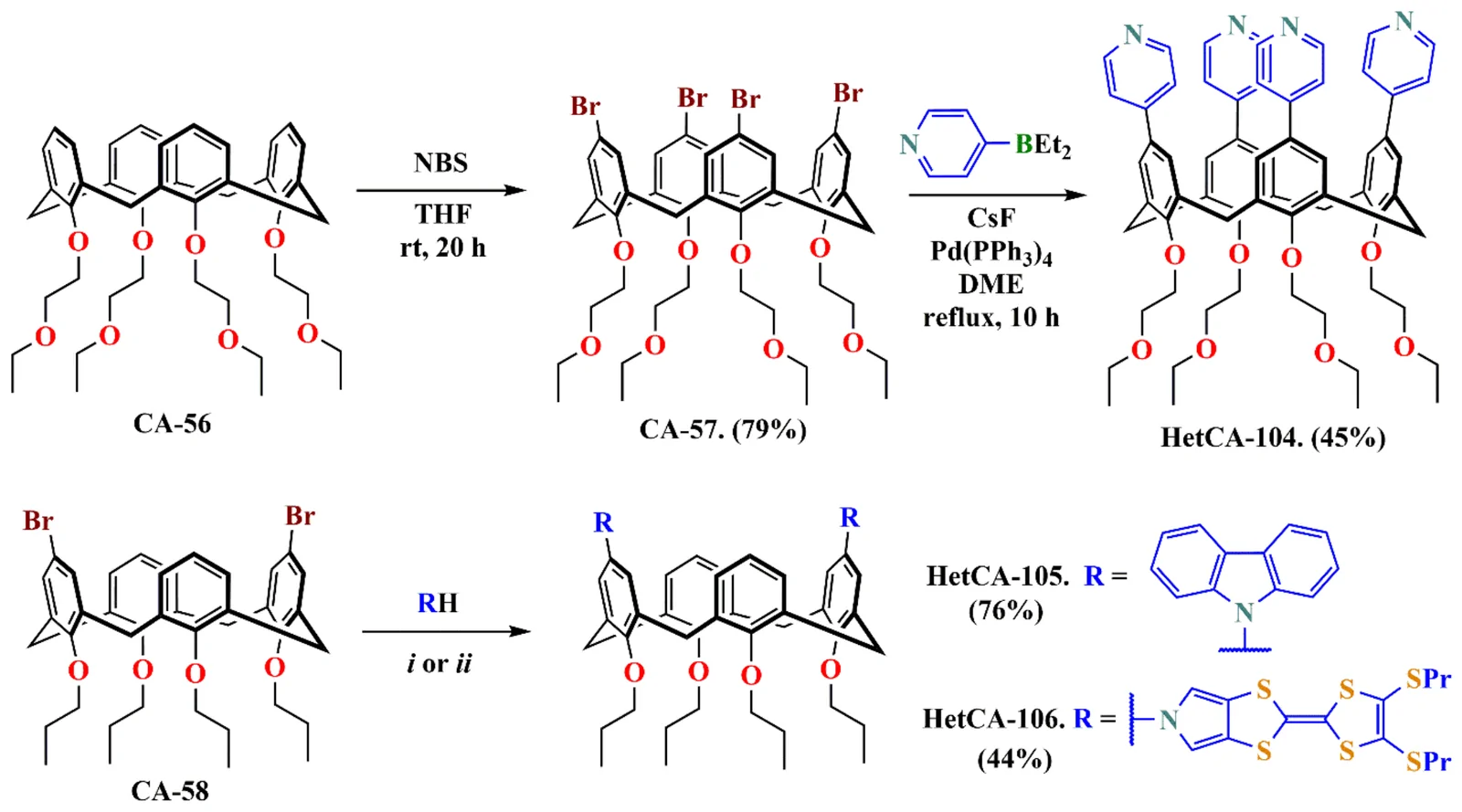
Examples of syntheses of **HetCA-104** via Suzuki reaction [457] and **HetCA-105**—**HetCA-106** via Ullmann reaction [465,466]. Conditions: *i*—Cu_2_O, *s*-collidine, reflux, 30 h; *ii*—CuI, K_3_PO_4_, *trans*-diaminocyclohexane, dioxane.

It is noteworthy that calixarenes can be modified with heterocyclic binding sites via cross-coupling reactions, such as the Suzuki or Sonogashira reactions [379,457,458,459,460,461,462,463,464], or via the Ullmann reaction [465,466]. Thus, the tetrapyridine derivative of calix[4]arene **HetCA-104** was obtained through the reaction of 4-(diethylboranyl)pyridine with the tetrabrominated **CA-57**, yielding 45% (Scheme 55). The Ullmann reaction also allowed the synthesis of **HetCA**s modified at the upper rim with relatively small fragments of carbazole, **HetCA-105** [465], and fused pyrrolotetratiafulvalene, **HetCA-106** [466] (Scheme 55).

**Scheme 56:**
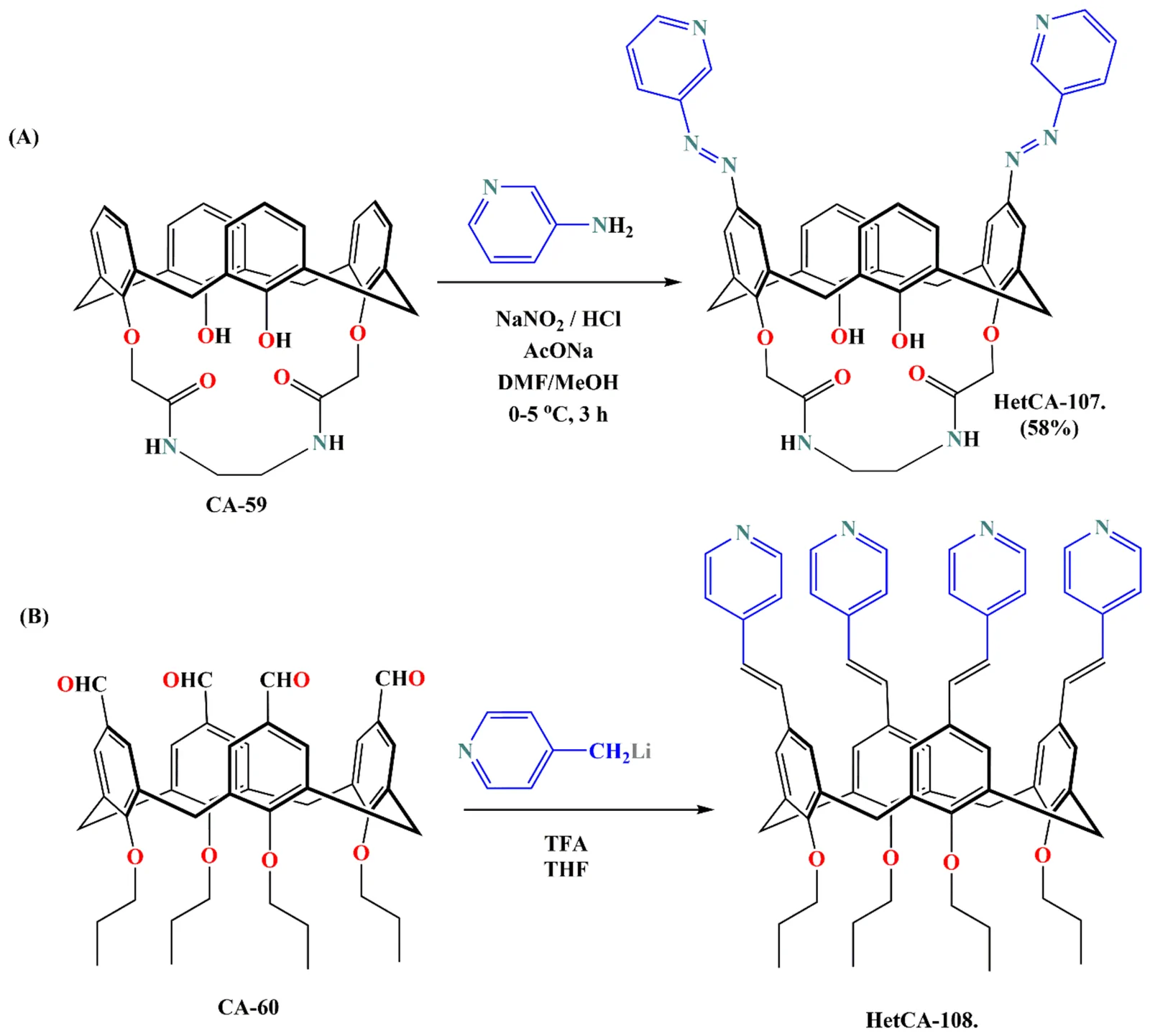
Synthesis of (**A**) **HetCA-107** via mild oxidation azo-coupling [467] and (**B**) **HetCA-108** via the Knövenagel-type reaction [469].

The introduction of heterocyclic fragments into calixarene structures has become popular due to azo compounds, which enable the production of sensitive photographic materials [467,468]. The yields of the bis-azopyridine derivative **HetCA-107** of calix[4]arene were up to 58% [467] (Scheme 56A). There were only a few studies on the synthesis of **HetCA-108** obtained by the Knövenagel reaction [469]. Unfortunately, the authors did not specify the exact conditions and yields of this reaction, which makes a comprehensive analysis impossible (Scheme 56B).

The synthetic approaches discussed in Section 3.1 vary greatly in terms of the structure of the initial macrocyclic substrates and the heterocyclic structures themselves [427,432,433,434,435,437,439,440,441,446,447,470,471,472] (Table S13). Over the past couple of decades, significant progress has been made in **HetCA** chemistry, resulting in the production of a vast number of new compounds with applications in various fields. These approaches can be simple and inexpensive, such as the Menshutkin reaction or most variants of the Williamson synthesis. Alternatively, they can be expensive and require complex catalysts, such as azide–alkyne addition or the Steglich reaction. Regardless, all of these methods allow for the achievement of structures with predetermined properties.

### 3.2. Divergent Approaches

The use of this family of synthetic tools is less common than the convergent synthesis of **HetCA** schemes, which is described in Section 3.1. Firstly, this is due to the limited number of heterocyclic fragments that can be introduced into a macrocyclic structure ab novo. Secondly, the calixarene platform itself requires certain structural and functional preparation to form a heterocyclic moiety within the macrocycle when interacting with another non-heterocyclic structure. This section of our review article classifies compounds based on the structure of the macrocyclic precursor. However, the approaches still vary considerably.

#### 3.2.1. Synthesis of Heterocyclic Fragments in Side Chains

**Scheme 57:**
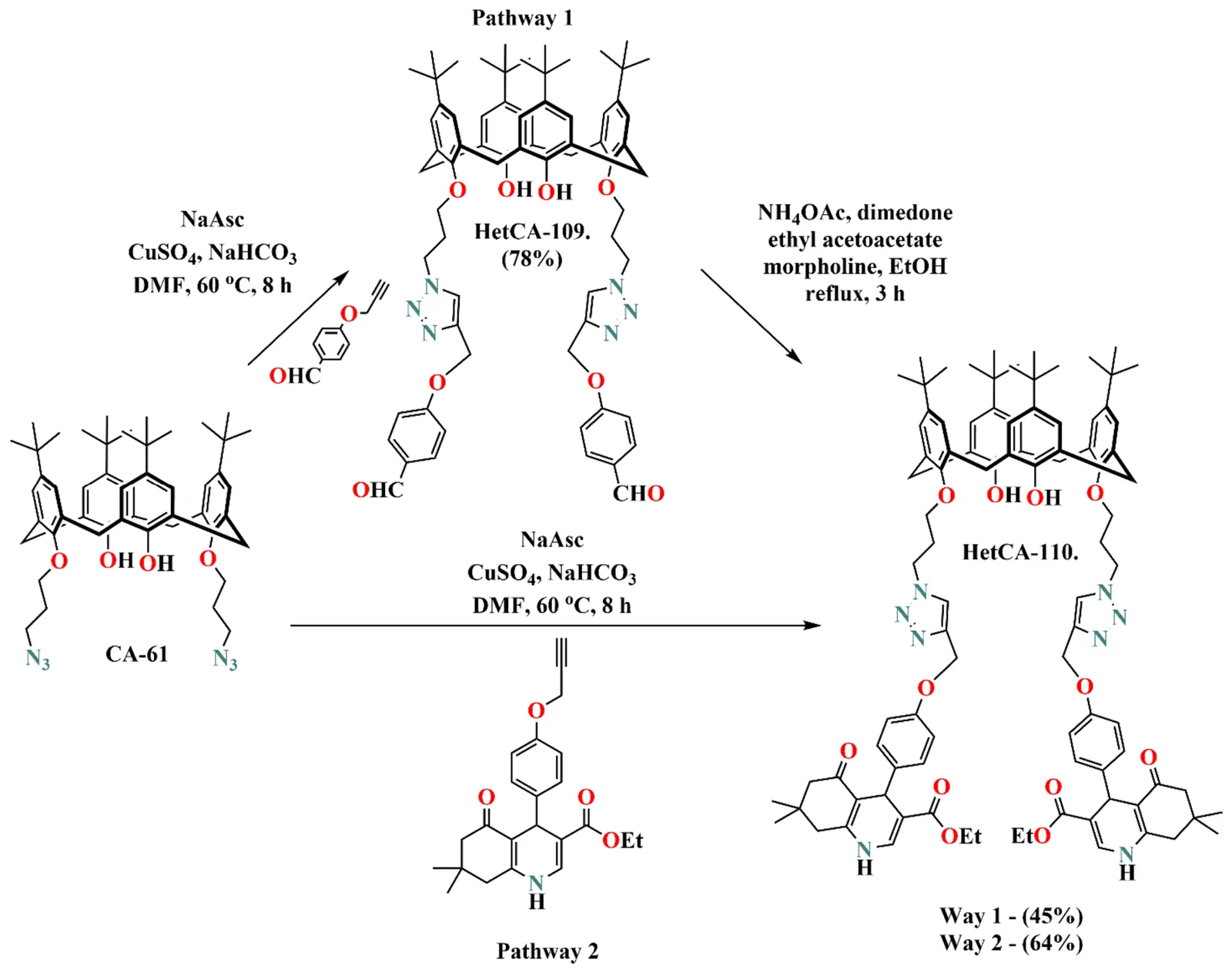
Synthesis of **HetCA-110** via divergent (pathway 1) and convergent (pathway 2) approaches [473].

It should be noted that examples of obtaining the same calixarenes modified with heterocyclic fragments by combining different approaches are unique. For example, Khan et al. from the University of Karachi (Pakistan) reported two approaches for obtaining **HetCA-110** containing triazole and hexahydroquinoline fragments [473] (Scheme 57). According to the first one, the divergent method, an aldehyde was first introduced onto the calixarene **CA-61** via CuAAC to synthesize calixarene **HetCA-109**. Then, **HetCA-109** was condensed with dimedone and acetoacetic ester, yielding **HetCA-110** (45%) (Scheme 57, pathway 1). According to the second, the convergent pathway, the hexahydroquinoline cycle was formed separately from the macrocyclic platform **CA-61**. Then, via the CuAAC reaction of **CA-61** with the heterocyclic propargyl derivative, calixarene **HetCA-110** was obtained with 64% yield (Scheme 57, pathway 2).

Apparently, the most common reaction in the divergent modification of calixarenes is CuAAC. There are already several reviews that specialize in this topic, as well as interesting synthetic works [321,474,475,476,477]; therefore, a detailed analysis of this synthetic branch is beyond the scope of our review paper. In addition, in Section 3.1, we describe CuAAC methods in which the resulting 1,2,3-triazole ring was not the only heterocyclic fragment.

**Scheme 58:**
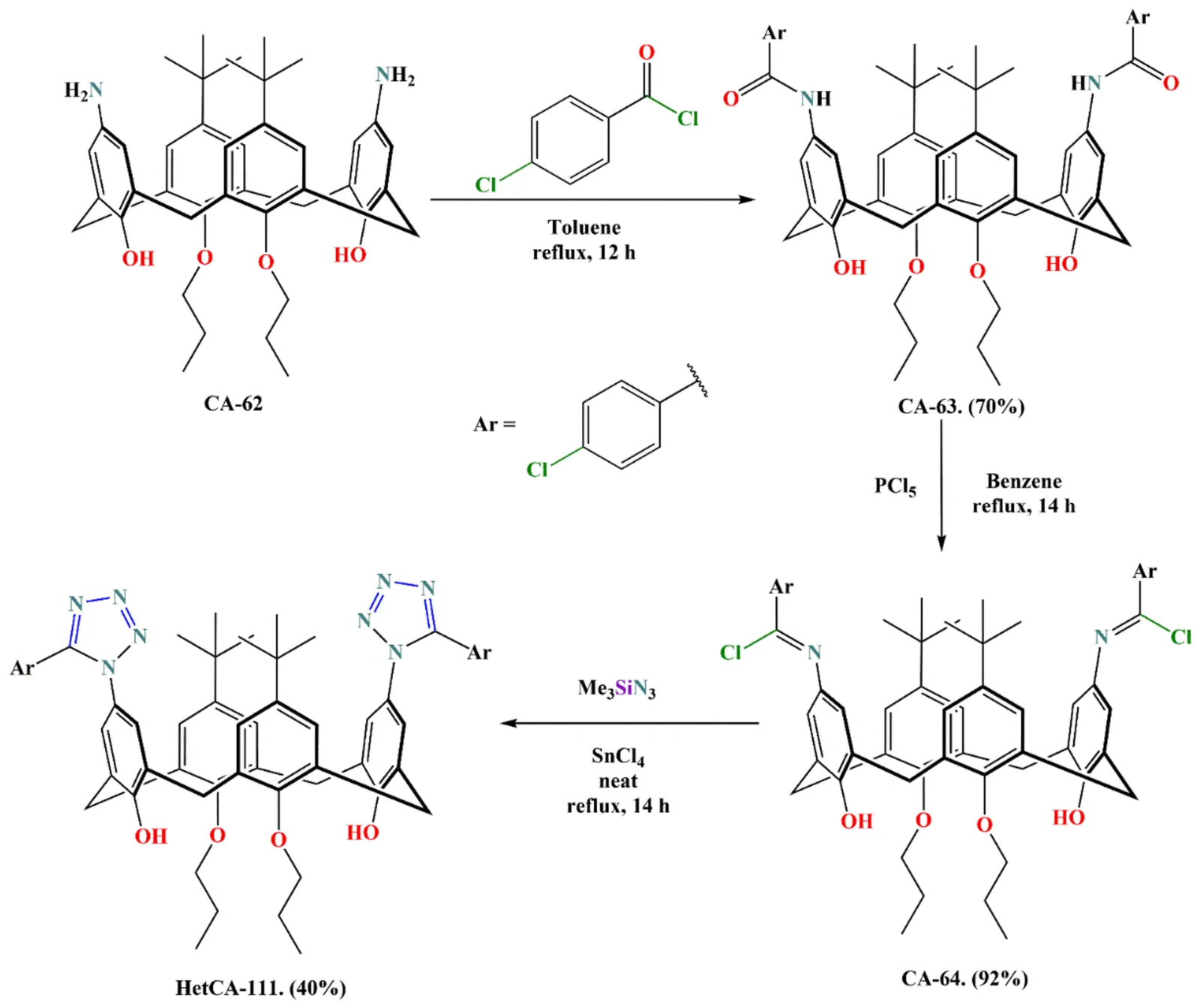
Synthesis of **HetCA-111** from macrocyclic diamine **CA-62**, its *p*-chlorobenzyl derivative **CA-63** and imidochloride derivative **CA-64** [478].

Structures related to triazoles and tetrazoles are quite rare in this field of chemistry. Scientific groups of V. Kalchenko and J. Lipkowski proposed an unusual synthetic approach for obtaining a tetrazole derivative of calix[4]arene **HetCA-111** [478]. The synthetic scheme involves introducing azide fragments into the macrocyclic structure **CA-64** containing an imidochloride functional group (Scheme 58). The yield of the cyclization stage is 40%.

**Scheme 59:**
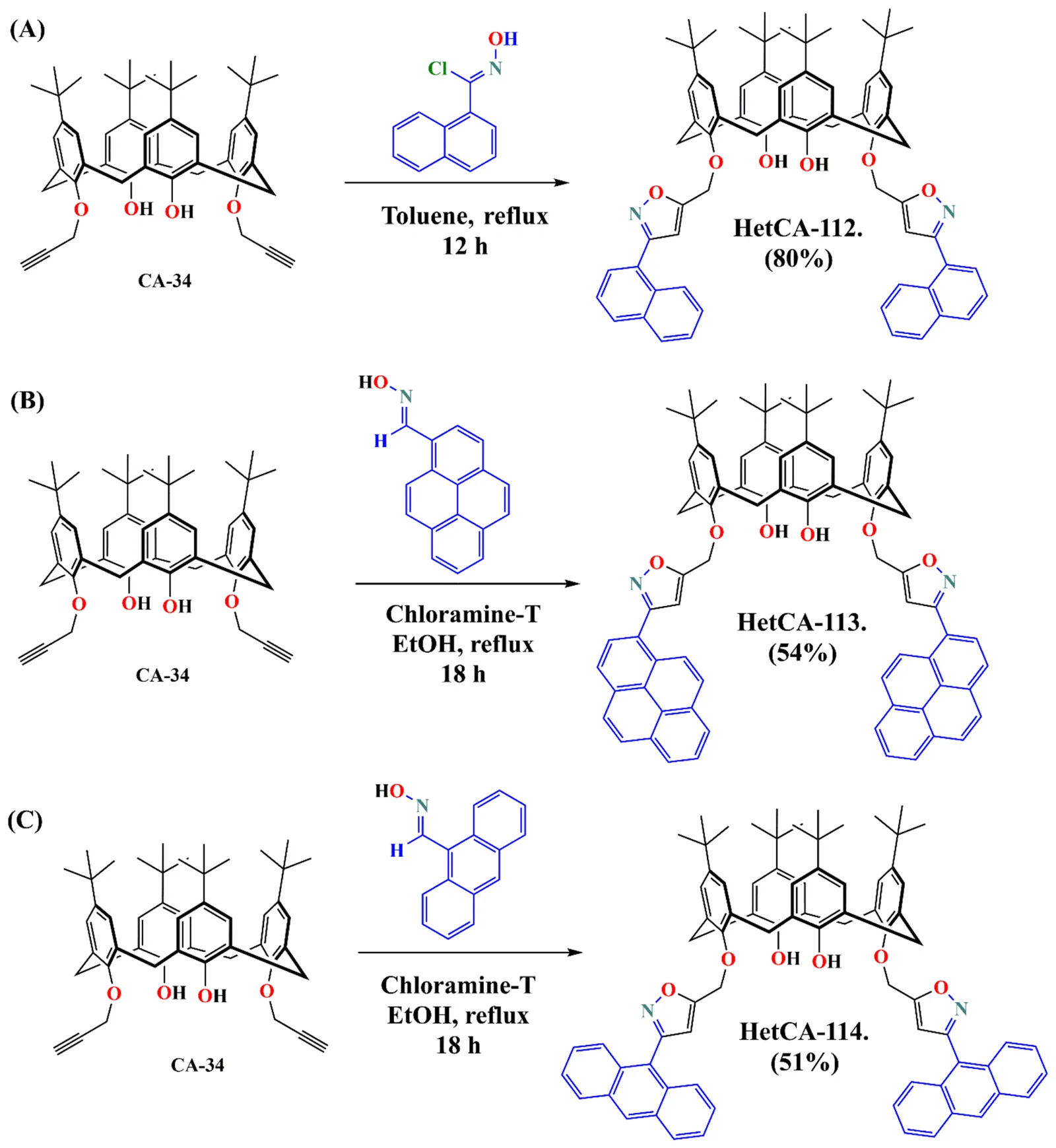
(**A**) Preparation of an isoxazole derivative **HetCA-112** by cycloaddition reaction of 1-naphthyl hydroximoyl chloride with compound **CA-34** [482]. (**B**,**C**) Synthesis of isoxazole **HetCA** derivatives **HetCA-113** and **HetCA-114** from oximes, Chloramine T, and a macrocyclic propargyl derivative **CA-34** [483].

In contrast to the classic CuAAC method, a cycloaddition reaction to a triple bond produces other heterocycles. There were numerous examples of the preparation of 1,2-oxazole (isoxazole) linkers [479,480,481]. A classic example of this approach is the cycloaddition of 1-naphthyl hydroximoyl chloride to calixarene **CA-34** to form **HetCA-112** with a good yield of 80% [482]. However, the synthetic unavailability of hydroxymoyl chlorides may limit the prevalence of this approach. Nevertheless, researchers from Maynooth University (Ireland) demonstrated in 2015 that the necessary reagent can be generated in situ from the corresponding oxime and Chloramine-T. This yields polyaromatic hydrocarbon derivatives **HetCA-113** and **HetCA-114** with moderate yields of 51–54% [483] (Scheme 59).

**Scheme 60:**
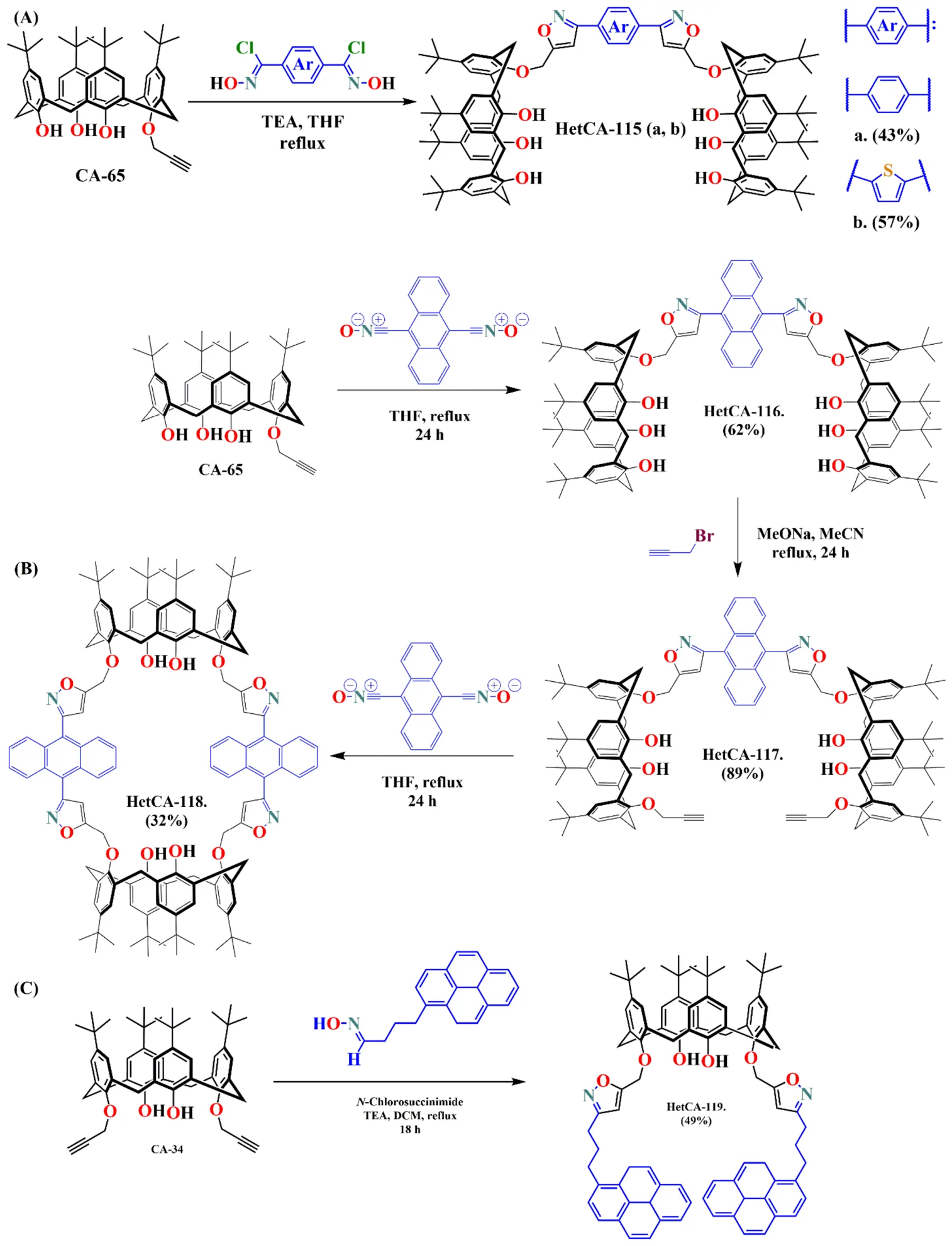
Synthesis of (**A**) bis-calixarene **HetCA-115** containing an isoxazole bridge [484,485]; (**B**) bis-calixarene **HetCA-118** from bis-nitroxide provided by Tsai et al. [484]; (**C**) cycloaddition of an aliphatic oxime to a macrocycle **HetCA-119** in the presence of *N*-chlorosuccinimide as a chlorinating agent [486].

Bis-calix[4]arenes, in which the linker is an isoxazole fragment, are of considerable interest in terms of synthesis, supramolecular properties, and aesthetics. Bis-calixarenes **HetCA-115** that contain an additional benzene or thiophene fragment in the linker between two macrocyclic segments have been synthesized (Scheme 60A) [484,485]. Nitroxides can also be used for the same purposes and yield good results. For example, information is available on the stepwise preparation of a derivative of *p*-*tert*-butylcalix[4]arene and anthracene-9,10-bis-(carbonitroxide) **HetCA-116**–**HetCA-118** (Scheme 60B) [484]. Additionally, aliphatic derivatives can be introduced into cycloaddition reactions alongside aromatic derivatives of nitriles and oximes. Thus, in 2019, two oxime equivalents obtained from 1-pyrenebutanal were reacted with calixarene **CA-65** to produce a bis-derivative **HetCA-119** containing two isoxazole fragments [486].

**Scheme 61:**
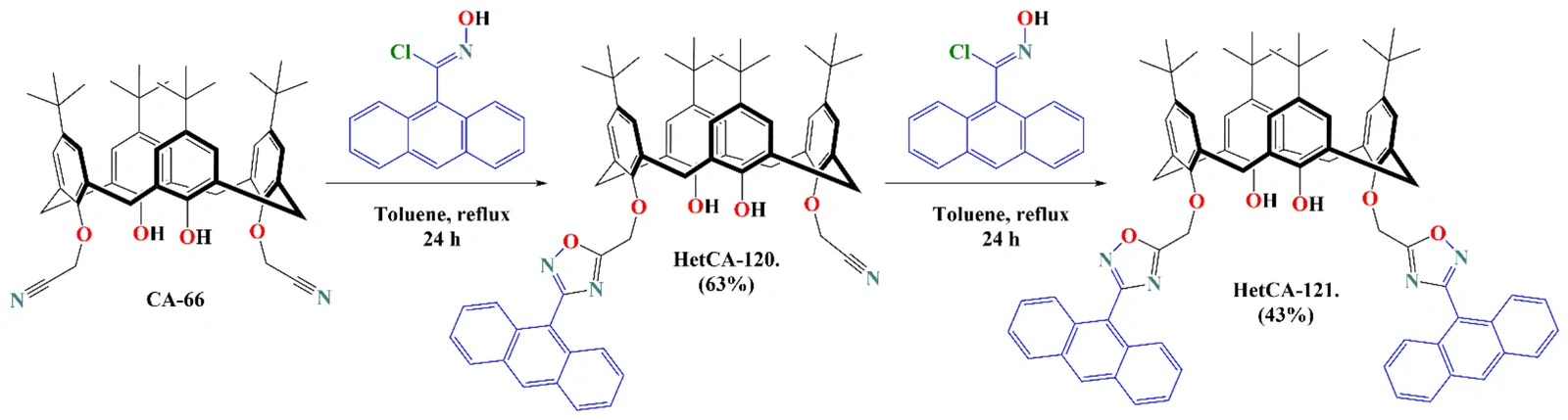
Cycloaddition of hydroximoyl chloride to a macrocyclic bis-nitrile **CA-66** [487].

A unique variation in this cycloaddition method involves the bis-nitrile **CA-66**, which was successfully developed in 2015 by V. Kovalev from Moscow University (Russia) in collaboration with colleagues from Taiwan [487]. Two molecules of 1-anthracene hydroxymoyl chloride were introduced into the macrocycle in two stages, forming 1,2,4-oxadiazole-based **HetCA-120** and **HetCA-121** with yields of 63% and 43% at each stage, respectively (Scheme 61).

Using multiple bonds in calixarene substituent structures is a mainstream approach to divergent synthesis of heterocyclic macrocycle derivatives. Thus, in addition to the synthetic schemes described above, similar to CuAAC, which are used to introduce substituents of various natures, in which the heterocycle is formed rather as a side structure (albeit a very important one, acting, among other things, as a pharmacophore [488] and as a coordinating center for metal cations [489]), there are also approaches in which the creation of a heterocycle is the main focus.

**Scheme 62:**
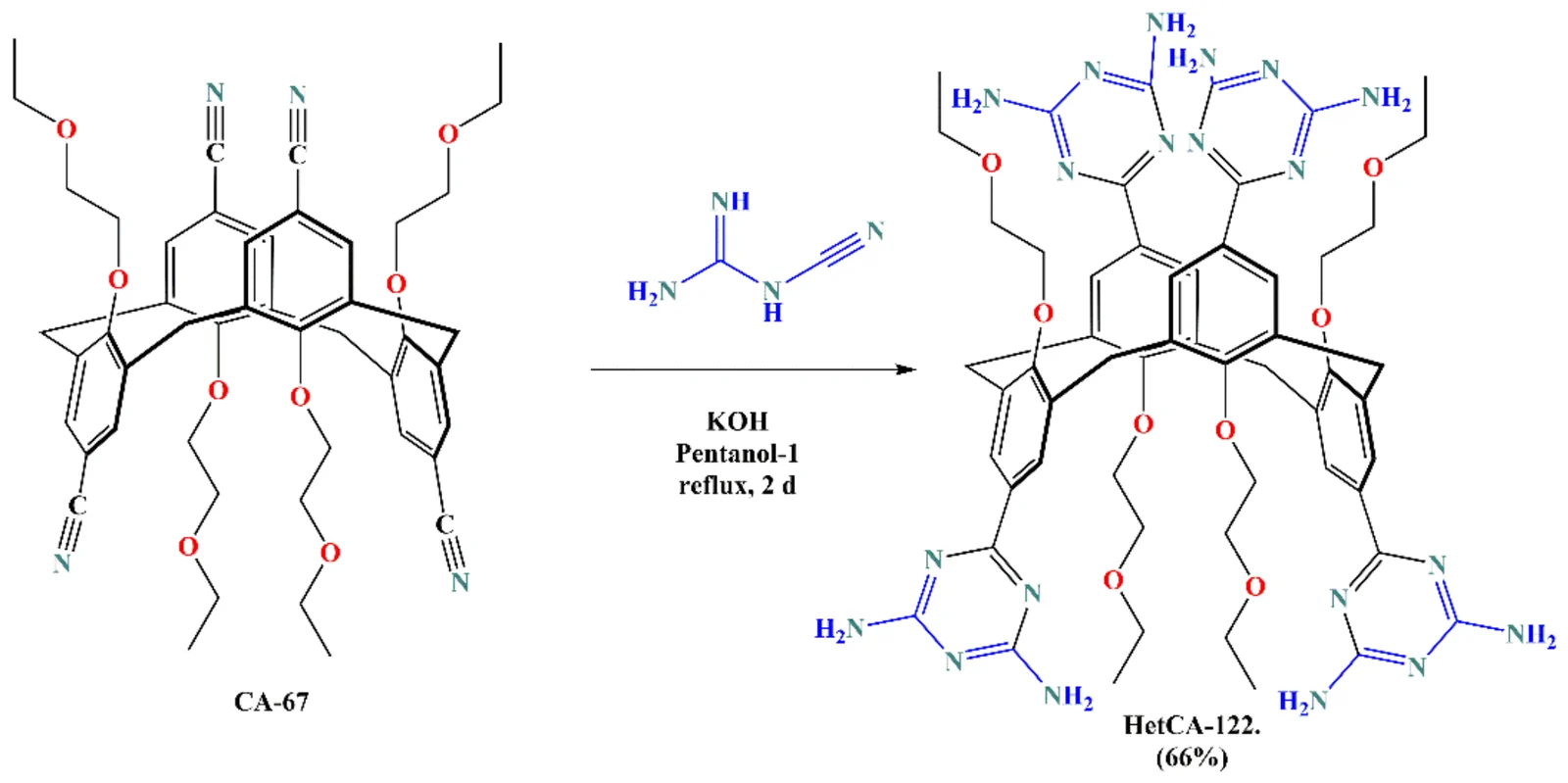
Synthesis of **HetCA-122** [490].

The divergent approach allows for the creation of heterocyclic structures of various natures. For example, O. Barton and his co-authors from Bielefeld University (Germany) proposed an unusual method for obtaining triazine macrocyclic derivative **HetCA-122** (Scheme 62) [490]. During several reactions, the upper rim of the calix[4]arene was modified with nitrile fragments, which reacted with cyanoguanidine in the presence of a strong base in pentanol-1, resulting in products containing two and four triazine fragments, with yields of 23% and 66%, respectively.

**Scheme 63:**
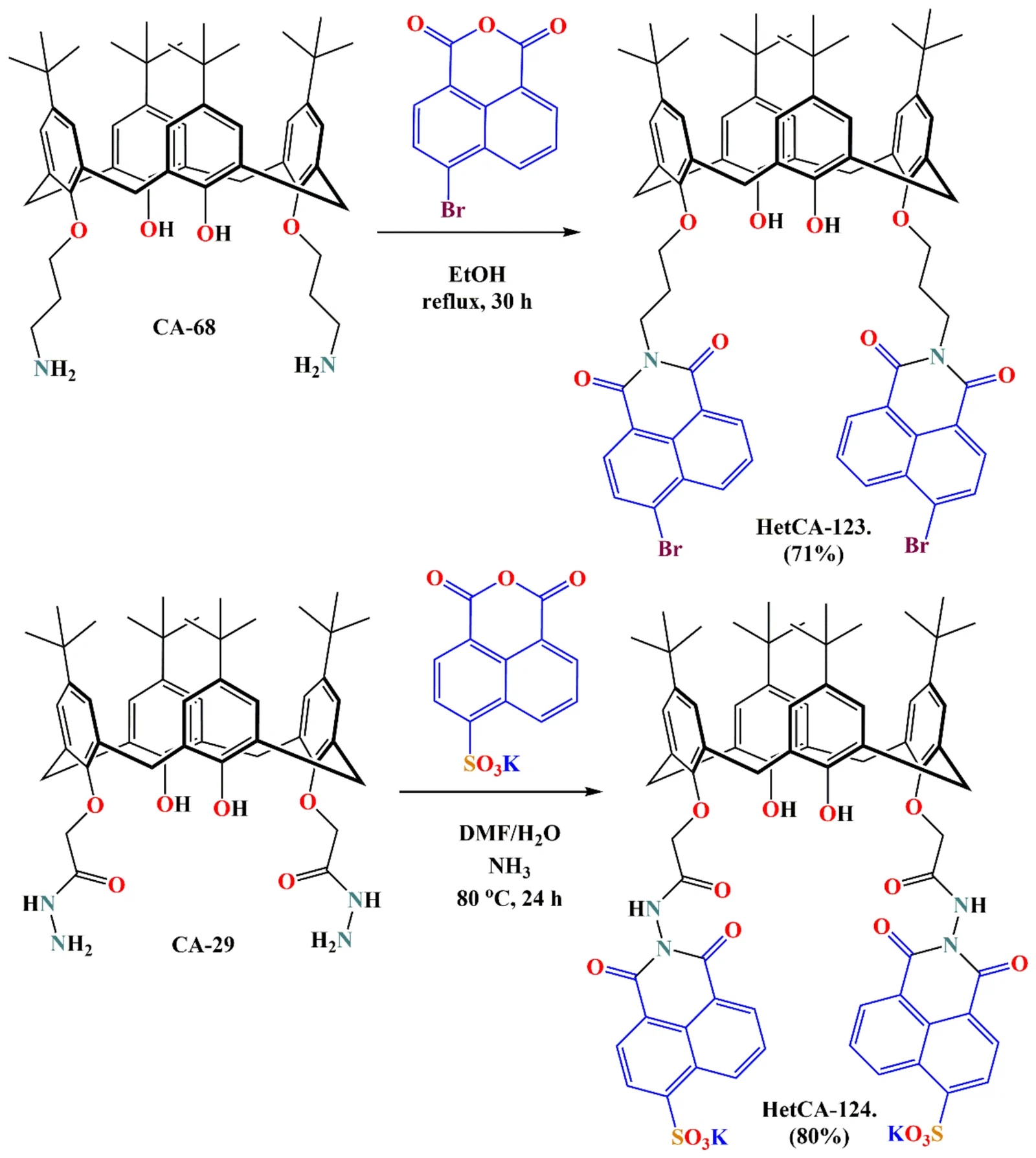
Synthesis of **HetCA-123** and **HetCA-124** [491,496].

A very simple method for introducing heterocycles is the interaction of amino groups of calixarenes with cyclic anhydrides of dibasic acids; for example, the reaction of a macrocyclic amine leads to the formation of naphthalimide [491,492,493,494] or hydrazide [495,496] with 1,8-naphthalene anhydride derivatives to produce macrocycles **HetCA-123** and **HetCA-124** (Scheme 63).

**Scheme 64:**
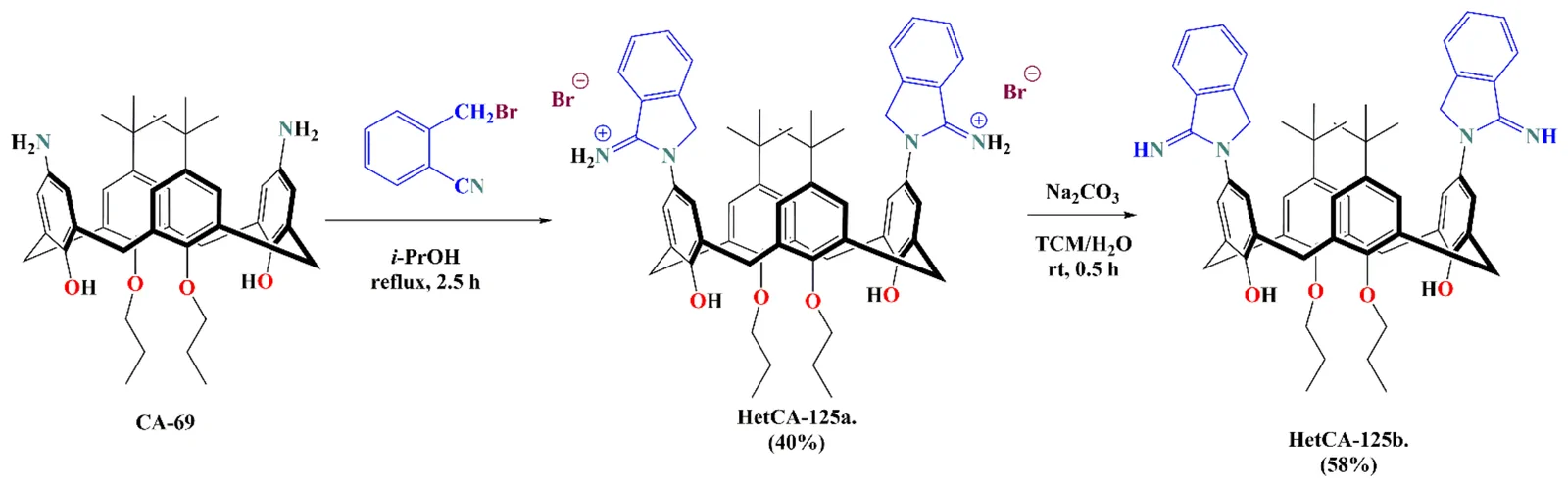
Synthesis of isoindole derivative **HetCA-125** [497].

Cyclization reaction allowed to synthesize isoindole derivative **HetCA-125** in moderate yields [497] (Scheme 64). For this purpose, aminocalix[4]arene **CA-69** is taken and exposed to 2-(bromomethyl)benzonitrile upon heating. The resulting charged derivative is then further treated with a base to obtain target **HetCA-125**.

**Scheme 65:**
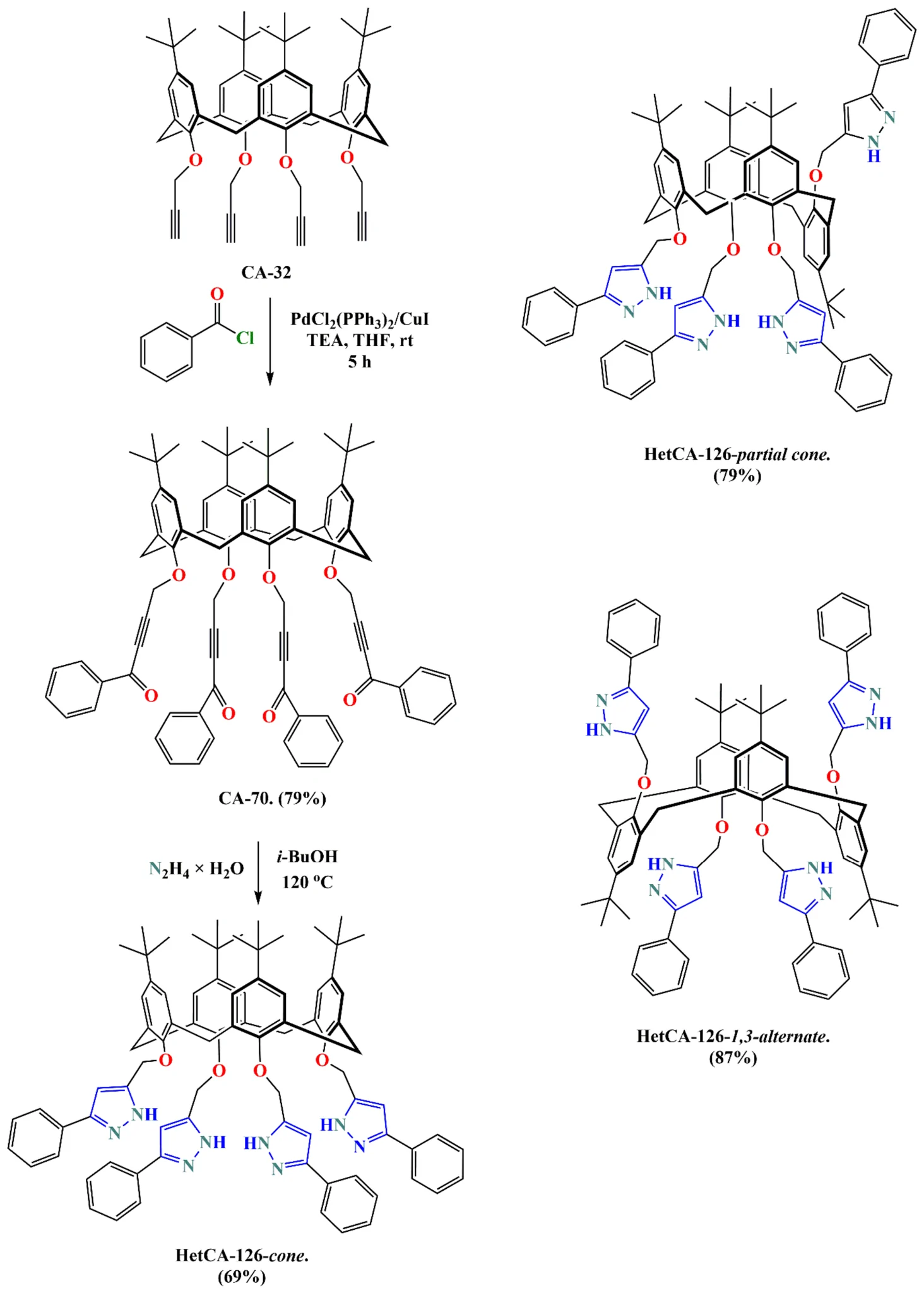
Pericyclic reaction for the synthesis of tetrapyrazole derivatives **HetCA-126** [498].

In 2018, a method for the divergent heterocyclic functionalization of *p*-*tert*-butylcalix[4]arenes based on a two-step synthesis was proposed by Muravev et al. [498] (Scheme 65). The first step of which involves introducing benzoyl chloride into the structure of the tetrakis-propargyl derivatives of calixarenes **CA-32** via the Glaser reaction. This stage produced macrocyclic derivatives **CA-70** containing α-ketoacetylene fragments with yields ranging from 79 to 91%. Derivatives **CA-70** then reacted with hydrazine hydrate in isobutyl alcohol at refluxing temperature in the next iteration, yielding pyrazole derivatives **HetCA-126** ranging from 69 to 87%.

**Scheme 66:**
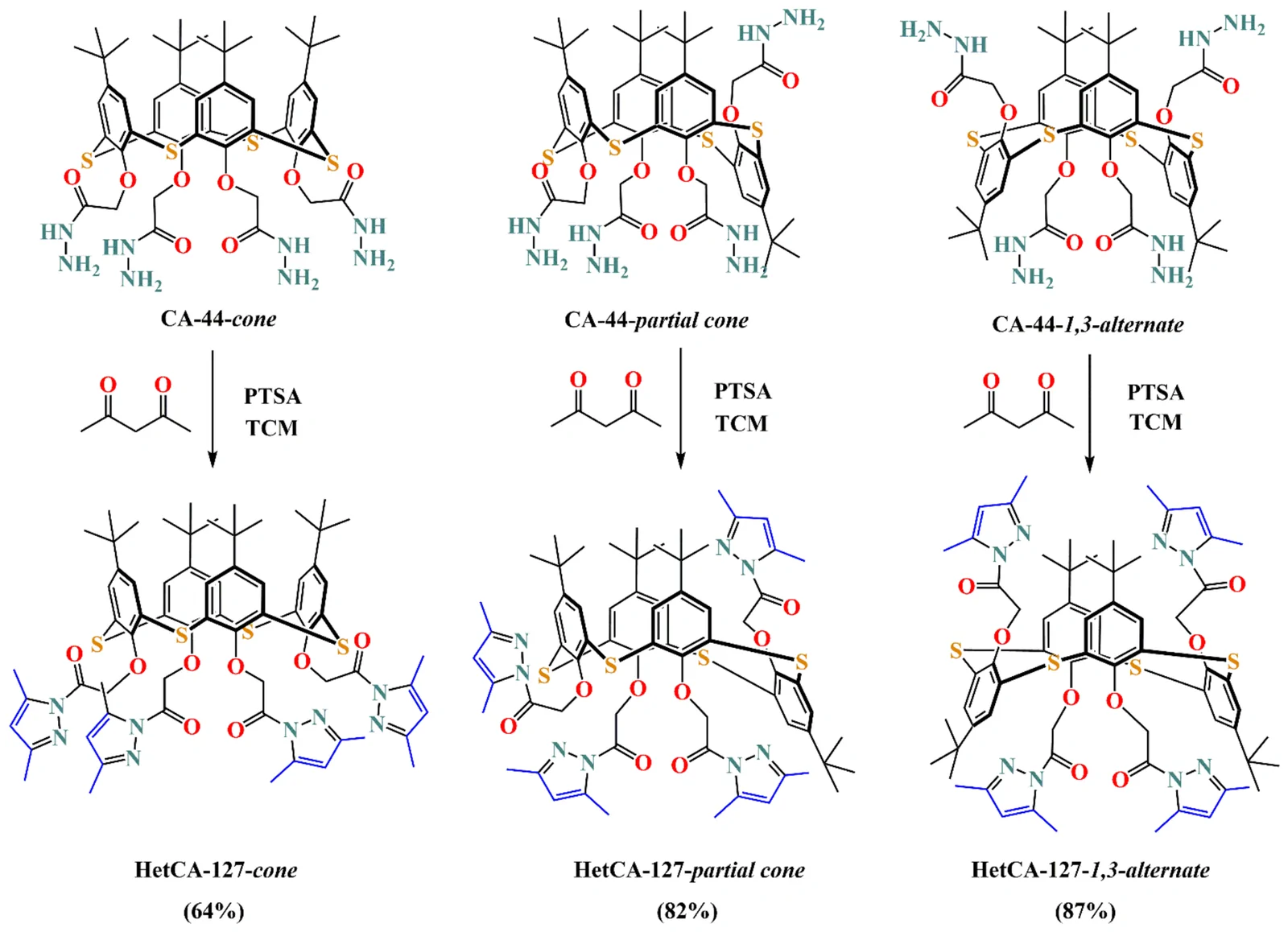
Synthesis of 3,5-dimethylpyrazole derivatives of **HetCA-127** in three stereoisomeric forms [499].

In 2024, a completely different method was established for creating pyrazole fragments on three stereoisomeric forms of *p*-*tert*-butylthiacalix[4]arene (Scheme 66) [499]. Tetrahydrazide thiacalixarene **CA-44** was used as a starting compound, which was reacted with acetylacetone in the presence of *p*-toluenesulfonic acid as a catalyst, resulting in pyrazole derivatives **HetCA-127** with 64–87% yields.

**Scheme 67:**
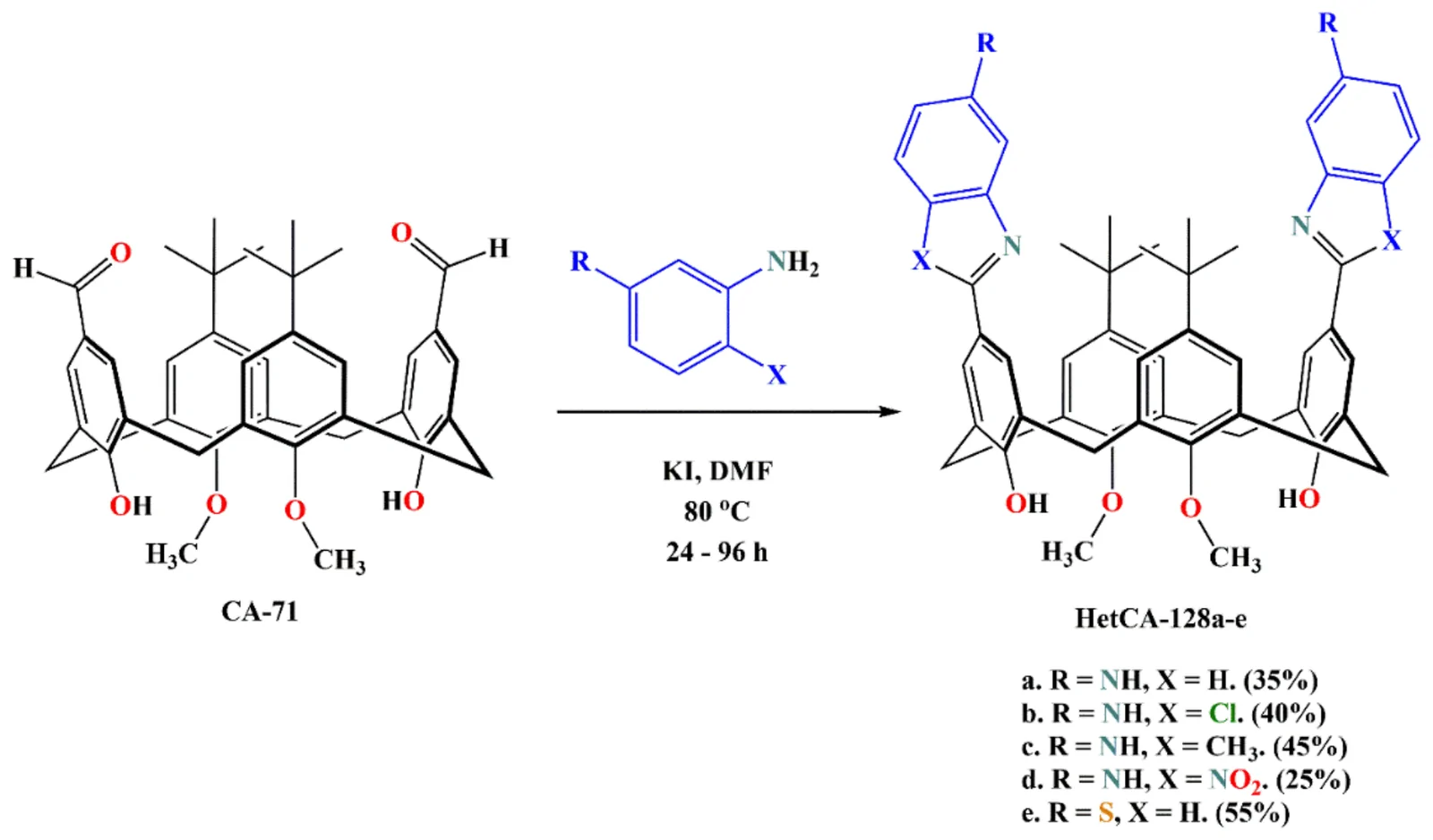
Modification of calix[4]arene aldehyde **CA-71** with benzimidazole and benzothiazole fragments along the upper rim [506].

Calix[4]arenes modified with carbonyl functional groups can successfully create heterocyclic substituents in the pyrazole series and beyond [500,501,502,503,504,505]. For example, Ozkan et al. obtained calix[4]arenes **HetCA-128** modified with benzimidazole and benzothiazole fragments on the upper rim [506] (Scheme 67).

**Scheme 68:**
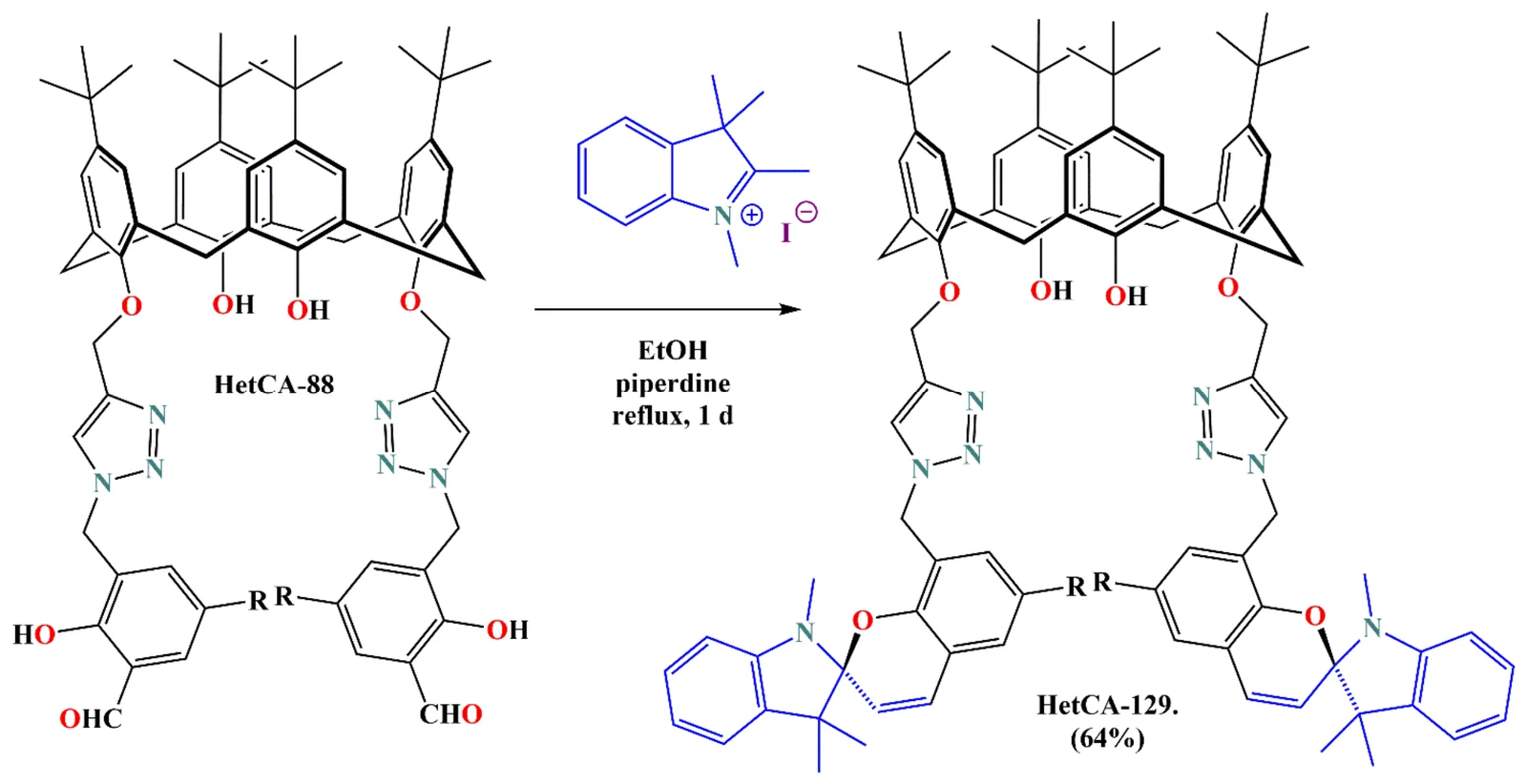
Synthesis of bis-indolinaspyrone derivative **HetCA-129** [507].

Of particular interest is the possibility of introducing labile heterocyclic systems that open or close under certain conditions, such as a change in pH or the presence of specific ions. For example, the synthesis of calixarene **HetCA-129** as a sensor for copper(II) cations was published in 2019 by Nag et al. During the final stage of the synthesis process, the *N*-methylindolium compound interacts with the *o*-hydroxybenzaldehyde moieties of the calixarene platform. This interaction results in the formation of a bis-indolinaspyrone derivative **HetCA-129** with a yield of 64% (Scheme 68) [507]. The reaction takes place within 24 h in boiling EtOH.

The divergent strategy of synthesizing macrocyclic heterocyclic derivatives has found application in porphyrin derivatives. The chemistry of porphyrins and phthalocyanines is one of the fastest-growing areas of science. These macrocyclic derivatives exhibit a wide range of biologically useful properties, allowing them to be used in theranostics [508,509,510,511,512,513,514]. Combining two macrocyclic platforms, i.e., porphyrin and calixarene, produced structures with the beneficial properties of both. Previous sections discussed the introduction of porphyrin fragments using a convergent approach to form an intact macrocycle. This section discusses divergent methods for obtaining porphyrin derivatives of calixarenes, a topic extensively covered in the literature.

**Scheme 69:**
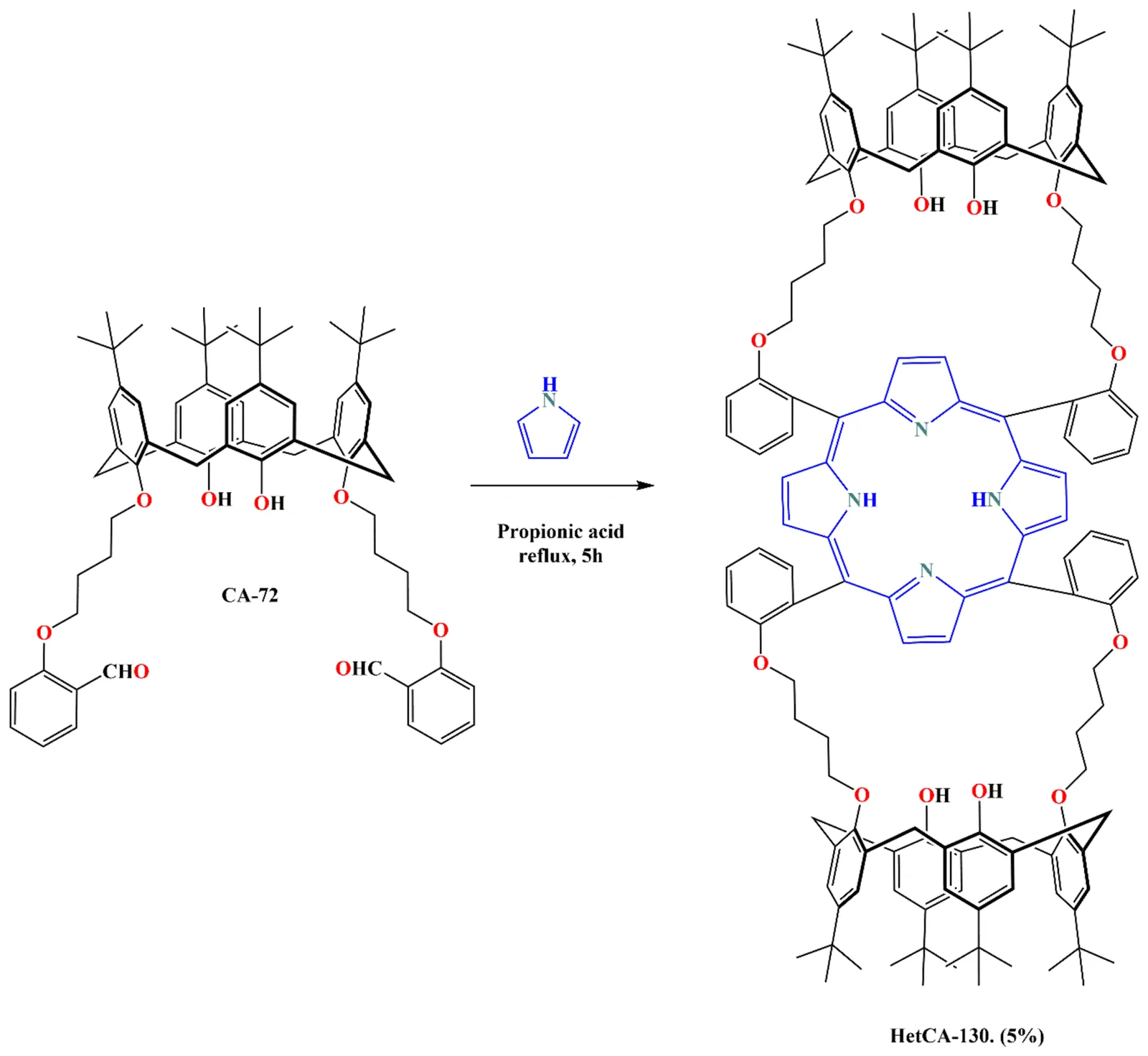
Synthesis of bis-calixarene **HetCA-130** via condensation of macrocyclic dialdehyde **CA-72** and pyrrole [278].

The aldehyde moiety does not necessarily have to be directly conjugated to the calixarene skeleton. Thus, in an article by Rudkevich et al. [278], porphyrin–calixarene derivative **HetCA-130** was obtained with a yield of 5% starting from aldehyde **CA-72** (Scheme 69). The arbitrary position of the aldehyde group opens up great possibilities for obtaining porphyrin **HetCA**s of various natures.

**Scheme 70:**
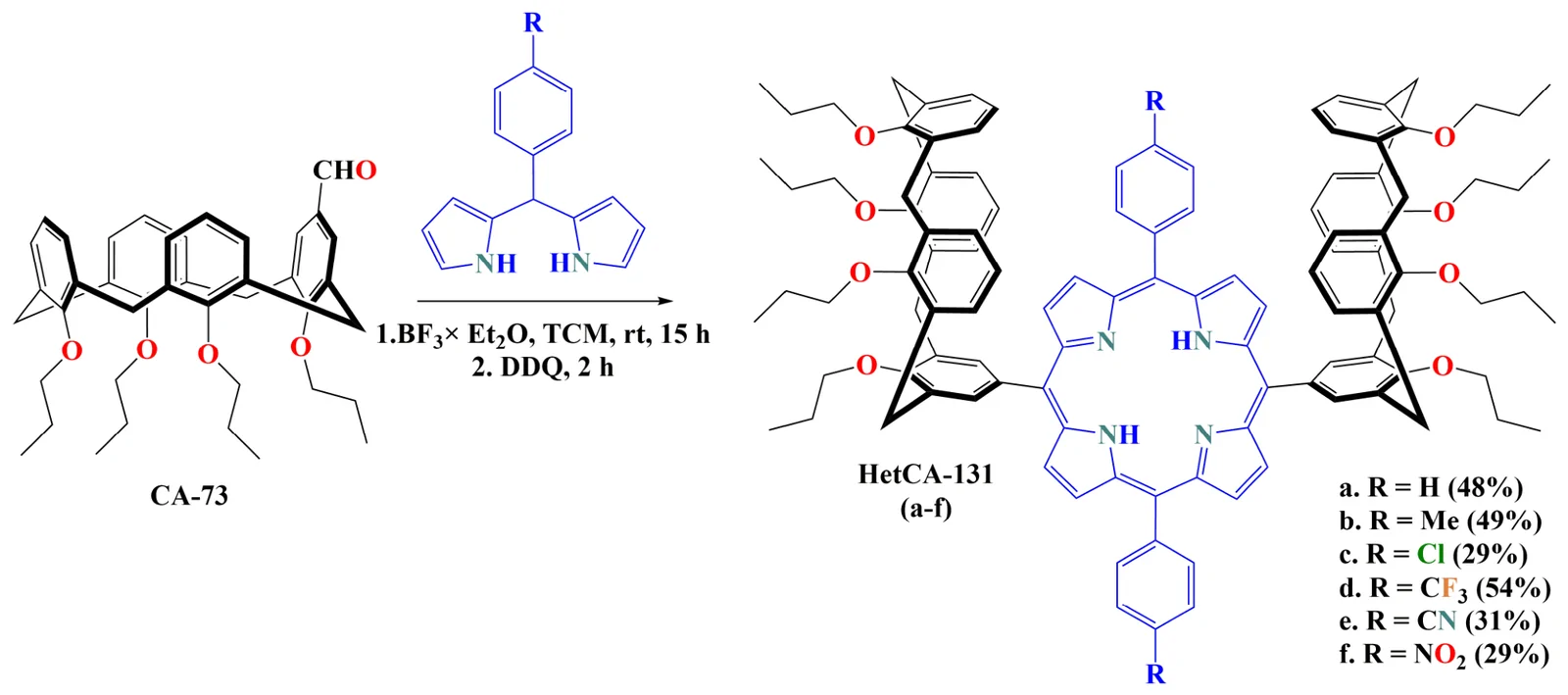
Synthesis of bis-calixarene **HetCA-131** [515].

Subsequent studies avoided using free pyrrole, instead preferring bis-pyrrole derivatives. Typically, a bis-pyrrole fragment serves as the structural foundation for porphyrin assembly and can be incorporated into the calixarene composition in various ways. One noteworthy method was used by Dudič et al. [515]. Macrocyclic aldehyde **CA-73** was reacted with bis-pyrrole derivative **HetCA-131**, followed by oxidation with 2,3-dichloro-5,6-dicyano-1,4-benzoquinone (DDQ), resulting in bis-calixarenes with yields of 31–54%, two parts of which are linked by a porphyrin fragment modified with various substituents (Scheme 70). Similar syntheses are also described in the works [516,517,518,519,520].

**Scheme 71:**
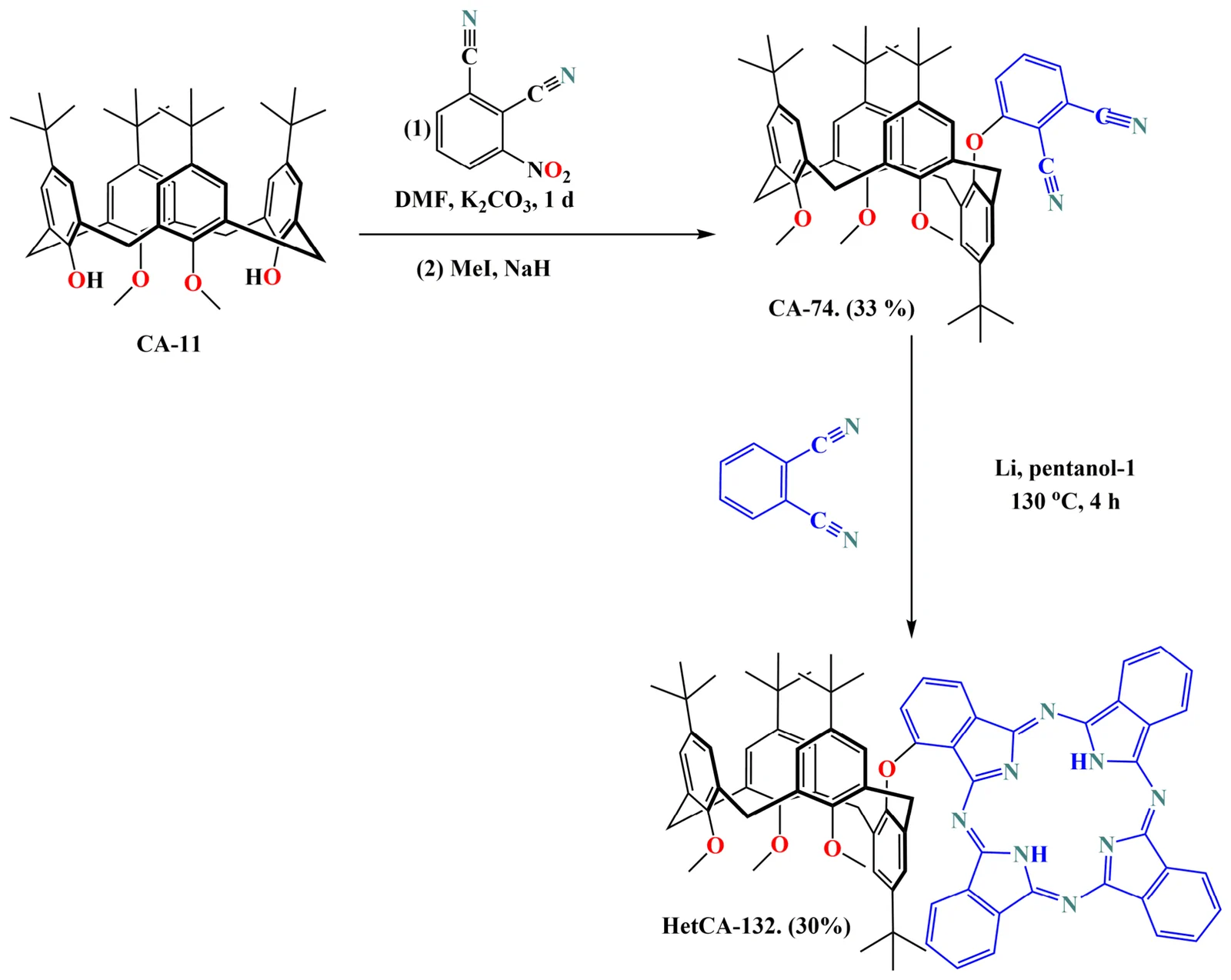
Divergent synthesis of phthalocyanine-modified **HetCA-132** [521].

O’Malley et al. (Ireland) demonstrated an interesting route to produce the macrocyclic derivative **HetCA-132** [521] modified with a tetraaza-phthalocyanine fragment (Scheme 71). To create a phthalocyanine macrocycle, the *o*-dinitrilophenyl substituent of calixarene **CA-74** was reacted with phthalocyanine in pentanol in the presence of metallic lithium and heated to boiling, with a final product yield of 30%. A similar synthesis was also presented in the work [522].

#### 3.2.2. Synthesis of Upper Rim-Bridged Calixarenes

The more unusual approach involves modifying the calixarene skeleton itself, transforming it into a calixheteroarene [523]. P. Lhoták and his co-authors from the University of Chemistry and Technology in Prague (Czech Republic) noted that this type of heterocyclic modification of calixarenes is quite rare [122], although such reactions can often proceed stereoselectively [524]. In some cases, such heterocyclic cross-linking reactions along the upper rim can lead to the formation of new ionic structures in the mass spectra [525].

Upper-rim-bridged calixarenes, in contrast to the side-chain divergent approaches discussed in Section 3.2.1, are obtained by forming a heterocyclic bridge directly between two or more positions of the upper rim, thereby incorporating the heterocyclic unit into the macrocyclic framework itself. This structural modification imposes additional conformational constraints that can stabilize a defined macrocyclic geometry and, in some cases, generate inherently chiral architectures. Consequently, upper-rim bridging can provide increased structural rigidity, preorganized binding cavities, and opportunities for chiral recognition, offering functional features that are difficult to achieve through conventional side-chain functionalization.

**Scheme 72:**
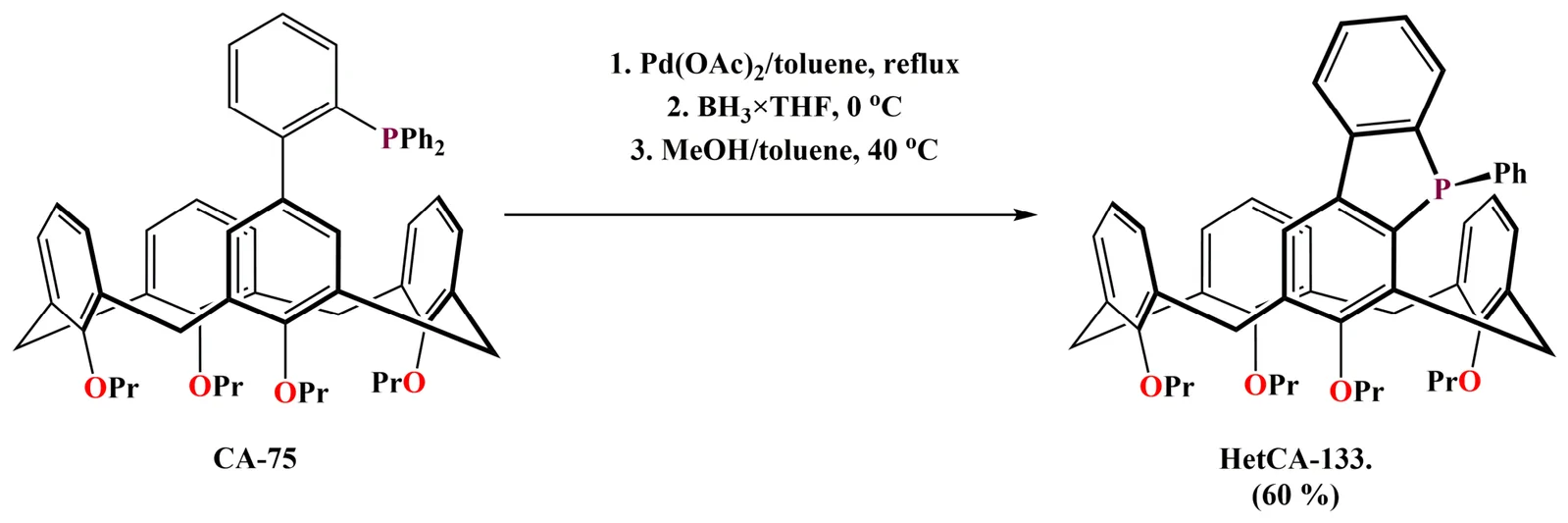
Synthesis of macrocyclic phosphole derivative **HetCA-133** [526].

In 2017, Elaieb et al. obtained calixarene-condensed phospholes **HetCA-133** using the Catani method [526] (Scheme 72). This reaction is characterized by stereoselectivity, which can be explained by the preferential formation of a 3:1 *endo*-adduct intermediate with borane. **HetCA-133** was isolated from the reaction mixture by column chromatography before the third stage.

**Scheme 73:**
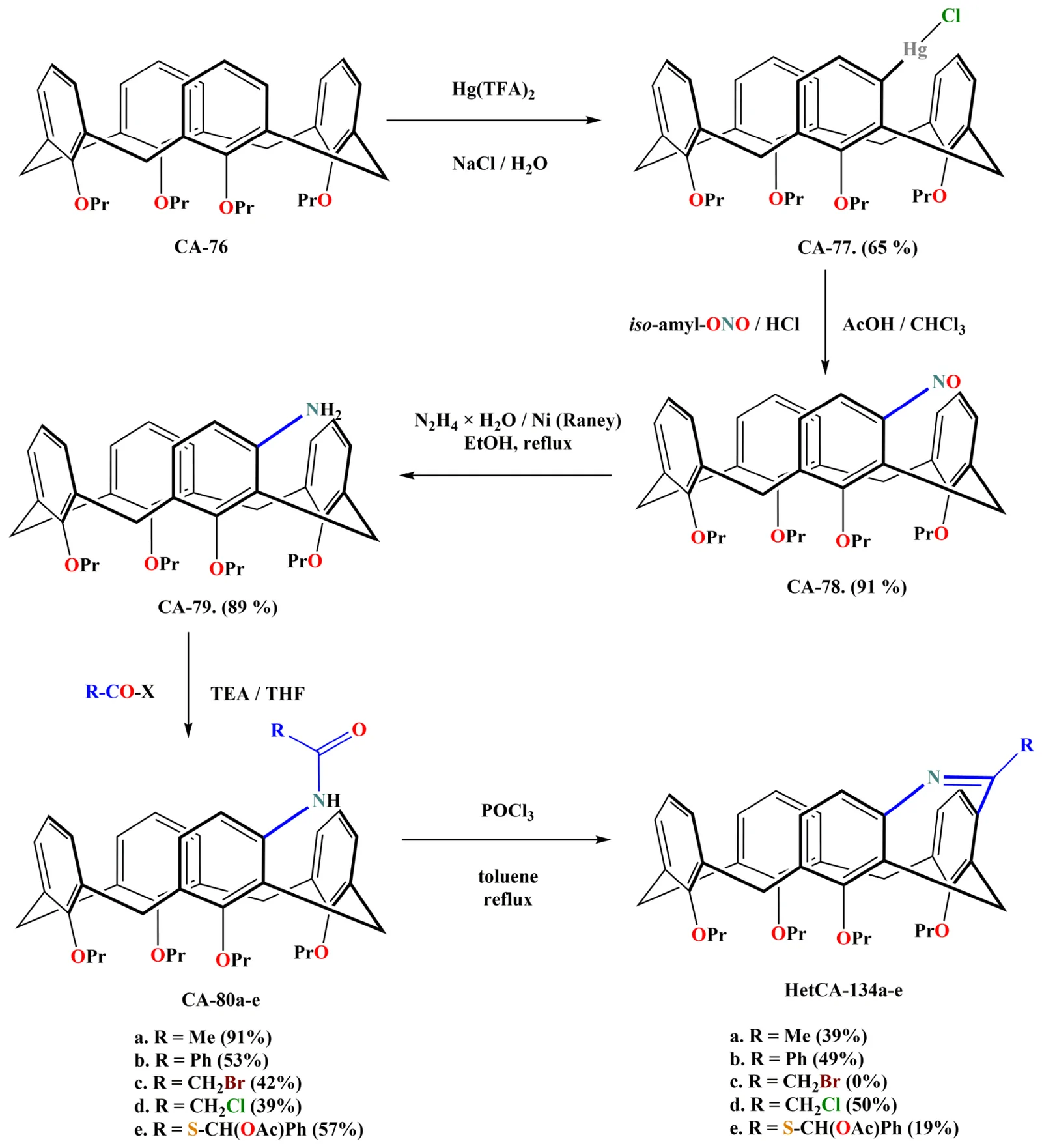
Synthesis of isoquinoline derivative **HetCA-134** [527].

The approach developed by Lhotak and co-authors, i.e., introducing an amino group in the *meta*-position to the hydroxyl group, is noteworthy [527]. The amino group is introduced in several stages via a mercury-organic intermediate compound **CA-77** (Scheme 73). The same scientific group developed the approach for obtaining this compound [528,529] and nitrosyl derivative **CA-78** [530] earlier. Then, aminocalixarene **CA-79** was converted into amide derivative **CA-80**, which was subjected to the Bischler–Napieralski reaction to form isoquinoline derivatives of calixarene **HetCA-134**.

**Scheme 74:**
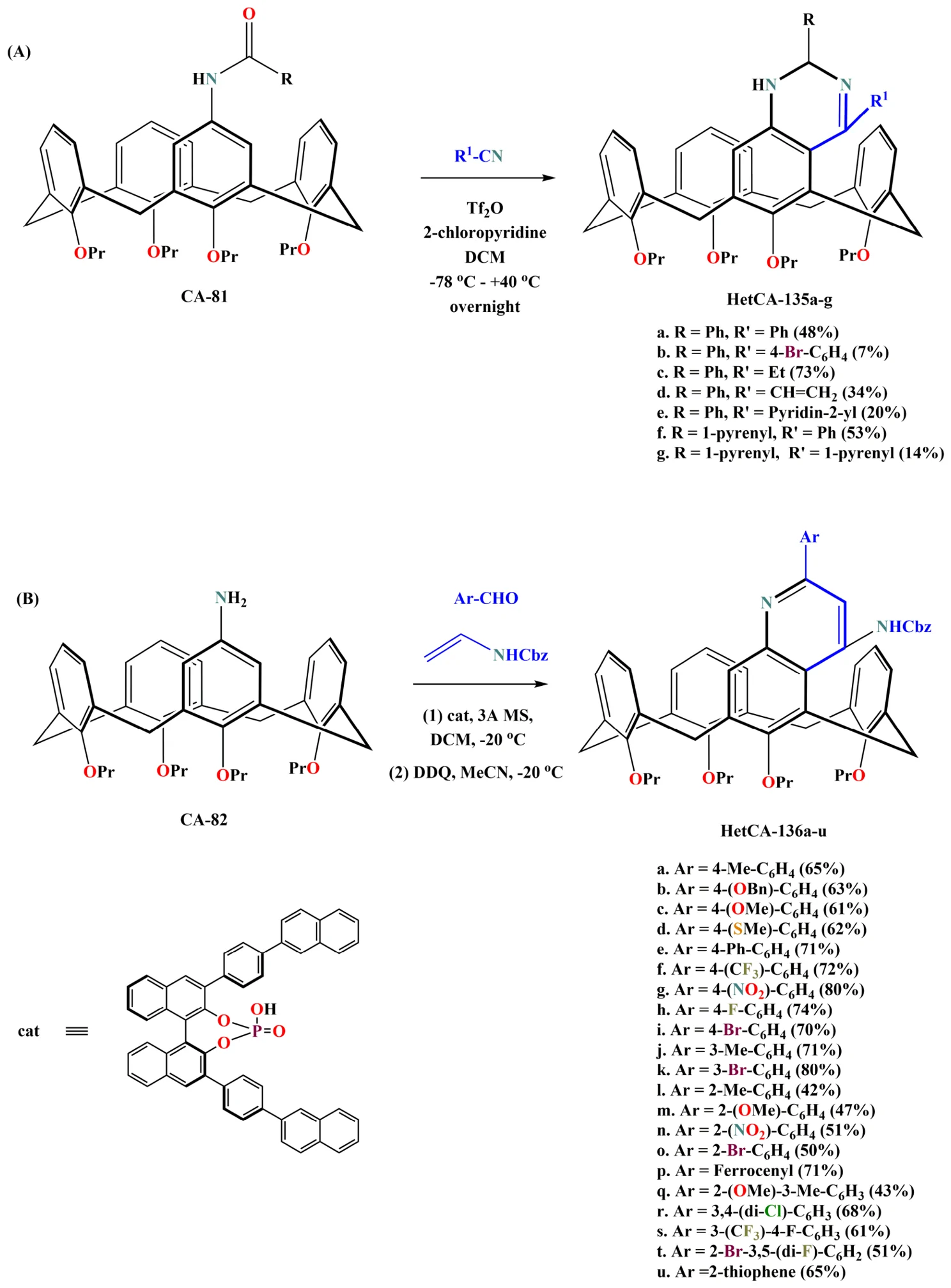
Synthesis of (**A**) quinazoline derivatives **HetCA-135** [122], (**B**) quinoline derivatives **HetCA-136** [531].

Tlusty et al. obtained a series of calixarenes **HetCA-135** condensed with quinazolines [122] from calixarenes **CA-81** containing amide fragments in the *para*-position to the hydroxyl group (Scheme 74A). A logical improvement on this method can be found in a study published by Jiang et al. [531]. In particular, the reaction of amine **CA-82** acylation on the upper rim and its cyclization into quinoline now took place in a one-pot variant to synthesize **HetCA-136**. The chemistry of such **HetCA**s has been gradually developing in recent years, as evidenced by the rapidly growing number of publications on this topic [532,533,534] (Scheme 74B).

Thus, we can conclude in Section 3.2 that the divergent strategy of **HetCA** synthesis, when used alongside the convergent strategy, is a powerful tool for obtaining macrocyclic compounds that are both scientifically and practically significant. The heterocyclic fragments introduced by the divergent strategy can be quite diverse, including quinolines, tetrazoles, phthalocyanines, porphyrins, spiropyranes, and benzimidazoles. Additionally, a special set of methods allowed heterocyclic fragments to be “built up” directly as a continuation of the aromatic rings of the calixarene core. This method has recently gained popularity in chemical synthesis. In summary, the synthetic section of our review paper (Section 3) highlights the extensive variety of synthesized **HetCA**s, incorporating diverse heterocyclic fragments, varying numbers of substituents, and modifications to the upper and lower rims. The arsenal of synthetic approaches for obtaining **HetCA**s is vast. Selecting heterocyclic structures gives the macrocycle the desired physicochemical properties, such as solubility in certain solvents and the ability to interact with various substrates.

## 4. Analysis of Synthetic Methods and Structure–Property Relationships

### 4.1. Analysis of Synthetic Approaches

The literature review demonstrates that Williamson-type nucleophilic substitution reactions predominate among synthetic approaches to the preparation of **HetCA**s (~60% of all synthetic studies analyzed in the review), with benzylic electrophiles (Type **I**, ~22%) being the most frequently used. CuAAC reactions also account for a significant share (~14%), reflecting the current trend toward using click chemistry to introduce bioactive moieties. Cross-linking reactions (Steglich-like, ~11%) and Schiff base synthesis (~9%) predominate in studies aimed at creating biomimetic systems. The smaller proportion of divergent approaches (~19%) is explained by the higher synthetic complexity of assembling the heterocycle directly on the macrocyclic scaffold.

An analysis of nucleophilic substitution reactions involving heterocyclic electrophiles (Section 3.1.1) shows that the choice of electrophile is determined not only by the reactivity of the heterocycle but also by the desired degree of substitution, conformational control, and steric constraints of the macrocyclic core. The most versatile are benzylic electrophiles (Type **I**), providing high yields and selectivity. The nature of the base and solvent is critical for the preparation of partially substituted derivatives, and for thiacalixarenes, the template effect of the alkali metal cation is crucial. It is important to emphasize that the vast majority of the syntheses reviewed use unmodified (thia)calix[4]arenes containing free phenolic hydroxyl groups as starting substrates. This is a key advantage of the approach under consideration, as it allows for the direct introduction of heterocyclic fragments, bypassing the preliminary functionalization of the macrocyclic core.

Unlike Section 3.1.1, which describes cases where the electrophilic center is located on a heterocycle, the methods described in Section 3.1.2 involve transferring the electrophilic center to the macrocyclic backbone. This allows for the introduction of heterocyclic moieties containing nucleophilic groups (amines, thiols, alcohols) that are incompatible with direct alkylation conditions. Although this approach requires preliminary functionalization of the calixarene (introduction of haloalkyl, tosyl, or acyl groups), it provides higher yields in the final step (50–99%) and opens access to biologically active conjugates, including penicillin, quinolone, and peptide-based prodrugs.

A comparative analysis of Section 3.1.1 and Section 3.1.2 reveals their complementarity: the first method is preferable for rapid screening of heterocycle libraries on unmodified macrocycles, while the second is suitable for the targeted introduction of functionally complex and sensitive fragments. The choice of approach is determined not only by the nature of the heterocycle but also by the required degree of substitution, conformational control, and the end use of the target product. Williamson-type alkylation reactions often suffer from insufficient selectivity, particularly in the case of thiacalix[4]arenes. In some cases, this approach also requires the use of strong bases, such as sodium hydride, and is poorly compatible with substrates bearing hydroxyl or amino groups. Nevertheless, the method is relatively inexpensive and enables the introduction of a broad range of structurally diverse, relatively simple substrates.

Steglich-type cross-linking reactions (Section 3.1.3) are among the most reliable methods for synthesizing new **HetCA**s. Their key advantage is the exceptionally mild conditions, allowing the introduction of heat-labile, stereochemically sensitive and biologically active heterocycles incompatible with harsh alkylation conditions. The main reaction types are amidation, esterification, and hydrazide condensation. The use of activating agents (DCC, EDC, and PyBOP) in combination with organic bases (DMAP, TEA, and DIPEA) provides high yields (40–98%) at room temperature. This approach is particularly in demand for the creation of biomimetic systems, peptide–calixarene conjugates, glycoclusters, and prodrugs, where preservation of the native pharmacophore structure is critical for biological activity. For cross-linking reactions, the main limitation remains the formation of difficult-to-remove by-products (for example, DCU from DCC), which complicates purification, especially for polar or large macrocyclic products. The development of polymer-supported coupling reagents or water-soluble carbodiimides can facilitate this issue. Additionally, the relatively low yields observed for the introduction of sterically hindered heterocycles via amide bond formation (for example, porphyrins) indicate that alternative activation strategies, such as the use of HATU or PyBOP, may be explored more systematically.

Azide–alkyne addition methods (in particular, CuAAC) are also suitable for the creation of biomimetic **HetCA**s. The key difference between this approach and those discussed above is that the reaction forms a new heterocycle (1,2,3-triazole), which serves not only as a linker but also as an active functional element capable of metal coordination, hydrogen bonding, and biomolecular recognition. The mild conditions of CuAAC (room temperature, aqueous–organic mixtures, and the absence of strong bases) ensure compatibility with sensitive functional groups, making this method suitable for the introduction of biologically active moieties (sugars, peptides, and nucleosides) and the creation of multivalent glycoclusters. High yields (43–99%) and the commercial availability of azide and alkyne derivatives have led to the widespread use of CuAAC in **HetCA** chemistry, particularly in research aimed at creating biocompatible systems and sensors. Regarding CuAAC methods, the development of copper-free variants that would eliminate toxicity concerns and simplify purification represents a particularly promising direction.

The synthesis of Schiff bases, hydrazones, and related compounds (Section 3.1.5) represents one of the most direct and efficient methods for introducing heterocyclic fragments into the structure of (thia)calix[4]arenes. The key advantage of this approach is its exceptional simplicity: the condensation reaction between macrocyclic amines or hydrazides and heterocyclic aldehydes/ketones typically proceeds without catalysts under mild conditions in high yields (68–95%). Unlike methods requiring activating agents or metal catalysts, this approach requires neither additional reagents nor protection of functional groups. The resulting C=N bond acts not only as a linker but also as a functional element capable of coordinating metals (via the nitrogen atom), participating in hydrogen bonds, and exhibiting photophysical properties (isomerization, fluorescence). This makes Schiff bases and hydrazones particularly attractive for the creation of chemosensors, fluorescent probes, and chelating ligands. The main limitations are the hydrolytic instability of the C=N bond in acidic environments and the possibility of E/Z isomerism, which, however, does not diminish the value of this approach for the design of sensor systems.

Electrophilic addition to a tertiary nitrogen atom (Section 3.1.6) is the least common of the convergent methods, but its functional significance extends far beyond the simple introduction of a heterocyclic moiety. Quaternization allows for the introduction of a permanent positive charge into the **HetCA** structure, opening the way to water-soluble derivatives capable of electrostatic interaction with anionic substrates (DNA, proteins, and cell membranes). For quaternization reactions, the main challenge is the control of selectivity, particularly in thiacalixarenes, where the “*distorted cone*” conformation can lead to undesired elimination products. The design of more sterically controlled alkylating agents or the use of milder conditions could improve the reproducibility and scope of this approach. The potential of quaternization to create water-soluble, cationic **HetCA**s for biomedical applications remains largely underexploited.

Unlike convergent approaches (Section 3.1), where the heterocyclic moiety is introduced as is, divergent strategies (Section 3.2) involve the stepwise construction of the heterocycle directly on the macrocyclic backbone. Although less common, these approaches open access to unique structures that are otherwise inaccessible: isoxazole and tetrazole linkers, fused heterocycles (quinazolines, isoquinolines), and more complex macrocyclic systems (porphyrin–calixarenes, phthalocyanine–calixarenes). Divergent approaches, in turn, can be divided into two groups: the creation of heterocyclic fragments in side substituents of **HetCA**s and the synthesis of upper rim-bridged calixarenes. The main limitations of divergent approaches are the multi-step nature, often low yields (especially for porphyrins), and the need for careful control of stereochemistry and regioselectivity. However, these methods provide the greatest structural diversity and enable the creation of heterocyclic systems with unique properties that are often unattainable by convergent methods.

Each of the synthetic strategies discussed above combines specific advantages with a number of practical limitations. Future developments in the synthesis of **HetCA**s will likely focus on addressing the key limitations of each methodology. For Williamson-type alkylation, improved selectivity for thiacalixarene partial substitution remains a priority. Cross-linking reactions would benefit from by-product-free coupling strategies, while Schiff base chemistry requires more hydrolytically stable analogs. CuAAC would be further enhanced by copper-free variants, and quaternization would gain from milder, more selective conditions. Divergent approaches hold promise for the construction of hetero-multivalent systems, provided that one-pot or cascade reactions can be developed to reduce the number of synthetic steps. The integration of emerging techniques such as flow chemistry, photochemical activation, and computational reaction design is expected to accelerate progress across all these areas.

### 4.2. Analysis of the Structure–Property Relationships of HetCAs and Synergy in the Accumulation of Heterocyclic Fragments

When selecting a specific synthesis strategy, researchers focus not only on synthetic accessibility, but also on the set of emerging structural features that impart specific physicochemical properties to materials based on the synthesized **HetCA**s. Unlike acyclic systems, where the properties of heterocycles manifest additively, their immobilization on a preorganized platform leads to the formation of qualitatively new, emergent properties that cannot be reduced to the sum of the characteristics of the individual components. This phenomenon is due to spatial proximity, mutual electronic interactions, and the cooperative functioning of heterocyclic ensembles within the rigidly defined geometry of the macrocycle. Such emergent properties arise as a result of the synergy between the heterocyclic moieties and the macrocyclic backbone.

The design of target **HetCA** structures is driven by the specific functions and properties that researchers aim to achieve. In the case of calix[4]arenes, several structural factors play an important role, including the degree of substitution (monosubstitution, proximal 1,2-disubstitution, distal 1,3-disubstitution, trisubstitution, and tetrasubstitution), the nature of the heterocyclic fragment, the type of linker connecting the heterocycle to the macrocyclic framework, and the conformation of the macrocycle (*cone*, *partial cone*, or *1,3-alternate*). As noted above (at the beginning of Section 3), the nature of the heterocyclic moieties plays a crucial role in determining the potential applications of the modified macrocycles. Depending on their structural features, heterocycles can provide **HetCA**s with coordination properties, optical activity, biological functions, electronic properties, catalytic activity, or serve as versatile platforms for further transformations. The connection between the presence of certain structural features of specific **HetCA**s and the physicochemical properties they exhibit can be most clearly illustrated by specific examples.

#### 4.2.1. Influence of Calixarene Rim

The choice of a calixarene rim for modification is not a trivial matter and depends on many factors. Firstly, the lower rim is most often modified because it is synthetically simpler than approaches that involve de-alkylation of the upper rim and then modifying it. Among the key advantages of lower-rim modification is the ability to achieve targeted conformational control, yielding *cone*, *partial cone*, and *1,3-alternate* stereoisomers. In contrast, upper-rim functionalization in the vast majority of cases results in the formation of derivatives exclusively in *cone* conformation.

The spatial orientation of the substituents, determined by their position on the macrocyclic core, is also significant. In *cone* conformation, the substituents anchored to the lower rim point inward into the macrocyclic cavity, while the upper rim substituents point outward, toward the periphery of the molecule, making them maximally accessible for interaction with external targets—proteins, cell surfaces, and polymer matrices. Therefore, the upper-rim modification is preferred for creating biocompatible systems where functional groups must be exposed to the external environment, as in the case of **HetCA-78** [384]. Lower-rim modification is suitable for creating ligands in host–guest systems, as the substituents are then oriented toward each other. Most examples of the preparation of ligands for various substrates described in this review involve lower-rim-modified **HetCA**s.

#### 4.2.2. Influence of Number of Substituents and Their Spatial Arrangement

As an example of the influence of the degree of substitution and the spatial arrangement of heterocyclic fragments on the spectral properties of calixarenes, bithiazole derivatives reported in the work [185] can be considered. The authors obtained a series of calixarene derivatives (**HetCA-3**, **HetCA-5**, **HetCA-9**, **HetCA-11**, and **HetCA-13**) modified with bithiazole fragments and studied their fluorescence properties. The accumulation of bithiazole moieties on the calix[4]arene platform results in non-additive fluorescence properties determined by two competing intramolecular processes: lateral and transannular quenching. The efficiency of these processes depends on the number and relative positions of the heterocycles: a distal (**HetCA-5**) position minimizes losses, while proximal (**HetCA-9**) and triple substitution (**HetCA-11**) enhance quenching. The tetrasubstituted derivative **HetCA-13** exhibits the highest absolute intensity due to energy redistribution across multiple chromophores.

#### 4.2.3. Influence of Conformations of (Thia)calix[4]arene Backbone

The conformations of **HetCA**s play an important role in the properties of the obtained compounds, as they affect their solubility, as well as the topology of the binding sites, which, for example, is clearly demonstrated in work [383]. Biomimetic **HetCA-77b** containing *D*-galactopyranose fragments showed different affinities for the lectin PA-IL depending on the conformation of macrocycle **HetCA-77b**, namely, the macrocycle in *partial cone* and *1,3-alternate* conformations had a higher affinity than the *cone* stereoisomer.

#### 4.2.4. Influence of Nature of Linkers Between Macrocyclic Core and Heterocyclic Moiety

**Figure 7:**
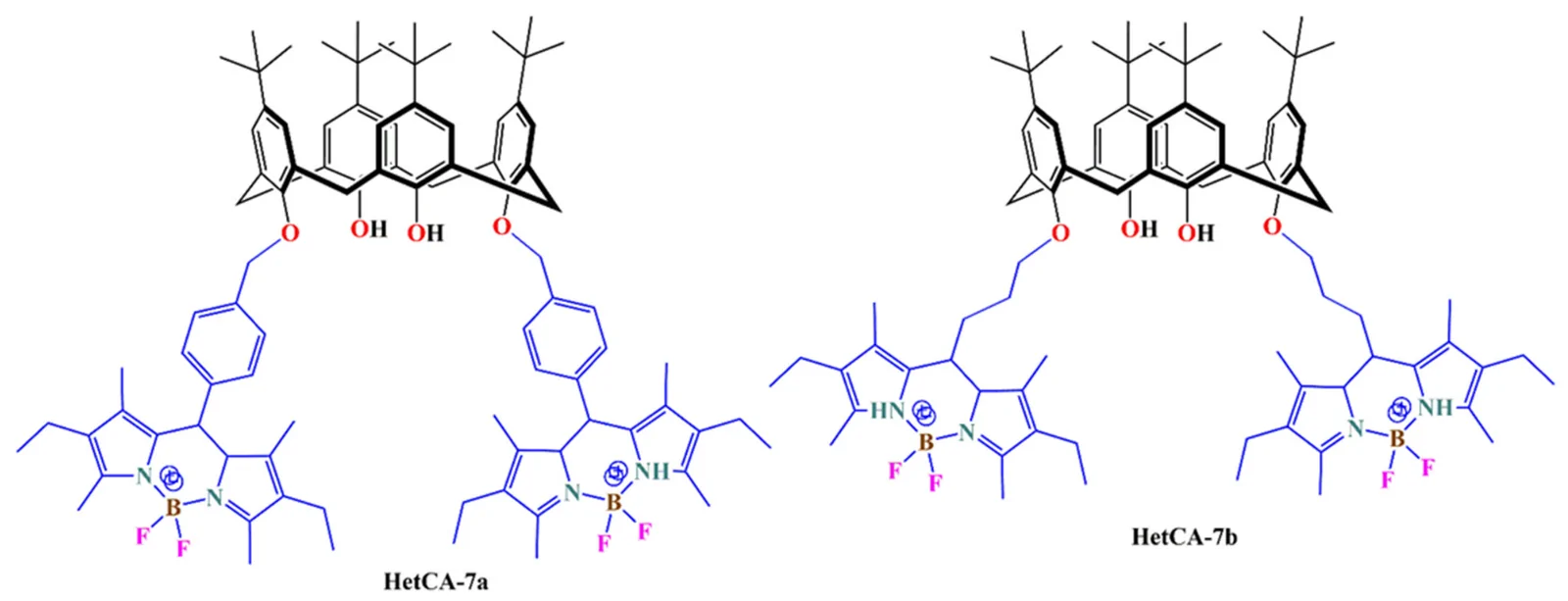
Structures of **HetCA-7a** and **HetCA-7b** with different linkers.

The properties of **HetCA**s are governed not only by the macrocyclic conformation and the number and spatial arrangement of heterocyclic substituents, but also by the chemical nature and flexibility of the linkers connecting these fragments to the macrocyclic platform [73]. Thus, variation in the linker provides a means for targeted modulation of biological activity: a rigid aromatic linker (like in **HetCA-7a**) enhances DNA binding, whereas a flexible aliphatic linker (as in **HetCA-7b**) facilitates intercalation and promotes more efficient cleavage. These results demonstrate the crucial role of the linker as a structural element that determines not only the conformational mobility of the chromophore but also the mechanism of its interaction with biological targets (Figure 7).

Every detail often matters in the design of **HetCA**s. For example, the linking of the nitrobenzofurazan moieties to the macrocyclic backbone via amino groups in the case of **HetCA-28** is no coincidence. In sensor **HetCA-28**, photoinduced electron transfer from the amino group (donor) to the nitrobenzofurazan moiety (acceptor) leads to fluorescence quenching; coordination of Ag(I) or hydrogen bonding with AcO^−^ alters the electron density on the amino group, modulating the photoinduced electron transfer (PET) process and causing the spectral response [206].

The introduction of moieties that make linkers more rigid is a fairly common approach. For example, an important functional feature of the 1,2,3-triazole linker is its rigidity, which, as demonstrated by Cindro et al. [392], prevents the aggregation of carbohydrate moieties in glycocalixarene **HetCA-83**. By fixing the sugar residues in a spatially separated position, the triazole ring minimizes their intermolecular contacts, ensuring the stability of aqueous solutions.

#### 4.2.5. Influence of Calixarene Core Number

The synthesis of bis-calixarenes linked by heterocycle-containing linkers also pursues very specific goals. As a rule, bis-calixarenes are obtained due to the creation of an additional cavity between two residues of the macrocycle, in which additional coordination of the host with the guest molecule or ion can occur [233]. However, it should be emphasized that the creation of an additional cavity does not always lead to enhanced binding. As shown in [193], pyridine-bridged bis-calixarene **HetCA-17b** turned out to be an ineffective extractant of alkali metals, despite the presence of two calixarene cavities. This is explained by the mismatch of the cavity geometry (too low rigidity for Rb(I) and Cs(I)) and the lack of a cooperative effect between the two macrocycles. In contrast, in the case of **HetCA-115** [484], containing isoxazole and anthracene fragments, the rigid rectangular cavity ensures selective binding of methyl viologen due to cation–π interactions and the optimal cavity size.

The most important consequence of this synergy is the ability to finely tune the selectivity of molecular recognition. The preorganized structure of the calixarene scaffold, which carries several heterocycles, allows for the creation of binding pockets with precisely defined size, shape, and set of donor atoms. In this case, it is the combination of heterocycles that determines the geometry and electron density of the receptor site. This ensures high selectivity toward substrates with similar properties, including the distinction between alkali and alkaline earth metal cations, as well as between transition metals and lanthanides.

Thus, in this section, we highlighted the main trends in synthetic approaches, summarizing the previous Section 3, in which we discussed preparative details. In the second part of this section, we discussed the relationships between various structural features of calixarene (choice of upper or lower rim, the number of substituents and their relative orientation, macrocycle conformation, and the nature of the linker). The structure–property relationships discussed above directly govern the functional behavior of **HetCA**s in diverse application areas. To provide an overview of these connections, Table 1 summarizes how the key structural parameters of **HetCA**s influence their performance in coordination chemistry, sensing, catalysis, and biomedical applications. This framework serves as a guide for the detailed discussion of each application domain in Section 5.

## 5. Applications of Heterocycle-Containing Calixarenes

The synthesis of heterocycle-containing calix[4]arene derivatives (**HetCA**s) represents an important strategy for expanding the functional potential of calixarene-based systems, as discussed in Section 3 of this review. The wide variety of synthetic methods and structural modifications involving heterocyclic fragments has led to a broad range of compounds with diverse properties and applications. These applications are a consequence of the presence of heterocyclic sites in calix[4]arene structures, which act as binding sites for various compounds. Binding selectivity to targets is due to the presence of a rigid macrocyclic skeleton that preorganizes the heterocyclic binding sites and determines their mutual arrangement geometry.

In particular, calixarenes provide a versatile platform for the design and synthesis of ligands for various substrates whose properties can be systematically tuned for specific purposes. Owing to the preorganized structure of the calixarene framework and the relative ease of introducing various functional groups containing different heteroatoms, these macrocycles are widely regarded as classical host molecules in supramolecular and coordination chemistry.

The ability to coordinate with various substrates allows **HetCA**s to serve as promising catalysts in reactions such as aldol condensation, the Mannich reaction, and various cross-coupling reactions. The use of **HetCA**s in catalysis is an actively developing research area. Although truly breakthrough advances have not yet been achieved, the steadily growing number of studies demonstrates the significant potential of these systems for the design of new catalytic materials.

One of the most actively studied areas is the use of calixarene derivatives (and **HetCA**s in particular) as molecular carriers and drug delivery systems. Functionalized calixarenes are capable of forming host–guest complexes with pharmaceutical compounds, which can improve their stability, solubility, and bioavailability. In addition, certain calixarene derivatives have demonstrated the ability to selectively bind biomolecules, making them promising candidates for the development of biosensors and diagnostic tools.

**HetCA**s are also being investigated for their intrinsic biological activity. Depending on the nature of the substituents, these macrocycles can exhibit antimicrobial, antiviral, and anticancer properties. Moreover, the multivalent architecture of calixarenes allows the simultaneous presentation of several functional groups, which can enhance interactions with biological targets and increase the effectiveness of the resulting systems.

This section highlights the practical applications of **HetCA**s across a broad range of fields. Section 5.1 focuses on the coordination chemistry of calixarenes and their use in sensing systems. Section 5.2 then addresses the development of **HetCA**-based catalysts. Finally, Section 5.3 discusses selected aspects of the biomedical applications of **HetCA**s.

### 5.1. Complexation and Sensory Applications of Heterocycle-Containing Calixarenes

This section focuses on the complexation of **HetCA**s with various substrates. Depending on the nature of the guest species, it is divided into three parts, each addressing the interaction of **HetCA**s with one of the following classes of substrates: cations, anions, and neutral organic molecules. To facilitate navigation through the review, this section is numbered independently from Section 3, which is devoted to the syntheses of calixarenes.

#### 5.1.1. Complexation of Metal Cations by Heterocycle-Containing Calixarenes

**Figure 8:**
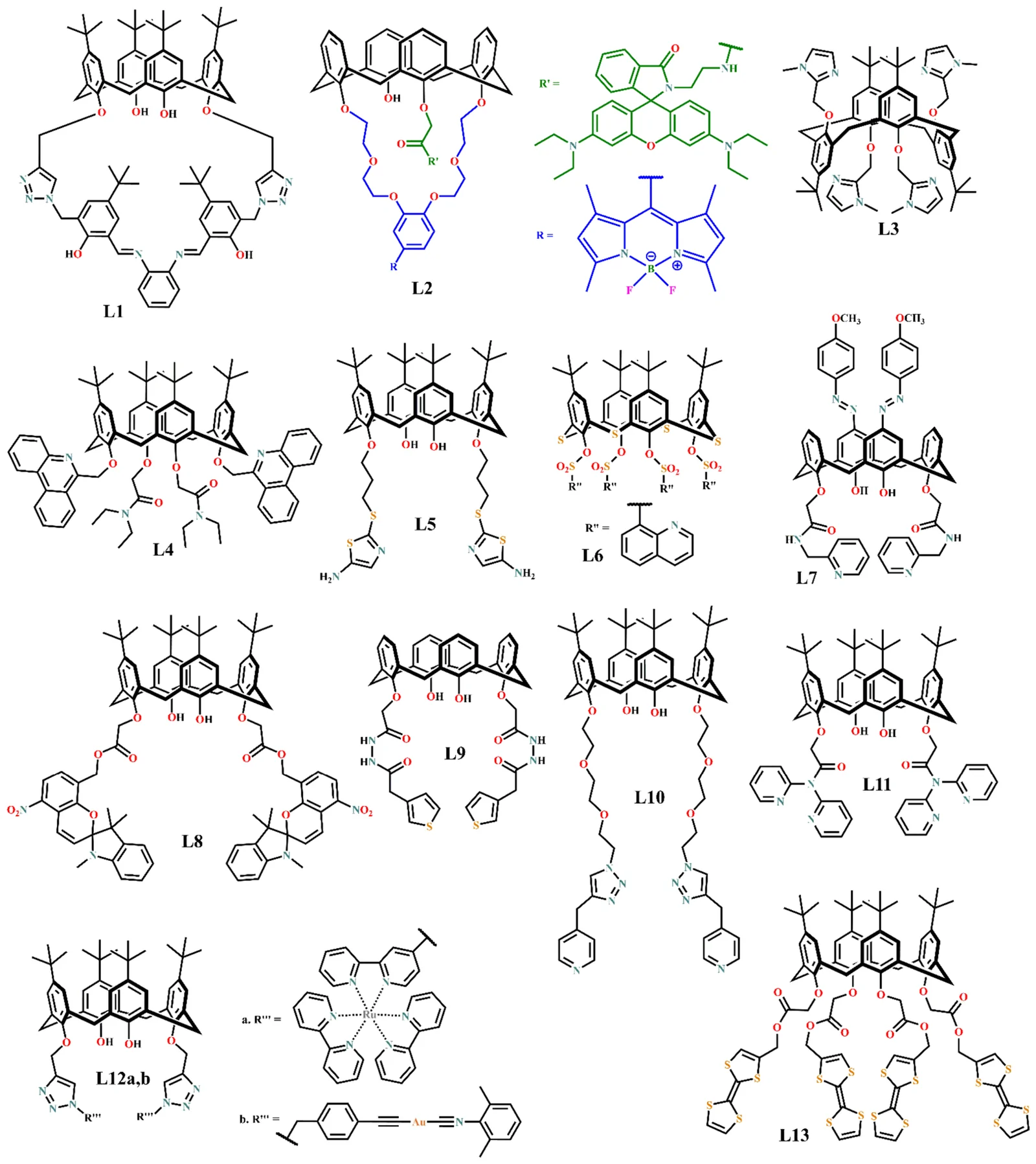
Structures of **HetCA**s **L1**–**L13** for metal cations complexation.

Metal complexes based on calixarenes are very common [535,536,537,538,539,540,541,542,543]. Fused heterocycles often act as the main structural unit of fluorescent compounds [244,544]. Figure 8 shows the structures of **HetCA**s **L1–L13** for metal catios complexation.

Many heterocycles are good fluorophores whose spectral characteristics depend on the concentration of ions and the solvents used. Additionally, over the past decade, calixarene derivatives like **L1** and **L2** have become increasingly prevalent in molecular logic gates. These gates are based on responses to various substrates, expressed as the conditional signals “1” and “0”, which are then converted into gate responses according to the laws of Boolean mathematics [545].

The **HetCA**s used for cation binding are predominantly characterized by 1,3-disubstitution at the lower rim with electrically neutral, structurally simple heterocyclic fragments containing nucleophilic nitrogen, oxygen, or sulfur atoms. In addition, short, rigid linkers are employed in the design of **HetCA** receptors to promote structural preorganization of the calixarene framework for selective binding of specific cations. In contrast, longer linkers (as in the case of **L10**), such as ethylene glycol ether derivatives, can also be employed; these not only provide additional donor sites but also enhance the solubility of macrocyclic derivatives in polar solvents. In most of the examples considered in Figure 8, the response of macrocyclic receptors toward cations is optical, as detected by UV–Vis absorption or fluorescence spectroscopy. In addition to these spectroscopic approaches, electrochemical sensing methods have also been employed for cation detection (**HetCA L13**).

In general, 1,3-disubstituted calixarenes are used for sensory applications [546], although not exclusively. Thus, in a highly indicative study [136], an international team of authors from Japan, China, the United Kingdom, and Canada demonstrated the high extraction capacity of the imidazolium derivative *p*-*tert*-butylcalix[4]arene **L3** in *1,3-alternate* stereoisomeric form toward Cu(II), Ag(I), and Pb(II) picrates (extraction efficiency up to 80%). In addition, macrocyclic derivative **L3** was also found to be capable of effectively extracting dichromate anions (up to 80%) in a water–methylene chloride system at pH = 1.5.

The preorganization of macrocyclic structures enables their use in analytical tasks, even those involving alkali metal cations [415,547]. In 2023, K. Leko and his colleagues from the University of Zagreb (Croatia) presented a new design for calix[4]arene **L4** modified with a phenanthridine ring that acts as a ligand for Li(I), Na(I), K(I), and Cs(I) cations [145]. The obtained complexes were studied using a number of spectral methods, including UV–visible absorption and fluorescence spectroscopy in solution, microcalorimetry (ITC), and XRD analysis. For instance, in the case of K^+^, the binding constant of the complex was pK = 1.70 in DMF at 25 °C. In another study by authors from the same university, a glycoside structure of a calixarene derivative was designed, which is also a sensor for alkali metals [392]. Recently, the same group of authors published another study on this issue [548].

H. Hadi and R. Safari demonstrated that a fluorescent selective sensor **L5** for Pt(II) cations can be obtained based on a classical calixarene modified with aminothiol [227]. The titration data confirm a binding constant of 9.87 × 10^4^ M^−1^ and a 1:1 stoichiometry of the calixarene–platinum complex. According to density functional calculations, platinum ion coordination occurs through nitrogen atoms in the heterocycle.

One of the most popular approaches for detecting cations is the use of spectral methods, in particular fluorescence spectroscopy, which can be used to detect the presence of transition metal cations [143,147,385]. Fused heterocycles possess intrinsic fluorescence, which can be altered by the action of substrates [549]. Thus, Gujarat University (India) has shown [174] that a macrocyclic derivative of quinoline-8-sulfonate **L6**, which has its own fluorescence, undergoes quenching when interacting with cobalt Co(II), while the electronic absorption spectrum showed a slight bathochromic shift. As scope of this work, molecular docking and experimental determination of the stoichiometric ratio of metal cation to ligand (1:1) were performed.

**HetCA**s play a significant role in the synthesis of ligands for lanthanide cations [343,550,551]. A number of colorimetric sensors (e.g., **L7**–**L9**) based on the photochromism effect in the presence of various ions (including La(III), Pr(III), Eu(III), Gd(III), Dy(III), Er(III), Yb(III), Ca(II)^,^ Mg(II), Cu(II), Hg(II)), for instance, in papers [260,292,552,553,554]. **HetCA** pH sensors also can be obtained [555].

Calixarenes are an excellent material for synthesizing hybrid organo–inorganic particles that stabilize transition metal nanoparticles, such as silver [556]. Researchers from Wuhan (China) created a colorimetric sensor based on silver-based nanoparticles modified with calixarene containing triazole and pyridine fragments [388]. This appears to be an interesting development as in case of **HetCA L10** (iron(III) sensor). For example, systems for extracting rare earth metals and forming complexes with them, such as Eu(III) and Tb(III), are based on calixarenes modified with isoquinoline fragments [557,558].

**HetCA**s have been shown to exist and exhibit selectivity toward several substrates, each of which reacts differently. Thus, Joseph et al. synthesized a macrocyclic fluorescent sensor for Zn(II) and Ni(II) cations. When **HetCA L11** interacted with the Zn(II) cation, the fluorescence intensified; however, with the Ni(II) cation, the fluorescence was quenched [559].

There are data on the synthesis of **HetCA** ligands that can bind to noble metal ions, such as gold(I) and ruthenium(II). For example, polypyridine macrocyclic ligands form complexes with platinum group metals, in particular complex **L12a** with ruthenium(II). The resulting complexes exhibit the properties of fluorescent sensors for transition metal cations, in particular copper(II), with an association constant of 2.3 × 10^4^ M^−1^ in the HEPES–acetonitrile system. Under these conditions, metal cations act as fluorescence quenchers, which the authors explain by the photoelectron transfer effect [560]. A calixarene alkynyl complex **L12b** of gold(I) containing a triazole moiety was developed at the University of Hong Kong (China) [561]. This complex proved to be a sensor for zinc(II) cations with logK = 4.19 and a stoichiometry of 1:1 in a mixture of methylene chloride and acetonitrile.

In addition to spectral methods for detecting cations of certain metals, the ability of some redox-active heterocycles to selectively oxidize in the presence of certain metal cations can be utilized. Thus, J. Lyskawa and co-workers reported calixarene **L13** bearing a tetrathiafulvalene fragment. Using this electrochemically active unit, the authors developed a sodium cation sensor based on a *meta*-cyclophane framework and investigated its sensing behavior by cyclic voltammetry [287].

The study of coordination polymers based on calixarenes is a very active area of research [138,562]. In 2023, a group of authors from China described the synthesis of a calixarene-containing polymer impregnated with nickel(II) cations [177]. The polymer’s carbon skeleton is based on *p-tert*-butylcalix[4]arene, which has been modified with polyaminopyridine fragments. Nickel(II) cations are located within the cavities that exist between the heterocyclic sections. The resulting polymer was characterized using a variety of methods, including cryogenic microscopy, X-ray absorption fine structure spectroscopy, and IR and UV-Vis spectroscopy. Data on the electrochemical properties of the nickel-containing polymer based on a macrocyclic calixarene platform was also obtained. The following work [563] showed that this nickel-containing polymer can act as a microwave radiation absorber. Thus, a 1.5 mm layer of this hybrid material exhibits minimal reflection loss of −49.1 dB at a frequency of 13.04 GHz, corresponding to the microwave range of the electromagnetic spectrum.

Overall, the reported studies illustrate a clear tendency toward the development of increasingly sophisticated multifunctional hybrid receptor systems incorporating fragments such as triazole, BODIPY, and rhodamine. These structural motifs are widely employed to enhance the selectivity and signaling capabilities of metal-ion receptors. A dominant role is played by optical detection methods, particularly fluorescence and UV-Vis spectroscopy (in some cases other investigations methods can be used such as ITC or electrochemistry), reflecting the continuing demand for sensitive chemosensors capable of detecting toxic heavy metals (Hg(II), Pb(II), Cd(II)) as well as biologically relevant cations (Cu(II), Zn(II), Fe(III)). At the same time, the molecular design of ligands shows a gradual shift from relatively simple nitrogen-containing heterocycles to more complex architectures incorporating additional functional components, such as carbohydrates or fluorophores. This structural diversification enables more precise tuning of host–guest interactions and the thermodynamic stability of the resulting complexes (most commonly with 1:1 and 1:2 stoichiometries) and expands their applicability across a variety of media, ranging from organic solvents to aqueous environments. The available data on the complexation behavior of various **HetCA** structures are summarized in Table S14 [90,143,144,145,147,174,194,197,206,220,227,242,248,256,260,274,286,287,293,294,298,308,352,354,356,385,388,392,394,396,397,422,425,493,496,499,507,548,559,564,565,566,567,568,569,570,571,572,573,574,575,576,577,578,579,580,581,582,583,584,585,586,587,588,589,590,591,592,593,594,595,596,597,598,599,600,601,602,603,604,605,606,607,608,609,610,611,612].

#### 5.1.2. Complexation of Anions by Heterocycle-Containing Calixarenes

Some studies have demonstrated that **HetCA**-based receptors can interact with both cationic and anionic species. In such systems, the same ligand framework contains different binding sites capable of engaging in distinct types of noncovalent or coordination interactions, allowing the recognition of substrates of different charges. These results highlight the multifunctional character of calixarene scaffolds and illustrate their potential for the development of supramolecular systems capable of responding to both cations and anions.

**Figure 9:**
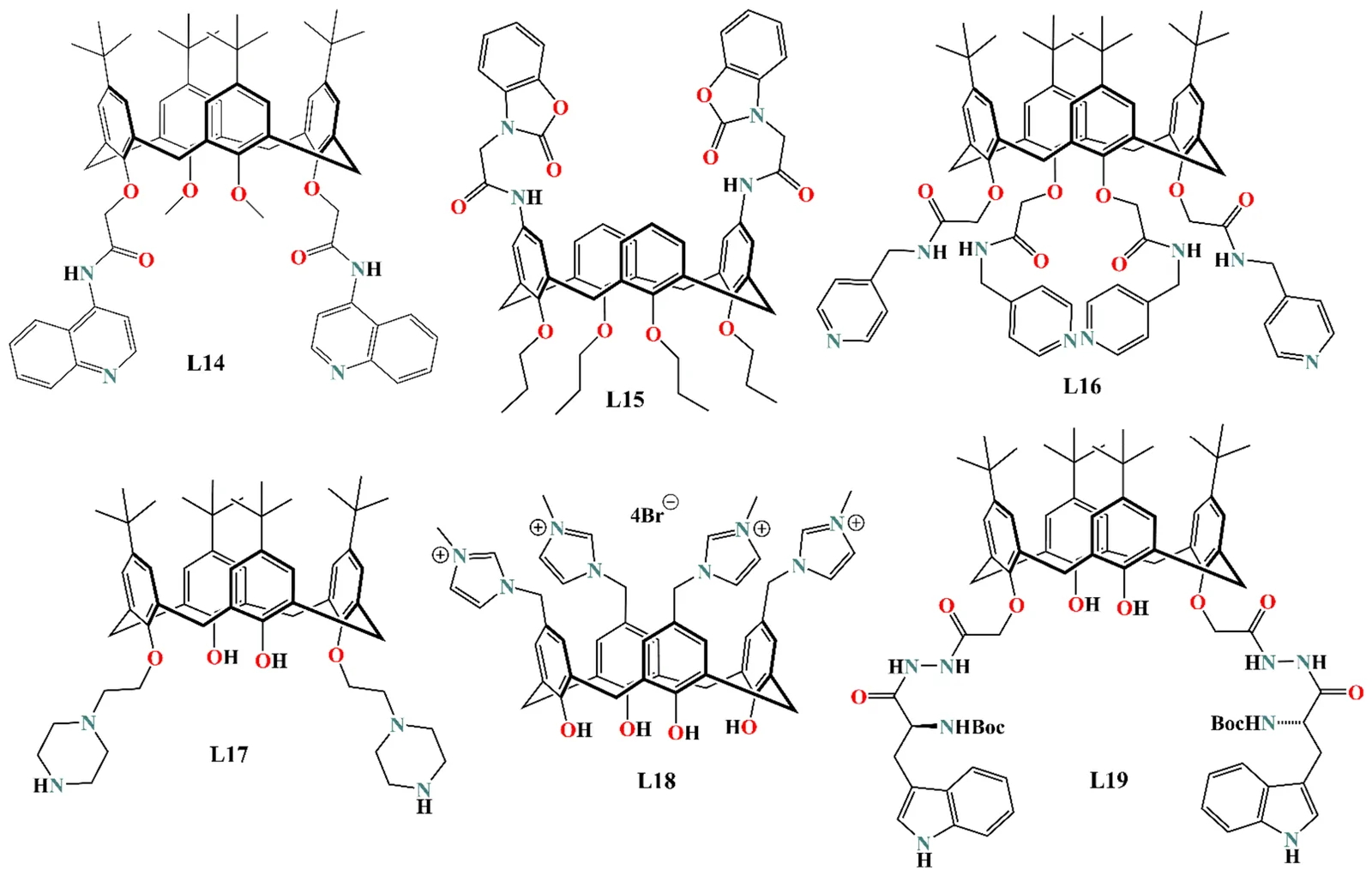
Structures of **HetCA**s **L14**–**L19** for anion complexation.

**HetCA** sensors can also be used to detect anions [613]. Figure 9 shows the structures of calixarene-based receptors for anions. A selective sensor **L14** for Sr(II) and CN^−^ ions was also obtained on the basis of the calixarene platform [274]. The aminoquinoline moiety acts as the binding site. When bound to the specified ions, the intensity of the recorded fluorescence increases. The detection limit for both ions in acetonitrile is less than 1.4 nM, making this sensor suitable for practical analytical studies. Gómez-Machuca’s group at the University of Chile achieved satisfactory results by creating a calixarene-based sensor **L15** with two benzothiazolinone fragments on the upper rim. This sensor can detect copper(II) cations as well as cyanide, fluoride, iodide, and acetate anions. The detection limits ranged from 5.13 to 83.17 μM, depending on the ion’s nature [308,614].

**HetCA**s have also been investigated as receptors for anionic species. Appropriate functionalization of the macrocyclic framework with groups capable of hydrogen bonding, electrostatic interactions, or other noncovalent contacts enables the formation of host–guest complexes with a variety of anions. These studies demonstrate that calixarene-based systems can serve as effective platforms for anion recognition and sensing, further expanding the scope of their applications in supramolecular and coordination chemistry. Compared with macrocyclic sensors designed for cation detection, those developed for anion recognition typically incorporate linkers and heterocyclic moieties bearing functional groups capable of hydrogen bonding with anions (such as N-H moieties). Positively charged imidazolium fragments can also facilitate anion recognition through electrostatic interactions with anionic substrates (as in the case of **L18**).

To promote sustainable development, analytical sensors must be created to detect toxic pollutants, such as arsenate ions. For example, calixarene **L16** has been found to be capable of selectively binding arsenate ions due to its four pyridine fragments [175]. Thus, with a pH of 3.5 and 1,2-dichloroethane as the organic phase, the extraction coefficient was 81.6%. The experiment was also conducted in the presence of chlorides, sulfates, and nitrates. The presence of these interfering ions slightly reduced the extraction efficiency to 78.8% when all of the ions were present simultaneously. Arsenate ions are toxic to animals [615,616,617], as well as to agricultural crops [618,619]. During a cascade of biochemical transformations in liver cells, arsenates are converted into organotin compounds, such as the highly toxic dimethylarsenite. In other words, lethal synthesis occurs [616]. In addition to the above work, we can draw attention to a similar study that resulted in the production of pyridine derivatives based on a macrocyclic platform. These derivatives are also capable of selectively detecting arsenate ions [246]. The early detection of arsenate ions can certainly play a major role in the analysis of natural objects.

Derivatives of saturated heterocycles, such as *p*-*tert*-butylcalix[4]arene modified with piperidine-containing moieties, can be used to study substrates of different nature. As demonstrated by the work of Minhas et al. [146], macrocyclic derivative **L17** is capable of extracting both Hg(II) ions (using the picrate extraction method) and dichromate ions from an aqueous environment, as demonstrated in the paper of Bozkurt et al. [620]. Mercury(II) derivatives act as neurotoxins [621,622], and dichromate ions are potent carcinogens [623,624]. The extraction percentage of dichromate ions at pH = 1.5 was 60.8%, while Hg(II) at pH = 3.5 was almost quantitative. The importance of analytical detection of such a powerful toxicant as mercury is evident in the number of studies devoted to this topic.

Similarly, a group led by E.K. Bullough from the University of Leeds (UK) presented a calix[4]arene **L18** modified with positively charged imidazoline fragments that exhibited sensory properties related to chloride, bromide and nitrate ions [238]. Qing et al. reported a macrocyclic enantioselective sensor **L19** based on an *L*-tryptophan fragment designed for the recognition of biologically relevant molecules. In particular, the authors demonstrated that this **HetCA** is capable of distinguishing between the *L*- and *D*-forms of several amino acid salts, as well as tartrate and malate anions [625].

The available data on the complexation behavior of heterocycle-containing receptors toward various anionic guests reveal a wide structural diversity of the heterocyclic moeties employed in structures of **HetCA**s, including naphthalimide, benzofurazan, benzoxazolinone, coumarin, imidazolium, indole, isoquinoline, pyridine, quinoline, and triazole derivatives. These **HetCA**s demonstrate the ability to recognize a broad range of anions, such as F^−^, CN^−^, AcO^−^, Cl^−^, I^−^, and several oxoanions, with association constants typically ranging from moderate to relatively high values depending on the receptor structure and the nature of the guest. Similar to cation recognition systems, optical techniques (primarily fluorescence and UV-Vis spectroscopy) remain the most commonly employed methods for studying these interactions, although NMR spectroscopy is also used to investigate binding processes in solution. In most cases, the formation of host–guest complexes with 1:1 stoichiometry is observed, while 1:2 and 2:1 complexes are reported less frequently. The binding studies are generally performed in organic solvents or mixed solvent systems, reflecting the influence of medium polarity on anion recognition processes. The available data on the complexation behavior of various **HetCA** structures are summarized in Table S15 [204,206,238,274,293,308,496,565,570,582,625,626,627,628].

#### 5.1.3. Complexation of Organic Molecules by Heterocycle-Containing Calixarenes and Synthesis of Photomaterials

**Figure 10:**
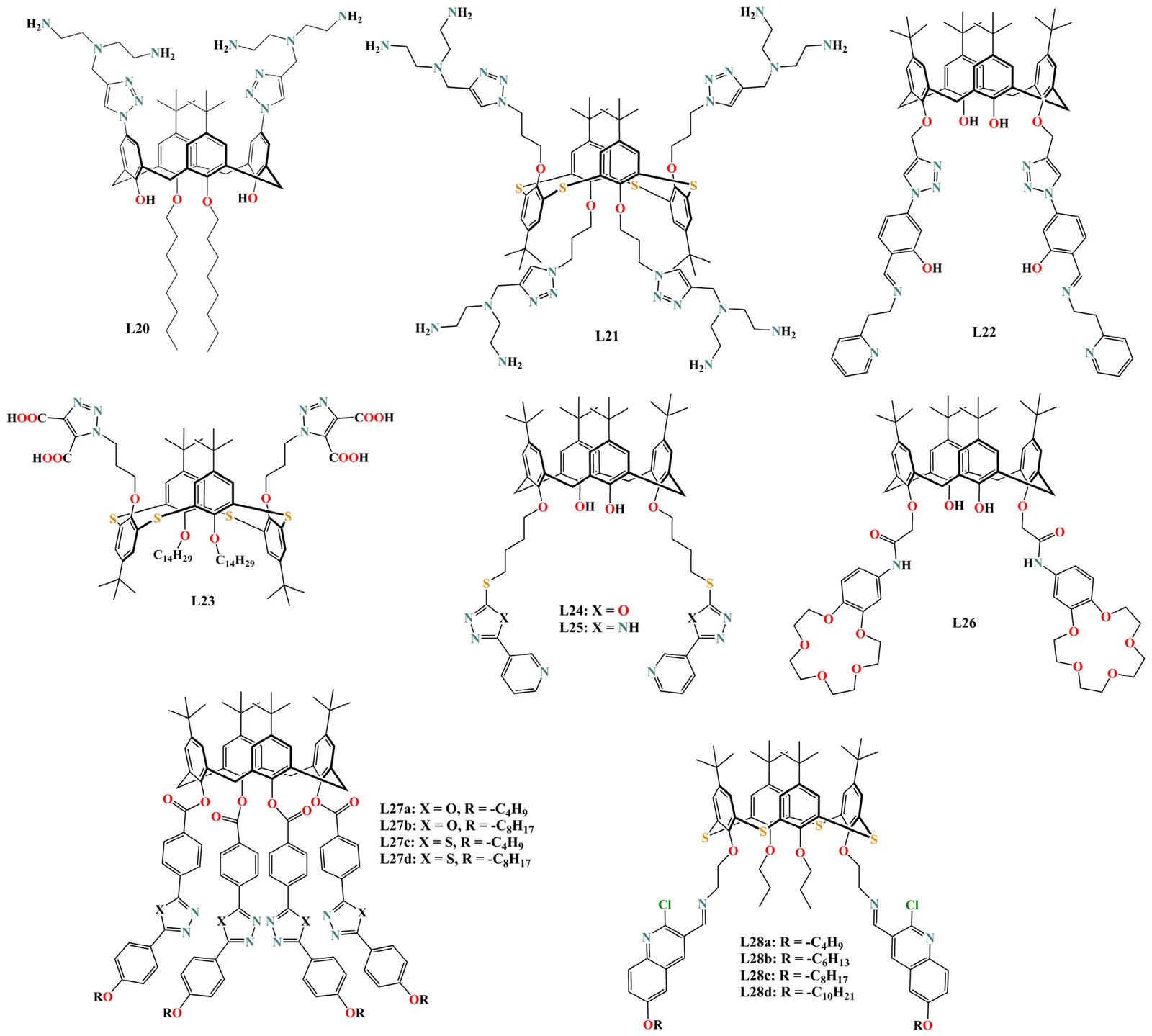
Structures of **HetCA**s **L20**–**L28** for organic molecule complexation.

**HetCA**s can bind to both ionic and molecular compounds, allowing them to be used for extracting organic dyes and for selectively recognizing drugs. **HetCA**s have consequently been explored as promising scaffolds for the development of optical and electrochemical sensors for biologically active compounds. For instance, fluorescent receptors based on modified calixarene platforms have demonstrated effective recognition of carboxylate-containing substrates through cooperative host–guest interactions [629], while electrochemical sensor systems incorporating calixarene derivatives have been applied to the detection of biologically important neurotransmitters such as dopamine [630] (see Section 5.1.4). More broadly, recent studies indicate that the combination of calixarene macrocycles with heterocyclic signaling units enables the detection of structurally diverse analytes (including amino acids, pharmaceuticals, enzymes, and nucleic-acid-related species) and supports their implementation in biosensing and imaging applications [631]. Figure 10 shows the structures of **HetCA**-based receptors for various organic molecules.

**HetCA**s designed for the recognition of neutral molecules generally differ from ion receptors by the use of longer linkers and the incorporation of both hydrogen-bond donors and acceptors, enabling complementary interactions with neutral substrates. The use of heterocyclic moieties such as quinolones (as in the case of **L28**) adds π-π-stacking interactions with organic substrates, including in vivo experiments.

Macrocycles that have been modified with triazine fragments can also interact with organic dyes [176]. There are also examples in the literature of creating indicators for organic compounds. For instance, a colorimetric sensor **L20** based on thiacalix[4]arene was developed that allows ATP and ADP to be distinguished with the naked eye [338]. Actually, for **HetCA**s, many cases of binding to DNA in buffer solutions have been described. Thus, works [341,434] demonstrated the binding in DNA grooves of triazole-based calixarene **L21** modified with ammonium nitrogen atoms obtained by azide–alkyne cycloaddition.

A promising direction is the production of organometallic complexes based on calixarenes modified with heterocyclic fragments, which act as indicators for certain substrates. For example, zinc- and silver-containing macrocyclic fluorescent complexes were obtained that selectively bind to cysteine, which acts as a fluorescence quencher [257,419,421], as well as Zn(II) complexes that selectively bind pyrophosphate ions [420], and Cd(II) complexes selectively reacting to dihydrogen phosphate anions [357] and cysteine (like **HetCA L22** in Figure 10) [421,632], Mg(II) complex on phosphate ions [633]. Of particular interest were rhodium(III) and iridium(III) complexes of calix[4]arene, which had a high association constant with potassium ions (logK = 8–12 in methylene chloride) [634]. Rao et al. have also reported the synthesis of ligands of a similar nature, whose complexes with Ni(II), Zn(II), and Cu(II) cations are capable of selectively binding to asparagine, glutamine, cysteine, and histidine [417,635], and ATP [418]. This research group has also developed macrocyclic ligands containing pyridine and triazole fragments. These ligands formed complexes with Zn(II) cations and could selectively bind pyrophosphate ions and ATP within HeLa cells [423].

Over the last decade, one of the main areas of focus has been the creation of polymer absorbents based on **HetCA**s. For example, T. Skorjanc et al. [636] have reported the production of calixarene polymers based on viologen derivatives that are capable of capturing dyes such as Congo red. Li et al. have demonstrated that polymers based on calixarene containing a triazine fragment are capable of extracting methylene blue and toluidine blue from water [637]. Using heterocyclically modified thiacalix[4]arenes as “antennas” for terbium(III) cations enabled the development of a pyridoxine hydrochloride sensor. This biologically significant molecule, in its cationic form, can displace the lanthanide cation from the calixarene **L23** complex and quench fluorescence. A response to vitamin B6 (7 μM) was therefore recorded in the presence of biologically significant molecules such as amino acids, adenosine phosphates, amines, quaternary ammonium compounds and sugars in 100-fold excess [330].

Organic dyes and drugs are some of the biggest environmental problems, as they are among the most common pollutants in wastewater [638]. To date, the absorption and extraction of dyes from water is considered one of the most convenient and in-demand methods [639]. The mechanisms of organic compound transfer necessarily include the formation of supramolecular structures, which are held together by electrostatic interactions, hydrogen bonds, coordination with heteroatoms, and π-π stacking [638,640]. Evidently, heterocycles can act as effective binding sites for organic dyes and drugs, which are often condensed heterocyclic systems themselves. Calixarenes have proven to be effective extractants [641,642]. The structures of **HetCA**s that act as extractants are quite diverse. Thus, calix[4]arenes **L24** and **L25** modified with oxadiazole and triazole fragments are capable of binding methyl orange [226].

Calixarenes functionalized with crown ether fragments represent an important class of hybrid macrocyclic receptors combining the preorganized calixarene cavity with the well-known cation-binding properties of crown ethers. Such systems exhibit enhanced affinity and selectivity toward alkali and alkaline-earth metal ions due to cooperative host–guest interactions involving both macrocyclic subunits. As a result, crown-ether-modified calixarenes are widely investigated as efficient ligands, ionophores, and sensing components in supramolecular and coordination chemistry. For instance, calixarenes **L26** that are modified with benzene-15-crown-5 fragments can bind to dyes such as methyl orange, brilliant green, and neutral red [150].

**HetCA**s may be of interest not only as a source of ligands for various types of substrates, but also in their own right for the production of optically interesting materials. Photoactive materials that can act as organic diodes and be used as photosensitizers in photovoltaics are also created based on **HetCA**s [285,497,643]. Blue light-emitting supramolecular liquid–crystalline materials based on **HetCA**s functionalized with 1,3,4-oxadiazole and 1,3,4-thiadiazole fragments was synthesized by Sharma et al. The thiadiazole-containing derivatives **L27** exhibited enhanced stabilization of the hexagonal columnar mesophase over a broad temperature range, whereas the oxadiazole analogs showed deeper blue emission in solution and thin films. Owing to their ordered columnar organization and strong emissive properties, these materials are promising candidates for applications in optoelectronic devices, particularly OLED technologies [289]. Rathod et al. reported that thiacalix[4]arenes **L28** functionalized with quinoline moieties and peripheral alkoxy chains were synthesized and thoroughly characterized. The authors demonstrated that the length and position of the alkoxy substituents strongly influence the mesomorphic behavior and the type of liquid–crystalline phases formed. The obtained compounds exhibited blue fluorescence in both solution and solid thin films due to the presence of quinoline units, which also enhance intermolecular interactions. Additionally, these fluorescent materials were successfully applied for staining model nematode organisms [416].

One of the key areas of research in materials science based on calixarenes in recent years has been the development of solar cells [644]. As a rule, photosensitizer moieties are covalently attached to a macrocyclic platform. This ensures greater stability of the resulting calixarene-based photosensitizer derivatives compared to free organic dyes, due to better bonding with carriers such as titanium dioxide [301] or indium–tin oxide [241]. There is also a relatively high level of efficiency in converting solar energy into electricity. Other advantages include an increased short-circuit current and an increased number of cycles during which the solar cell retains at least 90% of its efficiency. Synthetic circuits are also simple [300]. Additionally, the use of a supramolecular approach facilitates the selection of conditions for the location of the macrocyclic photosensitizer, enabling more precise adjustment of the properties of the resulting solar cells [235]. Using calixarenes with different substituents enables the introduction of various heterocyclic fragments, which increases the range of electromagnetic waves absorbed [299].

The reported studies demonstrate that **HetCA** derivatives can interact with a wide variety of neutral organic molecules, including dyes, nitroaromatic compounds, pharmaceutical agents, nucleotides, and biomacromolecules. A broad range of heterocyclic fragments (such as triazine, phenanthroimidazole, benzimidazole, BODIPY, carbazole, naphthalimide, phthalimide, tetrathiafulvalene, and triazole) has been employed to construct receptors with tailored recognition properties. These systems exhibit moderate to relatively strong binding affinities depending on the nature of the guest and the structural features of the receptor. As in many other recognition systems based on calixarene scaffolds, optical detection techniques, particularly fluorescence and UV-Vis spectroscopy, remain the dominant methods used to investigate host–guest interactions, while the formation of complexes most commonly follows 1:1 or 1:2 stoichiometry. The studies are typically conducted in organic solvents, mixed solvent systems, or buffered aqueous media, reflecting the diverse environments required for the recognition of different organic substrates. Table S16 shows the summarized data of **HetCA**s used as platforms for the detection and binding of structurally diverse organic guests [73,150,176,335,443,466,491,494,504,505,581,645,646,647,648,649].

Thus, **HetCA**s play a significant role in forming complex compounds with various substrates, including cations, anions, and neutral organic molecules such as dyes and pharmaceuticals. This can be exploited for the detection of certain compounds with a sufficiently low detection limit for use in analytical and biomedical applications, as well as for the extraction of pollutants from aqueous solutions. The incorporation of diverse heterocyclic fragments into the calixarene framework provides additional donor sites, signaling units, and structural elements that significantly expand the range of detectable substrates. Across the reported examples, optical techniques (fluorescence and UV-Vis spectroscopy) remain the most widely employed methods for investigating host–guest interactions, reflecting the continuing demand for sensitive and selective chemosensing systems. At the same time, the gradual increase in structural complexity of the receptors, achieved through the combination of heterocycles with fluorophores, redox-active units, or biomolecular fragments, enables fine control over binding strength, selectivity, and signal transduction. Taken together, these results highlight the flexibility of **HetCA**s as adaptable platforms for molecular recognition and sensing applications and indicate significant opportunities for further development of multifunctional supramolecular systems based on these macrocyclic scaffolds.

#### 5.1.4. Electrochemical Sensors Based on Heterocycle-Containing Calixarenes

**Figure 11:**
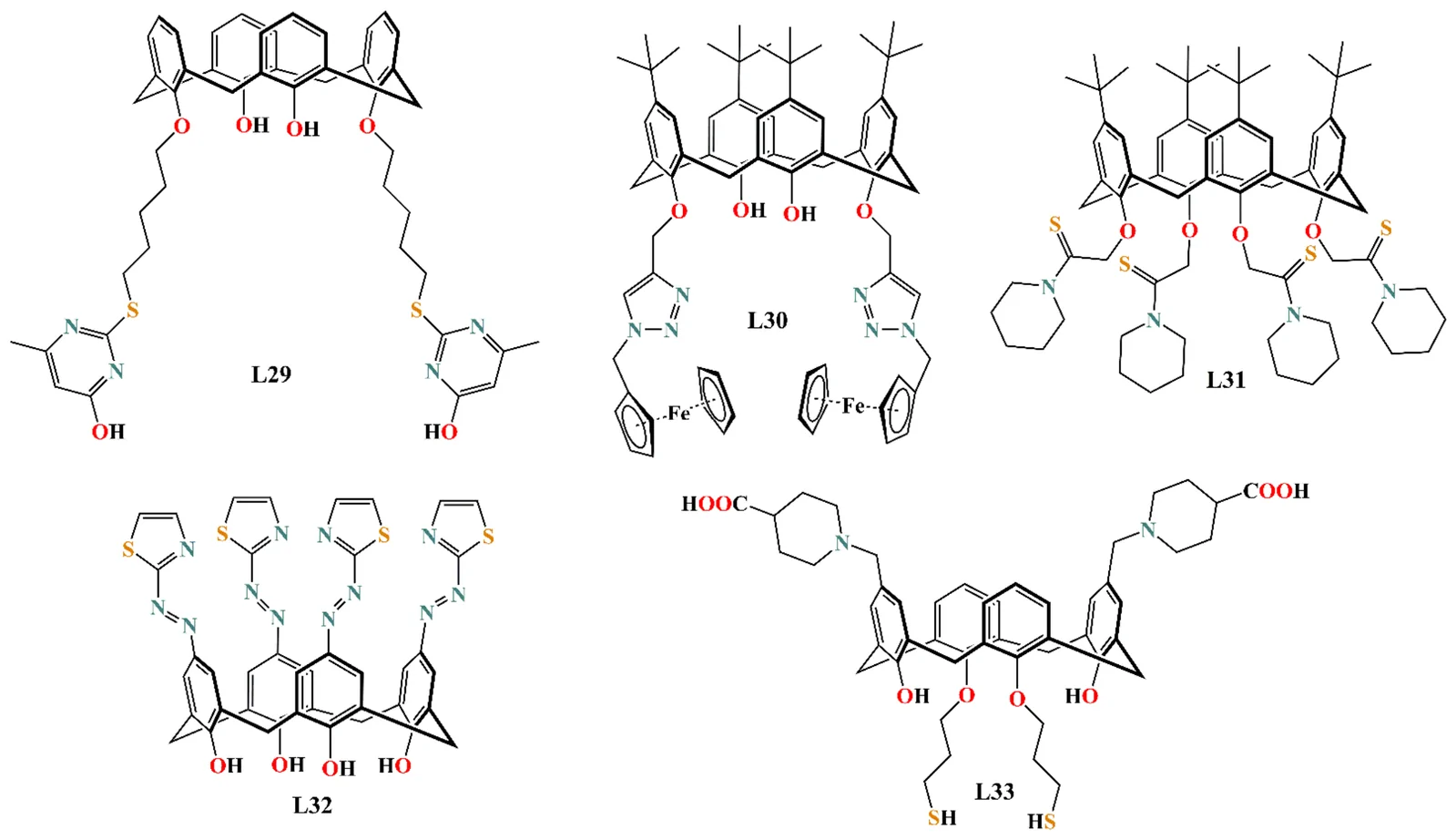
Structures of **HetCA**s **L29**–**L33** with electrochemical sensory application.

Calixarenes are increasingly being used in the field of electrochemical research, particularly for energy storage [650], creation of biosensors [651,652], development of electrochemically active nanoparticles [556], etc. **HetCA**s have been used in electrochemistry for over three decades [653,654]. They are used in electrochemical analysis to coat electrodes to detect different kinds of substrates, for example, silver(I) [655,656,657,658,659,660,661,662], mercury(II) [361,663], copper(I) [664], lead(II) [665,666,667], nickel(II) [668], scandium(III) cations [236] and even alkali metals cations [580] and organic molecules like dopamine [630]. Figure 11 shows the structures of **HetCA**s **L29**–**L33** used as part of electrochemical sensors.

Silver(I) cations are among the most commonly detected species using electrochemical methods with **HetCA**s. Lu et al. developed a series of ion-selective electrodes for silver(I) cations, using **HetCA L29** as an ionophore. Solid-state ion-selective electrodes for silver ions were constructed using a PVC-based membrane incorporating **HetCA L29** as the active ionophoric component. The developed sensor exhibited a near-Nernstian potentiometric response over a broad concentration range, accompanied by a low detection limit and rapid stabilization of the potential. The electrode demonstrated good operational stability, satisfactory selectivity toward Ag(I) in the presence of competing metal ions, and reliable performance within a moderately wide pH window, indicating its suitability for practical analytical applications [657].

Saravanan et al. from the University of Madras (India) synthesized new ferrocenyl-modified **HetCA**s via a CuAAC reaction. The obtained macrocyclic ferrocenyl derivatives (e.g., **HetCA L30**) demonstrated selective electrochemical sensing of Hg(II) ions at low concentrations, even in the presence of competing metal ions, as confirmed by cyclic voltammetry and NMR titration experiments [361].

An adsorptive stripping voltametric approach for Pb(II) determination was developed by Torma et al. using a **HetCA L31**-based chemically modified bismuth-film electrode deposited on a glassy carbon substrate [667]. The method relies on the preconcentration of lead ions followed by simultaneous anodic stripping detection and in situ formation of the bismuth film, providing favorable analytical performance. Under optimized conditions, the sensor exhibited good linearity, high precision, a low detection limit, and effective discrimination of Pb(II) in the presence of excess competing metal ions. The practical applicability of the proposed system was confirmed by the successful determination of trace lead in environmental water samples.

A nickel(II)-selective potentiometric sensor was developed using thiazole derivative **HetCA L32** as an ionophoric component incorporated into a PVC-based membrane. Calixarene **L32** is a relatively rare example of a macrocyclic derivative with a modification on the upper rim. The developed electrode exhibited a near-Nernstian response over a wide concentration range, along with a low detection limit and rapid response time. The sensor demonstrated stable performance within a broad pH window, good operational lifetime, and high selectivity toward Ni(II) ions in the presence of various competing mono-, bi-, and trivalent cations. Its practical applicability was confirmed in potentiometric titrations and in the determination of nickel ions in real samples [668].

**HetCA**s may even be capable of performing electrochemical analysis of organic molecules, such as dopamine. An electrochemical sensing platform based on an ion-channel mechanism was developed by Kurzątkowska et al. to study the interaction between functionalized **HetCA L33** mercaptoalkyl derivatives and dopamine at the aqueous/organic interface [630]. Several upper-rim-modified calix[4]arene derivatives were synthesized, characterized, and immobilized on gold electrodes via covalent Au-S bonds to form sensing interfaces. The formation of host–guest complexes with dopamine was confirmed by square-wave voltammetry using a redox marker, and the sensors enabled highly sensitive dopamine detection over a broad concentration range with a picomolar detection limit. The proposed systems also demonstrated good selectivity in the presence of common interfering biomolecules, indicating their suitability for analytical applications.

A review of **HetCA** sensory systems reveals a clear correlation between receptor structure and its analytical characteristics:

- The type of heterocycle determines the signaling mechanism: nitrogen-containing heterocycles (pyridine, imidazole, triazole) act as donor centers for metal cations, whereas conjugated heterocycles (BODIPY, naphthalimide, rhodamine) act as chromophores/fluorophores sensitive to changes in electron density during complex formation [73,220,496].
- The number and relative arrangement of heterocycles directly influence the stoichiometry and cooperativity of binding. For example, the distal 1,3-arrangement of heterocycles in **HetCA**s most often leads to the formation of 1:1 complexes, whereas the proximal 1,2-arrangement or tetrasubstitution promotes the formation of binuclear (1:2) or polynuclear complexes, which manifests as nonlinear dependencies in titration curves [185,593].
- The nature of the linker between the macrocycle and the heterocycle modulates flexibility and, consequently, entropy losses upon binding. Rigid (aryl, amide) linkers increase selectivity through preorganization, while flexible (aliphatic, polyethylene glycol) linkers improve the kinetics of complex formation and broaden the range of detectable substrates [73,145].
- The conformation of the macrocycle plays a decisive role in determining the size and shape of the binding pocket. The *cone* conformation creates a cone-shaped pocket that is optimal for binding metal cations, whereas *1,3-alternate* conformation forms a more open platform suitable for binding anions or neutral molecules [90,136,206].

Overall, the summarized data demonstrate that **HetCA**-based ion-selective systems provide reliable and efficient platforms for the potentiometric detection of metal cations, with silver ions being among the most extensively investigated targets. The reported sensors generally exhibit near-Nernstian responses over relatively broad pH windows and maintain stable analytical performance across wide concentration ranges. A key advantage of these systems is their good selectivity toward target ions in the presence of large excesses of competing alkali, alkaline earth, transition, and heavy metal cations, which is reflected by favorable selectivity coefficients. Table S17 contains more detailed analysis [580,630,655,656,657,658,659,660,661,662,663,665,666,667,668].

### 5.2. Catalytic Application of Heterocycle-Containing Calixarenes

**Figure 12:**
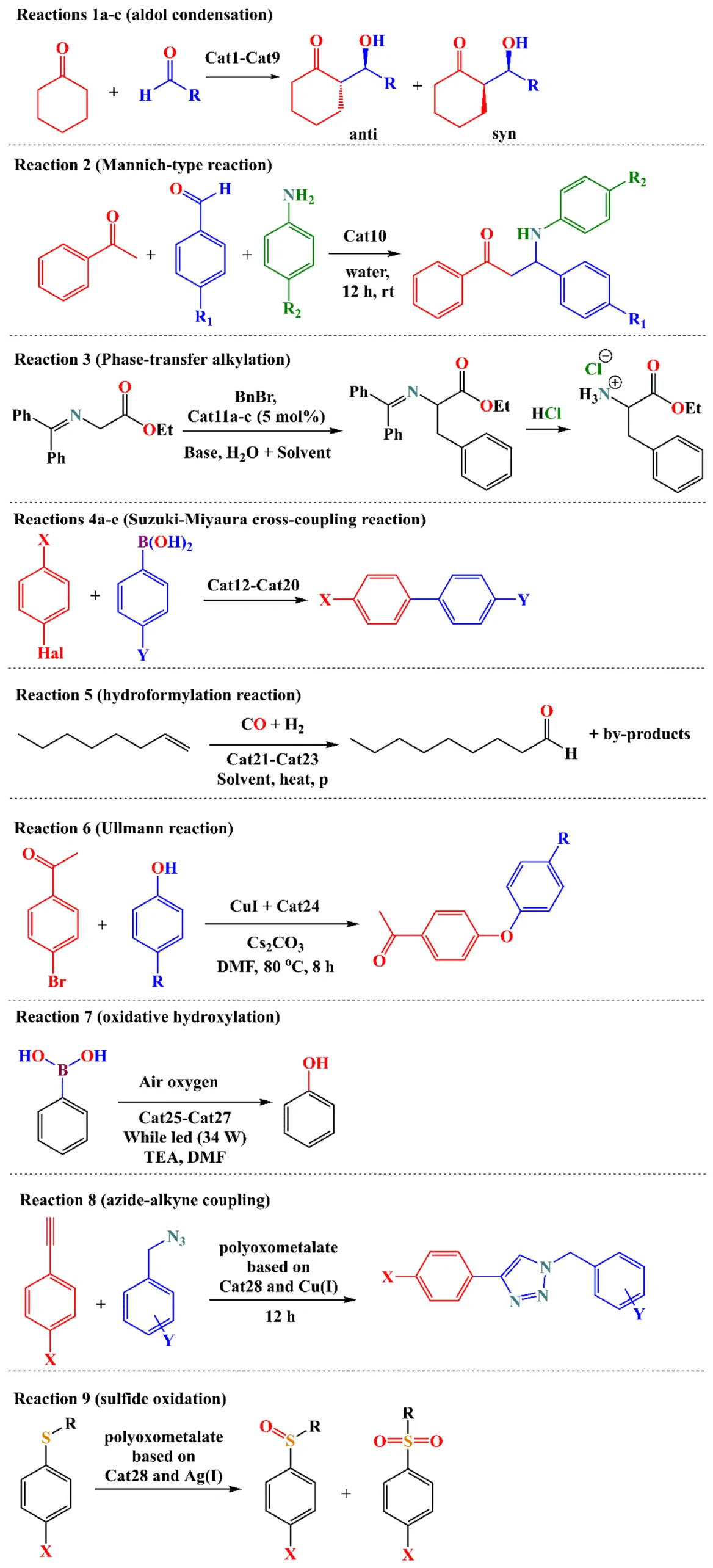
Reactions catalyzed by **HetCA**s.

**Figure 13:**
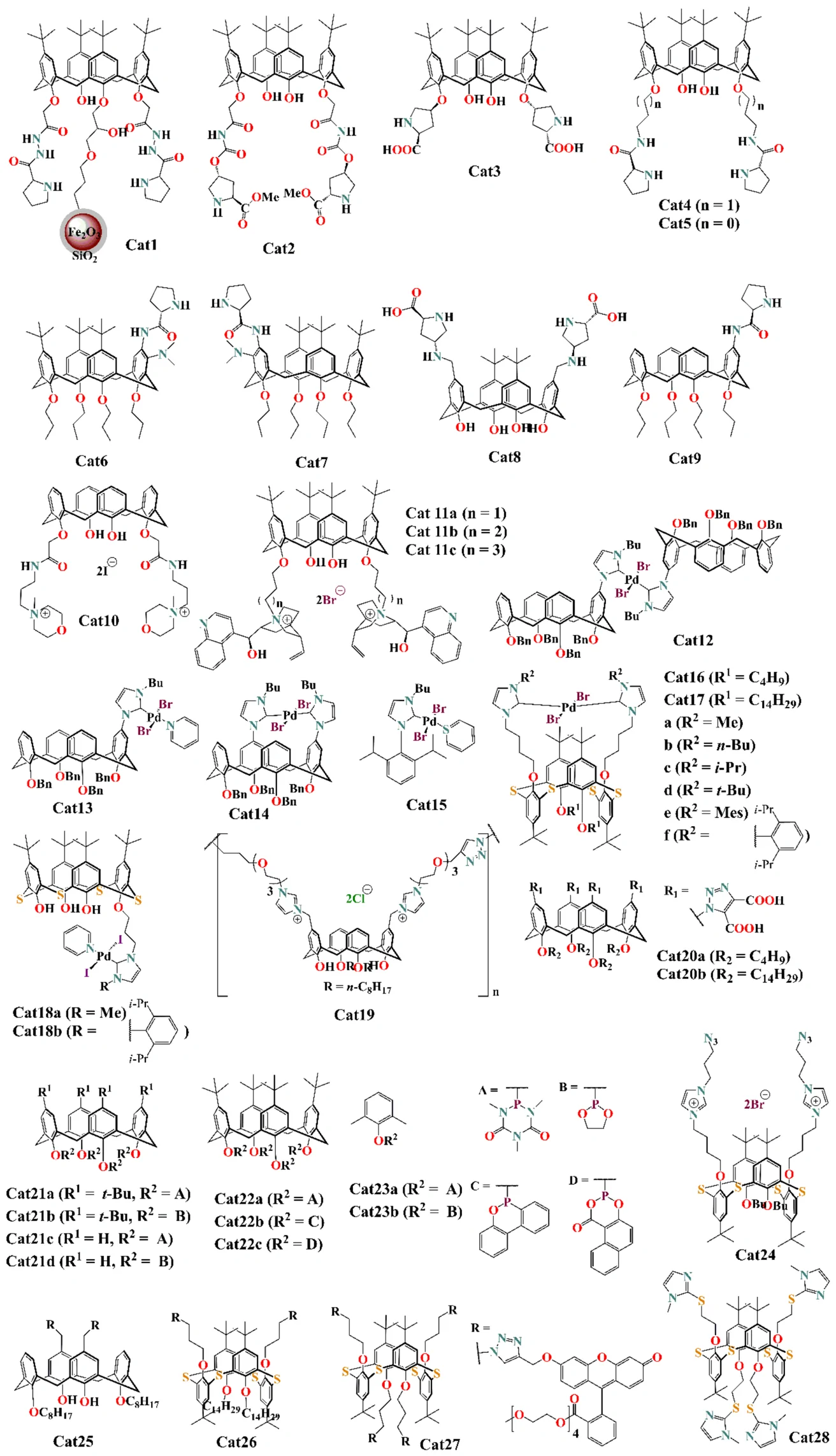
Structures of (thia)calixarenes **Cat1**–**Cat28**.

Calixarenes are third-generation host molecules [669]. They emerged in organic synthesis and supramolecular chemistry some time ago and have become one of the classic examples of host molecules. It would be difficult to imagine modern nanochemistry and nanotechnology without them [670,671]. However, calixarenes and their derivatives, such as thiacalixarenes, still have a lot of potential for producing the latest 21st-century materials. This section discusses the use of calixarenes as catalysts for some of the reactions shown in Figure 12. For ease of orientation in this section, we have introduced separate numbering for the catalyst structures (like **CatN**) shown in Figure 13.

Due to their rigid framework and ability to recognize and bind to specific substrates, calixarenes can act as catalysts in various chemical reactions [460,470,670,672,673]. Calixarene cavities can act as analogs of natural enzyme cavities by either locally increasing the concentration of reagent molecules within them, or arranging them in such a way that the activation energy of the transition state is reduced. This “cavity effect” arises from a combination of entropic and electronic factors. From an entropic perspective, a preorganized and relatively rigid cavity can reduce the conformational freedom of the substrates and catalytic sites, thereby decreasing the entropic penalty associated with their organization within the reactive environment. From an electronic perspective, the cavity creates a distinct microenvironment in which aromatic walls can participate in cation–π and C–H-π interactions, while heterocyclic substituents modulate the local electronic environment, and desolvation upon binding can alter the effective dielectric properties. The combined effects may favor substrate preorganization and transition-state stabilization, thereby enhancing catalytic activity and selectivity relative to less organized or acyclic analogs. The catalytic activity of **HetCA**s is enabled by the combination of a rigid, preorganized macrocyclic scaffold with appropriately positioned heterocyclic, donor, and metal-containing functionalities. The macrocyclic architecture provides a defined three-dimensional environment that can preorganize and concentrate substrates through coordination, hydrogen bonding, electrostatic, and π-π interactions, while N-, O-, and S-donor heterocyclic fragments serve as coordination sites for metal centers and allow their electronic properties to be tuned. Variation in the number and spatial arrangement of substituents, macrocycle conformation, linker length and flexibility, and steric bulk provides control over the accessibility and geometry of the catalytic sites of **HetCA** derivatives. In addition, the incorporation of multiple coordinating groups or metal centers can promote cooperative or bimetallic catalysis, whereas hydrophilic, ionic, or polyether substituents can improve solubility and enable catalysis in polar or aqueous media. Thus, the structural versatility of **HetCA** systems allows simultaneous control over substrate recognition, catalyst preorganization, steric environment, and electronic properties of the active center, making these macrocycles effective platforms for the design of tunable supramolecular and metal-based catalysts.

The range of reactions that can be catalyzed by **HetCA** is quite broad. For example, several studies [142,240,258,291] have described **HetCA**s (**Cat1**–**Cat6**) containing pyrrolidine moieties that can catalyze the aldol condensation reaction (Figure 12, reactions 1a–c). In particular, Aktas et al. obtained a catalyst for asymmetric aldol condensation based on *L*-proline. For example, the authors synthesized (*S*)-2-((*R*)-hydroxy(2-chlorophenyl)methyl)cyclohexan-1-one with a yield of 94% and an enantiomeric excess of 88% [291]. Similar studies involving macrocyclic catalysts containing *L*-proline fragments for aldol condensation have been conducted by research groups such as Xu et al. in Beijing [304] and Li et al. in Nanjing [674,675]. For more detailed information on the conditions for conducting model catalytic aldol condensation reactions, see Tables S18–S20.

In addition, Akceylan and Yilmaz report on calixarene **Cat10**, which contains quaternized morpholine fragments capable of catalyzing the Mannich-type reaction (Figure 12, reaction 2) [252]. The reaction is carried out in a three-component system, during which acetophenone, benzaldehyde, and aniline form Mannich-type adducts with yields ranging from 48 to 65% (Table S21).

A particularly noteworthy example of the synergy between synthetic calixarene chemistry and natural compound chemistry is the work of Bozkurt et al. [471]. Using the Menshutkin reaction, researchers obtained cinchonidine-modified calixarenes **Cat11a**–**c**. These compounds exhibit asymmetric phase transfer properties in the catalytic alkylation reaction of benzophenone glycine ester, resulting in the formation of phenylalanine hydrochloride (Figure 12, reaction 3, see also Table S22).

Of particular interest are macrocyclic complex catalysts for the Suzuki–Miyaura reaction (Figure 12, reaction 4a), **Cat12**–**Cat15,** containing transition metal ions such as palladium as the metal center, obtained, for example, by a group of French authors (Table S23) [440]. Additionally, a group led by V. Burilov and I. Antipin from Kazan Federal University (Russia) obtained complex palladium catalysts based on imidazolium *p*-*tert*-butylthiacalix[4]arenes for the Suzuki–Miyaura reaction [429,445], similar to the derivatives previously obtained by T. Falbusch and colleagues [427]. Researchers from Kazan have achieved a yield of ready-made catalysts ranging from 50 to 99% (Table S24). The resulting macrocyclic derivatives **Cat16**–**Cat18** exhibited high catalytic activity in the cross-coupling of phenylboronic acid and various aryl halides (Figure 12, reactions 4b and 4c, Table S25). In some cases, the conversion rate for the macrocyclic catalyst was notably higher than for the standard palladium catalyst [429,445]. An interesting modification of calixarene–palladium complexes is presented, in which a polymer formed through azide–alkyne coupling is employed in place of a monomeric macrocycle such as compound **Cat19** [437,438]. In particular, it has been demonstrated that the Suzuki–Miyaura reaction (Figure 12, reaction 4d), which is catalyzed by palladium(II) acetate, is more complete in the presence of a calixarene polymer **Cat19** (Table S26) [437]. Macrocyclic 1,2,3-triazole derivatives (such as compounds **Cat20a**–**b**) have generally proven to be highly effective (Table S27) for the preparation of palladium-containing catalysts for the Suzuki–Miyaura reaction (Figure 12, reaction 4e) [340].

The work of German researchers [208] vividly illustrates the thesis that macrocyclic catalysts are more effective than their monomeric analogs. Thus, the authors synthesized a series of ligands containing phosphorus-containing nitrogenous heterocycles, biuret derivatives and cyclic phosphite fragments, including calix[4]arene derivatives and monomeric compounds. Rhodium catalysts based on **Cat21**–**Cat23** were synthesized, and it was demonstrated that, in the case of macrocyclic structures, hydroformylation of oct-1-ene (Figure 12, reaction 5) proceeded more regioselectively than with monomeric catalysts, with n-nonanal predominating as the product (Table S28). The authors attributed this selectivity to the rigid preorganization of the calixarene structure. Similar studies were conducted on calixarenes containing other phosphorus-containing heterocyclic fragments [209]. It is also worth noting that the X-ray crystallography of the obtained complexes was studied in a separate piece of work, which demonstrated the planar coordination geometry of rhodium calixarene complexes [676].

Calixarenes that have been modified with heterocyclic fragments can act as catalysts not only for the Suzuki reaction, but also for other types of cross-coupling reactions, such as the Ullmann reaction (Figure 12, reaction 6) [432]. In this study, calixarene **Cat24** acts as a co-catalyst alongside CuI, enhancing the conversion rate of the reaction between phenols and bromobenzenes to nearly 100% in certain instances (Table S29). The authors explain this catalytic effect by proposing that micelle microreactors are formed, within which the molecules can interact more effectively with each other.

In recent years, green technologies for capturing carbon dioxide have been developed to promote sustainable development and reduce the impact of the greenhouse effect. Covalent organic polymers based on calixarenes containing imidazolium fragments can be used as carbon dioxide binding catalysts. These polymers offer significant advantages over conventional catalysts as they do not contain transition metal cations and are more resistant to physical stress. For example, Zhang et al. published the production of a calixarene polymer capable of effectively catalyzing the cycloaddition of carbon dioxide to oxiranes (epoxides) with quantitative yields in 2019 [677].

Effective photosensitizers can also be obtained from calixarene molecules that have been modified with heterocyclic dye molecules [678,679,680]. Conjugates of calixarenes and fluorescein **Cat25**–**Cat27** have been shown to be catalytically efficient in the oxidative hydroxylation of phenylboronic acid in a model reaction 7 (Figure 12), which is often used to evaluate the photosensitization efficiency of various processes in water due to the good solubility of phenylboronic acids and their oxidation products, phenols. Photocatalyst **Cat26** yielded the best result, producing more than 90% phenol after 10 h (Table S30). Another advantage of these macrocyclic catalysts is that they are free from metals, which are expensive and harmful to the environment [681,682].

Catalyst structures can be even more complicated. For example, they can belong to the complex polyoxometalate class [224]. For example, a copper-containing polyoxometalate catalyst was obtained from thiacalixarene **Cat28**, copper(I) chloride and silicotungstic acid [225]. The resulting material proved to be an effective catalyst for azide–alkyne coupling (Figure 12, reaction 8, see also Table S31). The silver-containing complex based on **Cat28** was ineffective in catalyzing the azide–alkyne addition reaction, but was found to promote the oxidation of organic sulfides to sulfoxides and sulfodioxides (Figure 12, reaction 9). This ability can be exploited to remove sulfide compounds from oil (Table S32) [225].

The **HetCA** catalytic systems examined demonstrate that their efficiency is determined not only by the catalytic center but also by the supramolecular environment:

- Heterocycles as ligands for metals: pyridines, imidazoles, 1,2,3-triazoles, and phosphorus-containing heterocycles serve as effective ligands for Pd, Rh, Cu, and other metals. Their chelating ability and capacity to stabilize the metal in various oxidation states form the basis of their catalytic activity [208,427,440,445].
- The role of the macrocyclic framework: the calixarene framework acts as a platform for creating a local high concentration of reactants and as a “molecular scaffold” that anchors ligands and substrates. This “cavity effect” often leads to increased regio- and enantioselectivity compared to monomeric analogs [142,208,240,304].
- Synergy between substituents: catalysts containing both metal-binding and non-coordinating (e.g., hydrophilic) groups exhibit improved properties. For example, the presence of amphiphilic moieties in **HetCA** allows for the creation of micellar microreactors that significantly accelerate reactions in aqueous media (Suzuki and Ullmann reactions) [432,450].

The incorporation of heterocyclic fragments expands the scope of calixarene catalysis by introducing additional coordination sites, catalytic centers, and opportunities for fine-tuning the electronic and steric properties of the catalyst. In summary, **HetCA**s constitute a versatile and rapidly developing class of catalytic systems that combine the advantages of a preorganized macrocyclic scaffold with the functional diversity of heterocyclic substituents. The examples discussed in this section demonstrate that these compounds can efficiently catalyze a broad range of transformations, including aldol and Mannich reactions, asymmetric phase-transfer processes, cross-coupling reactions, hydroformylation, cycloaddition of carbon dioxide to epoxides, photocatalytic oxidations, and azide–alkyne coupling reactions. In many cases, the catalytic performance of macrocyclic systems surpasses that of analogous monomeric catalysts owing to substrate preorganization, local concentration effects, and the unique microenvironment created by the calixarene cavity.

### 5.3. Biomedical Application of Heterocycle-Containing Calixarenes

#### 5.3.1. Biological Activity of Heterocycle-Containing Calixarenes

Heterocyclic fragments form the structural basis of many biologically active compounds and drugs. In fact, statistics show that more than 85% of drugs contain these fragments in their composition [683]. Heterocycles are also often considered to be privileged structures. Conversely, macrocyclic platforms are being used more and more to create biologically active compounds, including antimicrobials and anticancer drugs [391,684,685,686,687,688,689]. Professor P. Cragg of the University of Brighton (UK) notes in his book, “Supramolecular Chemistry: From Biological Inspiration to Biomedical Applications” [690], that life itself can be viewed as a supramolecular phenomenon. All biological systems at the molecular level involve molecular recognition and self-assembly through the formation of multiple non-covalent interactions. One example is the double-stranded structure of DNA, which is held together by hydrogen bonds that maintain the shape and size of the spiral. Similarly, the living cell itself is also a product of self-assembly, with its bilipid membrane, endoplasmic reticulum, and organelles. As the title of P. Cragg’s book [690] suggests, supramolecular chemistry has several levels of application in biomedical research. The first level involves using macrocyclic platforms as models of biological compounds that are easily synthesized [631,691]. Studying such interactions at the molecular level opens new avenues for creating effective therapeutic agents. The next level of supramolecular chemistry application involves using macrocyclic structures to carry biologically active compounds to specific locations, as supramolecular structures based on macrocyclic platforms can improve drug bioavailability [50,74,692,693,694,695]. Additionally, the macrocycle itself can act as a drug due to its structural basis as a potential drug that does not contain easily hydrolyzable ester fragments. For example, sugammadex, a derivative of cyclodextrin and a well-known antagonist of neuromuscular blockers, is a macrocycle. For instance, studies have shown that thiacalixarens, which contain an *L*-tryptophan fragment and a quaternized nitrogen atom, effectively inhibit chymotrypsin [452]. Recent studies of the properties of such structures have shown their high potential against bacterial strains (MIC = 0.5–32 μM), including *MRSA* (MIC = 3.9–7.8 μM) [696]. In particular, among (thia)calixarenes, there are also a number of patented anticancer drugs with proven efficacy [697,698,699].

Combining heterocyclic structures with (thia)calixarenes is promising for producing new biologically active materials because it combines the main advantages of macrocycles and heterocyclic fragments. Specifically, combining a macrocyclic platform with heterocyclic fragments enables the production of a fluorescent dye for staining nematodes [290], an anticancer drug [700,701,702], or a nucleic acid sensor [243,334].

One of the most common approaches to using calixarenes in drug production is to synthesize prodrugs—compounds that lack pharmacological activity but are metabolized into active compounds. Thus, in the works of Regnouf-de-Vains [74,133,228,703,704], calixarenes modified with nalidixic acid, a derivative of the quinoline heterocycle, were obtained. In vitro evaluation of their antibacterial activity demonstrated that these conjugates are effective against both Gram-negative (*E. coli*) and Gram-positive (*S. aureus*) strains, showing a significant increase in activity compared to the two products resulting from their hydrolysis [703]. The resulting macrocyclic derivatives exhibited activity against *S. aureus* when combined with a fragment of penicillin V [74]. Using a similar approach, the same research group obtained compounds with antiviral activity. They synthesized prodrugs based on calix[4]arene that contain one acyclovir fragment and are capable of releasing the drug [705].

Another approach to drug delivery systems exists as well [706]. A recent article [303] by researchers from Nankai University (China) reports on the production of calix[4]arene modified with a complex fragment such as biotin (also known as vitamin B7). The resulting calixarene has an affinity for receptors on tumor cells in the lungs, kidneys, and ovaries of mice and acts as a molecular container for doxorubicin. In recent decades, research into the interactions between calixarenes and their derivatives modified with heterocyclic fragments and malignant cells has become mainstream [707]. The resulting complex, which is based on calixarene and biotin and non-covalently binds doxorubicin, demonstrated high efficacy in vivo. As the authors expected, this carrier may be useful for other types of therapy, such as photodynamic therapy.

The 1,3,4-oxadiazole fragment belongs to promising pharmacophore groups that are bioisosteres of amides and esters and are part of biologically active compounds with antimicrobial [708], antiviral [709], cytostatic [710], antitumor [711], anti-inflammatory [712] and other types of activity. The combination of *p-tert-*butylcalix[4]arene and 1,3,4-oxadiazole produced an agent that was fairly effective against both *A. fumigatus* fungi and *E. coli* bacteria (MIC = 10 μg/mL) [134]. Introducing 1,3,4-oxadiazole and thiadiazole rings as pharmacophores into the calix[4]arene structure increased the molecule’s hydrophilicity, facilitating its penetration through the biological membranes of microorganisms and suppressing their growth [713].

Dendrimeric derivatives of calix[4]arene modified with two or six benzotriazole fragments were synthesized using click chemistry approaches [714]. This heterocycle was chosen due to the high biological activity of its derivatives [713]. The authors demonstrated that benzotriazole derivatives of calix[4]arene exhibited high activity against *K. pneumoniae* and an excellent chemosensitizing effect. The compounds were also found to be highly biocompatible in both in vitro and in vivo studies.

Beta-lactam antibiotics are among the most commonly prescribed medications [715,716,717]. By modifying the lower rim of the calix[4]arene with moieties of penicillins V or X [718] and cephalosporins [719], compounds with high antibacterial activity against Gram-positive and Gram-negative bacterial strains (*S. pyogenes*, *S. agalactiae*, *S. pneumoniae*, *S. aureus*) were obtained. The authors also proposed a mechanism of action and evaluated the macrocyclic effect by comparing the biological properties with monomeric analogs.

Many quaternary ammonium salts are biologically active compounds. Numerous publications in the literature are devoted to the synthesis and study of the antimicrobial activity of thiacalixarene derivatives [77,449,720,721,722,723,724,725,726,727]. One of the alternative positively charged fragments with biological activity is the imidazolium group [728,729], whose derivatives are widely used in the synthesis of ionic liquids [730,731]. A synthesis of quaternary ammonium and imidazolium derivatives of calix[4]arene substituted on the upper rim with alkyl substituents of varying lengths on the lower rim was carried out to compare their biological properties [732]. The authors found that macrocycles with propyl substituents on the lower rim and imidazolium fragments on the upper rim exhibited the greatest antibacterial activity, with MIC = 10–50 μg/mL. This was explained by the greater affinity of the lipophilic imidazolium fragments for the bacterial membrane. A similar study examined the synthesis of disubstituted calixarenes with amide groups and various heterocyclic fragments (furan, tetrahydrofuran, morpholine, and pyridine) on the lower rim [733]. These fragments are hydrophilic, and the morpholine and pyridine heterocycles can be protonated in water. This explains why they have good antibacterial activity against bacterial strains. In the works of Regnouf-de-Vains [734,735] on synthesizing calixarenes with lower rim groups containing bipyridine and bithiazole, high antibacterial activity was established only in the case of macrocycles with guanidinium fragments on the upper rim [735]. The obtained results confirm the link between positively charged groups and high biological activity.

Cationic aminoglycosides are known to have an affinity for nucleic acids, including bacterial and eukaryotic RNA and plasmid DNA molecules [736,737,738]. This allows these compounds to be used in developing gene delivery systems [739]. Many aminoglycosides are also antibiotics that target Gram-negative bacteria [740,741]. Tetrasubstituted calix[4]arenes containing amino sugar fragments, such as neomycin, kanamycin, and paromomycin, were synthesized using click chemistry approaches [686]. The authors demonstrated that the obtained compounds effectively bind to DNA, forming positively charged nanoparticles (d = 150 nm, zeta potential = +30 mV). These nanostructures exhibited antibacterial activity against the Gram-negative bacterium *E. coli*.

An attractive direction is the modification of calixarenes with glycosidic residues. The resulting glycoclusters based on calix[4]arenes have antibacterial activity [390], due to their ability to inhibit lectins—proteins that cause cell agglutination by binding carbohydrate residues on their surface to each other. For example, a paper by a large international team of authors was published in which they synthesized calix[4]arene modified with four residues of the iminosaccharide deoxynogirimicin, a known lectin inhibitor [76]. Introducing four fragments into the macrocyclic platform significantly increased the inhibition of certain lectins, particularly α-mannosidases, such as those isolated from Canavalia ensiformis (Jack bean α-mannosidase, JBαMan). Similar work was subsequently carried out by teams from the Czech Republic, Hungary, and France [75,368]. Additionally, A. Palmioli and his colleagues recently demonstrated the effectiveness of calix[4]arene modified with sugar residues on the upper rim against pathogenic *E. coli* strains capable of causing infectious urogenital diseases [384]. The mechanism involves inhibiting the lectin-adhesin FimH, whose molecules are located at the ends of bacteria’s pili and promote binding to the epithelium. This study employed saturation transfer difference (STD) nuclear magnetic resonance (NMR) spectroscopy to determine the inhibition constant and the nature of the binding between the inhibitor and the FimH adhesin. The authors also used X-ray crystallography to determine the structure of several Fim-H–calixarene complexes [742]. Additionally, a joint study published by A. Palmioli, A. Casnati, and their co-authors in 2025 examined the ability of calixarene modified with four proline amino acid residues to detect Gram-negative bacterial cell membranes, such as those of *E. coli* and *P. putida*, by binding to their surface lipopolysaccharides [743].

The human immunodeficiency virus (HIV) and the acquired immunodeficiency syndrome (AIDS) it causes are among the greatest challenges of our time. According to the World Health Organization, nearly 40 million people worldwide will be living with HIV by the end of 2023, nearly two-thirds of whom will be in Africa [744]. Today, the main treatment method is antiretroviral therapy, which enables people to live with minimal restrictions and reduces the risk for those around them. The Regnouf-de-Vains group has demonstrated the effectiveness of water-soluble, negatively charged bis-thiazolyl derivatives of calix[4]arenes against HIV [140].

The SARS-CoV-2 virus, which caused the devastating tragedy of recent years, is the culprit behind the global pandemic of the novel Coronavirus (COVID-19) [745]. However, searching for new antiviral agents remained a scientific priority until 2020 [746]. The group of Prof. R.E. Duval synthesized benzoxazolyl sulfoxal[4]arenes that were substituted at the lower rim. These compounds exhibited high antiviral activity against the human coronavirus 229E (HCoV-229E) [747]. The authors noted that the macrocycles they studied were several times more effective than chlorhexidine.

An extensive dataset on the biological activity of **HetCA**s allows us to identify several key structural motifs responsible for their pharmacological effect:

- Positively charged heterocycles: Imidazolium, pyridinium, and ammonium derivatives exhibit pronounced antibacterial activity due to electrostatic interactions with negatively charged bacterial membranes, as confirmed by MIC data ranging from 0.5 to 32 μM for various strains, including MRSA [77,449,696,721].
- Multivalent heterocyclic pharmacophores (beta-lactams, quinolones, 1,3,4-oxadiazoles and carbohydrate moieties), attached to a macrocycle, exhibit a multivalent effect, significantly enhancing affinity for biomolecular targets (lectins, enzymes) compared to their monomeric analogs [74,75,384,743].
- “Structure–permeability” relationships: The introduction of hydrophilic (e.g., furan, morpholine) or hydrophobic (long alkyl chains) substituents into the **HetCA** structure allows for the tuning of their ability to penetrate biological membranes, which is critical for achieving high bioavailability [733].

Thus, biomedical research on heterocyclic calixarene derivatives shows that these macrocyclic platforms are highly effective biologically active compounds. Heterocyclic motifs have proven effective in low-molecular derivatives such as quinolones, beta-lactams, and 1,3,4-oxadiazoles and act as pharmacophore fragments. Additionally, the development of glycoclusters based on *meta*-cyclophane has paved the way for the creation of new antibiotics that inhibit bacterial lectins. Additionally, it is worth noting that the macrocyclic platform significantly impacts the biological activity of target compounds. Calixarenes modified with heterocyclic fragments show great promise as medicinal drugs and are a promising area of research. This field of research is promising and has the potential to transform treatment approaches and improve quality of life. Table S33 provides a summary of the biomedical applications of **HetCA** derivatives [74,75,76,133,134,140,243,288,303,368,384,391,496,686,703,705,707,713,714,718,719,721,732,733,734,735,747,748,749,750,751,752].

#### 5.3.2. Supramolecular Systems Based on Heterocycle-Containing Calixarenes

Nobel laureate Jean-Marie Lehn defined supramolecular chemistry as “chemistry beyond the molecule” [753]. Supramolecular complexes are based on weak molecular interactions, such as hydrogen bonds, coordination bonds, hydrophobic interactions, and π-π stacking [754]. Two key concepts in supramolecular chemistry are the terms “guest” and “host.” A “host” is a molecule with convergent intermolecular bonds that possesses a cavity in which binding occurs. A “guest” is typically a small molecule with divergent intermolecular bonds. In other words, the host molecule acts as a receptor, or a “lock”, while the guest molecule acts as a substrate, or a “key”, in a “key-lock” analogy.

The study of the association of calixarene derivatives is actively researched in supramolecular chemistry [694,755]. Calixarenes modified with heterocyclic fragments (**HetCAs**) have long been the subject of intense interest for researchers in the field of coordination and supramolecular chemistry [540,756,757,758,759,760,761,762], since such macrocyclic platforms can act as ligands for obtaining various 3D structures, for example, zigzag MOFs [763], tubes [764], gold doped clusters [765], podands with transitive metals cations [166,456,766], agents for membrane transport of cations of various metals [767], etc. Of particular interest is the synthesis of asymmetric calixarenes, which can be used as receptors for chiral substrates [768]. The supramolecular chemistry of aggregates (micelles, vesicles, etc.) formed in solutions is of considerable interest since varying conditions (the solvent nature, concentration and ratio of compounds, and temperature) allow the resulting aggregates to be easily rearranged. Water is of particular interest as a medium because most biological processes occur in aqueous environments. Therefore, studying the behavior of supramolecular associates in this solvent allows us to better understand the course of similar natural processes [769]. The creation of molecular machines that can perform useful work at the nanoscale is the pinnacle of supramolecular and nanochemistry. It is impossible to imagine this without self-assembly, a phenomenon noted in 1996 by future Nobel Laureate Sir J. F. Stoddart [770].

Self-assembly can lead to the formation of regular structures at the level of micelles and small associates. Recently, the authors from the University of Kitakyushu studied the association of calix[4]arenes modified with triazole fragments and long polyethylene glycol tails [771]. These micelles are distinguished by the regular number of molecules that form these aggregates, which coincides numerically with the number of faces in the Platonic solids. Therefore, these authors named these aggregates Platonic micelles [771]. Modern methods, such as dynamic light scattering (DLS) [404,772,773], fluorescence spectroscopy [774,775], conductometry [776], scanning (SEM) and transmission (TEM) electronic microscopy [60,404,777], and atomic force microscopy (AFM) [778] can be used to study supramolecular systems in solutions, in particular those based on calix[4]arenes.

DLS is one of the most common methods in supramolecular chemistry. It is a simple method that requires only a few sample weights. This method is based on several principles, including the Doppler effect and the refractive index and viscosity of the solvent at specific temperatures. The results of the mathematical analysis of the experiment are the hydrodynamic diameter (D) and the polydispersity index (PDI). In general, the associates are considered monodisperse at PDI values less than 0.25 [779]. Associates can form from either molecules of the same nature, in which case we speak of self-association, or from different molecules, in which case the aggregation of two or more components occurs. As a rule, the parameters of the resulting associates are determined by the DLS method in solution, as well as direct electron microscopy methods [780]. The obtained data are then analyzed for convergence. Table S34 shows examples of supramolecular systems based on **HetCA**s [404,441,443,781,782]. As we can see, the associated parameters (D and PDI) were typically studied in water or acetonitrile at temperatures of 20 and 25 °C.

New catalysts for various processes can be obtained, in addition to the use of heterocyclic derivatives of calixarenes in the development of new materials with special physical and technical properties. One such area is the production of liquid crystals, which is the subject of a fairly extensive set of literature [783]. *p*-*tert*-Butyl(thia)calix[4]arenes in the *cone* conformation are most commonly characterized by the conventional discotic columnar (Col) structure [350]. Heterocyclic structures in the composition of macrocycle substituents can play the role of both active binding sites and linker fragments. Thus, in the now practically classic work by Koh et al., stilbene calixarene structures containing a pyridine fragment are reported, which is protonated by a number of *p*-alkoxybenzoic acids (with a hydrocarbon radical containing 12 or more carbon atoms), forming Col-phases [784]. Calixarenes, in general, are likely to form Col_h_-phases, i.e., columnar structures with hexagonal symmetry, as described in a number of studies [205,261,289,416,785,786].

Calixarenes modified with unsaturated heterocycles provide an excellent foundation for producing self-healing materials. Researchers from Isfahan University (Iran) and Drexel University (USA) have developed a new self-healing polyurethane material based on these calixarenes [144]. In polymer synthesis, a bis-furan derivative on a calixarene platform interacts with a polymeric dimaleimide in a Diels–Alder reaction. The resulting elastomers were damaged but demonstrated surface restoration when slightly heated, a phenomenon that the authors attributed to the Diels–Alder reaction and cross-linking at the cut site.

Thus, heterocycle-containing calixarenes represent a versatile class of supramolecular building blocks that combine the structural preorganization of the calixarene platform with the coordination, hydrogen-bonding, and π-interaction capabilities of heterocyclic fragments. Such compounds are capable of forming a wide variety of supramolecular architectures, ranging from discrete host–guest complexes and mixed aggregates to extended coordination networks, metal–organic frameworks, tubular assemblies, and ordered liquid–crystalline phases. Their ability to self-assemble in solution is strongly influenced by external factors such as solvent, concentration, temperature, and the nature of the heterocyclic substituents, which makes these systems highly tunable. Modern physicochemical methods, including DLS, fluorescence spectroscopy, conductometry, SEM, TEM, and AFM, provide complementary information about the size, morphology, and dispersity of the resulting aggregates. In addition to fundamental interest in molecular recognition and self-organization, heterocycle-containing calixarenes demonstrate significant practical potential in the design of functional materials, ion-transport systems, liquid crystals, catalytic systems, and self-healing polymeric materials. Therefore, the integration of heterocyclic fragments into calixarene frameworks offers broad opportunities for the development of advanced supramolecular systems with controllable structure and properties.

### 5.4. Limitations of Heterocycle-Containing Calixarenes Application

Despite the substantial progress in the development of **HetCA**s for sensing, catalysis, and biomedical applications, several challenges continue to limit their transition from proof-of-concept studies to practical applications.

A major issue is the solubility and aggregation of **HetCA**s. Although hydrophilic substituents such as carbohydrate moieties, PEG chains, and quaternary ammonium groups can improve aqueous solubility, many derivatives remain poorly soluble in water or buffer media, requiring organic co-solvents that may affect binding equilibria and promote aggregation. More systematic characterization of aggregation, using methods such as DLS and diffusion NMR, is therefore needed.

Synthetic scalability and reproducibility also remain important limitations, as the preparation of many **HetCA**s involves multistep procedures, low overall yields, sometimes expensive reagents, and complex purification, while variations in reaction conditions and product purity can affect their reported properties. The development of more efficient, robust, and scalable synthetic approaches, together with standardized characterization and testing protocols, would facilitate meaningful comparison between different systems.

Another important consideration is stability and pH dependence. **HetCA** derivatives containing hydrolytically labile linkages may exhibit limited stability in aqueous media, while protonation or deprotonation of heterocyclic and phenolic groups can substantially alter their binding properties. Selectivity in complex media also requires greater attention, since the performance observed in idealized laboratory conditions may not be retained in biological or environmental matrices containing competing species, proteins, and other potential interferents.

Finally, for biomedical applications, toxicity, biocompatibility, and long-term fate should be evaluated systematically, particularly for **HetCA**s containing positively charged or biologically active heterocyclic fragments. Addressing these challenges will require an integrated approach in which solubility, stability, selectivity, toxicity, synthetic accessibility, and reproducibility are considered at the molecular-design stage rather than evaluated only after a promising system has been identified. Such a shift from proof-of-concept optimization toward application-oriented molecular design will be essential for translating the structural versatility of **HetCA**s into robust sensing, catalytic, and biomedical technologies.

## 6. Conclusions

Over the past three decades, modification with heterocycles has become one of the most effective approaches to expanding the structural diversity and functional capabilities of (thia)calixarenes. The incorporation of nitrogen, sulfur, oxygen, and mixed heterocyclic moieties has enabled precise control of molecular recognition, coordination behavior, self-assembly, and physicochemical properties, leading to the emergence of a wide range of functional systems for application in supramolecular and coordination chemistry, catalysis, sensorics, and biomedicine.

As demonstrated in this review, significant progress has been made in the synthesis of (thia)calixarenes containing heterocycles. The development of versatile synthetic methods, including nucleophilic substitution reactions, cross-linking strategies, click chemistry, and Schiff base formation, has provided access to macrocyclic structures of diverse types. At the same time, the accumulated literature data clearly demonstrate that the nature, position, and connectivity of the heterocyclic moieties play a crucial role in determining the recognition and coordination properties of the resulting systems. The collected literature data show that the functional behavior of these systems is determined not only by the identity of the heterocycle itself, but also by its spatial arrangement on the macrocyclic platform, the architecture of the linker, and the conformation of the macrocycle, which underscores the importance of rational molecular design.

Despite significant advances, a number of unsolved problems remain. Structure–property relationships are often difficult to generalize due to the diversity of macrocyclic platforms and heterocyclic substituents. Furthermore, many studies continue to focus on the preparation and characterization of new compounds, while systematic investigations of their functional behavior remain relatively limited.

Future research directions should prioritize: (1) the development of water-soluble and biocompatible heterocyclic calixarenes for in vivo applications; (2) the design of copper-free clickable derivatives to eliminate toxicity and simplify purification; (3) the use of high-throughput screening and machine learning to accelerate structure–property optimization; and (4) the integration of heterocyclic calixarenes into solid-supported materials (e.g., MOFs, polymers) for recyclable catalysis and sensing. The integration of heterocyclic (thia)calixarenes into advanced materials, molecular devices, and hybrid functional systems is likely to further expand their scientific and technological significance. Given the continued growth of this field, heterocyclic modification will likely remain a key strategy for the development of next-generation calixarene-based architectures.

Thus, the development of heterocyclic (thia)calixarene chemistry reflects a transition from the empirical functionalization of macrocycles to rational molecular design based on an understanding of the relationship between structure, spatial organization, and function. Further development in this field will likely involve the creation of multifunctional systems with predictable properties for applications in coordination chemistry, catalysis, sensor technology, materials science, and biomedicine.

## Figures and Tables

**Table 1:** Structure–property design rules for heterocycle-containing calixarenes.

Structural Parameter	Effect on Metal Binding	Effect on Anion Recognition	Effect on Sensing	Effect on Catalysis	Effect on Bioactivity
Lower rim vs. Upper rim	Lower rim → preorganized cavity for cations; Upper rim → exposed groups for external targets	Lower rim → better for small anions; Upper rim → better for bulky anions	Lower rim → higher selectivity; Upper rim → faster response	Lower rim → better substrate preorganization	Upper rim → better accessibility for biomacromolecules
Linker length	Short rigid linkers → high selectivity; Long flexible linkers → lower selectivity but faster kinetics	Short linkers promote H-bonding; Long linkers enable conformational adaptation	Short linkers enhance PET efficiency; Long linkers reduce quenching	Short linkers → better enantioselectivity	Long linkers improve cellular uptake
Heterocycle type	N/O/S donors → transition metals; Crown ethers → alkali metals	H-bond donors (NH, OH) → anions; Imidazolium → electrostatic binding	Conjugated heterocycles (BODIPY, naphthalimide) → fluorescence; TTF → electrochemical response	NHC, pyridine, triazole → metal coordination for catalysis	Pharmacophores (β-lactams, quinolones, triazoles) → direct bioactivity
Substitution number	1,3-Disubstitution → 1:1 complexes; Tetrasubstitution → polynuclear complexes or cooperative binding	1,3-Disubstitution → selective; Tetrasubstitution → high capacity	Mono- → simple ON-OFF; Tetra- → ratiometric or multi-analyte	Di- → moderate activity; Tetra- → higher activity but possible steric hindrance	Higher substitution → enhanced multivalency and bioactivity
Conformation (*cone*, *partial cone*, *1,3-alternate*)	*Cone* → best for cations; *1,3-alternate* → best for anions and neutral guests	*Cone* → anion binding via convergent H-bonds; *1,3-alternate* → divergent binding sites	*Cone* → strong fluorescence; *1,3-alternate* → reduced self-quenching	*Cone* → enantioselective; *1,3-alternate* → better for bulky substrates	*Cone* → better for membrane penetration; *1,3-alternate* → higher water solubility

## Data Availability

The original contributions presented in this study are included in the article/Supplementary Material. Further inquiries can be directed to the corresponding authors.

## Supplementary Materials

The following supporting information can be downloaded at: https://www.mdpi.com/article/10.3390/molecules31173079/s1. Tables S1–S13: Experimental details for the synthesis of **HetCA**; Tables S14–S16: Comparison of the properties of host-guest (H:G) complexes formed by **HetCA**s and various subsrates; Table S17: Some of electroanalytical data of various **HetCA**s; Tables S18–S32: Results of Reactions 1–9; Table S33: Details of biological activity of **HetCA**s; Table S34: Examples of data of **HetCA**s association measured via DLS.

## Abbreviations

The following abbreviations are used in this manuscript:

AAC	Azide–alkyne cycloaddition
ADP	Adenosine diphosphate
AFM	Atomic force microscopy
ATP	Adenosine triphosphate
BODIPY	Boron-dipyrromethene, 4,4-difluoro-4-bora-3a,4a-diaza-s-indacene
CA	(Thia)calixarene
Cbz	Benzyloxycarbonyl
CDI	1,1′-Carbonyldiimidazole
CuAAC	Copper-catalyzed azide–alkyne cycloaddition
DCC	*N*,*N*′-Dicyclohexylcarbodiimide
DCM	Dichloromethane
DDQ	2,3-Dichloro-5,6-dicyano-1,4-benzoquinone
DIPEA	*N*,*N*-Diisopropylethylamine
DLS	Dynamic light scattering
DMAP	*p*-(*N*,*N*-dimethyl)aminopyridine
DMF	*N*,*N*-dimethylformamide
EDA	Ethylene diamine
EDCI	1-Ethyl-3-(3-dimethylaminopropyl)carbodiimide
EtOAc	Ethyl acetate
HATU	Azabenzotriazole tetramethyl uronium hexafluorophosphate
HBT	1*H*-1,2,3-Benzotriazol-1-ol
HEPES	4-(2-hydroxyethyl)-1-piperazineethanesulfonic acid
HetCA	(Thia)calixarene, modified with heterocyclic moieties
ITC	Isothermal titration calorimetry
MIC	Minimum inhibitory concentration
MOF	Metal–organic framework
MW	Microwaves
NaAsc	Sodium ascorbate
NHS	*N*-Hydroxysuccinimide
PAMAM	Polyamidoamine
PDI	Polydispersity index
PBS	Phosphate-buffered saline
PyBOP	Benzotriazol-1-yl-oxytripyrrolidinephosphonium hexafluorophosphate
SEM	Scanning electronic microscopy
TBTA	Tris-(benzyltriazolylmethyl)amine
TBTU	*O*-(Benzotriazol-1-yl)-*N*,*N*,*N’*,*N’*-tetramethyluronium tetrafluoroborate
TCM	Trichloromethane
TEA	Triethylamine
TEM	Transmission electronic microscopy
Tf	Triflate group
THF	Tetrahydrofuran
TPP	Triphenylphosphine
Ts	Tosyl group
TTF	Tetrathiafulvalene
XRD	X-Ray diffraction
