# Supplementary material for: The Synergy of Macrocycles and Heterocycles: A Review of Coordination Behavior, Sensing, and Functional Materials Based on Calixarenes
Source: Molecules. 2026 Sep 1;31(17):3079. doi: 10.3390/molecules31173079 (PMC13567074; doi:10.3390/molecules31173079)
Supplement: Supplementary file 1 [file molecules-31-03079-s001.zip › molecules-4499155-supplementary.pdf]

Supplementary Materials

For

The Synergy of Macrocycles and Heterocycles: A Review of Coordination Behavior, Sensing, and Functional Materials Based on Calixarenes

Valeriy Kalinin, Pavel Padnya*, and Ivan Stoikov*

A.M. Butlerov Chemical Institute, Kazan Federal University, 18 Kremlevskaya Street, 420008 Kazan, Russian Federation

*Correspondence: Pavel.Padnja@kpfu.ru (P.P.); Ivan.Stoikov@kpfu.ru (I.S.); Tel.: +7-843-233-7463 (I.S.).

Table S1. Experimental details for the synthesis of **HetCA** using type **I** electrophilic reagents.

Heterocycle	Base	Solvent	Conditions	Time (h)	Yield (%)	Ref.
			Monosubstituted **HetCA**s			
Bipyridine	NaH	DMF	40 °C	3	35 – 48	[S1, S2]
Bipyridine	$BaO/Ba(OH)_2$	DMF	N/D*	N/D	N/D	[S2]
Bithiazole	$NaHCO_3$	MeCN	Reflux	28	71	[S3, S4]
Pyridine (*m*-bismethylpyridine)	K_2CO_3	MeCN	Reflux	96	30	[S5]
Quinoline	NaH	DMF	rt	5	50	[S6]
			Disubstituted **HetCA**s			
Bipyridine	Na_2CO_3	Acetone	Reflux	48	18	[S7]
Benzimidazole	K_2CO_3	MeCN	Reflux, KI	22	75	[S8]
Bipyridine	K_2CO_3	Acetone	Reflux	24	56	[S9]
Bithiazole	NaH	DMF	rt	2	29 (proximal)	[S4]
Bithiazole	K_2CO_3	MeCN	Reflux	25	85 (distal)	[S3, S4]
Bithiazole	Na_2CO_3	Acetone	Reflux	36	26	[S7]
BODIPY	DIPEA	TCM / DMF	Reflux	72	60	[S10]
Phenanthridine	K_2CO_3	MeCN	Reflux, NaI	48	29	[S11]
Pyridine	NaH	DMF	60 °C	18	53	[S12]
Pyridine (Pycolyl)	K_2CO_3 or Cs_2CO_3	DMF/THF	Reflux	17 – 20	55 – 76	[S13, S14]
Pyridine (Pycolyl)	NaH	DMF	65 °C	24	45	[S15]
Quinoline	NaH	DMF	65 °C	24	20	[S15]
Pyridine (Pycolyl) (distal)	K_2CO_3 or Cs_2CO_3	DMF or MeCN	70-80 °C	20	60 – 72	[S16–S26]
Pyridine (Pycolyl) (proximal)	NaH	DMF	60 °C	Overnight	70	[S27]
Pyridine (Uracyl)	K_2CO_3	MeCN	Reflux, KI	3 – 96	74 – 90	[S28]
Thiazolyl	K_2CO_3	MeCN	Reflux	3.5	82	[S29]
			Trisubstituted **HetCA**s			
Bithiazole	$Ba(OH)_2/BaO$	DMF	rt	25	62	[S4]
			Tetrasubstituted **HetCA**s			
Bipyridine	Cs_2CO_3	Acetone	Reflux	96	21	[S7]
Bithiazole	NaH	DMF	rt	2	43	[S4, S30]
Bithiazole	Cs_2CO_3	Acetone	Reflux	96	30	[S7]
Pyridine (Pycolyl)	NaH	DMF	60 °C	24	80	[S16, S31]
Pyridine (Pycolyl)	Cs_2CO_3	DMF	70 °C	36	18 – 54	[S14, S16, S32–S35]
Pyridine (Pycolyl)	Cs_2CO_3	Acetone	Reflux or rt	16 – 40	46 – 50	[S36, S37]

* N/D means “no data”.

Table S2. Experimental details for the synthesis of **HetCA** using type **II** electrophilic reagents.

Heterocycle	Base	Solvent	Conditions	Time (h)	Yield (%)	Ref.
			Disubstituted **HetCA**s			
1,3,4-oxadiazole	K_2CO_3	Acetone	Reflux, KI	100	52 – 65	[S38]
Benzo-15-crown-5	K_2CO_3	MeCN	Reflux, KI	N/D*	70 – 75	[S39]
Coumarin	K_2CO_3 or Cs_2CO_3	MeCN	Reflux, NaI	24	54 – 59	[S40]
Pyrrolidine (*L*-proline)	K_2CO_3	Acetone	Reflux, KI	N/D	83	[S41]
Quinoline	K_2CO_3	Acetone	Reflux, NaI	24	55	[S42]
Thiadiazole	K_2CO_3	MeCN	Reflux	8	64 – 69	[S43]
			Tetrasubstituted **HetCA**s			
Pyridine	NaH	THF/DMF	Reflux, KI	72	77	[S44]
Tetrahydrofuran	NaH	THF/DMF	Reflux, KI	72	61	[S44]

* N/D means “no data”.

Table S3. Experimental details for the synthesis of **HetCA** using type **III** electrophilic reagents.

Heterocycle	Base	Solvent	Conditions	Time (h)	Yield (%)	Ref.
			Disubstituted **HetCA**s			
Chromenopyridine	K_2CO_3	DMF	MW	1	45 – 56	[S45]
Benzofurazan	-	EtOAc	60 °C	4	60	[S46]
1,3,5-triazine	K_2CO_3	Acetone	Reflux	38	80	[S47]
			Tetrasubstituted **HetCA**s			
Triazaphosphinane	BuLi	THF	rt	2	68	[S47]
Triazaphosphinane	BuLi	THF	rt	15	55	[S47]
1,3-dioxaphospholane	BuLi	THF	rt	16 – 96	67 – 85	[S48, S49]
Tetrazole/Furane (Ribose)	NaH	DMF	60 °C	18	60	[S50]

Table S4. Experimental details for the synthesis of **HetCA** using type **IV** electrophilic reagents.

Heterocycle	Base	Solvent	Conditions	Time (h)	Yield (%)	Ref.
			Monosubstituted **HetCA**s			
Phthalimide	Cs_2CO_3	Acetone	Reflux	24	65	[S51]
Quinolone	K_2CO_3	MeCN	Reflux, KI	20 – 22	46 – 53	[S52]
			Disubstituted **HetCA**s			
Bithiophene	K_2CO_3 or NaH	MeCN or DMF	Reflux	20 or 120	59 – 60	[S53, S54]
Imidazolinium	K_2CO_3	Acetone	Reflux	144	80	[S55]
Morpholine	NaH	THF / DMF	83 °C	3	73	[S56]
Oxiran/Furan	K_2CO_3	MeCN	Reflux	24	80	[S57]
Phtalimide	Na_2CO_3	MeCN	Reflux	50	79	[S58]
Phthalimide	K_2CO_3	DMF	70 °C	80	60	[S59]
Phthalimide	NaH	DMF	rt	144	75	[S60]
Piperidine	K_2CO_3	MeCN	Reflux	76	61	[S61]
Piperidine	NaH	THF / DMF	83 °C	3	73	[S56]
Pyridine	K_2CO_3	MeCN	Reflux, KI	144	30 – 54	[S62]
Pyrrolidine	NaH	THF / DMF	83 °C	3	80	[S56]
Quinolone	K_2CO_3	MeCN	Reflux, KI	20 – 22	60 – 67	[S52]
Quinolone	K_2CO_3	MeCN	Reflux	48	20	[S63]
Tetrathiafulvalene	CsF	MeCN	Reflux	35	36	[S64]
			Tetrasubstituted **HetCA**s			
Phtalimide	Na_2CO_3 or Cs_2CO_3	Acetone	Reflux	24 or 100	53 or 68	[S58]

Table S5. Experimental details for the synthesis of **HetCA** using type **V** electrophilic reagents.

Heterocycle	Base	Solvent	Conditions	Time (h)	Yield (%)	Ref.
Disubstituted **HetCA**s						
Pyrazine	K_2CO_3	Acetone	Reflux	24	84	[S65]
Pyridine	TEA	TCM	rt-Reflux	24 – 96	52 – 83	[S66, S67]
Pyridine	TEA	DCM	rt	4.5	55	[S68]
Pyridine	TEA	DCM	rt	Overnight	50	[S69]
Trisubstituted **HetCA**s						
Pyridine	TEA	TCM	rt-Reflux	24	72	[S70]
Tetrasubstituted **HetCA**s						
Quinoline	K_2CO_3	Acetone	Reflux	24	65	[S71]

Table S6. Experimental details for the synthesis of 1,3-disubstituted **HetCA**s from macrocyclic electrophilic agents.

Heterocycle	Base	Solvent	Conditions	Time (h)	Yield (%)	Ref.
Benzothiazole	$NaHCO_3$	THF/water	Reflux	48	75 – 95	[S72]
Benzothiazole	TEA	THF	rt	48	50	[S73]
Furan	-	THF	rt	3	50	[S74]
Tetrahydrofuran	-	THF	rt	3	65	[S74]
Indole	TEA	THF	rt	Overnight	79	[S75]
Morpholine	-	THF	rt	3	73 – 75	[S74, S76]
Oxadiazole, Pyridine	K_2CO_3	MeCN	Reflux	24	64	[S77]
Triazole, Pyridine	K_2CO_3	MeCN	50 °C	72	72	[S77]
Piperidine, pirymidine	-	DCM, THF	rt	4 – 5	79	[S78]
Piperidine, THF	-	DCM, THF	rt	4 – 5	85	[S78]
Pyran (Carbohydrate moiety)	TEA	THF	rt	48	52	[S79]
Pyran (Carbohydrate moiety)	DMAP	DCM	rt	3	20	[S80]
Pyrazole	TEA	THF	rt	48	71	[S81]
Pyrazole, Benzothiazoline	TEA	TCM	rt	24	52	[S82]
Pyridine (bis(2-pycolylamine))	TEA	THF	rt	48	35	[S83]
Pyrrolidine (*L*-proline)	Pyridine	DCM	rt	3	75	[S84]
Quinoline	K_2CO_3	MeCN	N/D*	N/D	25	[S85]
Spirobenzopyran	-	DCM	rt	36	15	[S86]
Thiadiazole	K_2CO_3	DMF	Reflux	6	69 – 76	[S87]
Thiazole	K_2CO_3	MeCN	55 °C	24	N/D	[S88]

* N/D means “no data”.

Table S7. Experimental details for the synthesis of tetrasubstituted **HetCA**s from macrocyclic electrophilic agents.

Heterocycle	Base	Solvent	Conditions	Time (h)	Yield (%)	Ref.
Indole	TEA	THF	rt	Overnight	78	[S75]
Benzotriazole	TEA	THF	rt	Overnight	70 – 77	[S89]
Pyridine	TEA	THF	rt	Overnight	57 – 93	[S89]
Imidazole	-	MeOH	Reflux	72	21	[S90]
Imidazole	-	DCM	rt	12	84	[S90]
Imidazole	K_2CO_3	DMF	90 °C	12	69	[S91]
Purine	TEA	DCM	rt	12	49	[S92]
Pyran (Carbohydrate moiety)	TEA	MeCN	rt	16	65	[S93, S94]
Pyridine	-	DCM	rt	Overnight	54 – 82	[S95]
Tetrazole	K_2CO_3	DMF	90 °C	10	67	[S96]

Table S8. Experimental details for the synthesis of **HetCA**s using cross-linking reactions based on macrocyclic acids.

Heterocycle	Base	Solvent	Conditions	Time (h)	Yield (%)	Ref.
			Monosubstituted **HetCA**s			
Benzimidazole	DCC/HBT	TCM	rt	20	80 – 85	[S97]
Bipyridine	EDC×HCl	Pyridine	rt	4	67	[S98]
			Disubstituted **HetCA**s			
Benzimidazole	DCC/HBT	TCM	rt	20	80 – 85	[S97]
Aza-crown	DCC/HBT	TCM/THF	rt	6	95 – 98	[S99]
Tetrathiafulvalene	Het-moiety was introduced via *N*-hydroxysuccinic derivative	THF	rt	24	76	[S100]
Porphyrin	DCC	DCM	rt	48	20	[S101]
Quinoline	DCC/DMAP	DCM	40 °C	48	59	[S102]
Rhodamine	EDC×HCl	DCM	rt	12	63	[S103]
Thiophene	EDC/DMAP	THF or DCM	rt	96	31 – 32	[S104, S105]
Thiophene, Benzothiadiazole	EDC/DMAP	THF	rt	120	55	[S106]
Thiospirolactam	DCC/DMAP	DCM	rt	24	25	[S107]
			Tetrasubstituted **HetCA**s			
Benzimidazole	DCC/HBT	TCM	rt	20	75	[S97]
Tetrathiafulvalene	DCC/DMAP/HBT	DCM	rt	24	60	[S108]
Imidazole	Het-moiety was introduced via *N*-hydroxysuccinic derivative	DCM	rt	48	40	[S109]
Pyrrolidine	Het-moiety was introduced via *N*-hydroxysuccinic derivative	EtOAc	30 °C	3	80	[S110]

Table S9. Experimental details for the synthesis of **HetCA**s using cross-linking reactions based on low-molecular-weight heterocyclic acids.

Heterocycle	Base	Solvent	Conditions	Time (h)	Yield (%)	Ref.
			Monosubstituted **HetCA**s			
Biotin	HATU/DIPEA	DCM	rt	6	67	[S111]
Pyrrolidine (*L*-proline)	DCC/DMAP	DCM	rt	10	48 or 71	[S112, S113]
Thymine	DCC/HBT	DMF	rt	24	85	[S114]
Thymine	TBTU/HBT/4-methylmorpholine	DCM	rt	0.5	69	[S115]
			Disubstituted **HetCA**s			
Pyrimidine (Thymine)	DCC/HBT	DMF	rt	24	86	[S114]
Pyrimidine (Thymine)	TBTU/HBT/4-methylmorpholine	DCM	rt	0.5	69	[S115]
Benzoxazolinone	DCC/DMAP	DCM	rt	48	55	[S116]
Isoquinoline	DCC/DMAP	THF	rt	48	72	[S117]
Pyrimidine (Uracil or Thymine)	CDI	DMF	rt	96	60 or 67	[S118]
Purine (Adenine)	CDI	DMF	rt	96	65	[S118]
Pyrazine	PyBOP/DIPEA	DMF	rt	72	67 or 86	[S119]
Pyrrolidine (*L*-proline)	DCC	DCM	rt	60	73	[S84, S120]
Thiophene	EDC×HCl	DCM	rt	24	58	[S121]
Thymine	DCC/HBT	DMF	rt	24	29	[S122]
Xanthene	EDC×HCl/DMAP	DCM	rt	48	79	[S123]
			Tetrasubstituted **HetCA**s			
1,3,4-oxadiazole	DCC/DMAP	DCM	rt	24	76 or 78	[S124]
1,3,4-thiadiazole	DCC/DMAP	DCM	rt	24	71 or 73	[S124]

Table S10. Experimental details for the synthesis of **HetCA**s using azide-alkyne coupling of heterocyclic azides to calix[4]arene propargyl derivatives.

Heterocycle	Base	Solvent	Conditions	Time (h)	Yield (%)	Ref.
			Disubstituted **HetCA**s			
Benzimidazole	$CuSO_4$/NaAsc	*t*-BuOH, DCM, H_2O	rt	12	73	[S125]
Pyran (Carbohydrate moiety)	CuI/TEA	MeCN	rt	Overnight	88	[S126]
Pyran (Carbohydrate moiety)	CuI/DIPEA	DMF	110 °C	Overnight	46	[S127]
Pyran (Carbohydrate moiety) + crown ether	CuI/NaAsc	EtOH/DCM	rt	24	53	[S128]
Pyran (Carbohydrate moiety), 1,2,3-triazole	TBTA/$CuSO_4$/ NaAsc	THF/H_2O	rt	120 – 168	43 – 88	[S129]
TTF	$CuSO_4$/NaAsc	DMF	90 °C	10	20 – 47	[S130, S131]
			Tetrasubstituted **HetCA**s			
Pyran (Carbohydrate moiety)	CuI/TEA	DMF/MeCN	rt	Overnight	11 – 12	[S126]
Pyran (Carbohydrate moiety)	CuI/DIPEA	DMF	110 °C	Overnight	77	[S127]
Pyran (Carbohydrate moiety)	$CuSO_4$/NaAsc	Dioxane/H_2O	80 °C	0.75	75	[S132]
Pyran (Carbohydrate moiety)	$CuSO_4$/NaAsc	DMF	90 °C	60	37	[S133]

Table S11. Experimental details for the synthesis of **HetCA**s using azide-alkyne coupling of heterocycle-containing alkynes to macrocyclic azides.

Heterocycle	Base	Solvent	Conditions	Time (h)	Yield (%)	Ref.
			Disubstituted **HetCA**s			
Coumarin	$CuSO_4$/NaAsc	MeCN/H_2O	90 °C	6	58	[S134]
Pyridine	$CuSO_4$/NaAsc	DMF	90 °C	2	58	[S135]
Quinoline	$CuSO_4$/NaAsc	DMF	90 °C	2	60	[S136]
			Tetrasubstituted **HetCA**s			
Pyran (Carbohydrate moiety)	CuI/DIPEA	DCM	rt	24	99	[S137]
Pyran (Carbohydrate moiety)	$CuSO_4$/NaAsc	DMF	70 °C	0.2	80	[S138]
Indole	CuI/TEA	Toluene/DME	40 °C	24	70 – 89	[S139]

Table S12. Experimental details for the synthesis of **HetCA**s using imine bond formation.

Heterocycle	Catalyst	Solvent	Conditions	Time (h)	Yield (%)	Ref.
			Monosubstituted **HetCA**s			
Pyridinium	-	EtOH	Reflux	2	39 – 85	[S140]
			Disubstituted **HetCA**s			
Quinoline	AcOH	EtOH	Reflux	4	68 – 76	[S141]
Pyridine	-	MeOH	rt	4 – 8	83 – 92	[S142, S143]
Thiophene	-	MeOH	rt	5	93	[S144]
			Tetrasubstituted **HetCA**s			
Pyridine	AcOH	EtOH	Reflux	20	44 – 69	[S145]
Thiophene	AcOH	EtOH	Reflux	20	71	[S145]
Pyrrole	AcOH	EtOH	Reflux	20	75	[S145]
Rhodamine	-	EtOH/TCM	Reflux	72	62	[S146]
Pyridine	-	DMF/EtOH	80 °C	24	89	[S147]
Indole (*N*-methylisatin)	-	EtOH	Reflux	24	90 – 95	[S148]

Table S13. Experimental details for the synthesis of **HetCAs** using quaternization.

Heterocycle	Solvent	Conditions	Time (h)	Yield (%)	Ref.
		Monosubstituted **HetCA**s			
Imidazole	MeCN	Reflux	30	88	[S149]
Morpholine	MeCN	Reflux	30	84	[S149]
Pyridine	MeCN	Reflux	60	76	[S149]
Imidazole	-	Reflux	24	99	[S150]
Imidazole	-	110 °C	24	95	[S151]
		Disubstituted **HetCA**s			
Imidazole	-	Reflux	24	98	[S150]
Cinchonidine	Toluene	Reflux	8	86 – 96	[S152]
Imidazole	MeCN	Reflux	10 – 50	54 – 87	[S149, S153–S159]
Morpholine	MeCN	Reflux	31 – 45	73 – 81	[S158]
Pyridine	MeCN	Reflux	12 – 37	79 – 88	[S158]
Imidazole	TCM	Reflux	48	95 – 99	[S160]
Imidazole	Toluene	Reflux	90	63	[S161]
Pyridine, Indolizine	MeCN	Reflux	20	69	[S162]
		Tetrasubstituted **HetCA**s			
Phthalimide	MeOH	Reflux	72	98	[S163]

Table S14. Comparison of the properties of host-guest (H:G) complexes formed by **HetCAs** and cations of various metals.

Heterocyclic fragment	Guest (G)	Binding constant (log K)	Stoichiometry (L:S) or (H:G)	Method	Solvent	Ref.
1,8-naphthalimide	Hg(II)	7.74	N/D*	Fluorescence	H_2O/EtOH	[S164]
Benzimidazole	Cu(II)	9.86	1:2	Fluorescence	MeOH	[S125]
	Hg(II)	4.32	1:1	Fluorescence	MeOH	[S165]
Benzofurazan	Cu(II)	5.49 – 5.67	2:1	Fluorescence	H_2O/MeCN	[S165]
	Ag(I)	4.12	1:1	Fluorescence	THF	[S46]
	Ag(I)	4.11	1:1	Fluorescence	THF/H_2O	[S167]
Benzothiazin	Co(II)	2.82	1:1	Fluorescence	DMSO	[S168]
Benzothiazole	Na(I)	2.91	1:1	Fluorescence	MeOH	[S169]
	Hg(II)	5.79	1:1	UV-Vis	DMF	[S170]
	Cu(II)	5.33	1:1	Fluorescence	EtOH/PBS buffer	[S171]
	Zn(II)	4.68	1:1	Fluorescence	EtOH/PBS buffer	[S171]
Benzotriazole	Ni(II)	10.95 – 11.06	1:2	Fluorescence	MeCN	[S172]
	Co(II)	11.25 – 11.26	1:2	Fluorescence	MeCN	[S172]
	Cu(II)	11.11 – 14.08	1:2	Fluorescence	MeCN	[S172]
Benzoxadiazole	Cr(III)	4.46	1:1	Fluorescence	TCM	[S173]
	Fe(III)	4.20	1:1	Fluorescence	TCM	[S173]
	Al(III)	4.72	1:1	Fluorescence	TCM	[S173]
Benzoxazolinone	Cu(II)	3.88	1:1	UV-Vis	MeCN	[S116]
Bipyridine	Eu(III)	5.4 – 5.9	1:1	UV-Vis	MeOH	[S1]
	Tb(III)	4.5 – 6.2	1:1	UV-Vis	MeOH	[S1]
	Gd(III)	5.4 – 6.1	1:1	UV-Vis	MeOH	[S1]
	Ag(I)	4.26	1:1	UV-Vis	DCM	[S174]
	Zn(II)	4.89	1:1	UV-Vis	DCM	[S174]
	Na(I)	3.40	1:1	NMR	$CDCl_3$/CD_3CN	[S9]
	K(I)	3.45	1:1	NMR	$CDCl_3$/CD_3CN	[S9]
	Ag(I)	3.57	1:1	NMR	$CDCl_3$/CD_3CN	[S9]
	Eu(III)	5.4 – 5.9	1:1	Fluorescence	MeOH	[S175]
	Tb(III)	4.5 – 6.2	1:1	Fluorescence	MeOH	[S175]
	Gd(III)	5.4 – 6.1	1:1	Fluorescence	MeOH	[S175]
BODIPY	Ca(II)	7.72	1:1	Fluorescence	MeCN	[S176]
	Hg(II)	5.48	1:1	Fluorescence	DMSO	[S177]
	Hg(II)	7.53	1:1	Fluorescence	MeOH/H_2O	[S178]
BODIPY/Rhodamine	Hg(II)	5.08	1:1	Fluorescence	MeCN	[S179]
	Ba(II)	5.23	1:1	Fluorescence	MeCN	[S179]
Carbazole	Cu(II)	4.72 – 4.93	1:1	Fluorescence	MeCN	[S180]
Coumarin	Fe(III)	3.74	1:1	Fluorescence	THF	[S181]
Coumarin	Cu(II)	4.26	1:1	Fluorescence	MeCN	[S182]
Fluorescein	Cu(II)	3.23	1:1	Fluorescence	MeCN/H_2O	[S183]
Fulvalene	Na(I)	N/D	1:1	NMR	$CDCl_3$/CD_3CN	[S108]
Furan	Cd(II)	8.71	2:1	Fluorescence	MeCN	[S184]
	Hg(II)	4.16	1:1	Fluorescence	MeCN	[S184]
	Pb(II)	3.93	1:1	Fluorescence	MeCN	[S184]
Imidazole	Cu(I)	11.87	1:1	Fluorescence	HEPES	[S185]
Indole	Na(I)	4.9	1:1	Fluorescence	MeCN	[S75]
	K(I)	3.4 – 7.3	1:1	Fluorescence	MeCN	[S75]
	Cs(I)	4.4 – 6.1	1:1	Fluorescence	MeCN	[S75]
Isoquinoline	Cu(II)	9.15	1:1	Fluorescence	MeCN	[S117]
Morpholine	Na(I)	7.50	1:1	Potentiometry	H_2O	[S186]

	K(I)	8.00	1:1	Potentiometry	H_2O	[S186]
	Rb(I)	7.30	1:1	Potentiometry	H_2O	[S186]
Naphthalimide	Co(II)	4.30	1:1	Fluorescence	DMF/H_2O	[S187]
	Cd(II)	9.25	2:1	Fluorescence	DMF/H_2O	[S187]
	Cu(II)	4.79	1:1	Fluorescence	MeCN	[S188]
Piperidine (Minoxidil)	Cu(II)	7.40	1:1	Fluorescence	EtOH/H_2O	[S189]
Phenanthridine	Li(I)	10.92	1:1	ITC	MeCN	[S11, S190]
	Na(I)	10.50	1:1	ITC	MeCN	[S11, S190]
	K(I)	6.35	1:1	ITC	MeCN	[S11, S190]
	Rb(I)	4.65	1:1	ITC	MeCN	[S11, S190]
	Cs(I)	2.85	1:1	ITC	MeCN	[S11, S190]
Phenanthridine, Triazole, Pyran (Carbohydrate moiety)	Li(I)	2.76	1:1	UV-Vis	MeOH	[S189]
	Na(I)	5.69	1:1	UV-Vis	MeOH	[S189]
	K(I)	3.50	1:1	UV-Vis	MeOH	[S189]
	Rb(I)	2.33	1:1	UV-Vis	MeOH	[S189]
Phenolphtalein	Zn(II)	11.65	1:2	Fluorescence	MeCN/H_2O	[S191]
Phenothiazine	Fe(III)	3.27	2:1	Fluorescence	THF/H_2O	[S192]
	Cr(III)	3.18	2:1	Fluorescence	THF/H_2O	[S192]
Phthalimide	Fe(III)	5.36	1:1	Fluorescence	THF	[S193]
	Ni(II)	3.23	1:1	Fluorescence	THF	[S193]
	Cu(II)	5.20	1:1	Fluorescence	THF	[S193]
	Zn(II)	4.36	1:1	Fluorescence	THF	[S193]
Pyrazole	Cu(II)	4.44 – 4.63	1:1	UV-Vis	MeOH/TCM	[S194]
	Hg(II)	4.04	1:1	Fluorescence	MeOH	[S81]
	Ag(I)	3.58	1:1	UV-Vis	MeOH/TCM	[S195]
	Hg(II)	3.48	1:1	UV-Vis	MeOH/TCM	[S195]
Pyridine	Ag(I)	13.13	1:2	UV-Vis	MeCN	[S172]
	Ni(II)	5.65	1:1	UV-Vis	MeCN	[S172]
	Co(II)	6.49	1:1	UV-Vis	MeCN	[S172]
	Zn(II)	10.87	1:2	UV-Vis	MeCN	[S172]
	Cu(II)	11.11	1:2	UV-Vis	MeCN	[S172]
	Li(I)	5.43	1:1	Electrochemistry	MeCN	[S196]
	Na(I)	5.20	1:1	Electrochemistry	MeCN	[S196]
	Zn(II)	4.26	1:1	Fluorescence	MeOH	[S226]
	Ni(II)	5.36	1:1	Fluorescence	MeOH	[S226]
	Cu(II)	4.26	1:1	Fluorescence	MeOH	[S197]
	Ag(I)	3.38 – 3.42	1:2	UV-Vis	DCM	[S198]
Pyridinium	Tl(I)	5.70	1:2	UV-Vis	DMSO	[S199]
Quinoline	Co(II)	4.36	1:1	Fluorescence	H_2O/THF	[S71]
	Zn(II)	4.57	1:1	Fluorescence	MeOH	[S42]
	Sr(II)	8.95	1:1	Fluorescence	H_2O/MeCN	[S102]
	Co(II)	10.84	2:1	Fluorescence	H_2O/MeCN	[S200]
	Cu(II)	9.18	1:2	Fluorescence	H_2O/MeCN	[S200]
	Hg(II)	3.93	1:1	Fluorescence	MeCN/H_2O	[S136]
	Fe(II)	3.64	1:1	Fluorescence	MeCN/H_2O	[S136]
	Cu(II)	3.08	1:1	Fluorescence	MeCN/H_2O	[S136]
	Cu(II)	4.29	1:1	Fluorescence	H_2O/THF	[S201]
Quinone / Triazole	Fe(III)	5.34	1:2	Fluorescence	MeCN	[S202]
Rhodamine	Hg(II)	3.93	1:2	Fluorescence	MeCN	[S103]
	Fe(II)	4.36	1:2	Fluorescence	MeCN	[S103]
	Zn(II)	4.32	1:2	Fluorescence	MeCN	[S103]
	Pb(II)	4.23	1:2	Fluorescence	MeCN	[S103]
	Hg(II)	4.08	1:1	Fluorescence	H_2O	[S202]

		5.52– 5.74	1:1	Fluorescence	DMF/HEPES	[S146]
Spirobenzopyran	Eu(III)	5.14	1:1	Fluorescence	MeCN	[S86]
Terpyridine	Zn(II)	6.52	1:1	Fluorescence	TCM/MeOH	[S62]
Terpyridine	Cu(II)	12.80	1:2	UV-Vis	DCM/EtOH	[S204]
	Ni(II)	13.40	1:2	UV-Vis	DCM/EtOH	[S204]
Thiazole	Pt(II)	5.00	1:1	Fluorescence	H_2O/MeOH	[S88]
Thiophene	Zn(II)	4.96	1:1	Fluorescence	HEPES:EtOH (1:2)	[S144]
Thiophene	Ag(I)	7.75	2:1	Fluorescence	MeCN	[S184]
	Cd(II)	15.45	3:2	Fluorescence	MeCN	[S184]
	Hg(II)	10.99	3:1	Fluorescence	MeCN	[S184]
	Pb(II)	2.56	1:1	Fluorescence	MeCN	[S184]
Thiospirolactam	Hg(II)	4.56	1:2	UV-Vis	H_2O/EtOH	[S107]
Triazole	Mg(II)	5.04	1:1	Fluorescence	DMSO	[S205]
	Fe(III)	4.30	1:1	Fluorescence	MeCN	[S206]
	Co(II)	5.68	1:1	Fluorescence	EtOH	[S207]
	Zn(II)	4.58 – 5.18	1:1	Fluorescence	MeOH	[S208, S209]
	Ca(II)	4.08	1:1	Fluorescence	MeCN	[S210]
	Fe(II)	2.98	1:1	Fluorescence	MeCN/TCM	[S128]
	Fe(III)	3.21	1:1	Fluorescence	MeCN/TCM	[S128]
	Fe(III)	4.90	1:1	Fluorescence	MeCN	[S211]
	Fe(III)	5.04 – 5.11	1:1	Fluorescence	MeCN/TCM	[S212]
	Ag(I)	3.65	1:1	Fluorescence	MeCN/TCM	[S213]
	Ag(I)	3.84	1:1	Fluorescence	MeCN/TCM	[S214]
	Zn(II)	4.23	1:1	Fluorescence	MeCN	[S215]
	Cd(II)	4.72	1:1	Fluorescence	MeCN	[S215]
	Hg(II)	6.80	1:1	NMR	$CD_3OH/CDCl_3$ (1:1)	[S216]
	Hg(II)	6.76	1:1	Fluorescence	MeCN	[S217]
	Cu(II)	6.76	1:2	Fluorescence	MeCN	[S217]
	Pb(II)	5.30	1:1	Fluorescence	MeCN	[S217]
	Zn(II)	4.79	1:1	Fluorescence	MeCN	[S217]
	Fe(III)	5.04 – 5.25	1:1	Fluorescence	MeCN/TCM	[S218]
	Fe(III)	5.08	1:1	Fluorescence	MeCN/TCM	[S219]
	Hg(II)	5.11	1:1	Fluorescence	MeCN/TCM	[S219]
	Ag(I)	5.11	1:1	Fluorescence	MeCN	[S220]
Triazole/Coumarin	Cu(II)	5.20	1:1	Fluorescence	MeCN	[S134]
Triazole/Pyran (Carbohydrate moiety)	Li(I)	3.25	1:1	UV-Vis	MeOH	[S137, S221]
	Na(I)	7.38	1:1	UV-Vis	MeOH	[S137, S221]
	K(I)	4.74	1:1	UV-Vis	MeOH	[S137, S221]
	Rb(I)	2.40	1:1	UV-Vis	MeOH	[S137, S221]
Triazole, Pyrydine	Fe(III)	4.73	1:1	Fluorescence	MeCN	[S135]
Triazole/Indolinspiropyran	Cu(II)	5.84	1:1	Fluorescence	MeCN	[S222]
Triazole/Pyridine	Zn(II)	5.60	1:1	Fluorescence	MeOH	[S223]
	Cd(II)	6.60	1:1	Fluorescence	MeOH	[S223]
Triazole/Pyrazole	Ag(I)	11.97	1:2	Fluorescence	TCM/EtOH	[S195]
	Hg(II)	12.16	1:2	Fluorescence	TCM/EtOH	[S195]

* N/D means “no data”.

Table S15. Comparison of the properties of host-guest (H:G) complexes formed by **HetCA**s and various anions.

Heterocyclic fragment	Guest (G)	Binding constant (log K)	Stoichiometry (L:S) or (H:G)	Method	Solvent	Ref.
1,8-naphthalimide	$Cr_2O_7^{2-}$	7.60 – 7.63	N/D*	Fluorescence	H_2O/EtOH	[S164]
Benzofurazan	$H_2PO_4^-$	4.25 – 5.27	2:1	Fluorescence	H_2O/MeCN	[S166]
	F^-	4.30 – 5.23	2:1	Fluorescence	H_2O/MeCN	[S166]
	AcO^-	4.20	1:1	Fluorescence	THF	[S46]
Benzoxazolinone	F^-	4.90	1:1	UV-Vis	MeCN	[S116]
	CN^-	4.58	1:1	UV-Vis	MeCN	[S116]
	AcO^-	4.40	1:1	UV-Vis	MeCN	[S116]
Coumarin	F^-	4.04	1:1	Fluorescence	MeCN	[S40]
	AcO^-	2.58	1:1	Fluorescence	MeCN	[S40]
Imidazolium	Cl^-	1.35	1:1	NMR	DMSO	[S90]
	Br^-	1.40	1:1	NMR	DMSO	[S90]
	NO_3^-	1.06	1:1	NMR	DMSO	[S90]
Indole (*L*-Triptophane)	*D*-Mandelate	3.08	1:1	Fluorescence	DMSO	[S224]
Isoquinoline	F^-	9.08	1:1	Fluorescence	MeCN	[S117]
Naphthalimide	F^-	4.04	1:1	Fluorescence	MeCN	[S188]
Pyridine	F^-	3.57	1:1	UV-Vis	MeCN	[S172]
	CN^-	3.89	1:1	UV-Vis	MeCN	[S172]
	Benzoate	4.60	1:1	NMR	Acetone	[S225]
Quinoline	CN^-	8.93	1:1	Fluorescence	H_2O/MeCN	[S102]
Triazole	I^-	2.99	1:1	Fluorescence	MeCN	[S227]
Triazole, Ferrocene	F^-	8.73	1:2	Fluorescence	MeCN/DCM (1:1)	[S228]

* N/D means "no data".

Table S16. Comparison of the properties of host-guest (H:G) complexes formed by **HetCAs** and molecular substrates of various nature.

Heterocyclic fragment	Guest (G)	Binding constant (log K)	Stoichiometry (L:S) or (H:G)	Method	Solvent	Ref.
1,3,5-triazine	Orange I	6.26	1:1	UV-Vis	DMSO	[S226]
	Methylene Blue	6.67	1:1	UV-Vis	DMSO	[S226]
	Neutral Red	6.58	1:1	UV-Vis	DMSO	[S226]
	Brilliant Green	6.91	1:1	UV-Vis	DMSO	[S226]
1*H*-Phenanthro[9,10-d]imidazolyl	Metronidazole	5.75	1:1	Fluorescence	MeCN	[S230]
Benzimidazole	TNT	4.48	1:3	Fluorescence	THF	[S231]
Benzo-15-crown-5	Orange I	4.93	1:1	UV-Vis	DMSO	[S39]
	Methylene Blue	4.67	1:1	UV-Vis	DMSO	[S39]
	Neutral Red	4.91	1:1	UV-Vis	DMSO	[S39]
	Brilliant Green	4.53	1:1	UV-Vis	DMSO	[S39]
BODIPY	pBR322 plasmid DNA	3.15 – 4.21	1:2	Fluorescence	DMSO	[S10]
Carbazole	C_{60}	4.57 – 4.62	1:1	Fluorescence	Toluene	[S232]
	C_{70}	4.64 – 4.75	1:1	Fluorescence	Toluene	[S232]
	Cytochrome C	5.66 – 7.78	1:1	Fluorescence	DMF	[S233]
Naphthalimide	Dopamine	4.84	1:1	Fluorescence	PBS	[S187]
	Trinitrophenol	5.53	1:1	Fluorescence	THF	[S134, S234]
	Pyrenaldehyde	4.95	1:2	Fluorescence	MeCN	[S325]
Phthalimide	Glycine	2.65	1:1	UV-Vis	H_2O	[S236]
	Phenylalanyne	4.36	1:1	UV-Vis	H_2O	[S236]
	Tyrosine	5.40	1:1	UV-Vis	H_2O	[S236]
	Tryptophane	5.11	1:1	UV-Vis	H_2O	[S236]
Tetrathiafulvalene	TCNQ	2.04 – 3.48	1:1	UV-Vis	DCM	[S162]
Triazole	BSA	4.67 – 4.93	1:1	Fluorescence	Tris-buffer	[S238]
	ATP	4.60	1:2	Fluorescence	EtOH	[S239]
	UTP	4.34	1:2	Fluorescence	EtOH	[S239]
	CTP	4.28	1:2	Fluorescence	EtOH	[S239]
	TTP	4.23	1:2	Fluorescence	EtOH	[S239]
	GTP	3.95	1:2	Fluorescence	EtOH	[S239]
	p-Nitroaniline	4.32	1:1	Fluorescence	MeCN	[S240]

Table S17. Some of electroanalytical data of various **Het-CA**s.

Heterocyclic fragment	Cation type (M)	pH range	Nernstian slope (mV decade^{-1})	Interfering cations (M^{int})	Selectivity coefficients, $-\log K^{pot}_{M, M^{int}}$	Limits of detection (M)	Ref.
Pyridine		N/D*	49.2–52.5	Na(I), K(I), NH_4(I), Ca(II), Mg(II), Cu(II), Zn(II), Ni(II), Cd(II), Pb(II), Hg(II)	1.5–4.6	N/D	[S241]
Benzothiazole		2.5–6.5	58	Na(I), K(I), NH_4(I), Ca(II), Mg(II), Cu(II), Zn(II), Ni(II), Cd(II), Pb(II), Hg(II), Co(II), Fe(III)	>2.6	$10^{-5.8}$	[S242, S243]
Benzothiazole		2.5–7.0	46.6–58.6	Na(I), K(I), NH_4(I), Ca(II), Mg(II), Cu(II), Zn(II), Ni(II), Cd(II), Pb(II), Hg(II), Co(II), Fe(III)	>2.0	1.3–5.0 × 10^{-6}	[S244]
Pyrimidine	Ag(I)	3.0–7.0	61.4	Na(I), K(I), Li(I), NH_4(I), Ca(II), Sr(II), Ba(II), Mg(II), Cu(II), Zn(II), Ni(II), Cd(II), Pb(II), Hg(II), Co(II), Fe(II), Fe(III)	>3.0	10^{-8}	[S245]
Pyridine		1.0–6.5	54.3-59.0	Na(I), K(I), NH_4(I), Ca(II), Mg(II), Cu(II), Zn(II), Ni(II), Cd(II), Pb(II), Hg(II), Co(II)	4.5	3.98–7.10 × 10^{-6}	[S246]
Pyridine		2.5–7.0	57	Na(I), K(I), NH_4(I), Ca(II), Mg(II), Cu(II), Zn(II), Ni(II), Cd(II), Pb(II)	>3.5	$10^{-5.3}$	[S247]
Pyridine		1.3–5.0	63	Na(I), K(I), Cu(II), Cd(II), Pb(II), Fe(III),	2.0-8.0	$10^{-4.5}$	[S248]
Thiazole	Hg(II)	3.0–7.0	61.1	Na(I), K(I), Li(I), NH_4(I), Ag(I), Ca(II), Sr(II), Ba(II), Mg(II), Cu(II), Zn(II), Ni(II), Cd(II), Pb(II), Hg(II), Co(II), Fe(II), Fe(III)	>3.0	4.5-8.0 × 10^{-7}	[S249]
Pyridine		5.0	24.5	Cu(II), Ca(II), Mg(II), Zn(II), Co(II), Ni(II),	>1.5	$10^{-6.2}$	[S250]
Pyridine	Pb(II)	3.0–6.5	24.6	Cd(II), Cu(II), Zn(II), Ni(II), Co(II)	N/D	4.0 × 10^{-9}	[S251]
Piperidine		1.0–4.5	N/D	Cd(II), Cu(II), Zn(II)	N/D	9.7 × 10^{-8}	[S252]
Thiazole	Ni(II)	3.0–7.6	28.8	Na(I), K(I), Li(I), NH_4(I), Ag(I), Ca(II), Sr(II), Ba(II), Mg(II), Cu(II), Zn(II), Ni(II), Cd(II), Pb(II), Hg(II), Co(II), Fe(III), Ce(III), Al(III),	0.43-1.7	9.0 × 10^{-7}	[S253]
Morpholine	Na(I)	1.0–5.0	60.0	Li(I), Cs(I), $(CH_3)_4N^+$, guanidinium, H^+, Ca(II), Mg(II), Sr(II), Hg(II)	0.6-3.4	N/D	[[illegible]
Piperidine	Dopamine	2.0; 7.0	N/D	Ascorbic acid, uric acid	N/D	4.0 × 10^{-12}	[S254]

* N/D means "no data".

Table S18. Results of Reaction 1a in different solvents [S41, S84, S112, S120, S255, S256].

Reaction 1a (aldol condensation)

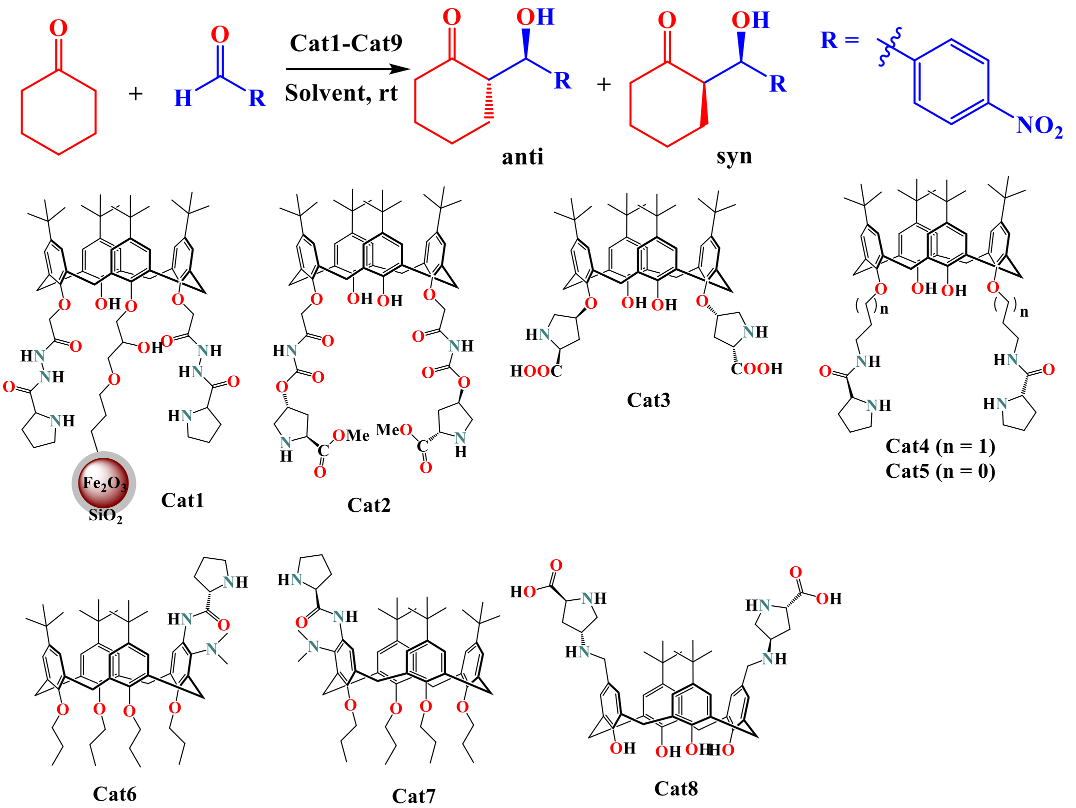

Entry	Catalyst	Concentration of catalyst (mol%)	Solvent	Time (h)	Yield (%)	Anti:syn	ee (%)	Ref.
1	**Cat1**	10	DMF	36	28	77:23	84	[S120]
2	**Cat1**	10	DMSO	36	33	76:24	71	[S120]
3	**Cat1**	10	TCM	36	61	75:25	62	[S120]
4	**Cat1**	10	THF	36	76	82:18	51	[S120]
5	**Cat1**	10	EtOH	36	28	57:43	37	[S120]
6	**Cat1**	10	H_2O	36	97	97:3	93	[S120]
7	**Cat2**	10	H_2O	24	92	86:14	73	[S41]
8	**Cat3**	2	H_2O	48	48	59:41	85	[S255]
9	**Cat4**	20	TCM	24	41	60:40	8	[S84]
10	**Cat5**	20	TCM	24	39	56:44	6	[S84]
11	**Cat4**	20	DMSO	24	50	64:36	13	[S84]
12	**Cat5**	20	DMSO	24	51	60:40	10	[S84]
13	**Cat4**	20	MeCN	24	39	56:44	12	[S84]
14	**Cat5**	20	MeCN	24	35	58:42	9	[S84]
15	**Cat4**	20	DMF	24	15	52:48	N/D	[S84]
16	**Cat5**	20	DMF	24	10	53:47	N/D	[S84]
17	**Cat4**	20	EtOH	24	<15	60:40	N/D	[S84]
18	**Cat4**	20	H_2O	24	90	65:35	46	[S84]
19	**Cat5**	20	H_2O	24	70	58:42	38	[S84]
20	**Cat4**	10	H_2O	24	93	67:33	48	[S84]
21	**Cat4**	5	H_2O	24	36	54:46	40	[S84]
22	**Cat6** + acetic acid	10	-	10	98	78:22	44	[S112]
23	**Cat7** + acetic acid	10	-	10	99	81:19	44	[S112]
24	**Cat8**	10	DMF	24	97	86:14	84	[S256]
25	**Cat8**	10	DMSO	24	73	78:22	64	[S256]
26	**Cat8**	10	TCM	24	88	87:13	81	[S256]
27	**Cat8**	10	THF	24	98	82:18	83	[S256]

Table S19. Results of Reaction 1b (water, r.t.) [S120].

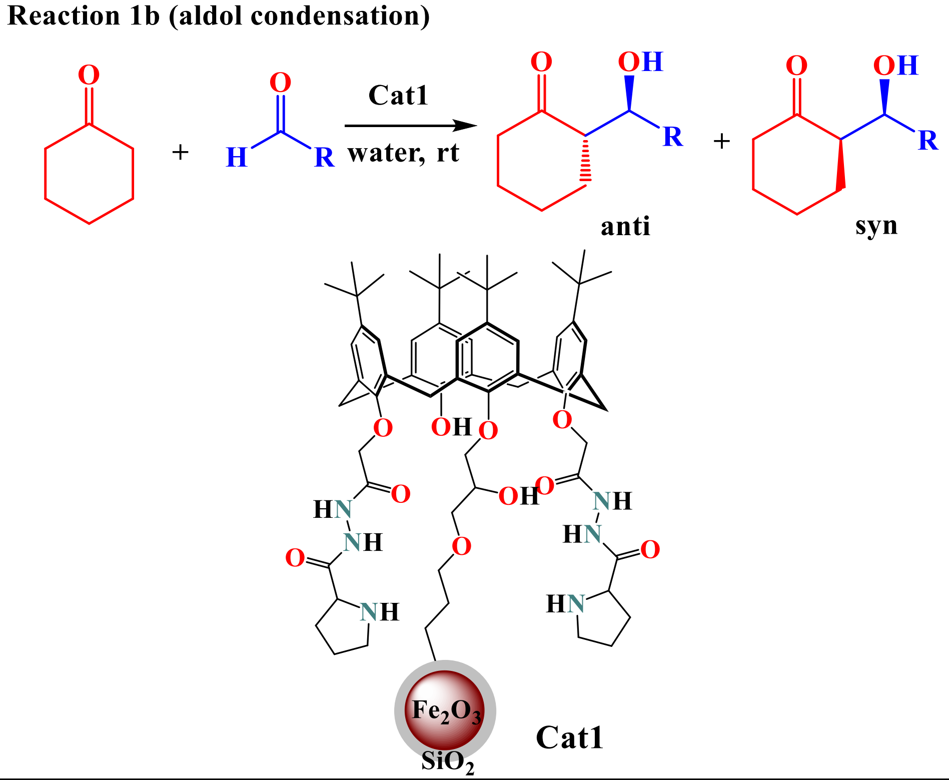

Entry	Catalyst	Concentration of catalyst (mol%)	R	Time (h)	Yield (%)	Anti:syn	ee (%)
1	**Cat1**	10	4-Cl-Ph	36	86	90:10	92
2	**Cat1**	10	4-Br-Ph	36	75	91:9	92
3	**Cat1**	10	4-F-Ph	36	91	94:6	90
4	**Cat1**	10	4-CN-Ph	36	90	84:16	84
5	**Cat1**	10	4-CF_3-Ph	36	91	87:13	84
6	**Cat1**	10	3-NO_2-Ph	36	74	84:16	90
7	**Cat1**	10	3-Cl-Ph	36	86	93:7	89
8	**Cat1**	10	2-NO_2-Ph	36	90	97:3	91
9	**Cat1**	10	2-Cl-Ph	36	94	79:21	85
10	**Cat1**	10	4-MeO-Ph	36	40	56:44	38

Table S20. Results of Reaction 1c (neat, –25 °C) [S257].

Reaction 1c (aldol condensation)

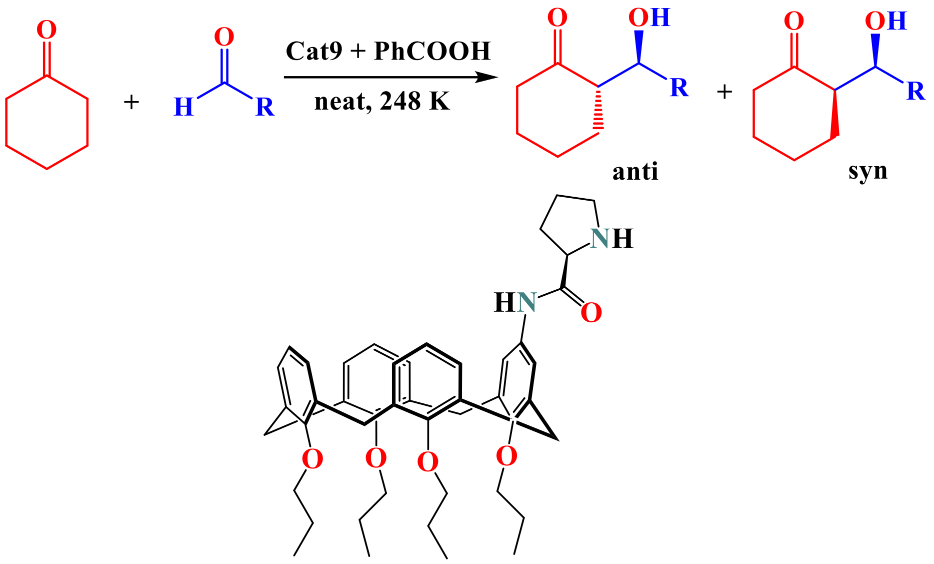

Cat9

Entry	Catalyst	Concentration of catalyst (mol%)	R	Time (h)	Yield (%)	Anti:syn	ee (%)
1	**Cat9** + PhCOOH	2	4-NO_2-Ph	48	>99	65:35	85
2	**Cat9** + PhCOOH	2	3-NO_2-Ph	48	96	53:47	46
3	**Cat9** + PhCOOH	2	2-NO_2-Ph	48	81	85:15	76
4	**Cat9** + PhCOOH	2	2-naphtyl	48	79	73:27	78
5	**Cat9** + PhCOOH	2	4-Br-Ph	48	92	55:45	68
6	**Cat9** + PhCOOH	2	4-Cl-Ph	48	77	46:54	97
7	**Cat9** + PhCOOH	2	4-NC-Ph	48	95	38:62	41
8	**Cat9** + PhCOOH	2	2-MeO-Ph	48	36	20:80	70

Table S21. Results of Reaction 2 [S76].

Reaction 2 (Mannich-type reaction)

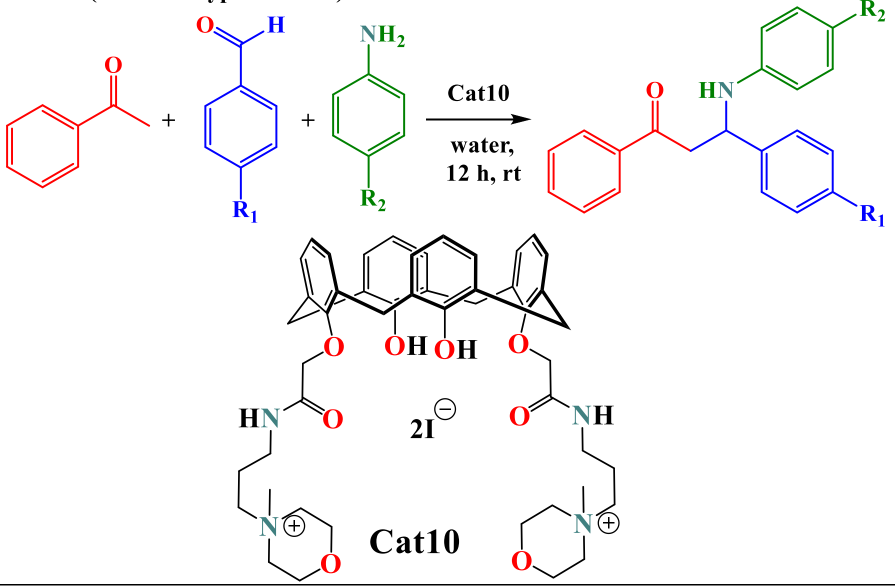

Entry	R_1	R_2	Time (h)	Yield (%)
1	H	H	6	62
2	H	MeO	6	50
3	NO_2	H	6	48
4	Cl	H	6	65

Table S22. Results of Reaction 3 [S152].

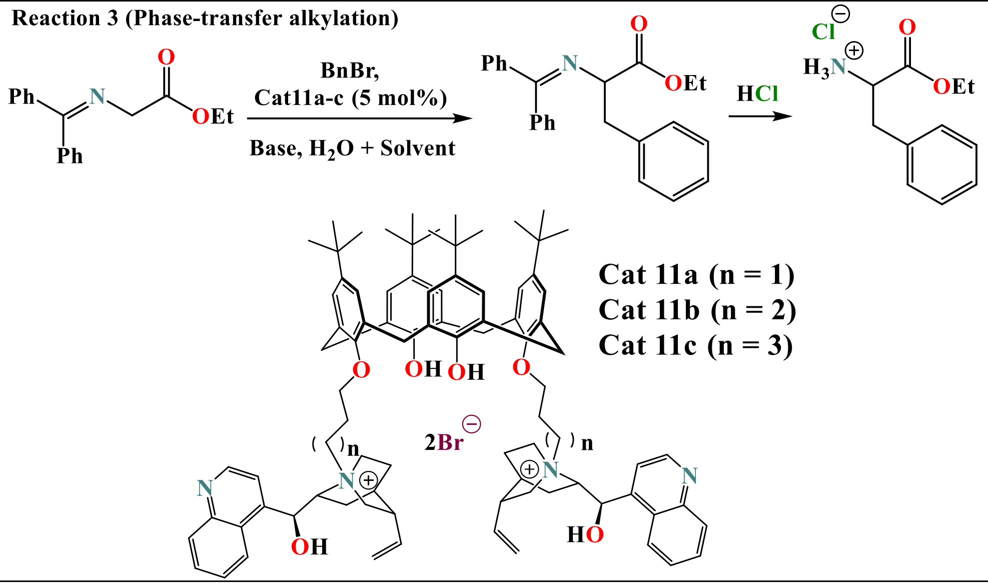

Entry	Catalyst	Solvent	Temperature (°C)	Base	Yield (%)	ee (S) (%)
1	**Cat11a**	MePh:TCM (7:3)	–20	KOH	87	25
2	**Cat11a**	MePh:TCM (7:3)	–20	NaOH	89	46
3	**Cat11a**	MePh:TCM (7:3)	0	NaOH	95	57
4	**Cat11a**	MePh:TCM (7:3)	rt	NaOH	92	52
5	**Cat11b**	MePh:TCM (7:3)	rt	KOH	93	15
6	**Cat11b**	MePh:TCM (7:3)	–20	KOH	90	10
7	**Cat11c**	MePh:TCM (7:3)	–20	KOH	91	12
8	**Cat11c**	MePh:TCM (7:3)	–20	NaOH	95	17
9	**Cat11c**	MePh:TCM (7:3)	0	NaOH	96	28
10	**Cat11c**	MePh:TCM (7:3)	0	KOH	97	22
11	**Cat11c**	MePh:TCM (7:3)	rt	KOH	98	18
12	**Cat11c**	MePh:TCM (7:3)	rt	NaOH	97	24
13	**Cat11c**	DCM	–20	KOH	89	9
14	**Cat11c**	DCM	0	KOH	93	15
15	**Cat11c**	DCM	rt	KOH	93	12
16	**Cat11c**	DCM	–20	NaOH	85	7
17	**Cat11c**	DCM	0	NaOH	88	14
18	**Cat11c**	DCM	rt	NaOH	91	13

Table S23. Results of Reaction 4a [S150].

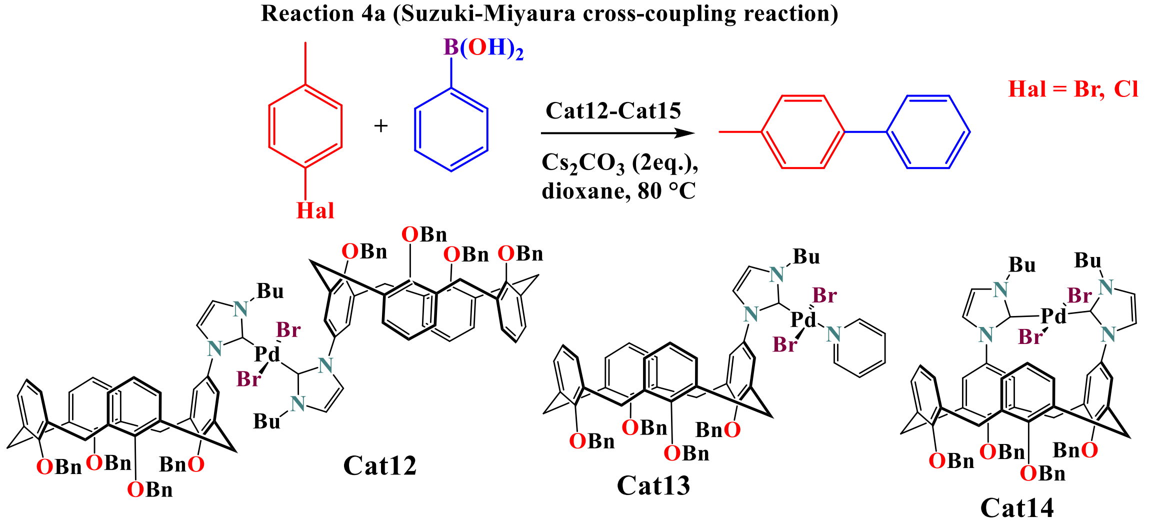

Entry	Catalyst	Concentration of catalyst (mol%)	Hal	Yield (%)
1	**Cat12**	0.1	Br	100
2	**Cat12**	0.05	Br	92
3	**Cat12**	0.01	Br	1
4	**Cat12**	1	Cl	67
5	**Cat13**	0.1	Br	100
6	**Cat13**	0.05	Br	84
7	**Cat13**	0.01	Br	2
8	**Cat13**	1	Cl	45
9	**Cat14**	0.1	Br	100*
10	**Cat14**	0.1	Br	4
11	**Cat15**	0.05	Br	61
12	**Cat15**	1	Cl	64

* After 24 h of synthesis

Table S24. Results of Reaction 4b [S258].

Reaction 4b (Suzuki-Miyaura cross-coupling reaction)

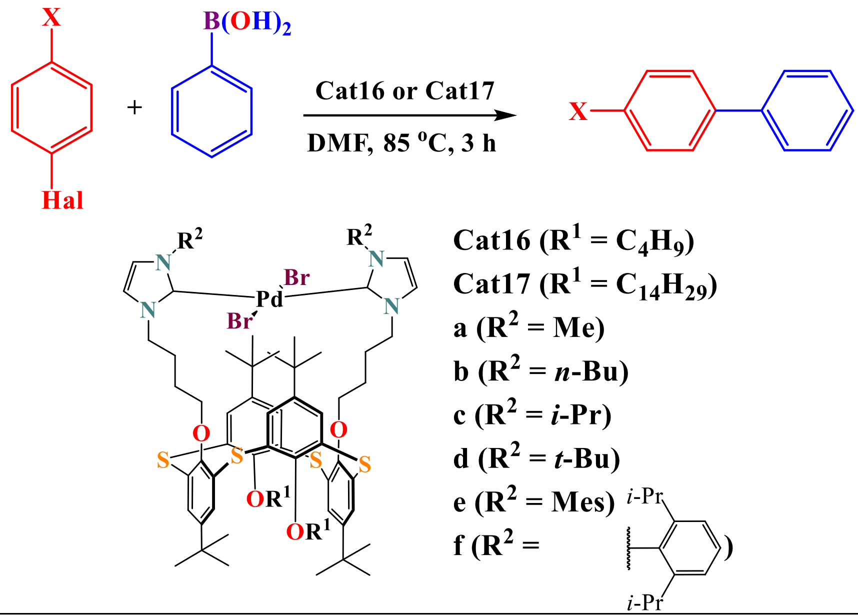

Entry	Catalyst	Hal	X	Concentration of catalyst (mol%)	Yield (%)
1	**Cat16a**	Br	NO_2	1	100
2	**Cat17a**	Br	NO_2	1	92
3	**Cat16b**	Br	NO_2	1	100
4	**Cat17b**	Br	NO_2	1	99
5	**Cat16c**	Br	NO_2	1	94
6	**Cat17c**	Br	NO_2	1	93
7	**Cat16d**	Br	NO_2	1	99
8	**Cat16e**	Br	NO_2	1	95
9	**Cat16f**	Br	NO_2	1	100
10	**Cat16a**	Br	MeO	1	57
11	**Cat17a**	Br	MeO	1	53
12	**Cat16b**	Br	MeO	1	59
13	**Cat17b**	Br	MeO	1	69
14	**Cat16c**	Br	MeO	1	57
15	**Cat17c**	Br	MeO	1	60
16	**Cat16d**	Br	MeO	1	63
17	**Cat16e**	Br	MeO	1	75
18	**Cat16f**	Br	MeO	1	83

Table S25. Results of Reaction 4c [S259].

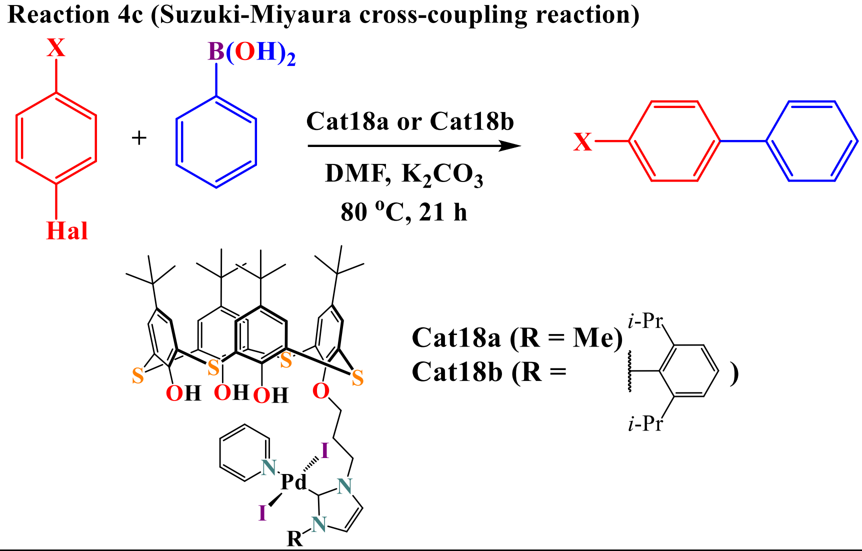

Entry	R	Hal	X	Concentration of catalyst (mol%)	Yield (%)
1	**Cat18a**	I	MeO	1	14
2	**Cat18a**	I	NO_2	1	35
3	**Cat18a**	Br	MeO	1	3
4	**Cat18b**	Br	MeO	1	37
5	**Cat18b**	I	MeO	1	61
6	**Cat18b**	Br	Me	1	56
7	**Cat18b**	Br	Mes	1	23
8	**Cat18b**	Br	Me_2CO	1	99

Table S26. Results of Reaction 4d [S156].

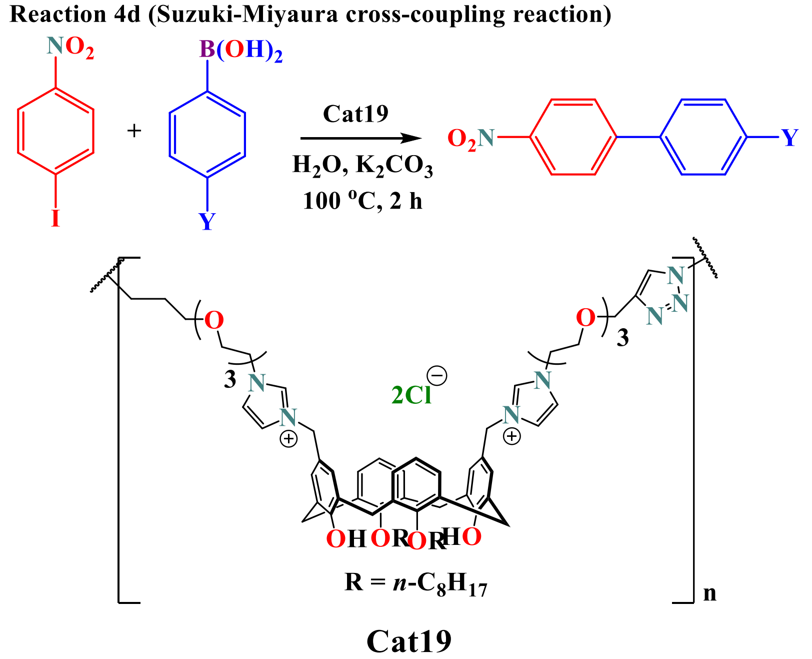

Entry	Catalyst	Y	Yield (%)
1	**Cat19**	H	94
2	**Cat19**	OMe	96
3	**Cat19**	C(O)Me	54
4	$Pd(OAc)_2$	H	51
5	$Pd(OAc)_2$	OMe	42
6	$Pd(OAc)_2$	C(O)Me	15

Table S27. Results of Reaction 4e [S260].

Reaction 4e (Suzuki-Miyaura cross-coupling reaction)

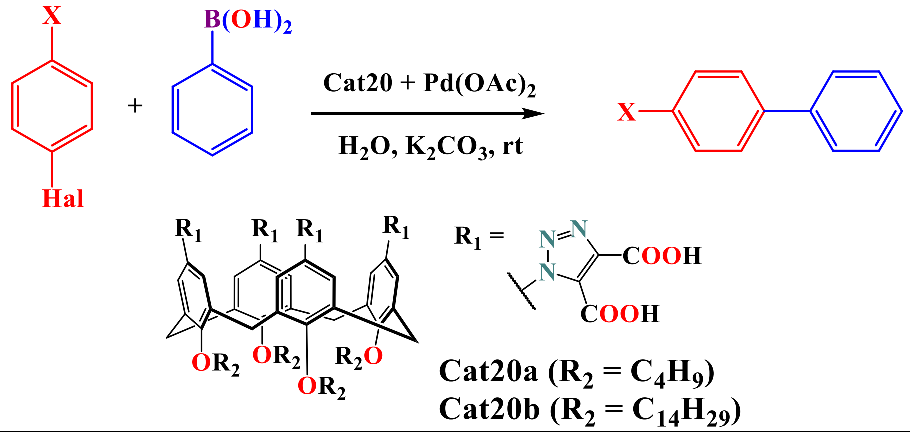

Entry	X	Hal	Catalyst	Time (h)	Yield (%)
1	NO_2	I	$Pd(OAc)_2$	10	2
2	NO_2	I	Na-stearate + $Pd(OAc)_2$	10	71
3	NO_2	I	**Cat20a** + $Pd(OAc)_2$	5	5
4	NO_2	I	**Cat20a** + $Pd(OAc)_2$	10	11
5	NO_2	I	**Cat20b** + $Pd(OAc)_2$	5	78
6	NO_2	I	**Cat20b** + $Pd(OAc)_2$	10	99
7	C(O)Me	Br	**Cat20b** + $Pd(OAc)_2$	10	99
8	NO_2	Br	**Cat20b** + $Pd(OAc)_2$	10	91
9	MeO	Br	**Cat20b** + $Pd(OAc)_2$	10	65
10	NO_2	Cl	**Cat20b** + $Pd(OAc)_2$	10	20

Table S28. Results of Reaction 5 [S48, S49].

Reaction 5 (hydroformylation reaction)

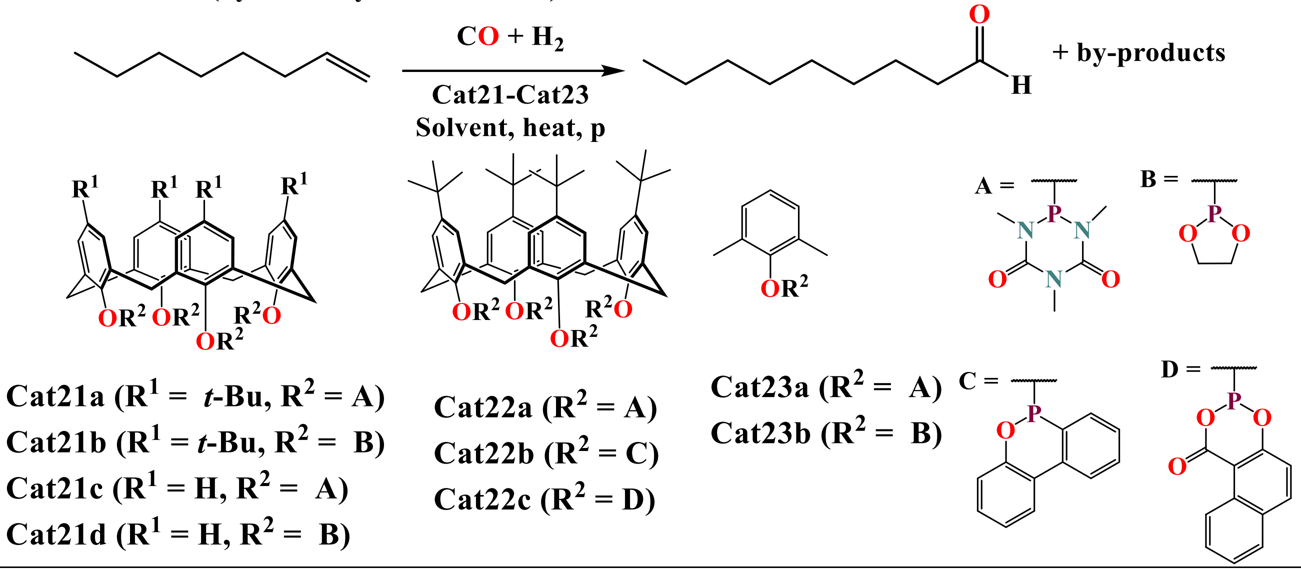

Entry	Ligand	Temperature (°C)	P (bar)	Solvent	Rh:L	Yield (%)	n/iso
1	**Cat21a**	120	50	Toluene	1:1	92.1	0.73
2	**Cat21a**	120	50	THF	1:1	95.0	0.87
3	**Cat21a**	120	50	THF	1:10	89.4	1.61
4	**Cat22a**	100	50	THF	1:1	75.7	0.92
5	**Cat22a**	100	50	THF	1:2	88.4	1.01
6	**Cat22a**	100	50	THF	1:10	86.6	1.36
7	**Cat21c**	100	40	DCM	1:1	99.0	0.77
8	**Cat21c**	100	40	DCM	1:10	4.0	2.31
9	**Cat22b**	100	40	DCM	1:4	95.9	0.78
10	**Cat22b**	100	40	DCM	1:40	99.0	1.43
11	**Cat21b**	120	40	THF	1:1	39.6	2.19
12	**Cat21b**	120	40	THF	1:5	3.4	0.0
13	**Cat21d**	100	40	DCM	1:1	27.4	2.58
14	**Cat22c**	100	40	DCM	1:4	92.4	1.54
15	**Cat23a**	100	30	THF	1:0.5	80.9	0.72
16	**Cat23a**	100	30	THF	1:1	99.9	0.98
17	**Cat23a**	100	30	THF	1:2	90.8	1.07
18	**Cat23a**	100	30	THF	1:10	54.2	1.73
19	**Cat23b**	100	30	THF	1:1	83.3	1.15
20	**Cat23b**	100	30	THF	1:2	85.4	1.43
21	**Cat23b**	100	30	THF	1:10	46.0	1.45

Table S29. Results of Reaction 6 [S161].

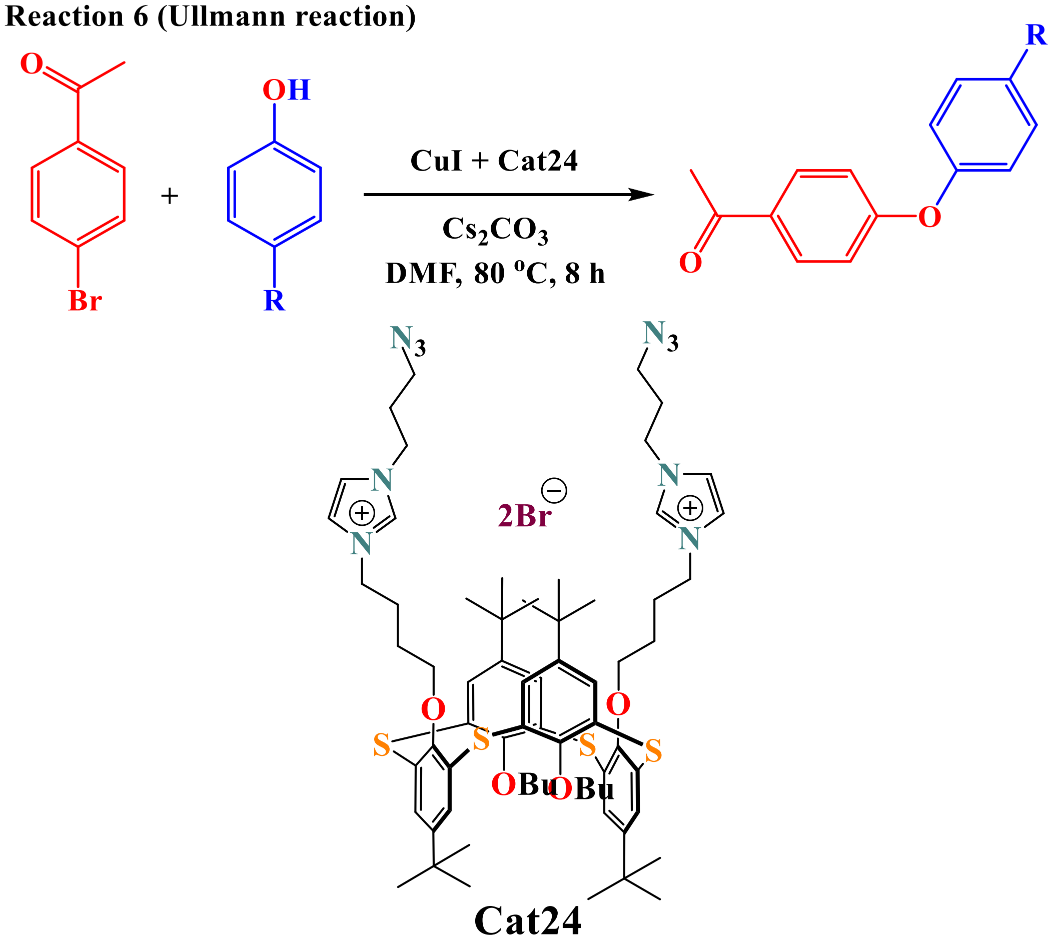

Entry	Catalyst	R	Yield (%)
1	CuI	H	52
2	**Cat24**+ CuI	H	71
3	**Cat24**+ CuI	*t*-Bu	86
4	**Cat24**+ CuI	MeO	99
5	**Cat24**+ CuI	NO_2	15

Table S30. Results of Reaction 7 [S261].

Reaction 7 (oxidative hydroxylation)

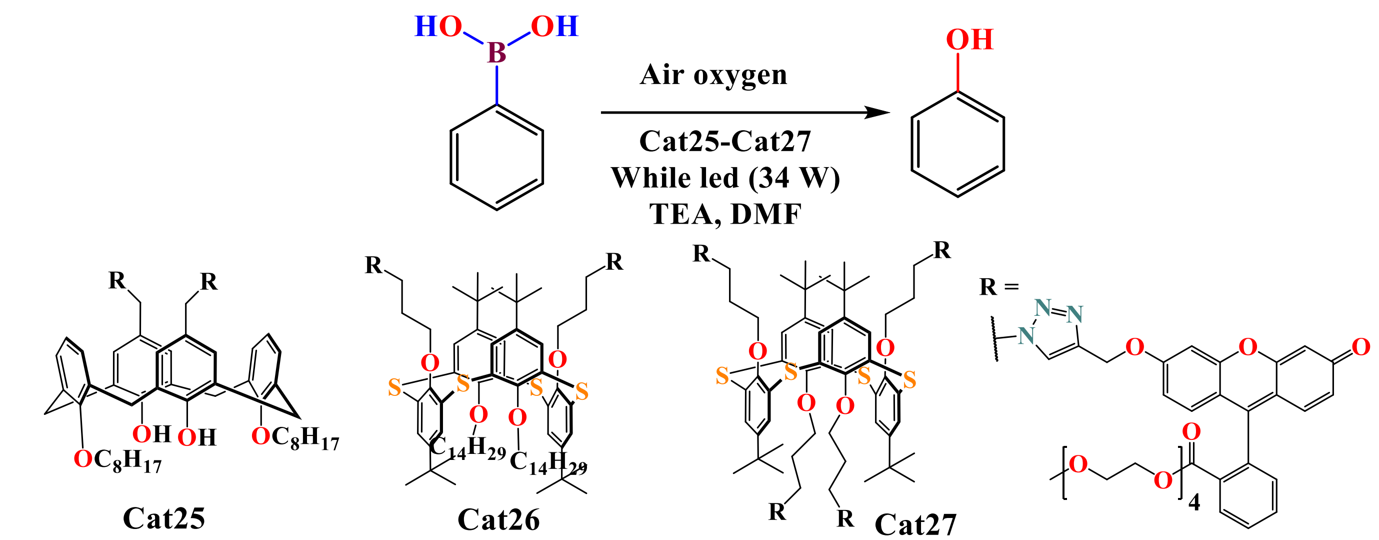

Entry	Catalyst	Concentration of catalyst (μM)	Yield (%)
1	**Cat25**	12	20
2	**Cat25**	25	41
3	**Cat25**	50	51
4	**Cat26**	12	52
5	**Cat26**	25	79
6	**Cat26**	50	93
7	**Cat27**	12	36
8	**Cat27**	25	48
9	**Cat27**	50	86

Table S31. Results of Reaction 8 [S91].

Reaction 8 (azide-alkyne coupling)

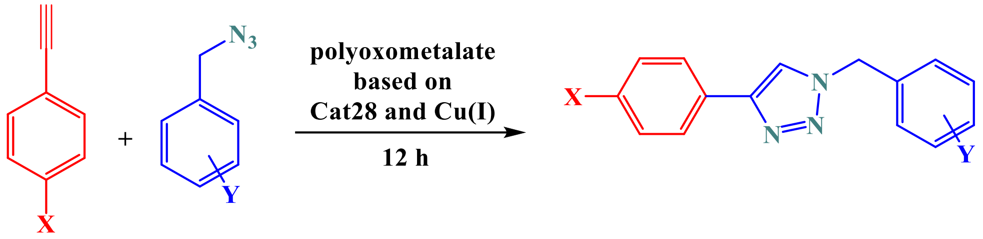

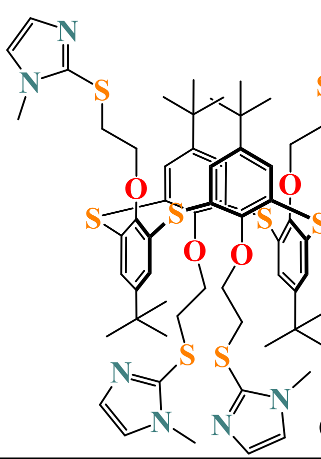

Cat28

Entry	X	Y	Conversion (%)
1	H	3-Me	97
2	H	4-Me	95
3	H	2-F	94
4	H	4-CN	94
5	H	4-NO_2	92
6	OCH_3	H	97
7	Cl	H	86
8	F	H	59

Table S32. Results of Reaction 9 [S91].

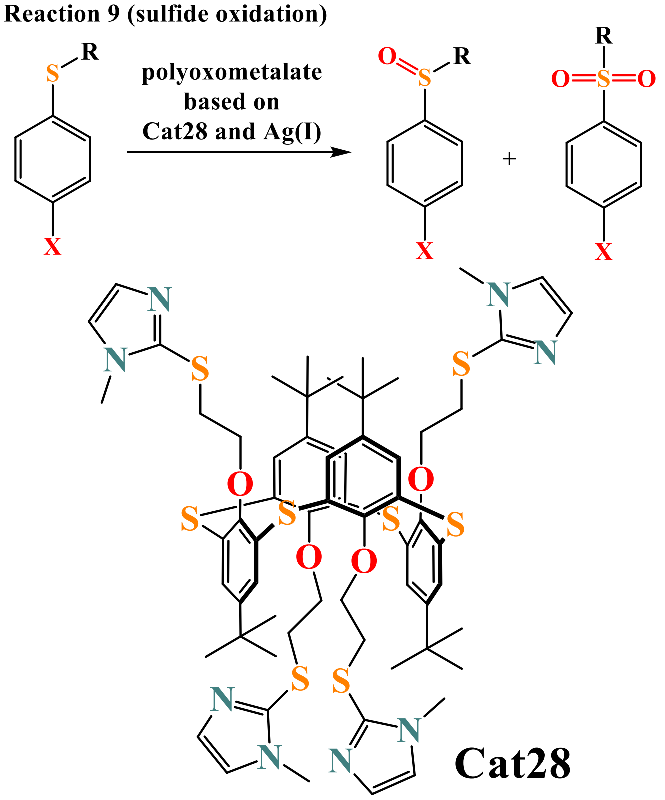

Entry	R	X	Time (h)	Conversion (%)
1	$-CH_2CH_3$	H	4	>99
2	$-CH_2$-CH=CH_2	H	4	>99
3	$-CH_3$	Cl	4	>99
4	$-CH_3$	Br	4	>99
5	$-CH_3$	OCH_3	4	>99

Table S33. Details of biological activity of **HetCA**s.

Heterocyclic fragment	Type of activity	Notices	Ref.
Quinolone, Penicillin	Antibacterial	*S. aureus*, *E. faecalis*, *E. coli*, *P. aeruginosa*	[S63]
Pyran (Carbohydrate moiety)	Lectins inhibition	Broad spectrum of action, mainly antibacterial	[S126, S127, S129, S132]
Quinolone	Antibacterial, targeted delivery of antibiotics	Absorption at the air-carbonate buffer interface and release at 20 and 37 °C	[S52]
1,3,4-oxadiazole	Antifungal, antibacterial	*E. coli*, *A. fumigatus*	[S38]
Bithiazole	Antiviral	Various viral strains	[S29]
Furan, Tetrahydrofuran, Morpholine	Binding to DNA pBR322 plasmids	-	[S74]
Pyrrolidine	Lectins inhibition	Analogue of imino Carbohydrate moietys	[S110]
Biotin	Antitumor, targeted delivery of doxorubicin	*In vivo* investigations in mice	[S111]
Triazole	Antitumor	IC50 (MCF7, PC-3, HSF)	[S139]
1,8-naphthalimide	Antibacterial	MIC (*E. coli*, *P. aeruginosa*, *K. pneumonia*, *S. aureus*, *P. mirabilis*, *Y. enterocolitica*, *S. lutea*, *B. cereus*, *C. albicans*)	[S164]
aminoglycoside, neomycin, kanamycin, paromomycin	Antibacterial, DNA complexation	*E. coli*, *S. lutea,* HeLa and U87-MG	[S203]
Quinolone (nalidixic acid)	Antibacterial	MIC (*S. aureus*, *K. pneumonia*, *P. aeruginosa*, *E. coli*)	[S262]
Aciclovir	Antiviral	Human herpesvirus	[S263]
Pyridine, quinoline, 1.3-thiazole	Cytostatic	dihomooxacalix[4]arene, IC50 (MCF-7, HepG2, SKOV3, and HeLa), HUVEC	[S264]
1,3,4-oxadiazole and 1,3,4-thiadiazole, Pyridine	Antibacterial, antimycobacterial, antifungal, antioxidant	MIC (*S. aureus*, *S. pyogenes*, *P. aeruginosa*, *E. coli*), *M. tuberculosis* H 37 Rv, MIC (*C. albicans*, *A. clavatus)*	[S265]
Benzotriazol	Antibacterial, antibiofilm	MIC/MBC (*S. aureus*, *MRSA*, *K. pneumonia*, *P. aeruginosa*, *E. coli*), HCT116, *In vivo* toxicity in mice	[S266]
β-lactam (penicillins V and X)	Antibacterial	MIC (*S. pyogenes*, *S. agalactiae*, *S. pneumoniae*)	[S267]
β-lactam (cephalosporins)	Antibacterial	MIC (*S. aureus*)	[S268]
Thiomorpholine	Antibacterial, hemolytic	MIC (*S. aureus, MRSA*)	[S269]
Pyrrolidine	Antibacterial, hemolytic	MIC (*S. aureus, MRSA*)	[S269]
Imidazole	Antibacterial	MIC (*S. aureus*, *K. pneumonia*, *P. aeruginosa*, *E. coli*)	[S270]
furan, tetrahydrofuran, morpholine, pyridine	Antimicrobial	MIC (*B. subtilis*, *B. cereus*, *E. faecalis*, *S. aureus*, *E. coli*, *P. aeruginosa*, *P. vulgaris*), MIC (*C. albicans*, *C. tropicalis*)	[S271]
Bipyridine, Bithiazole	Antibacterial	MIC (*S. aureus*, *K. pneumonia*, *P. aeruginosa*, *E. coli*)	[S272]
Bipyridine, Bithiazole	Antimycobacterial	MIC (*M. tuberculosis* H37Rv, MYC5165)	[S273]
Bithiazole	Antiviral	Human coronavirus 229E	[S274]
3,4-dihydropyrmidine-2-(1*H*)	Antibacterial, antifungal	MIC, MBC, IZ (*S. epidermiditis*, *S. aureus*, *S. pneumonia*, *E. faecalis*, *E. coli*, *P. aeruginosa*, *P. vulgaris*, *H. pylori*), MIC (*C. albicans*, *C. glabrata*, *A. fumigatus*, *A. niger*)	[S275]
Pyrrolidine	Antitumor	IC50 (DLD-1, A-549, HEPG2, PC-3, PNT1A)	[S276]
Isatin	Inhibition of enzymes, antioxidant, antibacterial, antiproliferative	IC50 (PNT1A, MCF-7, HeLa, DLD1, A549, MDA-MB-231), IC50 (COX-2, Aromatase), MIC (*E. coli*, *P. aeruginosa*, *K. pneumonia*, *S. aureus*, *S. enteritidis*, *S. lutea*, *B. cereus*, *C. albicans*)	[S277]
Pyrazole	Antitumor, hemolytic	IC50 (M-HeLa, MCF-7, A-549, PC3, Wi38), Flow cytometric Analysis	[S278]
pyrimidylthioamide	Antimicrobial	Agar disc diffusion method (*E. coli*, *K. pneumoniae*, *P. aeruginosa*, *S. aureus*, *S. typhimurium*, *B. cereus*, *B. subtilis*, *C. albicans*)	[S279]

Table S34. Examples of data of **Het-CA**s association measured via DLS.

Heterocyclic fragment	System	Solvent	Concentration (μM)	D (nm)	PDI	Temperature (°C)	Ref.
Self-association of **HetCA**s							
Indole	HetCA (*cone*)	H_2O	100	120 ± 12	0.26	25	[S280]
Indole	HetCA (*cone*)	H_2O	10	208 ± 27	0.39	25	[S280]
Indole	HetCA (*cone*)	H_2O	1	323 ± 37	0.48	25	[S280]
Indole	HetCA (*1,3-alternate*)	H_2O	100	131 ± 3	0.30	25	[S280]
Indole	HetCA (*1,3-alternate*)	H_2O	10	184 ± 15	0.34	25	[S280]
Indole	HetCA (*1,3-alternate*)	H_2O	1	292 ± 17	0.31	25	[S280]
Indole	HetCA (*cone*)	H_2O	100	134 ± 3	0.21	25	[S280]
Indole	HetCA (*cone*)	H_2O	10	236 ± 10	0.34	25	[S280]
Indole	HetCA (*cone*)	H_2O	1	258 ± 11	0.34	25	[S280]
Indole	HetCA (*1,3-alternate*)	H_2O	100	86 ± 1	0.22	25	[S280]
Indole	HetCA (*1,3-alternate*)	H_2O	10	162 ± 18	0.34	25	[S280]
Indole	HetCA (*1,3-alternate*)	H_2O	1	212 ± 23	0.36	25	[S280]
Fluorescein	HetCA (*1,3-alternate*)	H_2O	1000	587 ± 35	0.43	25	[S281]
Fluorescein	HetCA (*1,3-alternate*)	H_2O	100	433 ± 43	0.51	25	[S281]
Fluorescein	HetCA (*1,3-alternate*)	H_2O	10	285 ± 44	0.44	25	[S281]
Fluorescein	HetCA (*cone*)	H_2O	1000	329 ± 59	0.57	25	[S281]
Fluorescein	HetCA (*cone*)	H_2O	100	353 ± 93	0.64	25	[S281]
Fluorescein	HetCA (*cone*)	H_2O	10	199 ± 45	0.48	25	[S281]
Pyrrolidine	HetCA	MeCN	1000	1111.3 ± 138.9	0.67	25	[S282]
Association of **HetCA**s with different substrates							
Indole	HetCA + BSA	H_2O	3	6.6 ± 0.6	0.14	20	[S163]
Indole	HetCA + Glycine	H_2O	3	18 ± 6	0.43	20	[S236]
Indole	CA + Phenylalanine	H_2O	3	27 ± 5	0.39	20	[S236]
Indole	CA + Tyrosine	H_2O	3	24 ± 4	0.42	20	[S236]
Indole	CA + Tryptophane	H_2O	3	26 ± 4	0.48	20	[S236]
Pyrrolidine	HetCA + $LiNO_3$	MeCN	100	525.5 ± 64.7	0.73	25	[S282]

References

S1. Fischer, C; Sarti, G.; Casnati, A.; Carrettoni, B.; Manet, I.; Schuurman, R.; Guardigli, M.; Sabbatini, N.; Ungaro, R. 2,2′-Bipyridine lariat calixcrowns: A new class of encapsulating ligands forming highly luminescent Eu^{3+} and Tb^{3+} complexes. *Chem. - Eur. J.* **2000**, *6*, 1026–1034. https://doi.org/10.1002/(SICI)1521-3765(20000317)6:6%3C1026::AID-CHEM1026%3E3.0.CO;2-C.

S2. Grigg, R.; Holmes, J.M.; Jones, S.K.; Norbert, W.D. Luminescent pH sensors based on p-tert-butylcalix[4]arene-linked ruthenium(II) trisbipyridyl complexes. *J. Chem. Soc. Chem. Commun.* **1994**, *30*, 185–187. https://doi.org/10.1039/C39940000185.

S3. Oueslati, F.; Dumazet-Bonnamour, I.; Lamartine, R. Synthesis of new chromogenic 2,2′-bithiazoylcalix[4]arenes. *Tetrahedron Lett.* **2001**, *42*, 8177–8180. https://doi.org/10.1016/S0040-4039(01)01755-5.

S4. Pellet-Rostaing, S.; Regnouf-de-Vains, J.; Lamartine, R. 2,2′-Bithiazolyl-p-tert-butylcalix[4]arene podands. Synthesis and fluorescence properties. *Tetrahedron Lett.* **1996**, *37*, 5889–5892. https://doi.org/10.1016/0040-4039(96)01269-5.

S5. Marchand, A.P.; Chong, H.; Takhi, M.; Power, T.D. Synthesis and alkali metal picrate extraction studies of p-tert-butylcalix[4]arene crown ethers bridged at the lower rim with pyridyl units. *Tetrahedron* **2000**, *56*, 3121–3126. https://doi.org/10.1016/S0040-4020(00)00160-5.

S6. Casnati, A.; Sansone, F.; Sartori, A.; Prodi, L.; Montalti, M.; Zaccheroni, N.; Ugozzoli, F.; Ungaro, R. Quinoline-containing calixarene fluoroionophores: A combined NMR, photophysical and modeling study. *Eur. J. Org. Chem.* **2003**, *2003*, 1475–1485. https://doi.org/10.1002/ejoc.200390206.

S7. Oueslati, F.; Dumazet-Bonnamour, I.; Lamartine, R. New azothiacalix[4]arenes containing biheterocyclic subunits: Extraction and complexation properties. *Supramol. Chem.* **2005**, *17*, 227–232. https://doi.org/10.1080/10610270412331331679.

S8. Cante-Mota, I.; Moreno-Alcántar, G.; Flores-Alamo, M.; Castillo, I. Benzimidazole-derived calix[4]arenes with polymerizable styrene groups and their Cu(II) complexes. *Inorganica Chim. Acta* **2013**, *407*, 11–18. https://doi.org/10.1016/j.ica.2013.07.043.

S9. Ni, X.; Cong, H.; Yoshizawa, A.; Rahman, S.; Tomiyasu, H.; Rayhan, U.; Zeng, X.; Yamato, T. Heteroditopic thiacalix[4]arene receptor having ester and bipyridyl moieties for ions binding with positive/negative allosteric effect. *J. Mol. Struct.* **2013**, *1046*, 110–115. https://doi.org/10.1016/j.molstruc.2013.04.040.

S10. Huzur, A.; Kursunlu, A.N.; Ozkan, S.C.; Aksakal, F.; Yilmaz, A. Novel BODIPY-calix[4]arene fluorophores: DNA interaction, spectroscopic properties, electrophorese and molecular docking. *J. Mol. Struct.* **2024**, *1316*, 138999. https://doi.org/10.1016/j.molstruc.2024.138999.

S11. Leko, K.; Usenik, A.; Cindro, N.; Modrušan, M.; Požar, J.; Horvat, G.; Stilinović, V.; Hrenar, T.; Tomišić, V. Enhancing the cation-binding ability of fluorescent calixarene derivatives: Structural, thermodynamic, and computational studies. *ACS Omega* **2023**, *8*, 43074–43087. https://doi.org/10.1021/acsomega.3c06509.

S12. Vreekamp, R.H.; Verboom, W.; Reinhoudt, D.N. Lower rim−upper rim hydrogen-bonded adducts of calix[4]arenes. *J. Org. Chem.* **1996**, *61*, 4282–4288. https://doi.org/10.1021/jo9603082.

S13. Yamato, T.; Casas, C.P.; Rahman, S.; Gil, J.T.; Elsegood, M.R.; Redshaw, C. Synthesis, structures and inclusion properties of distal -bis-[(2-pyridylmethyl)oxy]tetrathiacalix[4]arenes. *J. Chem. Res.* **2006**, *2006*, 270–273. https://doi.org/10.3184/030823406776894247.

S14. Zhang, F.; Kumamaru, K.; Yamamoto, H. Synthesis, conformational studies and inclusion properties of tetrakis[(2-pyridylmethyl)oxy]thiacalix[4]arenes. *J. Incl. Phenom. Macrocycl. Chem.* **2002**, *42*, 51–60. https://doi.org/10.1023/A:1014540609454.

S15. Zairov, R.R.; Nagimov, R.N.; Sudakova, S.N.; Lapaev, D.V.; Syakaev, V.V.; Gimazetdinova, G.S.; Voloshina, A.D.; Shykula, M.; Nizameev, I.R.; Samigullina, A.I.; Gubaidullin, A.T.; Podyachev, S.N.; Mustafina, A.R. Polystyrenesulfonate-coated nanoparticles with low cytotoxicity for determination of copper(II) via the luminescence of Tb(III) complexes with new calix[4]arene derivatives. *Microchim. Acta* **2018**, *185*, 386. https://doi.org/10.1007/s00604-018-2923-2.

S16. Pappalardo, S.; Giunta, L.; Foti, M.; Ferguson, G.; Gallagher, J.F.; Kaitner, B. Functionalization of calix[4]arenes by alkylation with 2-(chloromethyl)pyridine hydrochloride. *J. Org. Chem.* **1992**, *57*, 2611–2624. https://doi.org/10.1021/jo00035a016.

S17. Beer, P.D.; Martin, J.P.; Drew, M.G. Calix[4]arene cryptand and new 1,3-bis-pyridyl,-bipyridyl and -alkylthioether calix[4]arenes designed to coordinate transition metal cations. *Tetrahedron* **1992**, *48*, 9917–9928. https://doi.org/10.1016/S0040-4020(01)92282-3.

S18. Arena, G.; Contino, A.; Longo, E.; Sciotto, D.; Sgarlata, C.; Spoto, G. Synthesis, characterization of a novel calixarene having dipyridyl pendants and study of its complexes with Cu(II) and Co(II). *Tetrahedron Lett.* **2003**, *44*, 5415–5418. https://doi.org/10.1016/S0040-4039(03)01319-4.

S19. Arena, G.; Contino, A.; Maccarrone, G.; Sciotto, D.; Sgarlata, C. Rendering a calixarene having dipyridyl pendants soluble in water results in different species with smaller binding constants. *Tetrahedron Lett.* **2007**, *48*, 8274–8276. https://doi.org/10.1016/j.tetlet.2007.09.131.

S20. Psychogios, N.; Regnouf-de-Vains, J. A new water-soluble ligand based on a calix[4]arene substituted by 2,2′-bipyridine chelating units. *Tetrahedron Lett.* **2001**, *42*, 2799–2800. https://doi.org/10.1016/S0040-4039(01)00306-9.

S21. Psychogios, N.; Regnouf-de-Vains, J.; Stoeckli-evans, H.M. A water-soluble calix[4]arene-based podand incorporating 4,4′-dicarboxy-2,2′-bipyridine chelating units − synthesis and complexation properties towards copper ions. *Eur. J. Inorg. Chem.***2004**, *2004*, 2514–2523. https://doi.org/10.1002/ejic.200300690.

S22. Mourer, M.; Regnouf-de-Vains, J. New water-soluble calix[4]arene-based bipyridyl podands incorporating carboxylate groups. *Tetrahedron Lett.* **2006**, *47*, 1895–1899. https://doi.org/10.1016/j.tetlet.2006.01.088.

S23. Psychogios, N.; Regnouf-de-Vains, J. A new water-soluble calix[4]arene podand incorporating p-phosphonate groups and 2,2′-bipyridine units. *Tetrahedron Lett.* **2002**, *43*, 7691–7693. https://doi.org/10.1016/S0040-4039(02)01843-9.

S24. Canevet, C.; Libman, J.; Shanzer, A. Molecular redox-switches by ligand exchange. *Angew. Chemie Int. Ed. English* **1996**, *35*, 2657–2660. https://doi.org/10.1002/anie.199626571.

S25. Dorta, R.; Shimon, L.J.; Rozenberg, H.; Ben-David, Y.; Milstein, D. A new ligand system based on a bipyridine-functionalized calix[4]arene backbone leading to mono- and bimetallic complexes. *Inorg. Chem.* **2003**, *42*, 3160–3167. https://doi.org/10.1021/ic020643a.

S26. Regnouf-de-Vains, J.; Dalbavie, J.; Lamartine, R.; Fenet, B. Quantitative solvent extraction from neutral aqueous nitrate media of silver(I) against lead(II) with a new calix[4]arene-based bipyridine podand. *Tetrahedron Lett.* **2001**, *42*, 2681–2684. https://doi.org/10.1016/S0040-4039(01)00262-3.

S27. Bottino, F.; Giunta, L.; Pappalardo, S. Calix[4]arenes with pyridine pendant groups. Regioselective proximal alkylation at the "lower rim". *J. Org. Chem.* **1989**, *54*, 5407–5409. https://doi.org/10.1021/jo00284a001.

S28. Vreekamp, R.H.; Verboom, W.; Reinhoudt, D.N. Aggregates of calix[4]arenes based on the 2,6-diaminotriazine imide system. *Recl. des Trav. Chim. des Pays-bas* **1996**, *115*, 363–370. https://doi.org/10.1002/recl.19961150705.

S29. Mourer, M.; Psychogios, N.; Laumond, G.; Aubertin, A.; Regnouf-de-Vains, J. Synthesis and anti-HIV evaluation of water-soluble calixarene-based bithiazolyl podands. *Bioorg. Med. Chem.* **2010**, *18*, 36–45. https://doi.org/10.1016/j.bmc.2009.11.016.

S30. Zeng, K.; Yang, Y.; Xu, J.; Wang, N.; Tang, W.; Xu, J.; Zhang, Y.; Wu, Y.; Xu, Y.; Wang, G.; Chen, P.; Wang, B.; Sun, X.; Jin, G.; Peng, H. Metal-backboned polymers with well-defined lengths. *Angew. Chemie Int. Ed.* **2023**, *62*, e202216060. https://doi.org/10.1002/anie.202216060.

S31. de Namor, A.F.D.; Piro, O.E.; Salazar, L.E.P.; Aguilar-Cornejo, A.F.; Al-Rawi, N.; Castellano, E.E.; Velarde, F.J.S. Solution thermodynamics of geometrical isomers of pyridino calix(4)arenes and their interaction with the silver cation. The X-ray structure of a 1:1 complex of silver perchlorate and acetonitrile with 5,11,17,23-tetra-tert-butyl-[25,26,27,28-tetrakis(2- pyridylmethyl)oxy]calix(4)arene. *J. Chem. Soc. Faraday Trans.* **1998**, *94*, 3097–3104. https://doi.org/10.1039/A805430D.

S32. de Namor, A.F.D.; Aguilar-Cornejo, A.; Soualhi, R.; Shehab, M.; Nolan, K.B.; Ouazzani, N.; Mandi, L. A calix(4)arene pyridine derivative and its monomeric component: Structural and thermodynamic aspects of their complexation with metal cations. *J. Phys. Chem. B* **2005**, *109*, 14735–14741. https://doi.org/10.1021/jp0514630.

S33. Oueslati, F.; Dumazet-Bonnamour, I.; Lamartine, R. New chromogenic azocalix[4]arene podands incorporating 2,2′-bipyridyl subunits. *New J. Chem.* **2003**, *27*, 644–647. https://doi.org/10.1039/B209528A.

S34. Casnati, A.; Fischer, C.; Guardigli, M.; Isernia, A.; Manet, I.; Sabbatini, N.; Ungaro, R. Synthesis of calix[4]arene receptors incorporating (2,2′-bipyridin-6-yl)methyl and (9-methyl-1,10-phenanthrolin-2-yl)methyl chromophores and luminescence of their Eu^{3+} and Tb^{3+} complexes. *J. Chem. Soc., Perkin Trans. 2* **1996**, *25*, 395–399. https://doi.org/10.1039/P29960000395.

S35. Ziessel, R.F.; Charbonnière, L.J.; Cesario, M.; Prangé, T.; Guardigli, M.; Roda, A.; Van dorsselaer, A.; Nierengarten, H. p-tert-Butylcalix[4]arene functionalised with bipyridyl carboxylates for lanthanide complexation: Synthesis, photophysical properties, solution and solid state behavior. *Supramol. Chem.* **2003**, *15*, 277–289. https://doi.org/10.1080/1061027021000057224.

S36. Ovsyannikov, A.; Lang, M.N.; Ferlay, S.; Solovieva, S.E.; Antipin, I.S.; Konovalov, A.I.; Kyritsakas, N.; Hosseini, M.W. Molecular tectonics: Pyridyl containing thiacalix[4]arene based tectons for the generation of 2- and 3-D silver coordination networks. *Dalton Trans.* **2013**, *42*, 116–126. https://doi.org/10.1039/c2dt31937c.

S37. Casas, C.P.; Yamato, T. Synthesis and Structures of [(4-pyridylmethyl)oxy]tetrathiacalix[4]arenes derived from O-alkylation of tetrathiacalix[4]arene with 4-(chloromethyl)pyridine. *J. Chem. Res.* **2005**, *2005*, 575–577. https://doi.org/10.3184/030823405774308844.

S38. Gezelbash, Z.D.; Dilmaghani, K.A. Synthesis, antifungal and antibacterial activity of calix[4]arene-based 1,3,4-oxadiazole derivatives. *J. Chinese Chem. Soc.* **2020**, *67*, 1446–1452. https://doi.org/10.1002/jccs.201900425.

S39. Yang, F.; Wu, Y.; Ye, J.; Guo, H.; Yan, X. Novel calixarene benzo-15-crown-5 derivatives: Synthesis and complexation for dyes. *J. Macromol. Sci. Part A* **2014**, *51*, 223–228. https://doi.org/10.1080/10601325.2014.871952.

S40. Lee, S.H.; Kim, H.J.; Lee, Y.O.; Vicens, J.; Kim, J.S. Fluoride sensing with a PCT-based calix[4]arene. *Tetrahedron Lett.* **2006**, *47*, 4373–4376. https://doi.org/10.1016/j.tetlet.2006.04.093.

S41. Aktas, M.; Uyanik, A.; Eymur, S.; Yilmaz, M. L-proline derivatives based on a calix[4]arene scaffold as chiral organocatalysts for the direct asymmetric aldol reaction in water. *Supramol. Chem.* **2016**, *28*, 351–359. https://doi.org/10.1080/10610278.2015.1073288. S42. Mummidivarapu, V.V.; Tabbasum, K.; Chinta, J.P.; Rao, C.P. 1,3-Di-amidoquinoline conjugate of calix[4]arene (L) as a ratiometric and colorimetric sensor for Zn^{2+}: Spectroscopy, microscopy and computational studies. *Dalton Trans.* **2012**, *41*, 1671–1674. https://doi.org/10.1039/c2dt11900e.

S43. Sharma, V.S.; Shah, P.A.; Sharma, A.S.; Ganga, V.S.R.; Shrivastav, P.S.; Prajapat, V. Upper/lower rim functionalized calixarene based AIE-active liquid crystals with self-assembly behavior: Photophysical and electrochemical studies. *J. Mol. Liq.* **2022**, *348*, 118047. https://doi.org/10.1016/j.molliq.2021.118047.

S44. Bayrakcı, M.; Yiğiter, Ş. Synthesis of tetrasubstituted calix[4]arene ionophores and their recognition studies toward toxic arsenate anions. *Tetrahedron* **2013**, *69*, 3218–3224. https://doi.org/10.1016/j.tet.2013.02.050.

S45. Ali, K.A.; Abdel hafez, N.A.; Elsayed, M.A.; Ibrahim, A.A. Microwave-assisted synthesis and heterocyclic functionalization of chromenopyridines on calixarene scaffold. *J. Heterocycl. Chem.* **2020**, *57*, 1838–1844. https://doi.org/10.1002/jhet.3912.

S46. Fu, Y.; Zeng, X.; Mu, L.; Jiang, X.; Deng, M.; Zhang, J.; Yamato, T. Use of a new thiacalix[4]arene derivative bearing two 4-chloro-7-nitrobenzofurazan groups as a colorimetric and fluorescent chemosensor for Ag^+ and AcO^-. *Sensors Actuators B: Chemical* **2012**, *164*, 69–75. https://doi.org/10.1016/j.snb.2012.01.071.

S47. Halay, E.; Acikbas, Y.; Capan, R.; Bozkurt, S.; Erdogan, M.; Unal, R. A novel triazine–bearing calix[4]arene: Design, synthesis and gas sensing affinity for volatile organic compounds. *Tetrahedron* **2019**, *75*, 2521–2528. https://doi.org/10.1016/j.tet.2019.03.027.

S48. Kunze, C.; Selent, D.; Neda, I.; Schmutzler, R.; Spannenberg, A.; Börner, A. Synthesis of new calix[4]arene-based phosphorus ligands and their application in the Rh(I) catalyzed hydroformylation of 1-octene. *Heteroat. Chem.* **2001**, *12*, 577–585. https://doi.org/10.1002/hc.1088.

S49. Kunze, C.; Selent, D.; Neda, I.; Freytag, M.; Jones, P.G.; Schmutzler, R.; Baumann, W.; Börner, A. Calix[4]arene-based bis-phosphonites, bis-phosphites, and bis-O-acyl-phosphites as ligands in the rhodium(I)-catalyzed hydroformylation of 1-octene. *Z. Anorg. Allg. Chem.* **2002**, *628*, 779–787. https://doi.org/10.1002/1521-3749(200205)628:4%3C779::AID-ZAAC779%3E3.0.CO;2-V.

S50. Dondoni, A.; Marra, A. Addressing the scope of the azide–nitrile cycloaddition in glycoconjugate chemistry. The assembly of C-glycoclusters on a calix[4]arene scaffold through tetrazole spacers. *Tetrahedron* **2007**, *63*, 6339–6345. https://doi.org/10.1016/j.tet.2007.03.045.

S51. Stoikov, I.I.; Galukhin, A.V.; Zaikov, E.N.; Antipin, I.S. Synthesis and complexation properties of 1,3-alternate stereoisomers of p-tert-butylthiacalix[4]arenes tetrasubstituted at the lower rim by the phthalimide group. *Mendeleev Commun.* **2009**, *19*, 193–195. https://doi.org/10.1016/j.mencom.2009.07.006.

S52. Sautrey, G.; Clarot, I.; Salem, A.B.; Rogalska, E.; Regnouf de vains, J. Organosoluble calixarene-based quinolone carriers: syntheses, evaluation and model hydrolytic studies at the air–water interface. *New J. Chem.* **2012**, *36*, 78–85. https://doi.org/10.1039/c1nj20636b.

S53. Rizzo, S.; Sannicolò, F.; Benincori, T.; Schiavon, G.; Zecchin, S.; Zotti, G. Calix[4]arene-functionalized poly-cyclopenta[2,1-b;3,4-b′]bithiophenes with good recognition ability and selectivity for small organic molecules for application in QCM-based sensors. *J. Mater. Chem.* **2004**, *14*, 1804–1811. https://doi.org/10.1039/B314345G.

S54. Marsella, M.J.; Newland, R.J.; Carroll, P.J.; Swager, T.M. Ionoresistivity as a highly sensitive sensory probe: investigations of polythiophenes functionalized with calix[4]arene-based ion receptors. *J. Am. Chem. Soc.* **1995**, *117*, 9842–9848. https://doi.org/10.1021/ja00144a009.

S55. Zhou, X.; Chen, W.; Song, Y. Superhydrophobic polyoxometalate/calixarene inorganic–organic hybrid materials with highly efficient desulfurization ability. *Eur. J. Inorg. Chem.* **2014**, *2014*, 812–817. https://doi.org/10.1002/ejic.201301407.

S56. de Namor, A.F.; Velarde, F.J.; Cabaleiro, M.C. New lower rim calix[4]arene amine derivatives: synthesis, characterisation and acid–base properties. Potential anion and cation binders. *J. Chem. Soc., Faraday Trans.* **1996**, *92*, 1731–1737. https://doi.org/10.1039/FT9969201731.

S57. Fatahi, A.; Mohammadi, A.; Isfahani, A.P. Multifunctional, robust, and self-healable polyurethane coatings cross-linked by Diels–Alder reaction of calix[4]arenes containing furan groups. *J. Appl. Polym. Sci.* **2024**, *141*, e54825. https://doi.org/10.1002/app.54825.

S58. Galukhin, A.V.; Zaikov, E.N.; Antipin, I.S.; Konovalov, A.I.; Stoikov, I.I. Mono-, 1,3-di- and tetrasubstituted p-tert-butylthiacalix[4]arenes containing phthalimide groups: Synthesis and functionalization with ester, amide, hydrazide and amino groups. *Macroheterocycles* **2012**, *5*, 266–274. https://doi.org/10.6060/mhc2012.120781s.

S59. Quiroga-Campano, C.; Gómez-Machuca, H.; Moris, S.; Pessoa-Mahana, H.; Jullian, C.; Saitz, C. Synthesis of calix[4]arenes bearing thiosemicarbazone moieties with naphthalene groups: Highly selective turn off/on fluorescent sensor for Cu(II) recognition. *J. Mol. Struct.* **2021**, *1225*, 129125. https://doi.org/10.1016/j.molstruc.2020.129125.

S60. Casnati, A.; Della ca', N.; Fontanella, M.; Sansone, F.; Ugozzoli, F.; Ungaro, R.; Liger, K.; Dozol, J. Calixarene-based picolinamide extractants for selective An/Ln separation from radioactive waste. *Eur. J. Org. Chem.* **2005**, *2005*, 2338–2348. https://doi.org/10.1002/ejoc.200400793.

S61. Minhas, F.T.; Memon, S.; Bhanger, M.I. Synthesis and characterization of novel calix[4]arene piperazine derivative for the extraction of transition metals and dichromate ions. *J. Incl. Phenom. Macrocycl. Chem.* **2010**, *67*, 295–302. https://doi.org/10.1007/s10847-009-9709-4.

S62. Li, L.; Du, L.; Sun, J.; Yan, C. Synthesis, crystal structure of bis-terpyridinyl-calix[4]arene derivatives and fluorescent sensor for Zn^{2+}. *Chem. Res. Chinese Univ.* **2013**, *29*, 874–878. https://doi.org/10.1007/s40242-013-3150-3.

S63. Ben salem, A.; Sautrey, G.; Fontanay, S.; Duval, R.E.; Regnouf-de-Vains, J. Molecular drug-organiser: Synthesis, characterization and biological evaluation of penicillin V and/or nalidixic acid calixarene-based podands. *Bioorg. Med. Chem.* **2011**, *19*, 7534–7540. https://doi.org/10.1016/j.bmc.2011.10.031.

S64. Zhao, B.; Zhu, X.; Peng, Q.; Yan, Z.; Derf, F.; Sallé, M. A novel redox-active calix[4]arene-tetrathiafulvalene dyad. *Open Chem.* **2011**, *9*, 1102–1108. https://doi.org/10.2478/s11532-011-0104-9.

S65. GüngöӦEfficient removal of carcinogenic azo dyes by novel pyrazine-2-carboxylate substituted calix[4, 8]arene derivatives. *Supramol. Chem.* **2019**, *31*, 776–783. https://doi.org/10.1080/10610278.2020.1711908.

S66. Kim, E.J.; Ahn, J.; Lee, H.; Noh, T.H.; Jung, O. Synthesis, structures, and ligating ability of 4-tert-butylcalix[n]arene (iso)nicotinoylate. *Tetrahedron Lett.* **2012**, *53*, 1240–1244. https://doi.org/10.1016/j.tetlet.2011.12.112.

S67. Liu, Y.; Zhao, B.; Chen, L.; He, X. Liquid membrane transport and silver selective electrode based on novel bis(3-pyridinecarboxylate) calix[4]arene as ionophore. *Microchem. J.* **2000**, *65*, 75–79. https://doi.org/10.1016/S0026-265X(00)00033-3.

S68. Mcconnell, A.J.; Serpell, C.J.; Beer, P.D. Extending the family of heteroditopic calix[4]diquinone receptors for cooperative and ion-pair recognition. *New J. Chem.* **2012**, *36*, 102–112. https://doi.org/10.1039/c1nj20708c.

S69. Phipps, D.E.; Beer, P.D. A [2]catenane containing an upper-rim functionalized calix[4]arene for anion recognition. *Tetrahedron Lett.* **2009**, *50*, 3454–3457. https://doi.org/10.1016/j.tetlet.2009.02.212.

S70. Lee, H.; Noh, T.H.; Kim, E.J.; Jung, O. Unusually rigid macrocyclic tris(picolinolato)-4-tert-butylcalix[4]arene: Its intramolecular interaction effect on picolinoylation. *J. Mol. Struct.* **2012**, *1027*, 53–56. https://doi.org/10.1016/j.molstruc.2012.06.015.

S71. Modi, K.; Panchal, U.; Dey, S.; Patel, C.; Kongor, A.; Pandya, H.A.; Jain, V.K. Thiacalix[4]arene-tetra-(quinoline-8-sulfonate): A sensitive and selective fluorescent sensor for Co (II). *J. Fluoresc.* **2016**, *26*, 1729–1736. https://doi.org/10.1007/s10895-016-1864-6. S72. Zeng, X.; Weng, L.; Chen, L.; Xu, F.; Li, Q.; Leng, X.; He, X.; Zhang, Z. The syntheses and Ag^{+}-selective electrode properties of benzothiazolylthiaalkoxy functionalized calix[4]arenes: an investigation of the structure–selectivity relationship in the ionophore-based ISEs. *Tetrahedron* **2002**, *58*, 2647–2658. https://doi.org/10.1016/S0040-4020(02)00124-2.

S73. Joseph, R.; Chinta, J.P.; Rao, C.P. Benzothiazole appended lower rim 1,3-di-amido-derivative of calix[4]arene: Synthesis, structure, receptor properties towards Cu^{2+}, iodide recognition and computational modeling. *Inorganica Chim. Acta* **2010**, *363*, 2833–2839. https://doi.org/10.1016/j.ica.2010.04.005.

S74. Özkan, Ş.Ç.; Yilmaz, A.; Özmen, İ. Synthesis of new calix[4]arene amide derivatives and investigation of their DNA cleavage activity. *Supramol. Chem.* **2014**, *26*, 25–31. https://doi.org/10.1080/10610278.2013.817578.

S75. Bolshchikov, B.; Volkov, S.; Sokolova, D.; Gorbunov, A.; Serebryannikova, A.; Gloriozov, I.; Cheshkov, D.; Bezzubov, S.; Chung, W.; Kovalev, V.; Vatsouro, I. Constructing bridged multifunctional calixarenes by intramolecular indole coupling. *Org. Chem. Front.* **2019**, *6*, 3327–3341. https://doi.org/10.1039/c9qo00859d.

S76. Akceylan, E.; Yilmaz, M. Synthesis of water-soluble calixarenes catalyzed one-pot Mannich-type reaction in aqueous media. *Polycycl. Aromat. Compd.* **2016**, *36*, 801–816. https://doi.org/10.1080/10406638.2015.1061025.

S77. Karatavuk, A.O. Novel oxadiazole- and triazole-based calixarene derivatives: synthesis and extraction properties. *Balıkesir Üniversitesi Fen Bilim. Enstitüsü Derg.* **2022**, *24*, 222–231. https://doi.org/10.25092/baunfbed.898019.

S78. Akceylan, E.; Akoz, E.; Sahin, O.; Yilmaz, M. Enantioselective hydrolysis of (R,S)-Naproxen methyl ester using Candida rugosa lipase with calix[4]arene derivatives. *J. Incl. Phenom. Macrocycl. Chem.* **2015**, *81*, 237–243. https://doi.org/10.1007/s10847-014-0452-0.

S79. Budka, J.; Tkadlecová, M.; Lhoták, P.; Stibor, I. Synthesis of novel calixsugars: calix[4]arene–monosaccharide conjugates based on amide bonds. *Tetrahedron* **2000**, *56*, 1883–1887. https://doi.org/10.1016/S0040-4020(00)00086-7.

S80. Dondoni, A.; Hu, X.; Marra, A.; Banks, H.D. First synthesis of bridged and double calixsugars. *Tetrahedron Lett.* **2001**, *42*, 3295–3298. https://doi.org/10.1016/S0040-4039(01)00433-6.

S81. Dessingou, J.; Tabbasum, K.; Mitra, A.; Hinge, V.K.; Rao, C.P. Lower Rim 1,3-di{4-antipyrine}amide conjugate of calix[4]arene: Synthesis, characterization, and selective recognition of Hg^{2+} and its sensitivity toward pyrimidine bases. *J. Org. Chem.* **2012**, *77*, 1406–1413. https://doi.org/10.1021/jo2022372.

S82. Peng, Q.; Zou, J.; Zeng, G.; Wen, Z.; Zheng, W. Stable blue-emitting molecular material derived from calix[4]arene and pyrazoline: Synthesis, optical and electrochemical properties. *Synth. Met.* **2009**, *159*, 1944–1949. https://doi.org/10.1016/j.synthmet.2009.06.019.

S83. Joseph, R.; Ramanujam, B.; Acharya, A.; Rao, C.P. Lower rim 1,3-di{bis(2-picolyl)}amide derivative of calix[4]arene (L) as ratiometric primary sensor toward Ag^{+} and the complex of Ag^{+} as secondary sensor toward Cys: Experimental, computational, and microscopy Studies and INHIBIT logic gate properties of L. *J. Org. Chem.* **2009**, *74*, 8181–8190. https://doi.org/10.1021/jo901676s.

S84. Eymur, S.; Akceylan, E.; Sahin, O.; Uyanik, A.; Yilmaz, M. Direct enantioselective aldol reactions catalyzed by calix[4]arene-based l-proline derivatives in the water. *Tetrahedron* **2014**, *70*, 4471–4477. https://doi.org/10.1016/j.tet.2014.05.034.

S85. Bagatin, I.A.; Toma, H.E. A calix[4]arene receptor modified with 8-hydroxyquinoline for supramolecular energy transfer. *New J. Chem.* **2000**, *24*, 841–844. https://doi.org/10.1039/B005127F.

S86. Liu, Z.; Jiang, L.; Liang, Z.; Gao, Y. A selective colorimetric chemosensor for lanthanide ions. *Tetrahedron* **2006**, *62*, 3214–3220. https://doi.org/10.1016/j.tet.2006.01.073.

S87. Sharma, V.S.; Sharma, A.S.; Shah, A.P.; Shah, P.A.; Shrivastav, P.S.; Athar, M. New class of supramolecular bowl-shaped columnar mesogens derived from thiacalix[4]arene exhibiting gelation and organic light-emitting diodes applications. *ACS Omega* **2019**, *4*, 15862–15872. https://doi.org/10.1021/acsomega.9b01776.

S88. Hadi, H.; Safari, R. Synthesis and experimental/theoretical evaluation of an efficient calix[4]arene based sensor for selective detection of Pt^{2+} ion. *Mol. Phys.* **2022**, *120*, e2108516. https://doi.org/10.1080/00268976.2022.2108516.

S89. Yushkova, E.A.; Stoikov, I.I.; Zhukov, A.Y.; Puplampu, J.B.; Rizvanov, I.K.; Antipin, I.S.; Konovalov, A. Heteroditopic p-tert-butyl thiacalix[4]arenes for creating supramolecular self-assembles by cascade or commutative mechanisms. *RSC Adv.* **2012**, *2*, 3906. https://doi.org/10.1039/C2RA01255C.

S90. Bullough, E.K.; Kilner, C.A.; Little, M.A.; Willans, C.E. Tetrakis(methylimidazole) and tetrakis(methylimidazolium) calix[4]arenes: competitive anion binding and deprotonation. *Org. Biomol. Chem.* **2012**, *10*, 2824. https://doi.org/10.1039/C2OB07025A. S91. Yu, M.; Guo, T.; Shi, X.; Yang, J.; Xu, X.; Ma, J.; Yu, Z. Polyoxometalate-bridged Cu(I)- and Ag(I)-thiacalix[4]arene dimers for heterogeneous catalytic oxidative desulfurization and azide–alkyne "click" reaction. *Inorg. Chem.* **2019**, *58*, 11010–11019. https://doi.org/10.1021/acs.inorgchem.9b01557.

S92. Kotch, F.W.; Sidorov, V.; Lam, Y.; Kayser, K.J.; Li, H.; Kaucher, M.S.; Davis, J.T. Water-mediated association provides an ion pair receptor. *J. Am. Chem. Soc.* **2003**, *125*, 15140–15150. https://doi.org/10.1021/ja0364165.

S93. Meunier, S.J.; Roy, R. Polysialosides scaffolded on p-tert-butylcalix[4]arene. *Tetrahedron Lett.* **1996**, *37*, 5469–5472. https://doi.org/10.1016/0040-4039(96)01167-7.

S94. Roy, R.; Kim, J.M. Amphiphilic p-tert-butylcalix[4]arene scaffolds containing exposed carbohydrate dendrons. *Angew. Chemie Int. Ed.* **1999**, *38*, 369–372. https://doi.org/10.1002/(SICI)1521-3773(19990201)38:3%3C369::AID-ANIE369%3E3.0.CO;2-1.

S95. Stoikov, I.I.; Zhukov, A.Y.; Agafonova, M.N.; Sitdikov, R.R.; Antipin, I.S.; Konovalov, A.I. p-tert-Butyl thiacalix[4]arenes functionalized at the lower rim by o-, m-, p-amido and o-, m-, p-(amidomethyl)pyridine fragments as receptors for α-hydroxy- and dicarboxylic acids. *Tetrahedron* **2010**, *66*, 359–367. https://doi.org/10.1016/j.tet.2009.10.075.

S96. Li, J.; Du, P.; Liu, Y.; Ma, J. Assembly of polyoxometalate-thiacalix[4]arene-based inorganic–organic hybrids as efficient catalytic oxidation desulfurization catalysts. *Dalton Trans.* **2021**, *50*, 1349–1356. https://doi.org/10.1039/D0DT04097E.

S97. Alekseeva, E.A.; Luk'Yanenko, A.P.; Basok, S.S.; Mazepa, A.V.; Gren', A.I. Synthesis of derivatives of p-tert-butylcalix[4]arene containing on lower rim fragments of 2-aminoalkylbenzimidazoles. *Russ. J. Org. Chem.* **2010**, *46*, 1403–1408. https://doi.org/10.1134/S107042801009023X.

S98. Beer, P.D.; Szemes, F.; Passaniti, P.; Maestri, M. Luminescent ruthenium(II) bipyridine−calix[4]arene complexes as receptors for lanthanide cations. *Inorg. Chem.* **2004**, *43*, 3965–3975. https://doi.org/10.1021/ic0499401.

S99. Alekseeva, E.A.; Basok, S.S.; Mazepa, A.V.; Luk'Yanenko, A.P.; Snurnikova, O.V.; Gren', A.I. p-tert-Butylcalix[4]arenes containing azacrown ether substituents at the lower rim as potential polytopic receptors. *Russ. J. Gen. Chem.* **2013**, *83*, 1738–1743. https://doi.org/10.1134/S1070363213090181.

S100. Zhao, B.; Blesa, M.; Mercier, N.; Le derf, F.; Sallé, M. A calixarene–amide–tetrathiafulvalene assembly for the electrochemical detection of anions. *New J. Chem.* **2005**, *29*, 1164. https://doi.org/10.1039/B504339E.

S101. Lyons, D.M.; Mohanraj, J.; Accorsi, G.; Armaroli, N.; Boyd, P.D. A supramolecular porphyrin–ferrocene–fullerene triad. *New J. Chem.* **2011**, *35*, 632. https://doi.org/10.1039/c0nj00825g.

S102. Sutariya, P.G.; Soni, H.; Gandhi, S.A.; Pandya, A. Turn on fluorescence strip based sensor for recognition of Sr^{2+} and CN^{-} via lowerrim substituted calix[4]arene and its computational investigation. *Spectrochim. Acta Part A* **2020**, *238*, 118456. https://doi.org/10.1016/j.saa.2020.118456.

S103. Chinta, J.P.; Dessingou, J.; Rao, C.P. Synthesis, characterization and ion recognition studies of lower rim 1,3-di{rhodamine} conjugate of calix[4]arene. *J. Chem. Sci.* **2013**, *125*, 1455–1461. https://doi.org/10.1007/s12039-013-0500-0.

S104. Colom, E.; Andrés-Castán, J.; Franco, S.; Garín,J.; Montoya, J.; Orduna,J.; Villacampa, B.; Blesa, M. Multichromophoric sensitizers based on calix[4]arene scaffold and 4H-pyranylidene moiety for DSSCs application. *Dyes Pigm.* **2017**, *136*, 505–514. https://doi.org/10.1016/j.dyepig.2016.08.067.

S105. Duerto, I.; Barrios, D.; Garín, J.; Orduna, J.; Villacampa, B.; Blesa, M. Difunctionalized dyes for DSSCs based on two different scaffolds: p-tert-butylcalix[4]arene or isophthalic acid. *Dyes Pigm.* **2020**, *182*, 108530. https://doi.org/10.1016/j.dyepig.2020.108530.

S106. Colom, E.; Andrés-Castán, J.; Barrios, D.; Duerto, I.; Franco, S.; Garín, J.; Orduna, J.; Villacampa, B.; Blesa, M. Modification of the electronic properties of the π-spacer of chromophores linked to calix[4]arene platform for DSSCs applications. *Dyes Pigm.* **2019**, *164*, 43–53. https://doi.org/10.1016/j.dyepig.2018.12.066.

S107. Anandababu, A.; Anandan, S.; Ashokkumar, M. A Simple Discriminating p-tert-butylcalix[4]arene thiospirolactam Rhodamine b based colorimetric and fluorescence sensor for mercury ion and live cell imaging applications. *ChemistrySelect* **2018**, *3*, 4413–4420. https://doi.org/10.1002/slct.201800044.

S108. Lyskawa, J.; Canevet, D.; Allain, M.; Sallé, M. An electron-rich three dimensional receptor based on a calixarene-tetrathiafulvalene assembly. *Tetrahedron Lett.* **2010**, *51*, 5868–5872. https://doi.org/10.1016/j.tetlet.2010.08.112.

S109. Molard, Y.; Bureau, C.; Parrot-Lopez, H.; Lamartine, R.; Regnouf-de-Vains, J. Synthesis of calix[4]arene podands bearing two and four histidine or glycine groups at the lower rim. Complexation properties towards cobalt(II) chloride. *Tetrahedron Lett.* **1999**, *40*, 6383–6387. https://doi.org/10.1016/S0040-4039(99)01243-5.

S110. Marradi, M.; Cicchi, S.; Sansone, F.; Casnati, A.; Goti, A. Low-generation dendrimers with a calixarene core and based on a chiral C2-symmetric pyrrolidine as iminosugar mimics. *Beilstein J. Org. Chem.* **2012**, *8*, 951–957. https://doi.org/10.3762/bjoc.8.107.

S111. Chen, M.; Tang, X.; Li, J.; Chen, F.; Jiang, Z.; Fu, R.; Li, H.; Hu, X.; Geng, W.; Guo, D. Active targeting tumor therapy using host-guest drug delivery system based on biotin functionalized azocalix[4]arene. *J. Control. Release* **2024**, *368*, 691–702. https://doi.org/10.1016/j.jconrel.2024.03.017.

S112. Xu, Z.; Li, G.; Chen, C.; Huang, Z. Inherently chiral calix[4]arene-based bifunctional organocatalysts for enantioselective aldol reactions. *Tetrahedron* **2008**, *64*, 8668–8675. https://doi.org/10.1016/j.tet.2008.07.001.

S113. Xu, Z.; Zhang, C.; Zheng, Q.; Chen, C.; Huang, Z. A new approach to enantiopure inherently chiral calix[4]arenes: Determination of their absolute configurations. *Org. Lett.* **2007**, *9*, 4447–4450. https://doi.org/10.1021/ol701714q.

S114. Zeng, C.; Zheng, Q.; Tang, Y.; Huang, Z. Synthesis of new calix[4]arenes containing nucleoside bases. *Tetrahedron* **2003**, *59*, 2539–2548. https://doi.org/10.1016/S0040-4020(03)00251-5.

S115. Kim, S.J.; Kim, B.H. Design and synthesis of calix[4]arene–nucleoside hybrids. *Tetrahedron Lett.* **2002**, *43*, 6367–6371. https://doi.org/10.1016/S0040-4039(02)01372-2.

S116. Gómez-Machuca, H.; Quiroga-Campano, C.; Jullian, C.; Saitz, C. Design, synthesis and evaluation of a bifunctional receptor based on calix[4]arene bearing benzoxazolinone group. *J. Mol. Struct.* **2024**, *1298*, 137062. https://doi.org/10.1016/j.molstruc.2023.137062.

S117. Sutariya, P.G.; Pandya, A.; Lodha, A.; Menon, S.K. Fluorescence switch on–off–on receptor constructed of quinoline allied calix[4]arene for selective recognition of Cu^{2+} from blood serum and F^- from industrial waste water. *The Analyst* **2013**, *138*, 2531. https://doi.org/10.1039/C3AN00209H.

S118. Wang, Z.; Lu, G.; Guo, X.; Wu, H. Synthesis of calix[4]arene derivatives containing a nucleobase and their interaction with complementary nucleosides at the air–water interface. *Supramol. Chem.* **2003**, *15*, 327–334. https://doi.org/10.1080/1061027031000075700.

S119. Alvarenga, M.E.; Guimarães, F.F.; Da silva, C.M.; Alves, R.B.; Lara, T.V.; Maia, L.J.; De santana, R.C.; Martins, F.T. Joining a host-guest platform and a light-emission motif: Pyrazinamide-calixarene hybrids. *J. Mol. Struct.* **2022**, *1258*, 132694. https://doi.org/10.1016/j.molstruc.2022.132694.

S120. Akceylan, E.; Uyanik, A.; Eymur, S.; Sahin, O.; Yilmaz, M. Calixarene-proline functionalized iron oxide magnetite nanoparticles (Calix-Pro-MN): An efficient recyclable organocatalyst for asymmetric aldol reaction in water. *Appl. Catal. A: General* **2015**, *499*, 205–212. https://doi.org/10.1016/j.apcata.2015.04.018.

S121. Vyas, G.; Bhatt, S.; Paul, P. Synthesis of calixarene-capped silver nanoparticles for colorimetric and amperometric detection of mercury (Hg^{II}, Hg^0). *ACS Omega* **2019**, *4*, 3860–3870. https://doi.org/10.1021/acsomega.8b03299.

S122. Shi, H.; Shi, X.; Yao, T.; Ji, L. Study of the molecular recognition of nucleotides and bases by a novel calixarene derivative containing uracil. *Chinese J. Chem.* **2008**, *26*, 170–174. https://doi.org/10.1002/cjoc.200890016.

S123. Sharma, V.S.; Patel, U.; Thakar, S.; Rathod, S.L.; Sharma, A.S.; Shrivastav, P.S.; Athar, M. Fluorescein-appended calixarene-functionalized supramolecular AIE-active liquid crystalline materials for self-assembly and bio-imaging applications. *New J. Chem.* **2023**, *47*, 1444–1459. https://doi.org/10.1039/d2nj05133h.

S124. Sharma, V.S.; Shah, A.P.; Sharma, A.S.; Athar, M. Columnar self-assembly, gelation and electrochemical behavior of cone-shaped luminescent supramolecular calix[4]arene LCs based on oxadiazole and thiadiazole derivatives. *New J. Chem.* **2019**, *43*, 1910–1925. https://doi.org/10.1039/c8nj04922j.

S125. Pathak, R.K.; Hinge, V.K.; Mondal, P.; Rao, C.P. Ratiometric fluorescence off-on-off sensor for Cu^{2+} in aqueous buffer by a lower rim triazole linked benzimidazole conjugate of calix[4]arene. *Dalton Trans.* **2012**, *41*, 10652. https://doi.org/10.1039/c2dt30432e.

S126. Csávás, M.; Malinovská, L.; Perret, F.; Gyurkó, M.; Illyés, Z.T.; Wimmerová, M.; Borbás, A. Tri- and tetravalent mannoclusters cross-link and aggregate BC2L-A lectin from Burkholderia cenocepacia. *Carbohydr. Res.* **2017**, *437*, 1–8. https://doi.org/10.1016/j.carres.2016.11.008.

S127. Kašáková, M.; Malinovská, L.; Klejch, T.; Hlaváčková, M.; Dvořáková, H.; Fujdiarová, E.; Rottnerová, Z.; Maťátková, O.; Lhoták, P.; Wimmerová, M.; Moravcová, J. Selectivity of original C-hexopyranosyl calix[4]arene conjugates towards lectins of different origin. *Carbohydr. Res.* **2018**, *469*, 60–72. https://doi.org/10.1016/j.carres.2018.08.012.

S128. Ling, I.; Hashim, R.; Sabah, K.J. Sugar thiacrown-ether appended calix[4]arene as a selective chemosensor for Fe^{2+} and Fe^{3+} ions. *RSC Adv.* **2015**, *5*, 88038–88044. https://doi.org/10.1039/C5RA15448K.

S129. Palmioli, A.; Moretti, L.; Vezzoni, C.A.; Legnani, L.; Sperandeo, P.; Baldini, L.; Sansone, F.; Airoldi, C.; Casnati, A. Multivalent calix[4]arene-based mannosylated dendrons as new FimH ligands and inhibitors. *Bioorg. Chem.* **2023**, *138*, 106613. https://doi.org/10.1016/j.bioorg.2023.106613.

S130. Zhao, B.; Zhu, X.; Chen, X.; Yan, Z.; Zhu, W. Novel clicked tetrathiafulvalene-calix[4]arene assemblies: Synthesis and intermolecular electron transfer toward p-chloranil. *Chinese Chem. Lett.* **2013**, *24*, 573–577. https://doi.org/10.1016/j.cclet.2013.04.026.

S131. Zhao, B.; Peng, Q.; Zhu, X.; Yan, Z.; Zhu, W. Metal-promoted intermolecular electron transfer in tetrathiafulvalene thiacalix[4]arene conjugates and tetrachlorobenzoquinone. *J. Org. Chem.* **2015**, *80*, 1052–1058. https://doi.org/10.1021/jo502390z.

S132. Brissonnet, Y.; Ortiz mellet, C.; Morandat, S.; Garcia moreno, M.I.; Deniaud, D.; Matthews, S.E.; Vidal, S.; Šesták, S.; Elikat, K.; Gouin, S.G. Topological effects and binding modes operating with multivalent iminosugar-based glycoclusters and mannosidases. *J. Am. Chem. Soc.* **2013**, *135*, 18427–18435. https://doi.org/10.1021/ja406931w.

S133. Sakamoto, S.; Fujii, S.; Yoshida, K.; Sakurai, K. Supramolecular chirality: Vesicle-to-chiral helix transition of the micelles consisting of a sugar-bearing calix[4]arene surfactant. *Langmuir* **2016**, *32*, 12434–12441. https://doi.org/10.1021/acs.langmuir.6b01671.

S134. Hosseinzadeh, R.; Domehri, E.; Tajbakhsh, M.; Bekhradnia, A. New fluorescent sensor based on a calix[4]arene bearing two triazole–coumarin units for copper ions: application for Cu^{2+} detection in human blood serum. *J. Incl. Phenom. Macrocycl. Chem.* **2019**, *93*, 245–252. https://doi.org/10.1007/s10847-018-0872-3.

S135. Zhan, J.; Wen, L.; Miao, F.; Tian, D.; Zhu, X.; Li, H. Synthesis of a pyridyl-appended calix[4]arene and its application to the modification of silver nanoparticles as an Fe^{3+} colorimetric sensor. *New J. Chem.* **2012**, *36*, 656–661. https://doi.org/10.1039/c2nj20776a.

S136. Tian, D.; Yan, H.; Li, H. A selective fluorescent probe of Hg^{2+} based on triazole-linked 8-oxyquinoline calix[4]arene by click chemistry. *Supramol. Chem.* **2010**, *22*, 249–255. https://doi.org/10.1080/10610270903410504.

S137. Cindro, N.; Požar, J.; Barišić, D.; Bregović, N.; Pičuljan, K.; Tomaš, R.; Frkanec, L.; Tomišić, V. Neutral glycoconjugated amide-based calix[4]arenes: complexation of alkali metal cations in water. *Org. Biomol. Chem.* **2018**, *16*, 904–912. https://doi.org/10.1039/c7ob02955a.

S138. Galante, E.; Geraci, C.; Sciuto, S.; Campo, V.L.; Carvalho, I.; Sesti-Costa, R.; Guedes, P.M.; Silva, J.S.; Hill, L.; Nepogodiev, S.A.; Field, R.A. Glycoclusters presenting lactose on calix[4]arene cores display trypanocidal activity. *Tetrahedron* **2011**, *67*, 5902–5912. https://doi.org/10.1016/j.tet.2011.06.065.

S139. Mironova, D.; Makarov, E.; Bilyukova, I.; Akyol, K.; Sultanova, E.; Evtugyn, V.; Davletshin, D.; Gilyazova, E.; Bulatov, E.; Burilov, V.; Solovieva, S.; Antipin, I. Aggregation, cytotoxicity and DNA binding in a series of calix[4]arene amphiphile containing aminotriazole groups. *Pharmaceuticals* **2023**, *16*, 699. https://doi.org/10.3390/ph16050699.

S140. Bitter, I.; Grün, A.; Tőke, L.; Tóth, G.; Balázs, B.; Mohammed Ziegler, I.; Grofcsik, A.; Kubinyi, M. Novel chromogenic pyridinium derivatives of calix[4]arenes,II. *Tetrahedron* **1997**, *53*, 16867–16876. https://doi.org/10.1016/S0040-4020(97)10121-1.

S141. Rathod, S.L.; Sharma, V.S.; Sharma, A.S.; Athar, M.; Shrivastav, P.S.; Parekh, H.M. Blue light-emitting quinoline armed thiacalix[4]arene 3D-scaffold: A systematic platform to construct fluorescent liquid crystals with bio-imaging applications. *J. Mol. Struct.* **2022**, *1270*, 133830. https://doi.org/10.1016/j.molstruc.2022.133830.

S142. Pathak, R.K.; Tabbasum, K.; Hinge, V.K.; Rao, C.P. Selective recognition of cysteine in its free and protein-bound states by the Zn^{2+} complex of a triazole-based calix[4]arene conjugate. *Chem. – Eur. J.* **2011**, *17*, 13999–14003. https://doi.org/10.1002/chem.201102791.

S143. Pathak, R.K.; Hinge, V.K.; Rai, A.; Panda, D.; Rao, C.P. Imino–phenolic–pyridyl conjugates of calix[4]arene (L_1 and L_2) as primary fluorescence switch-on sensors for Zn^{2+} in Solution and in HeLa cells and the recognition of pyrophosphate and ATP by $[ZnL_2]$. *Inorg. Chem.* **2012**, *51*, 4994–5005. https://doi.org/10.1021/ic202426v.

S144. Pathak, R.K.; Hinge, V.K.; Mondal, M.; Rao, C.P. Triazole-linked-thiophene conjugate of calix[4]arene: Its selective recognition of Zn^{2+} and as biomimetic model in supporting the events of the metal detoxification and oxidative stress involving metallothionein. *J. Org. Chem.* **2011**, *76*, 10039–10049. https://doi.org/10.1021/jo201865x.

S145. Klimentová, J.; Vojtíšek, P. New receptors for anions in water: Synthesis, characterization, X-ray structures of new derivatives of 5,11,17,23-tetraamino-25,26,27,28-tetrapropyloxycalix[4]arene. *J. Mol. Struct.* **2007**, *826*, 48–63. https://doi.org/10.1016/j.molstruc.2006.04.016.
S146. Yilmaz, B.; Keskinates, M.; Bayrakci, M. Novel integrated sensing system of calixarene and rhodamine molecules for selective colorimetric and fluorometric detection of Hg^{2+} ions in living cells. *Spectrochim. Acta Part A* **2021**, *245*, 118904. https://doi.org/10.1016/j.saa.2020.118904.
S147. Podyachev, S.N.; Sudakova, S.N.; Syakaev, V.V.; Burmakina, N.E.; Konovalov, A.I. Synthesis of new hydrazones based on tetrathiacalix[4]arene in the 1,3-alternate conformation. *Russ. J. Org. Chem.* **2010**, *46*, 1162–1166. https://doi.org/10.1134/S1070428010080087.
S148. Mohamad, A.D.; Omran, O.A. Synthesis, characterization of novel p-tert-butylthiacalix[4]arene derivatives and its application for extraction of alkali and transition elements. *J. Iran. Chem. Soc.* **2019**, *16*, 2197–2202. https://doi.org/10.1007/s13738-019-01691-6.
S149. Gafiatullin, B.K.; Paskevich, I.V.; Burilov, V.A.; Solovieva, S.E.; Antipin, I.S. One-pot synthesis of mono-substituted quaternized p-tert- butylthiacalix[4]arenes. *Macroheterocycles* **2022**, *15*, 53–58. https://doi.org/10.6060/mhc214097b.
S150. Brenner, E.; Matt, D.; Henrion, M.; Teci, M.; Toupet, L. Calix[4]arenes with one and two N-linked imidazolium units as precursors of N-heterocyclic carbene complexes. Coordination chemistry and use in Suzuki–Miyaura cross-coupling. *Dalton Trans.* **2011**, *40*, 9889. https://doi.org/10.1039/C1DT10838G.
S151. Alishahi, N.; Mohammadpoor-baltork, I.; Tangestaninejad, S.; Mirkhani, V.; Moghadam, M.; Kia, R. Calixarene based ionic liquid as an efficient and reusable catalyst for one-pot multicomponent synthesis of polysubstituted pyridines and bis-pyridines. *ChemistrySelect* **2019**, *4*, 5903–5910. https://doi.org/10.1002/slct.201900902.
S152. Bozkurt, S.; Durmaz, M.; Yilmaz, M.; Sirit, A. Calixarene-based chiral phase-transfer catalysts derived from cinchona alkaloids for enantioselective synthesis of α-amino acids. *Tetrahedron Asymmetry* **2008**, *19*, 618–623. https://doi.org/10.1016/j.tetasy.2008.02.006.
S153. Burilov, V.A.; Garipova, R.I.; Solovieva, S.E.; Antipin, I.S. Synthesis of bifunctional derivatives of calix[4]arene bearing azidoalkyl fragments in cone stereoisomeric form. *Dokl. Chem.* **2020**, *490*, 1–5. https://doi.org/10.1134/S0012500820010012.
S154. Gafiatullin, B.K.; Radaev, D.D.; Osipova, M.V.; Sultanova, E.D.; Burilov, V.A.; Solovieva, S.E.; Antipin, I.S. Amphiphilic N-oligoethyleneglycol-imidazolium derivatives of p-tert-butylthiacalix[4]arene: Synthesis, aggregation and interaction with DNA. *Macroheterocycles* **2021**, *14*, 171–179. https://doi.org/10.6060/mhc210439s.
S155. Sultanova, E.D.; Gafiatullin, B.K.; Ocherednyuk, E.A.; Garipova, R.I.; Volodina, A.A.; Daminova, A.G.; Evtugyn, V.G.; Burilov, V.A.; Solovieva, S.E.; Antipin, I.S. N-Oxyethylimidazolium calix[4]arenes and thiacalix[4]arenes: Difference in solubilization property and detection of adenine-containing nucleotides. *Macroheterocycles* **2023**, *16*, 168–176. https://doi.org/10.6060/mhc235118s.
S156. Burilov, V.A.; Bogdanov, I.M.; Garipova, R.I.; Volodina, A.A.; Mironova, D.A.; Evtugyn, V.G.; Solovieva, S.E.; Antipin, I.S. New bifunctional amphiphilic oxyethylimidazolium derivatives of calix[4]arene containing alkynyl/azide fragments: regularities of aggregation and polymerization under azide/alkyne cycloaddition conditions. *Russ. Chem. Bull.* **2022**, *71*, 131–138. https://doi.org/10.1007/s11172-022-3386-5.
S157. Burilov, V.; Mironova, D.; Sultanova, E.; Garipova, R.; Evtugyn, V.; Solovieva, S.; Antipin, I. NHC Polymeric particles obtained by self-assembly and click approach of calix[4]arene amphiphiles as support for catalytically active Pd nanoclusters. *Molecules* **2021**, *26*, 6864. https://doi.org/10.3390/molecules26226864.
S158. Burilov, V.A.; Ibragimova, R.R.; Gafiatullin, B.H.; Nugmanov, R.I.; Solovieva, S.E.; Antipin, I.S. Unusual reactivity of aliphatic and aromatic amines with bromoalkyl derivatives of thiacalix[4]arene in 1,3-alternate stereoisomeric form. *Macroheterocycles* **2017**, *10*, 215–220. https://doi.org/10.6060/mhc170514b.
S159. Burilov, V.A.; Mironova, D.A.; Ibragimova, R.R.; Evtugyn, V.G.; Osin, Y.N.; Solovieva, S.E.; Antipin, I.S. Imidazolium p-tert-butylthiacalix[4]arene amphiphiles—aggregation in water solutions and binding with adenosine 5′-triphosphate dipotassium salt. *BioNanoScience* **2018**, *8*, 337–343. https://doi.org/10.1007/s12668-017-0484-1.
S160. Fahlbusch, T.; Frank, M.; Maas, G.; Schatz, J. N-Heterocyclic carbene complexes of mercury, silver, iridium, platinum, ruthenium, and palladium based on the calix[4]arene skeleton. *Organometallics* **2009**, *28*, 6183–6193. https://doi.org/10.1021/om900642x.
S161. Burilov, V.A.; Garipova, R.I.; Sultanova, E.D.; Mironova, D.A.; Evtugyn, V.G.; Osin, Y.N.; Solovieva, S.E.; Antipin, I.S. Synthesis, Aggregation behavior, and catalytic activity in the Ullmann reaction of amphiphilic p-tert-butylthiacalix[4]arene with azidoalkylimidazolium moieties. *Macroheterocycles* **2019**, *12*, 340–345. https://doi.org/10.6060/mhc191174b.
S162. Danac, R.; Rusu, R.; Rotaru, A.; Pui, A.; Shova, S. New conjugates of calix[4]arenes bearing dipyridine and indolizine heterocycles. *Supramol. Chem.* **2012**, *24*, 424–435. https://doi.org/10.1080/10610278.2012.688122.

S163. Andreyko, E.A.; Padnya, P.L.; Stoikov, I.I. Supramolecular self-assembly of water-soluble nanoparticles based on amphiphilic p-tert-butylthiacalix[4]arenes with silver nitrate and fluorescein. *Colloids Surf., A* **2014**, *454*, 74–83. https://doi.org/10.1016/j.colsurfa.2014.04.021.

S164. Yildirim, A.; Bhatti, A.A.; Uysal, A.; Yilmaz, M. Dual fluorescence response of calix[4]arene-1,8-naphthalimide derivatives towards Hg(II) /Cr(VI) and their antimicrobial studies of transparent biofilms with hyaluronic acid. *Int. J. Biol. Macromol.* **2024**, *273*, 132955. https://doi.org/10.1016/j.ijbiomac.2024.132955.

S165. Joseph, R.; Ramanujam, B.; Acharya, A.; Khutia, A.; Rao, C.P. Experimental and computational studies of selective recognition of Hg^{2+} by amide linked lower rim 1,3-dibenzimidazole derivative of calix[4]arene: Species characterization in solution and that in the isolated complex, including the delineation of the nanostructures. *J. Org. Chem.* **2008**, *73*, 5745–5758. https://doi.org/10.1021/jo800073g.

S166. Alizada, M.; Gul, A.; Oguz, M.; Kursunlu, A.N.; Yilmaz, M. Ion sensing of sister sensors based-on calix[4]arene in aqueous medium and their bioimaging applications. *Dyes Pigm.* **2021**, *184*, 108741. https://doi.org/10.1016/j.dyepig.2020.108741.

S167. Yu, M.; Liu, B.; Guo, J.; Wu, F. A sustainable modificatory 1,2-alternate thiacalix[4]arene for detection of silver ion. *Dyes Pigm.* **2023**, *210*, 110983. https://doi.org/10.1016/j.dyepig.2022.110983.

S168. Fateh, F.; Yildirim, A.; Bhatti, A.A.; Yilmaz, M. A new benzothiazin-functionalized calix[4]arene-based fluorescent chemosensor for the selective detection of Co^{2+} Ion. *J. Fluoresc.* **2021**, *31*, 1075–1083. https://doi.org/10.1007/s10895-021-02745-w.

S169. Morakot, N.; Tomapatanaget, B.; Ngeon-Tae, W.; Aeungmaitrepirom, W.; Tuntulani, T. Synthesis and fluorescence sensing properties of calix[4]arenes containing fluorophores. *Supramol. Chem.* **2005**, *17*, 655–659. https://doi.org/10.1080/10610270500142484.

S170. Bingol, H.; Kocabas, E.; Zor, E.; Coskun, A. A novel benzothiazole based azocalix[4]arene as a highly selective chromogenic chemosensor for Hg^{2+} ion: A rapid test application in aqueous environment. *Talanta* **2010**, *82*, 1538–1542. https://doi.org/10.1016/j.talanta.2010.07.047.

S171. Erdemir, S.; Aydin, D.; Oguz, M. A Ratiometric and switchable fluorescent sensor based on calix[4]arene-HBT with a large stokes shift for the real-time detecting of Zn^{2+} and Cu^{2+} in living cells. *IEEE Sensors J.* **2023**, *23*, 6734–6741. https://doi.org/10.1109/JSEN.2023.3244323.

S172. Ochoa, A.; Hernández-Arancibia, B.; Herrera-Muñoz, J.; Gómez-Machuca, H.; Saitz, C. Thiosemicarbazone- and thiourea-functionalized calix[4]arenes in cone and 1,3-alternate conformations: recezptors for the recognition of ions. *Chemosensors* **2025**, *13*, 48. https://doi.org/10.3390/chemosensors13020048.

S173. Dinda, S.K.; Upadhyay, A.; Polepalli, S.; Althaf hussain, M.; Rao, C.P. Development of nitrobenzoxadiazole-appended calix[4]arene conjugate (L) for selective sensing of trivalent Cr^{3+}, Fe^{3+}, and Al^{3+} ions in solution and in the solid state and imaging MCF7 cells by {L + Al^{3+}}. *ACS Omega* **2019**, *4*, 7723–7733. https://doi.org/10.1021/acsomega.9b00582.

S174. De gaetano, Y.; Clarot, I.; Regnouf-De-Vains, J. Selective complexation and extraction of Ag(I) and Zn(II) versus Pb(II) by polymer beads modified by attachment of a bipyridyl–calixarene-based chelate. *New J. Chem.* **2012**, *36*, 1339. https://doi.org/10.1039/C2NJ40083A.

S175. Casnati, A.; Baldini, L.; Sansone, F.; Ungaro, R.; Armaroli, N.; Pompei, D.; Barigelletti, F. Synthesis, complexation and photophysics in protic solvents of lanthanide complexes of novel calix[4]arene polycarboxylic-2,2′-bipyridine mixed ligands. *Supramol. Chem.* **2002**, *14*, 281–289. https://doi.org/10.1080/10610270290026194.

S176. Kim, H.J.; Kim, J.S. BODIPY appended cone-calix[4]arene: selective fluorescence changes upon Ca^{2+} binding. *Tetrahedron Lett.* **2006**, *47*, 7051–7055. https://doi.org/10.1016/j.tetlet.2006.07.088.

S177. Lorente, A.; Ochoa, A.; Rodriguez-Lavado, J.; Rodriguez-Nuévalos, S.; Jaque, P.; Gil, S.; Sáez, J.A.; Costero, A.M. Unconventional OFF–ON response of a mono(calix[4]arene)-substituted BODIPY sensor for Hg^{2+} through dimerization reversion. *ACS Omega* **2023**, *8*, 819–828. https://doi.org/10.1021/acsomega.2c06161.

S178. Sulak, M.; Kursunlu, A.N.; Girgin, B.; Karakuş, Ö.Ö.; Güler, E. A highly selective fluorescent sensor for mercury (II) ion based on BODIPY and calix[4]arene bearing triazolenaphthylene groups; synthesis and photophysical investigations. *J. Photochem. Photobiol. A: Chemistry* **2017**, *349*, 129–137. https://doi.org/10.1016/j.jphotochem.2017.09.022.

S179. Yuan, M.; Zhou, W.; Liu, X.; Zhu, M.; Li, J.; Yin, X.; Zheng, H.; Zuo, Z.; Ouyang, C.; Liu, H.; Li, Y.; Zhu, D. A multianalyte chemosensor on a single molecule: Promising structure for an integrated logic gate. *J. Org. Chem.* **2008**, *73*, 5008–5014. https://doi.org/10.1021/jo8005683.

S180. Costa, A.I.; Barata, P.D.; Fialho, C.B.; Prata, J.V. Highly sensitive and selective fluorescent probes for Cu(II) detection based on calix[4]arene-oxacyclophane architectures. *Molecules* **2020**, *25*, 2456. https://doi.org/10.3390/molecules25102456.

S181. Uttam, B.; Jahan, I.; Sen, S.; Rao, C.P. Coumarin–calix[4]arene conjugate-anchored SiO_2 nanoparticles as an ultrasensor material for Fe^{3+} to work in water, in serum, and in biological cells. *ACS Omega* **2020**, *5*, 21288–21299. https://doi.org/10.1021/acsomega.0c03373.

S182. Samanta, K.; Rao, C.P. A Bifunctional thioether linked coumarin appended calix[4]arene acquires selectivity toward Cu^{2+} sensing on going from solution to SAM on gold. *ACS Appl. Mater. Interfaces* **2016**, *8*, 3135–3142. https://doi.org/10.1021/acsami.5b10481.
S183. Chawla, H.M.; Shukla, R.; Pandey, S. Novel fluorescein appended calix[4]arenes for preferential recognition of Cu^{2+} ions. *Tetrahedron Lett.* **2013**, *54*, 2063–2066. https://doi.org/10.1016/j.tetlet.2013.02.017.
S184. Noamane, M.H.; Ben othmen, A.; Al-Ayed, A.S.; Kim, Y.; Hayami, S.; Hamdi, A. Interaction of 2-furanylmethyl- and 2-thienylmethyl-amide derivatives of 1,3-di(carboxymethyl)calix[4]arene with metal salts. *J. Incl. Phenom. Macrocycl. Chem.* **2019**, *94*, 249–256. https://doi.org/10.1007/s10847-019-00908-8.
S185. Maurin, A.; Varatharajan, S.; Colasson, B.; Reinaud, O. A water-soluble calix[4]arene-based ligand for the selective linear coordination and stabilization of copper(I) ion in aerobic conditions. *Org. Lett.* **2014**, *16*, 5426–5429. https://doi.org/10.1021/ol502650c. S186. Bocheńska, M.; Zielińska, A.; Pomećko, R.; HubscheBruder, V.; Arnaud-Neu, F. Lower rim substituted tert-butyl calix[4]arene (part VII): Ionophoric properties of calix[4]arene-crown-6 derivatives in plasticized PVC-membrane electrodes and in solution. *J. Incl. Phenom. Macrocycl. Chem.* **2005**, *52*, 129–134. https://doi.org/10.1007/s10847-004-6332-2.
S187. Sayin, S.; Cay, S. Novel An efficient fluorescent probe based on calix[4]triazacrown-5 with naphthalimide group for Co^{2+}, Cd^{2+} and dopamine detection. *J. Fluoresc.* **2024**, *34*, 729–741. https://doi.org/10.1007/S10895-023-03314-Z.
S188. Xu, Z.; Kim, S.; Kim, H.N.; Han, S.J.; Lee, C.; Kim, J.S.; Qian, X.; Yoon, J. A naphthalimide–calixarene as a two-faced and highly selective fluorescent chemosensor for Cu^{2+} or *Tetrahedron Lett.* **2007**, *48*, 9151–9154. https://doi.org/10.1016/j.tetlet.2007.10.109.
S189. Modrušan, M.; Cindro, N.; Usenik, A.; Leko, K.; Glazer, L.; Tomaš, R.; Horvat, G.; Požar, J.; Tomišić, V. Complexation of alkali and alkaline earth metal cations by fluorescent glycoconjugated calix[4]arene derivative: Thermodynamic and computational studies. *Croat. Chem. Acta* **2024**, *97*, 189–203. https://doi.org/10.5562/cca4115.
S190. Usenik, A.; Modrušan, M.; Leko, K.; Borovec, J.; Marinac, S.; Hok, L.; Cindro, N.; Vianello, R.; Horvat, G.; Požar, J.; Hrenar, T.; Tomišić, V. A combined thermodynamic and computational study of alkaline earth metal cations complexation by a fluorescent calix[4]arene receptor. *Int. J. Mol. Sci.* **2025**, *26*, 1264. https://doi.org/10.3390/ijms26031264.
S191. Erdemir, S.; Malkondu, S.; Kocyigit, O. A reversible calix[4]arene armed phenolphthalein based fluorescent probe for the detection of Zn^{2+} and an application in living cells. *Luminescence* **2019**, *34*, 106–112. https://doi.org/10.1002/bio.3585.
S192. Sun, Q.; Mu, L.; Zeng, X.; Zhao, J.; Yamato, T.; Zhang, J. Synthesis and fluorescence properties of a 1,3-disubstituted thiacalix[4]arene crown-5 armed with phenothiazine moieties. *Sci. China Chem.* **2015**, *58*, 539–544. https://doi.org/10.1007/s11426-014-5202-z.
S193. Banthia, S.; Samanta, A. Calix[4]azacrown and 4-aminophthalimide-appended calix[4]azacrown: Synthesis, structure, complexation and fluorescence signaling behaviour. *Org. Biomol. Chem.* **2005**, *3*, 1428. https://doi.org/10.1039/B500912J.
S194. Kalinin, V.; Rasperetikhina, E.; Bukharov, M.; Shibaeva, K.; Islamov, D.; Babaeva, O.; Stoikov, I. Synthesis and selective Cu(II) complexation of lower rim substituted thiacalixarenes containing pyrazole fragments. *Chim. Techno Acta* **2024**, *11*, 202411418. https://doi.org/10.15826/chimtech.2024.11.4.18.
S195. Chen, Y.; Chen, M.; Lee, K.; Shen, L.; Hung, H.; Niu, H.; Chung, W. 1,3-Alternate calix[4]arene functionalized with pyrazole and triazole ligands as a highly selective fluorescent sensor for Hg^{2+} and Ag^{+} ions. *Front. Chem.* **2020**, *8*, 593261. https://doi.org/10.3389/fchem.2020.593261.
S196. de Namor, A.F.D.; Rawi, N.A.; Piro, O.E.; Castellano, E.E.; Gil, E. New lower rim calix(4)arene derivatives with mixed pendent arms and their complexation properties for alkali-metal cations. Structural, electrochemical, and thermodynamic characterization. *J. Phys. Chem. B* **2002**, *106*, 779–787. https://doi.org/10.1021/jp0131976.
S197. Joseph, R.; Ramanujam, B.; Acharya, A.; Rao, C.P. Fluorescence switch-on sensor for Cu^{2+} by an amide linked lower rim 1,3-bis(2-picolyl)amine derivative of calix[4]arene in aqueous methanol. *Tetrahedron Lett.* **2009**, *50*, 2735–2739. https://doi.org/10.1016/j.tetlet.2009.03.109.
S198. Yamato, T.; Pérez-Casas, C.; Yoshizawa, A.; Rahman, S.; Elsegood, M.R.; Redshaw, C. Hydrogen bonding receptors of tetraamide derivatives derived from thiacalix[4]arene in cone- and 1,3-alternate conformation. *J. Incl. Phenom. Macrocycl. Chem.* **2009**, *63*, 301–308. https://doi.org/10.1007/s10847-008-9523-4.
S199. Mohammed-Ziegler, I. Selective complex formation of some chromogenic calix[4]arene derivatives detected by Fourier transform infrared spectroscopy 1. Solution experiments. *Spectrochim. Acta Part A* **2003**, *59*, 19–27. https://doi.org/10.1016/S1386-1425(02)00123-3.
S200. Sayin, S. A highly selective fluorescence probe for Co^{2+} or Cu^{2+} detection based on a new tetraquinoline-substituted calix[4]arene derivative. *Tetrahedron* **2022**, *122*, 132960. https://doi.org/10.1016/j.tet.2022.132960.
S201. Sayin, S. Synthesis of new quinoline-conjugated calixarene as a fluorescent sensor for selective determination of Cu^{2+} ion. *J. Fluoresc.* **2021**, *31*, 1143–1151. https://doi.org/10.1007/S10895-021-02749-6.

S202. Bhatti, A.A.; Oguz, M.; Yilmaz, M. New water soluble p-sulphonatocalix[4]arene chemosensor appended with rhodamine for selective detection of Hg^{2+} ion. *J. Mol. Struct.* **2020**, *1203*, 127436. https://doi.org/10.1016/j.molstruc.2019.127436.

S203. Bono, N.; Pennetta, C.; Sganappa, A.; Giupponi, E.; Sansone, F.; Volonterio, A.; Candiani, G. Design and synthesis of biologically active cationic amphiphiles built on the calix[4]arene scaffold. *Int. J. Pharm.* **2018**, *549*, 436–445. https://doi.org/10.1016/j.ijpharm.2018.08.020.

S204. Molard, Y.; Parrot-Lopez, H. Molecular scaffolds for di-metallic complexation: the synthesis, characterisation and complexation properties of tetrakis-terpyridinyl-calix[4]arene. *Tetrahedron Lett.* **2002**, *43*, 6355–6358. https://doi.org/10.1016/S0040-4039(02)01386-2.

S205. Nehra, A.; Hinge, V.K.; Rao, C.P. Phenylene-diimine-capped conjugate of lower rim 1,3-calix[4]arene as molecular receptor for Mg^{2+} via arm conformational changes followed by aggregation and mimicking the species by molecular mechanics. *J. Org. Chem.* **2014**, *79*, 5763–5770. https://doi.org/10.1021/jo500892r.

S206. Nag, R.; Vashishtha, M.; Rao, C.P. Switching the ion selectivity from Fe^{3+} to Al^{3+} by a triazole-appended calix[4]arene-based amphiphile. *ChemistrySelect* **2018**, *3*, 1248–1256. https://doi.org/10.1002/slct.201702999.

S207. Mummidivarapu, V.V.; Hinge, V.K.; Tabbasum, K.; Gonnade, R.G.; Rao, C.P. Triazole-linked anthracenyl-appended calix[4]arene conjugate as receptor for Co(II): Synthesis, spectroscopy, microscopy, and computational studies. *J. Org. Chem.* **2013**, *78*, 3570–3576. https://doi.org/10.1021/jo400038x.

S208. Pathak, R.K.; Ibrahim, S.; Rao, C.P. Selective recognition of Zn^{2+} by salicylaldimine appended triazole-linked di-derivatives of calix[4]arene by enhanced fluorescence emission in aqueous-organic solutions: Role of terminal –CH_2OH moietieefs in conjunction with the imine in recognition. *Tetrahedron Lett.* **2009**, *50*, 2730–2734. https://doi.org/10.1016/j.tetlet.2009.03.126.

S209. Pathak, R.K.; Dikundwar, A.G.; Row, T.N.; Rao, C.P. A lower rim triazole linked calix[4]arene conjugate as a fluorescence switch on sensor for Zn^{2+} in blood serum milieu. *Chem. Commun.* **2010**, *46*, 4345. https://doi.org/10.1039/C0CC00219D.

S210. Zhan, J.; Fang, F.; Tian, D.; Li, H. Anthraquinone-modified calix[4]arene: Click synthesis, selective calcium ion fluorescent chemosensor and INHIBIT logic gate. *Supramol. Chem.* **2012**, *24*, 272–278. https://doi.org/10.1080/10610278.2012.656124.

S211. Sreedevi, P.; Nair, J.B.; Preethanuj, P.; Jeeja, B.S.; Suresh, C.H.; Maiti, K.K.; Varma, R.L. Calix[4]arene based redox sensitive molecular probe for SERS guided recognition of labile iron pool in tumor cells. *Anal. Chem.* **2018**, *90*, 7148–7153. https://doi.org/10.1021/acs.analchem.8b01982.

S212. Aljabri, M.D.; Rahman, S.; Georghiou, P.E. Synthesis, complexation and DFT computational studies of bis(naphthyl)methane-"capped" triazole-linked calix[4]arenes as Fe^{3+} fluorescent chemosensors. *ChemistrySelect* **2017**, *2*, 1214–1218. https://doi.org/10.1002/slct.201601923.

S213. Ho, I.; Haung, K.; Chung, W. 1,3-Alternate calix[4]arene as a homobinuclear ditopic fluorescent chemosensor for Ag^{+} ions. *Chem. – Asian J.* **2011**, *6*, 2738–2746. https://doi.org/10.1002/asia.201100023.

S214. Wang, N.; Sun, C.; Chung, W. A highly selective fluorescent chemosensor for Ag^{+} based on calix[4]arene with lower-rim proximal triazolylpyrenes. *Sensors Actuators B: Chemical* **2012**, *171-172*, 984–993. https://doi.org/10.1016/j.snb.2012.06.014.

S215. Park, S.Y.; Yoon, J.H.; Hong, C.S.; Souane, R.; Kim, J.S.; Matthews, S.E.; Vicens, J. A Pyrenyl-appended triazole-based calix[4]arene as a fluorescent sensor for Cd^{2+} and Zn^{2+}. *J. Org. Chem.* **2008**, *73*, 8212–8218. https://doi.org/10.1021/jo8012918.

S216. Rusu, R.; Szumna, A.; Rosu, N.; Dumea, C.; Danac, R. New triazole appended tert-butyl calix[4]arene conjugates: Synthesis, Hg^{2+} binding studies. *Tetrahedron* **2015**, *71*, 2922–2926. https://doi.org/10.1016/j.tet.2015.03.060.

S217. Zhu, L.; Gong, S.; Gong, S.; Yang, C.; Qin, J. Novel Pyrene-armed calix[4]arenes through triazole connection: Ratiometric fluorescent chemosensor for Zn^{2+} and promising structure for integrated logic gates. *Chinese J. Chem.* **2008**, *26*, 1424–1430. https://doi.org/10.1002/cjoc.200890259.

S218. Rahman, S.; Assiri, Y.; Alodhayb, A.N.; Beaulieu, L.Y.; Oraby, A.K.; Georghiou, P.E. Naphthyl "capped" triazole-linked calix[4]arene hosts as fluorescent chemosensors towards $Fe^{3+}$and Hg^{2+}: an experimental and DFT computational study. *New J. Chem.* **2016**, *40*, 434–440. https://doi.org/10.1039/C5NJ01362C.

S219. Alodhayb, A.N.; Braim, M.; Beaulieu, L.Y.; Valluru, G.; Rahman, S.; Oraby, A.K.; Georghiou, P.E. Metal ion binding properties of a bimodal triazolyl-functionalized calix[4]arene on a multi-array microcantilever system. Synthesis, fluorescence and DFT computation studies. *RSC Adv.* **2016**, *6*, 4387–4396. https://doi.org/10.1039/C5RA12685A.

S220. Ni, X.; Zeng, X.; Redshaw, C.; Yamato, T. Synthesis and evaluation of a novel pyrenyl-appended triazole-based thiacalix[4]arene as a fluorescent sensor for Ag^{+} ion. *Tetrahedron* **2011**, *67*, 3248–3253. https://doi.org/10.1016/j.tet.2011.03.008.

S221. Požar, J.; Cvetnić, M.; Usenik, A.; Cindro, N.; Horvat, G.; Leko, K.; Modrušan, M.; Tomišić, V. The role of triazole and glucose moieties in alkali metal cation complexation by lower-rim tertiary-amide calix[4]arene derivatives. *Molecules* **2022**, *27*, 470. https://doi.org/10.3390/molecules27020470.

S222. Nag, R.; Polepalli, S.; Althaf hussain, M.; Rao, C.P. Ratiometric Cu^{2+} binding, cell Imaging, mitochondrial targeting, and anticancer activity with nanomolar IC50 by spiro-indoline-conjugated calix[4]arene. *ACS Omega* **2019**, *4*, 13231–13240. https://doi.org/10.1021/acsomega.9b01402.

S223. Souchon, V.; Maisonneuve, S.; David, O.; Leray, I.; Xie, J.; Valeur, B. Photophysics of cyclic multichromophoric systems based on β-cyclodextrin and calix[4]arene with appended pyridin-2′-yl-1,2,3-triazolegroups. *Photochem. Photobiol. Sci.* **2008**, *7*, 1323–1331. https://doi.org/10.1039/b810465d.
S224. Qing, G.; He, Y.; Wang, F.; Qin, H.; Hu, C.; Yang, X. Enantioselective fluorescent sensors for chiral carboxylates based on calix[4]arenes bearing an L-tryptophan unit. *Eur. J. Org. Chem.* **2007**, *2007*, 1768–1778. https://doi.org/10.1002/ejoc.200600917.
S225. Sansone, F.; Baldini, L.; Casnati, A.; Lazzarotto, M.; Ugozzoli, F.; Ungaro, R. Biomimetic macrocyclic receptors for carboxylate anion recognition based on C-linked peptidocalix[4]arenes. *Proc. Natl. Acad. Sci.* **2002**, *99*, 4842–4847. https://doi.org/10.1073/pnas.062625499.
S226. Joseph, R.; Ramanujam, B.; Pal, H.; Rao, C.P. Lower rim 1,3-di-amide-derivative of calix[4]arene possessing bis-{N-(2,2′-dipyridylamide)} pendants: a dual fluorescence sensor for Zn^{2+} and Ni^{2+}. *Tetrahedron Lett.* **2008**, *49*, 6257–6261. https://doi.org/10.1016/j.tetlet.2008.08.049.
S227. Kim, J.; Park, S.; Kim, S.; Thuery, P.; Souane, R.; Matthews, S.E.; Vicens, J. A pyrenyl-appended triazole-based calix[4]arene as a fluorescent sensor for iodide ion. *Bull. Korean Chem. Soc.* **2010**, *31*, 624–629. https://doi.org/10.5012/bkcs.2010.31.03.624.
S228. Hosseinzadeh, R.; Maliji, F.; Golchoubian, H.; Bekhradnia, A. A novel ferrocene-based calix[4]arene as an efficient optical and electrochemical sensor for highly selective fluoride recognition. *ChemistrySelect* **2019**, *4*, 3914–3920. https://doi.org/10.1002/slct.201900241.
S229. Yang, F.; Liu, W.; Xie, J.; Bai, X.; Guo, H. Novel deep-cavity calix[4]arene derivatives with large s-triazine conjugate systems: synthesis and complexation for dyes. *J. Incl. Phenom. Macrocycl. Chem.* **2013**, *76*, 311–316. https://doi.org/10.1007/s10847-012-0200-2.
S230. Simsir, E.A.; Erdemir, S.; Tabakci, M.; Tabakci, B. Nano-scale selective and sensitive optical sensor for metronidazole based on fluorescence quenching: 1H-Phenanthro[9,10-d]imidazolyl-calix[4]arene fluorescent probe. *Anal. Chim. Acta* **2021**, *1162*, 338494. https://doi.org/10.1016/j.aca.2021.338494.
S231. Kandpal, M.; Bandela, A.K.; Hinge, V.K.; Rao, V.R.; Rao, C.P. Fluorescence and piezoresistive cantilever sensing of trinitrotoluene by an upper-rim tetrabenzimidazole conjugate of calix[4]arene and delineation of the features of the complex by molecular dynamics. *ACS Appl. Mater. Interfaces* **2013**, *5*, 13448–13456. https://doi.org/10.1021/am404356v.
S232. Barata, P.D.; Costa, A.I.; Costa, S.; Prata, J.V. Fluorescent bis-calix[4]arene-carbazole conjugates: Synthesis and inclusion complexation studies with fullerenes C60 and C70. *Molecules* **2021**, *26*, 5000. https://doi.org/10.3390/molecules26165000.
S233. Prata, J.V.; Barata, P.D. Fostering protein–calixarene interactions: From molecular recognition to sensing. *RSC Adv.* **2016**, *6*, 1659–1669. https://doi.org/10.1039/C5RA19887A.
S234. Bandela, A.K.; Bandaru, S.; Rao, C.P. A Fluorescent 1,3-diaminonaphthalimide conjugate of calix[4]arene for sensitive and selective detection of trinitrophenol: Spectroscopy, microscopy, and computational Studies, and its applicability using cellulose strips. *Chem. – Eur. J.* **2015**, *21*, 13364–13374. https://doi.org/10.1002/chem.201500787.
S235. Bandela, A.; Chinta, J.P.; Hinge, V.K.; Dikundwar, A.G.; Row, T.N.; Rao, C.P. Recognition of polycyclic aromatic hydrocarbons and their derivatives by the 1,3-dinaphthalimide conjugate of calix[4]arene: Emission, absorption, crystal structures, and computational studies. *J. Org. Chem.* **2011**, *76*, 1742–1750. https://doi.org/10.1021/jo1023409.
S236. Andreyko, E.A.; Padnya, P.L.; Stoikov, I.I. Amphiphilic p-tert-butylthiacalix[4]arenes containing quaternary ammonium groups: from small molecules toward water-soluble nanoscale associates. *J. Phys. Org. Chem.* **2015**, *28*, 527–535. https://doi.org/10.1002/poc.3433.
S237. Düker, M.H.; Schäfer, H.; Zeller, M.; Azov, V.A. Rationally designed calix[4]arene–pyrrolotetrathiafulvalene receptors for electron-deficient neutral guests. *J. Org. Chem.* **2013**, *78*, 4905–4912. https://doi.org/10.1021/jo400502t.
S238. Burilov, V.; Mironova, D.A.; Ibragimova, R.R.; Solovieva, S.E.; Antipin, I.S. Interactions of new bis-ammonium thiacalix[4]arene derivatives in 1,3-alternate stereoisomeric form with bovine serum albumin. *BioNanoScience* **2016**, *6*, 427–430. https://doi.org/10.1007/s12668-016-0255-4.
S239. Mummidivarapu, V.V.S.; Hinge, V.K.; Samanta, K.; Yarramala, D.S.; Rao, C.P. Supramolecular complexation of biological phosphates with an acyclic triazolium-linked anthracenyl-1,3-diconjugate of calix[4]arene: Synthesis, characterization, spectroscopy, microscopy, and computational studies. *Chem. – Eur. J.* **2014**, *20*, 14378–14386. https://doi.org/10.1002/chem.201403353.
S240. Zhan, J.; Zhu, X.; Fang, F.; Miao, F.; Tian, D.; Li, H. Sensitive fluorescence sensor for nitroaniline isomers based on calix[4]arene bearing naphthyl groups. *Tetrahedron* **2012**, *68*, 5579–5582. https://doi.org/10.1016/j.tet.2012.04.076.
S241. Zeng, X.; Weng, L.; Chen, L.; Leng, X.; Ju, H.; He, X.; Zhang, Z. The synthesis of some pyridyl functionalized calix[4]arenes as the sensor molecule for silver ion-selective electrodes. *J. Chem. Soc. Perkin Trans. 2* **2001**, *30*, 545–549. https://doi.org/10.1039/B008114K.
S242. Chen, L.; Zeng, X.; He, X.; Zhengzhi; Zhang. Selective electrodes for silver based on polymeric membranes containing calix[4]arene derivatives. *Fresenius' J. Anal. Chem.* **2000**, *367*, 535–538. https://doi.org/10.1007/s002160000397.

S243. Zeng, X.; Weng, L.; Chen, L.; Leng, X.; Zhang, Z.; He, X. Improved silver ion-selective electrodes using novel 1,3-bis(2-benzothiazolyl)thioalkoxy-p-tert-butylcalix[4]arenes. *Tetrahedron Lett.* **2000**, *41*, 4917–4921. https://doi.org/10.1016/S0040-4039(00)00746-2.

S244. Chen, L.; Ju, H.; Zeng, X.; He, X.; Zhang, Z. Silver ion-selective electrodes based on novel containing benzothiazolyl calix[4]arene. *Anal. Chim. Acta* **2001**, *437*, 191–197. https://doi.org/10.1016/S0003-2670(01)00994-1.

S245. Lu, J.; Pang, D.; Zeng, X.; He, X. A new solid-state silver ion-selective electrode based on a novel tweezer-type calixarene derivative. *J. Electroanal. Chem.* **2004**, *568*, 37–43. https://doi.org/10.1016/j.jelechem.2004.01.017.

S246. Mahajan, R.; Kaur, I.; Kumar, M. Silver ion-selective electrodes employing Schiff base p-tert-butyl calix[4]arene derivatives as neutral carriers. *Sensors Actuators B: Chemical* **2003**, *91*, 26–31. https://doi.org/10.1016/S0925-4005(03)00062-5.

S247. Chen, L.; He, X.; Zhao, B.; Liu, Y. Calixarene derivative as the neutral carrier in silver ion-selective electrode and liquid membrane transport. *Anal. Chim. Acta* **2000**, *417*, 51–56. https://doi.org/10.1016/S0003-2670(00)00912-0.

S248. Evtugyn, G.; Stoikov, I.; Beljyakova, S.; Shamagsumova, R.; Stoikova, E.; Zhukov, A.; Antipin, I.; Budnikov, H. Ag selective electrode based on glassy carbon electrode covered with polyaniline and thiacalix[4]arene as neutral carrier. *Talanta* **2007**, *71*, 1720–1727. https://doi.org/10.1016/j.talanta.2006.08.004.

S249. Lu, J.; Tong, X.; He, X. A mercury ion-selective electrode based on a calixarene derivative containing the thiazole azo group. *J. Electroanal. Chem.* **2003**, *540*, 111–117. https://doi.org/10.1016/S0022-0728(02)01298-6.

S250. Liu, Y.; Zhao, B.T.; Zhang, H.Y.; Ju, H.F.; Chen, L.X.; He, X.W. Molecular design of calixarene, part 4, synthesis of novel double-armed p-(tert-butyl)calix[4]arene-derived amides and their lead(II)(Pb^{2+})-selective-electrode properties. *Helv. Chim. Acta* **2001**, *84*, 1969–1975. https://doi.org/10.1002/1522-2675(20010711)84:7%3C1969::AID-HLCA1969%3E3.0.CO;2-Q.

S251. Chen, L.; Zhang, J.; Zhao, W.; He, X.; Liu, Y. Double-armed calix[4]arene amide derivatives as ionophores for lead ion-selective electrodes. *J. Electroanal. Chem.* **2006**, *589*, 106–111. https://doi.org/10.1016/j.jelechem.2006.02.001.

S252. Torma, F.; Grün, A.; Bitter, I.; Tóth, K. Calixarene/Nafion-modified bismuth-film electrodes for adsorptive stripping voltammetric determination of lead. *Electroanalysis* **2009**, *21*, 1961–1969. https://doi.org/10.1002/elan.200904634.

S253. Kumar, P. All solid state nickel(II)-selective potentiometric sensor based on an upper rim substituted calixarene. *Electroanalysis* **2012**, *24*, 2005–2012. https://doi.org/10.1002/elan.201200228.

S254. Kurzątkowska, K.; Sayin, S.; Yilmaz, M.; Radecka, H.; Radecki, J. Calix[4]arene derivatives as dopamine hosts in electrochemical sensors. *Sensors Actuators B: Chemical* **2015**, *218*, 111–121. https://doi.org/10.1016/j.snb.2015.04.110.

S255. Wang, L.; Pan, Y.; Li, Z.; Chen, J. Highly enantioselective direct aldol reactions catalyzed by proline derivatives based on a calix[4]arene scaffold in the presence of water. *Synlett* **2009**, *2009*, 2356–2360. https://doi.org/10.1055/s-0029-1217710.

S256. Uyanik, A.; Bayrakci, M.; Eymur, S.; Yilmaz, M. Upper rim-functionalized calix[4]arene-based l-proline as organocatalyst for direct asymmetric aldol reactions in water and organic media. *Tetrahedron* **2014**, *70*, 9307–9313. https://doi.org/10.1016/j.tet.2014.10.063.

S257. Li, Z.; Lu, C.; Huang, G.; Ma, J.; Sun, H.; Wang, L.; Pan, Y. Novel prolinamide organocatalysts based on calix[4]arene scaffold for the enantioselective direct aldol reaction. *Lett. Org. Chem.* **2010**, *7*, 461–466. https://doi.org/10.2174/157017810791824919.

S258. Burilov, V.A.; Gafiatullin, M.B.; Mironova, D.A.; Sultanova, E.D.; Evtugyn, V.G.; Osin, Y.N.; Islamov, D.R.; Usachev, K.S.; Solovieva, S.E.; Antipin, I.S. Amphiphilic Pd^{II}-NHC complexes on 1,3-alternate p-tert-butylthiacalix[4]arene platform: Synthesis and catalytic activities in coupling and hydrogenation reactions. *Eur. J. Org. Chem.* **2020**, *2020*, 2180–2189. https://doi.org/10.1002/ejoc.202000059.

S259. Gafiatullin, B.; Akchurina, A.; Fedoseeva, A.; Sultanova, E.; Islamov, D.; Usachev, K.; Burilov, V.; Solovieva, S.; Antipin, I. PEPPSI-type Pd(II)—NHC complexes on the base of p-tert-butylthiacalix[4]arene: Synthesis and catalytic activities. *Inorganics* **2023**, *11*, 326. https://doi.org/10.3390/inorganics11080326.

S260. Fatykhova, G.A.; Makarov, E.G.; Mironova, D.A.; Sultanova, E.D.; Burilov, V.A.; Solovieva, S.E.; Antipin, I.S. New Amphiphilic calix[4]Arene derivatives with 4,5-dicarboxytriazolyl fragments: Synthesis and use in micellar catalysis. *Russ. J. Phys. Chem. B* **2019**, *13*, 401–407. https://doi.org/10.1134/S1990793119030163.

S261. Burilov, V.; Fatykhova, A.; Mironova, D.; Sultanova, E.; Nugmanov, R.; Artemenko, A.; Volodina, A.; Daminova, A.; Evtugyn, V.; Solovieva, S.; Antipin, I. Oxyethylated fluoresceine—(thia)calix[4]arene conjugates: Synthesis and visible-light photoredox catalysis in water–organic media. *Molecules* **2022**, *28*, 261. https://doi.org/10.3390/molecules28010261.

S262. Dibama, H.M.; Clarot, I.; Fontanay, S.; Salem, A.B.; Mourer, M.; Finance, C.; Duval, R.E.; Regnouf-de-Vains, J. Towards calixarene-based prodrugs: Drug release and antibacterial behaviour of a water-soluble nalidixic acid/calix[4]arene ester adduct. *Bioorg. Med. Chem. Lett.* **2009**, *19*, 2679–2682. https://doi.org/10.1016/j.bmcl.2009.03.139.

S263. Sautrey, G.; Clarot, I.; Rogalska, E.; Regnouf-de-Vains, J. New potential prodrugs of aciclovir using calix[4]arene as a lipophilic carrier: synthesis and drug-release studies at the air–water interface. *New J. Chem.* **2012**, *36*, 2060. https://doi.org/10.1039/C2NJ40338B.

S264. An, L.; Liu, J.; Peng, X.; Zheng, Y.; Wang, C.; Huang, T. Preparation and in vitro bioactivity evaluation of N-heterocyclic-linked dihomooxacalix[4]arene derivatives. *RSC Adv.* **2019**, *9*, 41287–41297. https://doi.org/10.1039/c9ra06876g.

S265. Patel, M.B.; Modi, N.R.; Raval, J.P.; Menon, S.K. Calix[4]arene based 1,3,4-oxadiazole and thiadiazole derivatives: Design, synthesis, and biological evaluation. *Org. Biomol. Chem.* **2012**, *10*, 1785. https://doi.org/10.1039/C2OB06730G.

S266. Agrahari, A.K.; Singh, A.K.; Singh, A.S.; Singh, M.; Maji, P.; Yadav, S.; Rajkhowa, S.; Prakash, P.; Tiwari, V.K. Click inspired synthesis of p-tert -butyl calix[4]arene tethered benzotriazolyl dendrimers and their evaluation as anti-bacterial and anti-biofilm agents. *New J. Chem.* **2020**, *44*, 19300–19313. https://doi.org/10.1039/D0NJ02591G.

S267. Pur, F.N.; Dilmaghani, K.A. Calixpenams: Synthesis, characterization, and biological evaluation of penicillins V and X clustered by calixarene scaffold. *Turkish J. Chem.* **2014**, *38*, 288–296. https://doi.org/10.3906/kim-1307-32.

S268. Pur, F.N.; Dilmaghani, K.A. Calixcephems: Clustered cephalosporins analogous to calixpenams as novel potential anti-MRSA agents. *Turkish J. Chem.* **2014**, *38*, 850–858. https://doi.org/10.3906/kim-1403-2.

S269. Fang, S.; Dang, Y.; Li, H.; Li, H.; Liu, J.; Zhong, R.; Chen, Y.; Liu, S.; Lin, S. Membrane-active antibacterial agents based on calix[4]arene derivatives: Synthesis and biological evaluation. *Front. Chem.* **2022**, *10*, 816741. https://doi.org/10.3389/fchem.2022.816741.

S270. Melezhyk, I.O.; Rodik, R.V.; Iavorska, N.V.; Klymchenko, A.S.; Mely, Y.; Shepelevych, V.V.; Skivka, L.M.; Kalchenko, V.I. Antibacterial properties of tetraalkylammonium and imidazolium tetraalkoxycalix[4]arene derivatives. *Anti-infective Agents* **2015**, *13*, 87–94. https://doi.org/10.2174/2211352513666150327002110.

S271. Özkan, Ş.Ç.; Yilmaz, A.; Arslan, E.; Açık, L.; Sayın, Ü.; Mutlu, E.G. Novel copper(II) complexes of p-tert-butylcalix[4]arene diamide derivatives: synthesis, antimicrobial and DNA cleavage activities. *Supramol. Chem.* **2015**, *27*, 255–267. https://doi.org/10.1080/10610278.2014.978868.

S272. Mourer, M.; Fontanay, S.; Duval, R.E.; Regnouf-de-Vains, J. Synthesis, characterization, and biological evaluation as antibacterial agents of water-soluble calix[4]arenes and phenol derivatives incorporating carboxylate groups. *Helvetica Chim. Acta* **2012**, *95*, 1373–1386. https://doi.org/10.1002/hlca.201200044.

S273. Mourer, M.; Dibama, H.M.; Constant, P.; Daffé, M.; Regnouf-de-Vains, J. Anti-mycobacterial activities of some cationic and anionic calix[4]arene derivatives. *Bioorg. Med. Chem.* **2012**, *20*, 2035–2041. https://doi.org/10.1016/j.bmc.2012.01.041.

S274. Geller, C.; Fontanay, S.; Mourer, M.; Dibama, H.M.; Regnouf-de-Vains, J.; Finance, C.; Duval, R. Antiseptic properties of two calix[4]arenes derivatives on the human coronavirus 229E. *Antivir. Res.* **2010**, *88*, 343–346. https://doi.org/10.1016/j.antiviral.2010.09.009.

S275. Parsajam, H.; Dilmaghani, K.A.; Zonouz, A.M. Synthesis, in-vitro and molecular docking studies of 3,4- dihydropyrmidine-2-(1H) one-based calix[4]arenes as antimicrobial agent and kinase inhibitor. *J. Mol. Struct.* **2024**, *1312*, 138597. https://doi.org/10.1016/j.molstruc.2024.138597.

S276. Oguz, M.; Yildirim, A.; Durmus, I.M.; Karakurt, S.; Yilmaz, M. Synthesis of new calix[4]arene derivatives and evaluation of their cytotoxic activity. *Med. Chem. Res.* **2022**, *31*, 52–59. https://doi.org/10.1007/s00044-021-02813-7.

S277. Oguz, A.; Uysal, A.; Özkan, B.N.; Oguz, M.; Yilmaz, M. Isatin-modified calixarene derivatives: A comprehensive study on synthesis, enzyme inhibition, antioxidant, antimicrobial, and antiproliferative activities. *Bioorg. Chem.* **2025**, *157*, 108280. https://doi.org/10.1016/j.bioorg.2025.108280.

S278. Muravev, A.A.; Voloshina, A.D.; Sapunova, A.S.; Gabdrakhmanova, F.B.; Lenina, O.A.; Petrov, K.A.; Shityakov, S.; Skorb, E.V.; Solovieva, S.E.; Antipin, I.S. Calix[4]arene–pyrazole conjugates as potential cancer therapeutics. *Bioorg. Chem.* **2023**, *139*, 106742. https://doi.org/10.1016/j.bioorg.2023.106742.

S279. Uysal akku¸, G.; Al, E.; Korcan, S.E. Selective extraction of toxic heavy metals and biological activity studies using pyrimidylthioamide functionalised calix[4]arene. *Supramol. Chem.* **2015**, *27*, 522–526. https://doi.org/10.1080/10610278.2015.1020944.

S280. Mostovaya, O.; Shiabiev, I.; Ovchinnikov, D.; Pysin, D.; Mukhametzyanov, T.; Stanavaya, A.; Abashkin, V.; Shcharbin, D.; Khannanov, A.; Kutyreva, M.; Shen, M.; Shi, X.; Padnya, P.; Stoikov, I. PAMAM-calix-dendrimers: Third generation synthesis and impact of generation and macrocyclic core conformation on hemotoxicity and calf thymus DNA binding. *Pharmaceutics* **2024**, *16*, 1379. https://doi.org/10.3390/pharmaceutics16111379.

S281. Akhmedov, A.; Terenteva, O.; Subakaeva, E.; Zelenikhin, P.; Shurpik, R.; Shurpik, D.; Padnya, P.; Stoikov, I. Fluorescein-labeled thiacalix[4]arenes as potential theranostic molecules: Synthesis, self-association, and antitumor activity. *Pharmaceutics* **2022**, *14*, 2340. https://doi.org/10.3390/pharmaceutics14112340.

S282. Stoikov, I.I.; Yushkova, E.A.; Antipin, I.S.; Konovalov, A.I. Synthesis of silver and lithium sub-micro- and nanoparticles coated with derivatives of p-tert-butyl thiacalix[4]arenes. *J. Nanoparticle Res.* **2011**, *13*, 6603–6611. https://doi.org/10.1007/s11051-011-0568-8.
